# A study of the photochemical behavior of terarylenes containing allomaltol and pyrazole fragments

**DOI:** 10.3762/bjoc.18.61

**Published:** 2022-05-27

**Authors:** Constantine V Milyutin, Andrey N Komogortsev, Boris V Lichitsky, Valeriya G Melekhina

**Affiliations:** 1 N.D. Zelinsky Institute of Organic Chemistry, Russian Academy of Sciences, Leninsky Pr., 47, Moscow 119991, Russian Federation

**Keywords:** allomaltol, ESIPT process, photochemistry, pyrazoles, terarylenes

## Abstract

The photochemical properties behavior of 3-hydroxy-4-pyranone containing terarylenes with a pyrazole bridge fragment were studied. It was shown that UV-induced 6π-electrocyclization of the 1,3,5-hexatriene system was not observed for the considered objects molecules. At the same time, the phototransformation of such systems proceeds exclusively in the direction of the contraction of the pyranone ring leading to unstable α-hydroxydiketones. For the first time the possibility of isolation of the resulting α-hydroxydiketones in pure form was demonstrated. Wherein, it was shown that relatively low stable α-hydroxydiketones can be trapped by reaction with 1,2-phenylenediamine. The general method for the preparation of the corresponding quinoxalines on the basis of the aforementioned condensation was implemented. It was demonstrated that the studied photoreaction does not depend on the type of pyrazole bridge. The structures of three of synthesized products were established by X-ray diffraction.

## Introduction

The investigation of the photochemical behavior of organic products is a significant part of modern materials science and technology. The UV-induced processes are extensively employed in synthetic organic chemistry [1–7]. The advantage of photochemical methods is the possibility of the preparation of diverse products that are problematic to obtain using other chemical approaches. It should be noted that light can be considered as a traceless agent, therefore, UV-induced processes are of considerable interest in the context of green chemistry [8–9]. At the same time in some cases UV irradiation of organic compounds leads to the formation of highly reactive intermediates. Such objects may, possessing a specific reactivity, which define their further application in organic synthesis. Thus, an actual problem is to study the photochemical behavior of various compounds capable of photogeneration of these unstable intermediates. Moreover, the results of such investigations can be used for the development of UV-promoted synthetic methods.

Among the photochemical processes used in organic synthesis, 6π-electrocyclization of 1,3,5-hexatriene systems is attracting considerable attention [10–17]. The photocyclization of terarylenes leading to the formation of polyheterocyclic compounds is one of the most widely studied processes of this type. Due to the wide variety of this products and their synthetic availability, terarylenes are convenient models for a detailed study of the correlation between the structure of such objects and their photochemical properties [18–21]. As a rule, the phototransformation of terarylenes proceeds in two stages: 6π-electrocyclization of 1,3,5-hexatriene systems and subsequent aromatization of the central benzene ring.

**Scheme 1:**
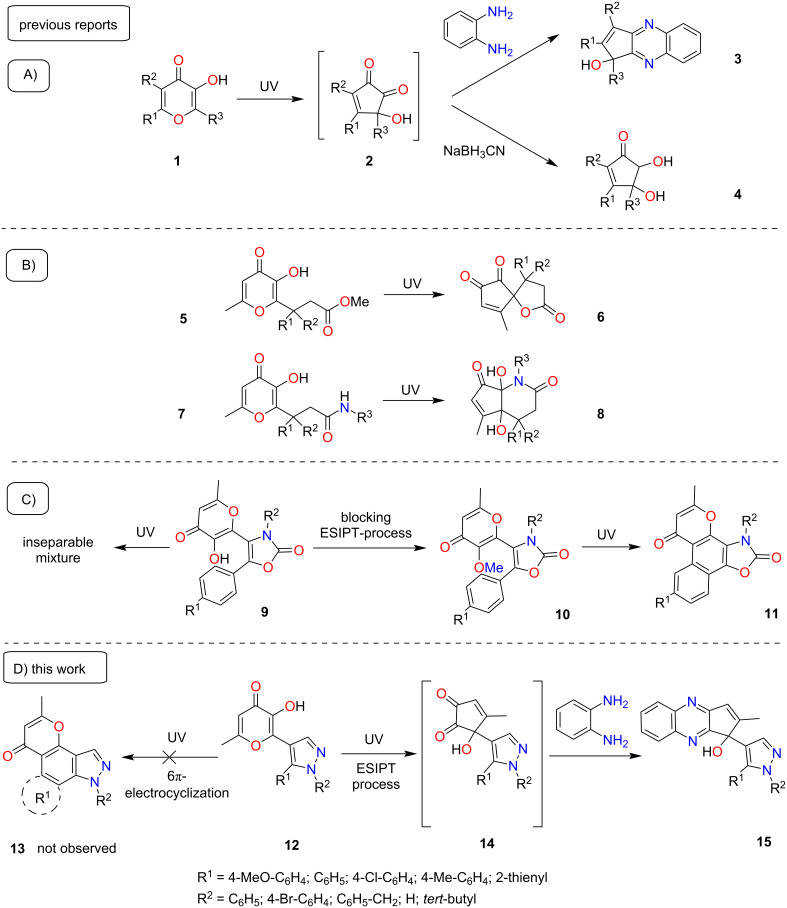
Photochemical transformations of 3-hydroxypyran-4-one derivatives.

Among the significant diversity of terarylenes, compounds containing a 5-hydroxy-2-methyl-4*H*-pyran-4-one (allomaltol) fragment are of particular interest. For such systems two types of photoreactions can occur under UV irradiation: the classical photocyclization of the 1,3,5-hexatriene system and excited state intramolecular proton transfer (ESIPT) – promoted photoprocesses typical for a 3-hydroxypyran-4-one core. For the ESIPT-induced reactions the general direction is the contraction of the 4-pyranone cycle **1** leading to the formation of unstable α-hydroxy-1,2-diketones. These intermediates can be captured intramolecularly using various functional groups present in the molecule or by employment of additional reagents. For example, a convenient method for trapping of unstable α-hydroxy-1,2-diketones **2** is the condensation with 1,2-phenylenediamine resulting in the formation of fused quinoxalines **3** (Scheme 1A) [22]. Another variant of similar catching reaction is reduction by NaBH_3_CN with the formation of diols **4** (Scheme 1A) [23]. Thus, the introduction of additional reagents allows the use of photogenerated α-hydroxy-1,2-diketones in the synthesis of various products. An alternative approach for trapping of such unstable intermediates is the preliminary introduction of functional groups into the 3-hydroxypyran-4-one structure. In this case, the intermediate α-hydroxy-1,2-diketone is captured by intramolecular reaction with an active substituent in the side chain (Scheme 1B) [24–25]. Thus, the combination of ESIPT-induced photoreactions of 3-hydroxypyran-4-one derivatives and various trapping methods opens up significant synthetic possibilities. At the same time, such processes can be complicated if additional photosensitive fragments are presented in the structure. For example, we previously studied the photochemical behavior of oxazolone terarylenes **9** containing the allomaltol fragment (Scheme 1C) [26]. It has been shown that UV irradiation of such systems leads to a complex mixture of products. At the same time the blocking of ESIPT-processes by the modification of the hydroxy group allows one to direct the photoreaction exclusively to the pathway of 6π-electrocyclization of the 1,3,5-hexatriene system. Thus, the study of the photochemical properties of similar bifunctional objects provides access to novel UV irradiation-based synthetic methods.

Continuing our studies devoted to the photochemistry of terarylenes [27–32], in this communication we studied the UV-promoted reaction of pyrazole derivatives **12** containing a 3-hydroxypyran-4-one fragment (Scheme 1D). In contrast to the previously described phototransformation of analogously substituted oxazolones **9**, in the considered case, the photoreaction proceeds exclusively as a contraction of the pyranone ring, while 6π-electrocyclization of the hexatriene system was not observed.

## Results and Discussion

**Scheme 2:**
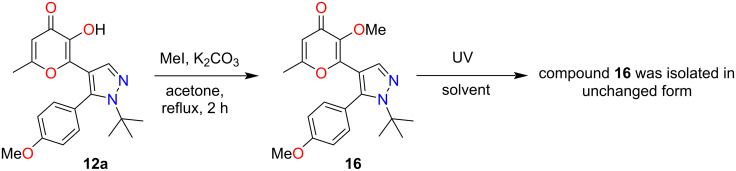
Synthesis and study of the photochemical behavior of compound **16**.

The starting pyrazoles **12** containing the 3-hydroxypyran-4-one fragment were obtained by a literature method [33]. The methoxy derivative **16** was synthesized by methylation of the corresponding pyrazole **12a** according to the standard procedure [24,26] (Scheme 2). Initially, based on the previously obtained results for terarylenes with an oxazolone bridge fragment, we chose pyrazole **16** containing a methoxy group in the allomaltol core as a model object for studying photochemical properties. As it was shown previously for similar oxazolone derivatives [26] the presence of a methoxyl substituent in the compound **16** must exclude the occurrence of ESIPT-promoted photoprocesses. Therefore, it could be expected that UV irradiation of such a compound would lead only to 6π-electrocyclization of the 1,3,5-hexatriene system. Initially, the photochemical behavior of pyrazole **16** was studied using NMR spectroscopy in a solution of DMSO-*d*_6_. UV irradiation at a wavelength of 365 nm for 48 h in an NMR tube did not lead to any transformations of the starting pyrazole **16**. Our attempts to carry out the photocyclization of pyrazole **16** in various solvents (DMF, acetonitrile, toluene, ethanol, methylene chloride, dioxane) were also unsuccessful. In all cases, pyrazole **16** was isolated unchanged. At the same time similar results were obtained employing UV irradiation at a wavelength of 312 nm. Thus, based on the data presented above, we can conclude that for terarylenes containing a pyrazole bridge fragment and a pyran-4-one substituent, 6π-electrocyclization of 1,3,5-hexatriene system does not occur (Scheme 2).

**Scheme 3:**
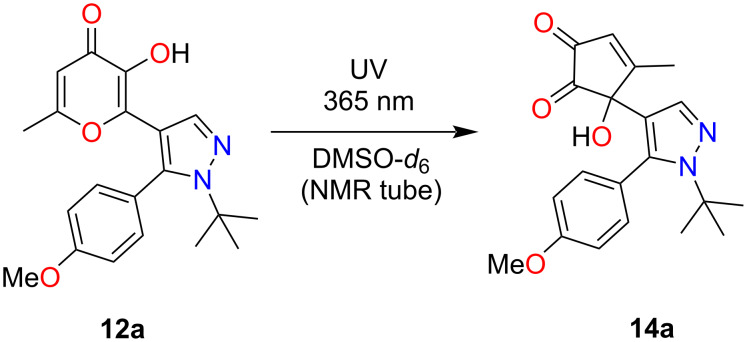
Photoreaction of compound **12a**.

As it was demonstrated earlier [26], UV irradiation of terarylenes containing oxazolone and 3-hydroxypyran-4-one fragments **9** can lead to two types of photoprocesses. Based on the aforementioned results we can assume that the key difference of the related pyrazole derivatives considered in this report is the absence of a pathway associated with 6π-electrocyclization. Therefore, we supposed that for the corresponding pyrazoles **12** containing a hydroxy group in the allomaltol fragment only ESIPT-induced contraction of the pyranone ring will proceed under UV irradiation. At first, pyrazole **12a** was selected as a model object for the investigation of this hypothesis. It was shown that the photoreaction of pyrazole **12a** regiospecifically led to the formation of α-hydroxy-1,2-diketone **14a** (Scheme 3).

**Figure 1:**
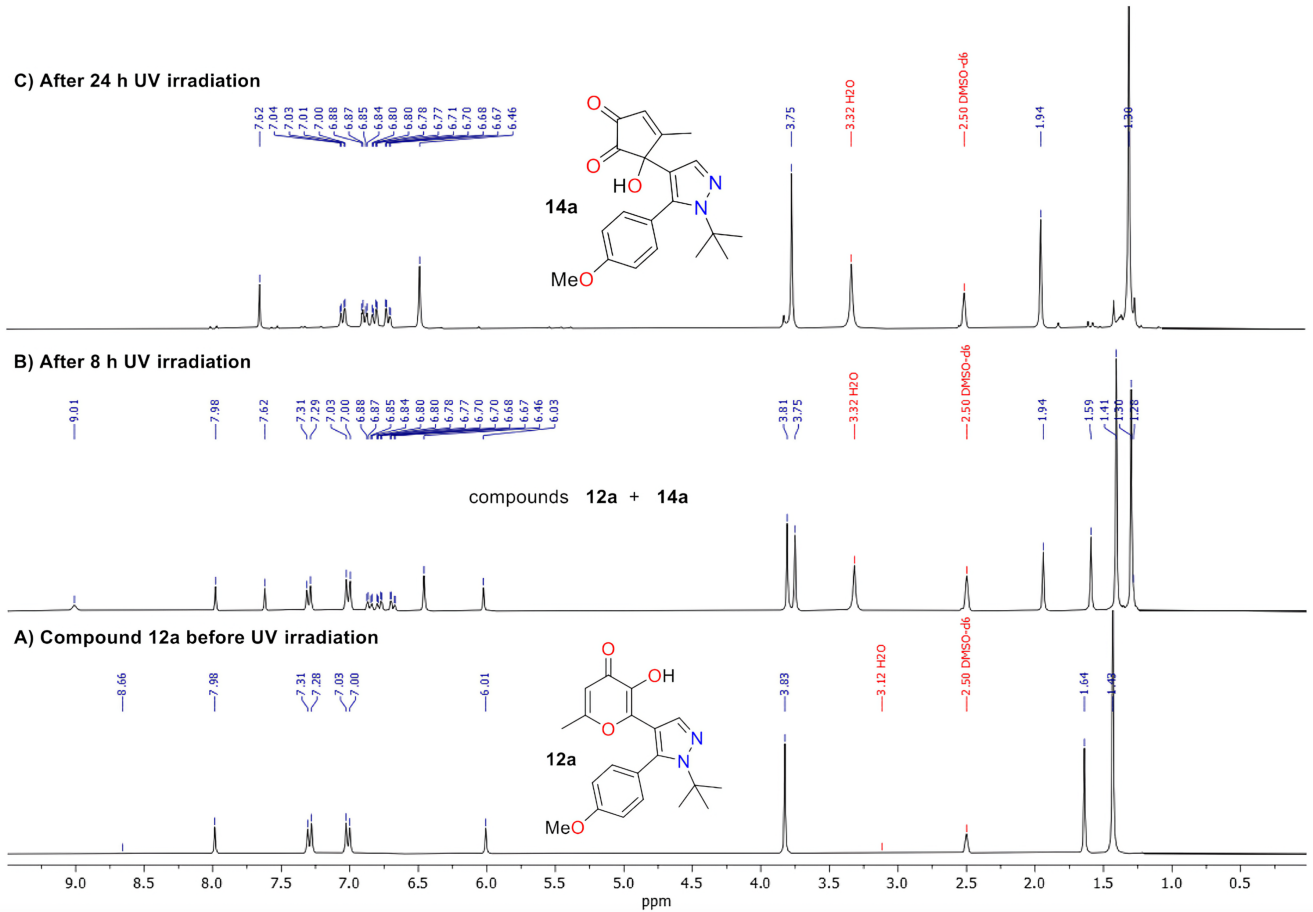
^1^H NMR monitoring of the photoreaction of compound **12a** under UV irradiation (365 nm) in DMSO-*d*_6_ solution.

The phototransformation of compound **12a** was studied using NMR monitoring. UV irradiation was carried out at a wavelength of 365 nm in a solution of DMSO-*d*_6_ (Figure 1). It should be noted that after UV irradiation for 8 h of the studied sample the NMR spectrum contained signals of protons of the product **14a** along with signals of the starting compound **12a** (Figure 1B). Complete conversion to the target compound **14a** was observed after irradiation for 24 h (Figure 1C), confirming the regiospecificity of the studied reaction. It could be concluded that only the ESIPT-promoted contraction of the pyranone ring proceeds under UV irradiation of compound **12a**, while the 6π-electrocyclization products were not detected in the reaction mixture. However, it should be mentioned that the resulting α-hydroxy-1,2-diketone **14a** has a relatively low stability and storing of the NMR sample of compound **14a** in darkness at room temperature for 5 days resulted in complete decomposition.

Despite the labile nature of α-hydroxy-1,2-diketone **14a** we attempted to isolate this product in pure form. It should be noted that in contrast to the methylated derivative **16**, pyrazole **12a** has a low solubility in most organic solvents excluding acetic acid, DMSO and DMF. Initially, UV irradiation of compound **12a** was carried out at a wavelength of 365 nm in a solution of DMSO in common glassware for 24 h. However, subsequent water treatment did not allow the isolation of the target product **14a**. Due to the fact that aqueous medium accelerates the decomposition of target α-hydroxy-1,2-diketone **14a**. Subsequently, we used DMF as a solvent for carrying out the studied photoreaction. Unexpectedly, the starting pyrazole **12a** was isolated unchanged after 24 hours of UV irradiation, indicating that DMF also is not suitable for the considered photoprocess. Probably, the DMF molecule forms an intermolecular hydrogen bond with the hydroxy group of the allomaltol fragment leading to complete suppression of the ESIPT process. Finally, AcOH was used as a solvent for the photochemical synthesis of α-hydroxy-1,2-diketone **14a**. The starting pyrazole **12a** was irradiated for 24 h, the acetic acid was evaporated, and the residue was triturated with Et_2_O affording the α-hydroxy-1,2-diketone **14a** in pure form in 47% yield. It should be noted that the presented conditions are optimal. A further increase in the photoreaction time to 48 h leads to a diminished yield of **14a** (34%). This can be attributed to the low stability of α-hydroxy-1,2-diketone **14a**, which undergoes degradation upon keeping in solution. Also, it was shown that irradiation at a wavelength of 312 nm for 24 h leads to a slight decrease in the yield of compound **14a** (42%). Thus, in this communication we have described for the first time the phototransformation of the 3-hydroxypyran-4-one fragment with the isolation of the resulting substituted α-hydroxy-1,2-diketone in pure form. The structure of the obtained compound **14a** was confirmed using ^1^H, ^13^C NMR spectroscopy as well as 2D NMR experiments (HSQC, HMBC, COSY). Interestingly, in the ^1^H NMR spectrum of the product **14a** the aromatic protons of the 4-methoxyphenyl substituent are represented as four singlets due to the presence of an asymmetric center and the hindered rotation of the aryl substituent relative to the pyrazole ring. The nonequivalence of corresponding carbon atoms of the aromatic ring is also observed in the ^13^C NMR spectrum.

**Scheme 4:**
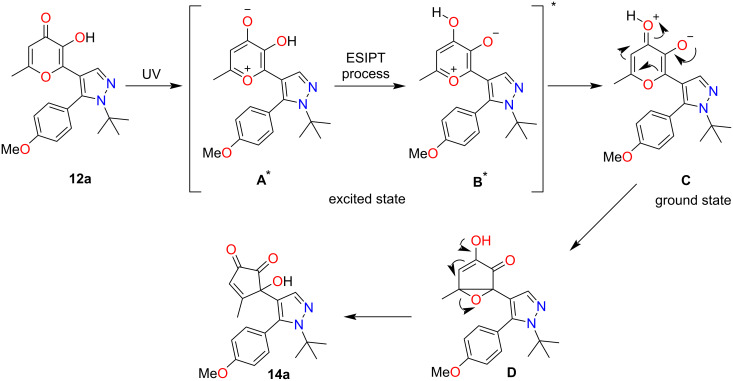
Proposed mechanism for the photoreaction of compound **11a**.

The plausible route for the formation of α-hydroxy-1,2-diketone **14a** is presented in Scheme 4. Based on literature data we suppose that the photoreaction proceeds through an excited state intramolecular proton transfer (ESIPT) [34–36]. At first, compound **12a** under UV irradiation undergoes rapid proton transfer from the excited state A* resulting in the formation of photoisomer B* followed by the conversion to intermediate **C**. Further transformation of zwitter-ion **C** to bicyclic oxirane **D** and its subsequent opening leads to the final α-hydroxy-1,2-diketone **14a**.

Using the process conditions described above for the preparation of α-hydroxy-1,2-diketone **14a**, we attempted to apply the elaborated method to the synthesis of a number of similar compounds with various substituents. Indeed, the proposed approach allowed the synthesis of a variety of substituted α-hydroxy-1,2-diketone **14**, however, according to NMR spectroscopy, the purity of the synthesized compounds **14b**–**l** did not exceed 80%. Attempts to carry out additional purification of these products using recrystallization or chromatographic methods were unsuccessful due to the low stability of the resulting α-hydroxy-1,2-diketone **14** and their partial decomposition during purification. Thus, the purity of the obtained compounds **14** does not allow a complete characterization by various analytical methods.

**Scheme 5:**
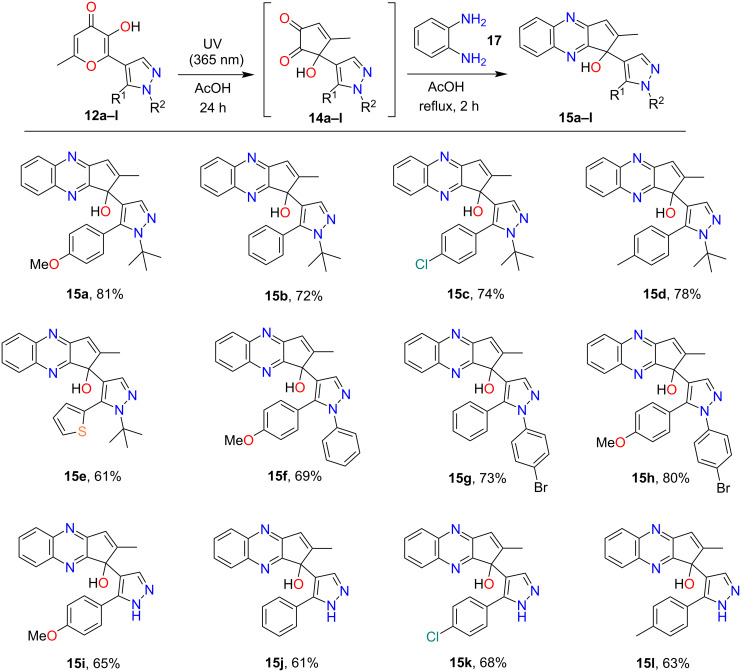
Synthesis of compounds **15a**–**l**. Reaction conditions: 1) **12a**–**l** (0.5 mmol), AcOH (25 mL), UV irradiation, 365 nm, 24 h. 2) **17** (0.6 mmol, 0.07 g), reflux, 2 h.

Based on this fact we made attempts to convert the labile α-hydroxy-1,2-diketone **14** into stable derivatives. A convenient method for the transformation of α-hydroxy-1,2-diketone into the corresponding substituted quinoxalines by reaction with 1,2-phenylenediamine was described in the literature [22]. The application of this protocol allowed the conversion of previously obtained unstable α-hydroxy-1,2-diketone **14a**–**l** into the corresponding quinoxalines **15a**–**l** (Scheme 5). As it was shown earlier, acetic acid is the optimal solvent for the studied compounds and this solvent is also widely used for the condensation of α-diketones with 1,2-phenylenediamine [37–40]. We suggested that it is possible to exclude isolation of unstable α-hydroxy-1,2-diketones **14** and thereby reduce losses at this step. Indeed, UV irradiation (365 nm) of the starting **12a**–**l** pyrazoles and subsequent thermal condensation with 1,2-phenylenediamine (**17**) leads to the corresponding quinoxalines **15a**–**l** in good yields (Scheme 5).

**Figure 2:**
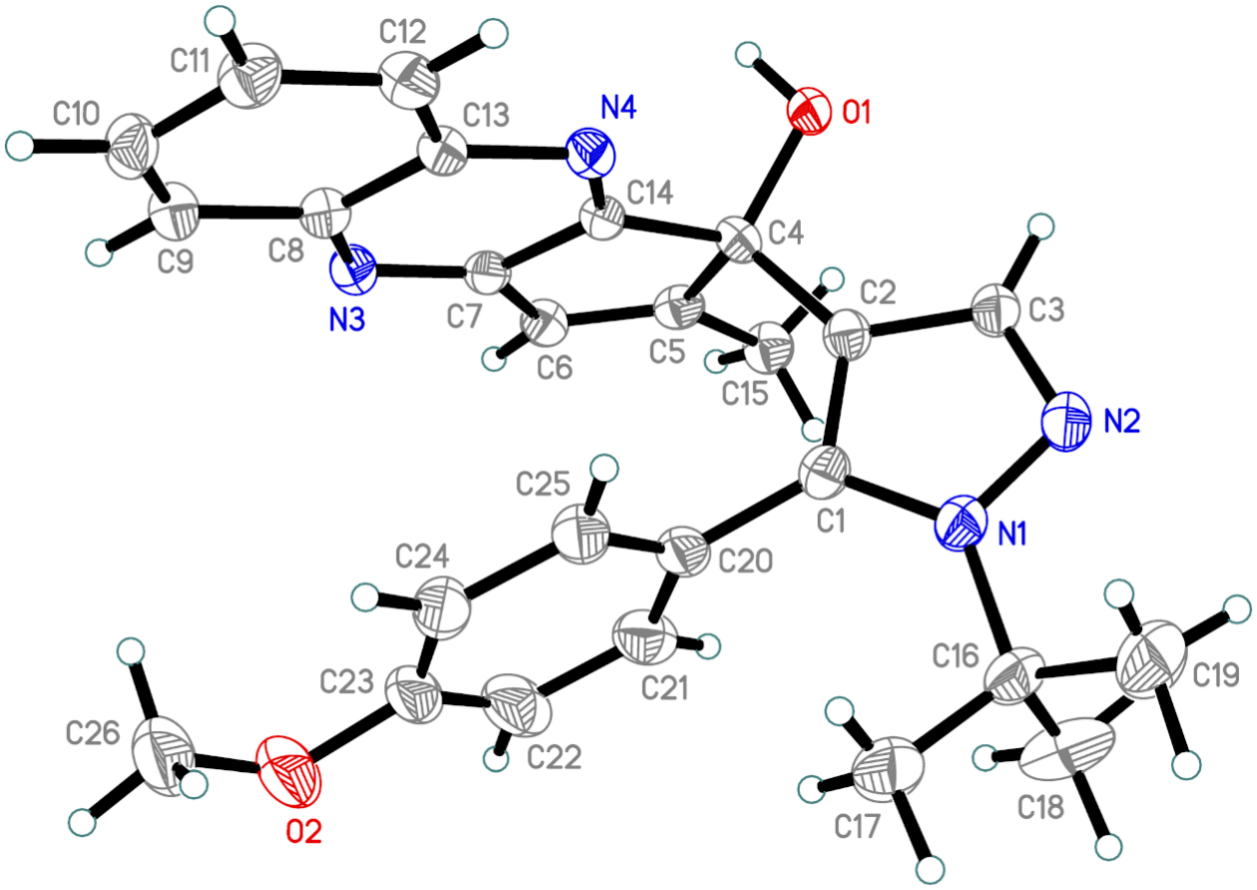
The X-ray crystal structure of compound **15a**.

The structure of **15a** was confirmed by single-crystal X-ray diffraction (Figure 2) as a representative example. It should be noted that X-ray analysis of the final quinoxaline unambiguously proves the conversion of the allomaltol fragment into the corresponding α-hydroxy-1,2-diketone formed in situ as a result of the photoreaction.

**Scheme 6:**
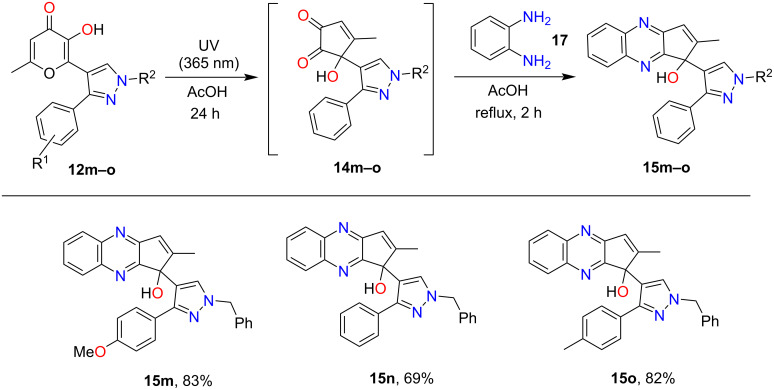
Synthesis of compounds **15m**–**o**. Reaction conditions: 1) **12m**–**o** (0.5 mmol), AcOH (25 mL), UV irradiation, 365 nm, 24 h. 2) **17** (0.6 mmol, 0.07 g), reflux, 2 h.

**Figure 3:**
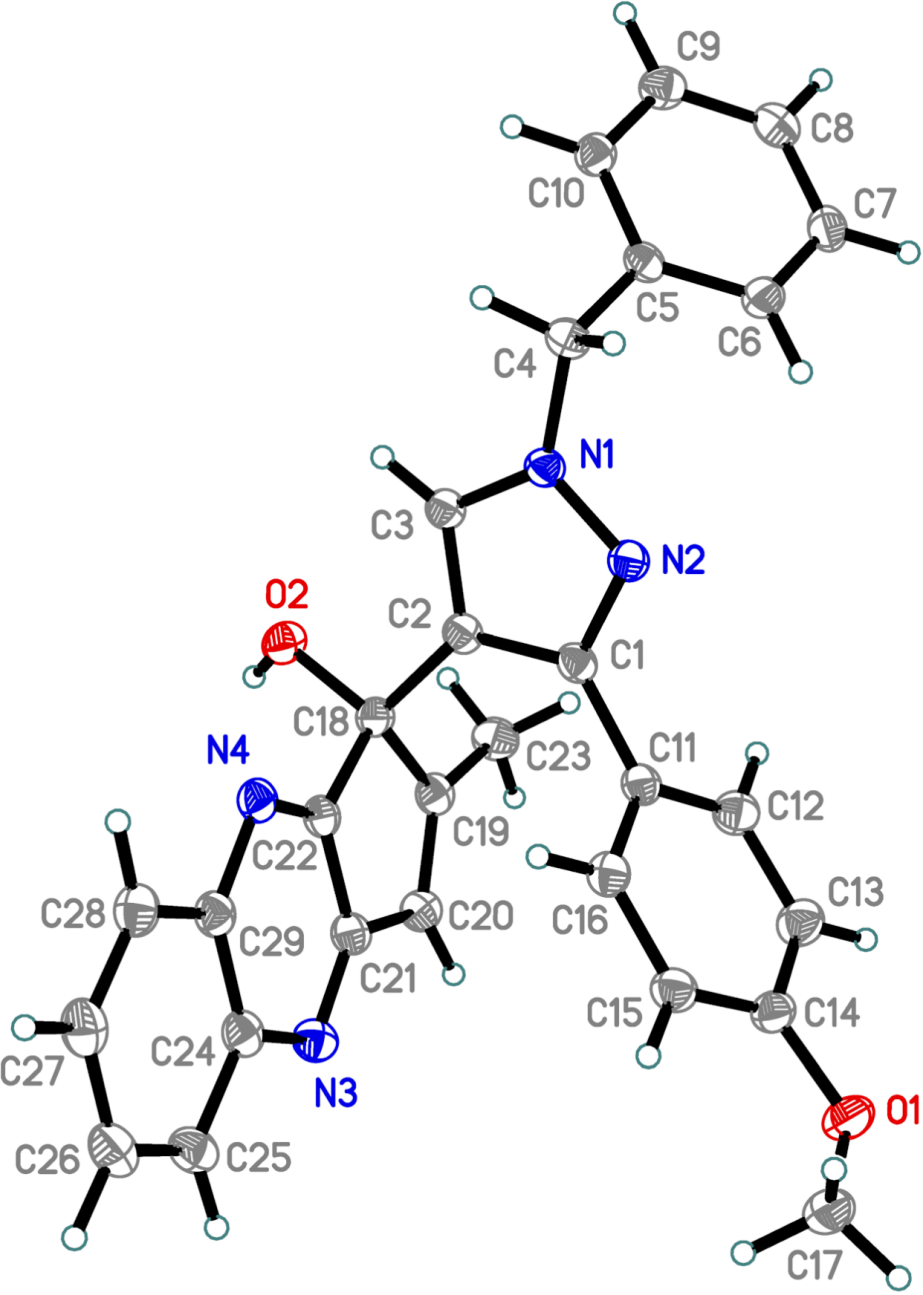
The X-ray crystal structure of compound **15m**.

As shown above, only the phototransformation of the allomaltol fragment occurs under UV irradiation of pyrazoles **12a**–**l**, while the 6π-electrocyclization of the hexatriene system is not observed. Based on this fact we assumed that the structure of the pyrazole fragment should not affect the photoprocess. Therefore, isomeric pyrazoles **12m**–**o** also can be used as starting compounds. Indeed, UV irradiation (365 nm) of these objects in acetic acid and subsequent condensation with 1,2-phenylenediamine led to quinoxalines **15m**–**o** in good yields (Scheme 6). The structure of **15m** was confirmed by single-crystal X-ray diffraction (Figure 3).

**Scheme 7:**
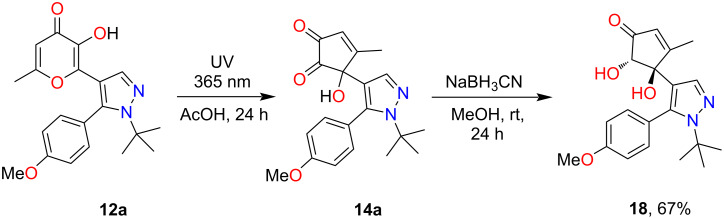
Synthesis of compound **18**.

**Figure 4:**
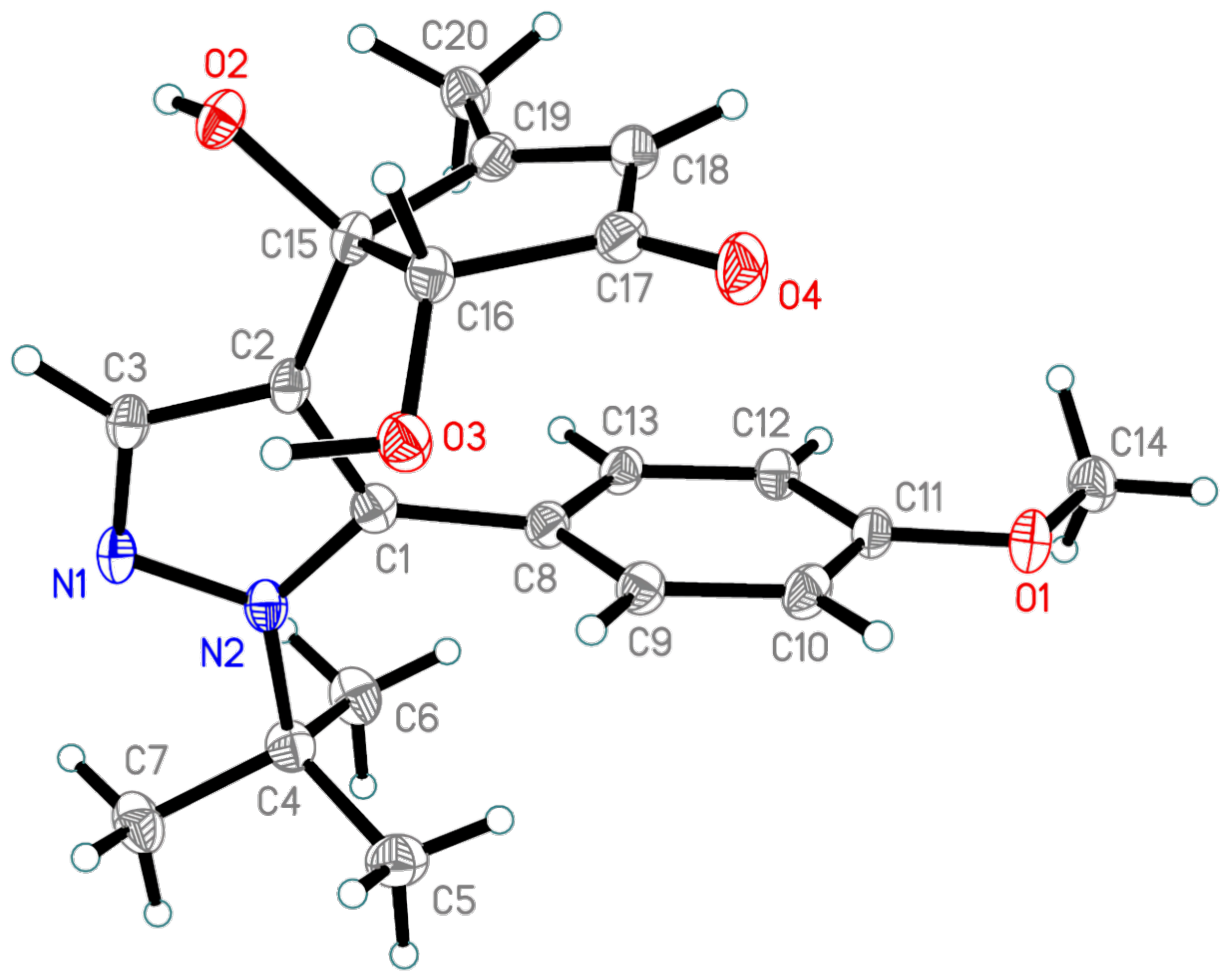
The X-ray crystal structure of compound **18**.

It should be mentioned that other options for trapping of photogenerated unstable α-hydroxy-1,2-diketones are also possible. For example, reduction via NaBH_3_CN can be used as an alternative approach [23]. Initial UV irradiation of pyrazole **12a** and subsequent interaction with NaBH_3_CN results in the formation of the product **18** in 67% yield (Scheme 7). It should be noted that the reaction proceeds regiospecifically with the participation of one carbonyl group. Also worth mentioning is the diastereospecificity of the studied process, which leads to the formation of a *trans*-diastereomer (mixture of *S*/*S*- and *R*/*R*-enantiomers). The structure of vicinal diol **18** was unambiguously established by single-crystal X-ray diffraction (Figure 4).

## Conclusion

In summary, we have studied the photochemical behavior of terarylenes containing pyrazole and allomaltol fragments. It was shown that 6π-electrocyclization of the 1,3,5-hexatriene system does not implement for these objects compounds. At the same time UV-irradiation of the studied terarylenes regiospecifically leads to contraction of the 4-pyranone ring. As a result, labile substituted α-hydroxy-1,2-diketones can be obtained. For the first time the possibility of isolation of the final α-hydroxy-1,2-diketone in pure form was demonstrated in the case of **14a**. We proposed the approach for trapping of unstable α-hydroxy-1,2-diketones in situ employing the condensation with 1,2-phenylenediamine. Based on the aforementioned protocol a method for the synthesis of a wide range of condensed quinoxalines was suggested. It was found that the photogenerated contraction of the 4-pyranone fragment does not depend on the type of pyrazole bridge. The structures of three of the synthesized products were confirmed by X-ray diffraction.

## Supporting Information

File 1 Experimental procedures, characterization data of all products, copies of ^1^H, ^13^C, 2D NMR, HRMS spectra of all new compounds, and X-ray crystallographic data.
