# Supplementary material for: A study of the photochemical behavior of terarylenes containing allomaltol and pyrazole fragments
Source: Beilstein J Org Chem. 2022 May 27;18:588–96. doi: 10.3762/bjoc.18.61 (PMC9152273; doi:10.3762/bjoc.18.61)
Supplement: File 1 — Experimental procedures, characterization data of all products, copies of 1H, 13C, 2D NMR, HRMS spectra of all new compounds, and X-ray crystallographic data. [file Beilstein_J_Org_Chem-18-588-s001.pdf]

BEILSTEIN JOURNAL OF ORGANIC CHEMISTRY

Supporting Information

for

A study of the photochemical behavior of terarylenes containing allomaltol and pyrazole fragments

Constantine V. Milyutin, Andrey N. Komogortsev, Boris V. Lichitsky and Valeriya G. Melekhina

Beilstein J. Org. Chem. **2022,** *18,* 588–596. doi:10.3762/bjoc.18.61

Experimental procedures, characterization data of all products, copies of ^{1}H, ^{13}C, 2D NMR, HRMS spectra of all new compounds, and X-ray crystallographic data

License and Terms: This is a supporting information file under the terms of the Creative Commons Attribution License (https://creativecommons.org/licenses/by/4.0). Please note that the reuse, redistribution and reproduction in particular requires that the author(s) and source are credited and that individual graphics may be subject to special legal provisions.
The license is subject to the *Beilstein Journal of Organic Chemistry* terms and conditions: (https://www.beilstein-journals.org/bjoc/terms)

Table of content

1. General information. S3

2. All synthesized starting compounds S4

3. Characterization data of compounds **19**, **20**, **12** and **16**....S6

4. Experimental procedure and characterization data of compounds **14a**, **15** and **18**....S12

5. Copies of ^{1}H and ^{13}C NMR spectra for compounds **19**, **20**, **12** and **16**. S19

6. Copies of ^{1}H and ^{13}C NMR spectra for compounds **14a**, **15** and **18.** S37

7. Copies of HRMS for all compounds. S54

8. 2D NMR spectra for compound **14a**....S89

9. X-ray crystallographic data and refinement details S93

10. References S125

1. General information.

Unless otherwise stated, all starting chemicals were commercially available and were used as received. Starting compounds **19a**,**b** , **20a**,**b**[1] and **12a,f,h,i**[2] were prepared according to a procedure described previously.

NMR spectra were recorded with Bruker AM 300 (300 MHz) and Bruker DRX 500 (500 MHz) spectrometers in DMSO-d_6. Chemical shifts (ppm) are given relative to solvent signals (DMSO-d_6: 2.50 ppm (^{1}H NMR) and 39.52 ppm (^{13}C NMR)). High-resolution mass spectra (HRMS) were obtained on a Bruker micrOTOF II instrument using electrospray ionization (ESI). The melting points were determined on a Kofler hot stage.

UV-irradiation was carried out with a Vilber Lourmat VL-6.LM lamp (365 nm). Photochemical reactions were performed in common borosilicate glassware at 27 °C. The distance from the light source to the irradiation vessel was 4 cm.

2. All synthesized starting compounds

The synthesis of compounds **19** and **20**:

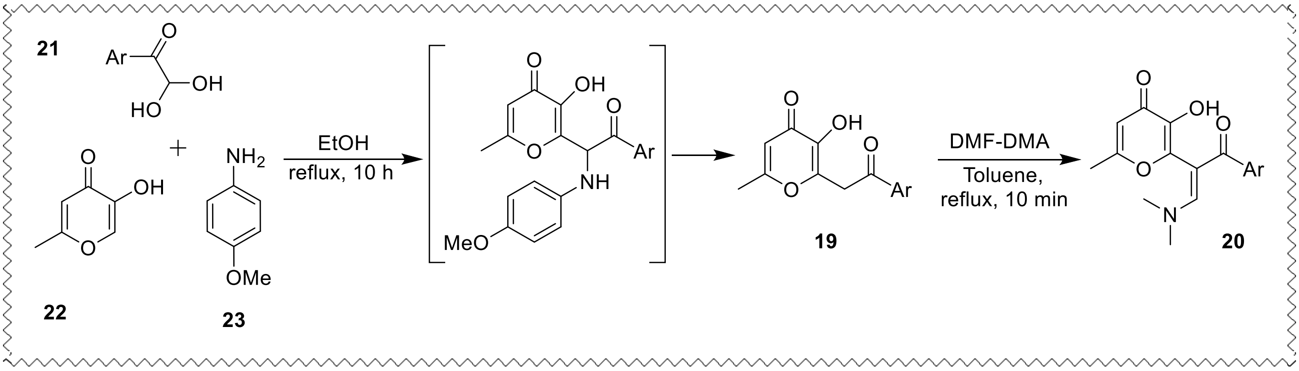

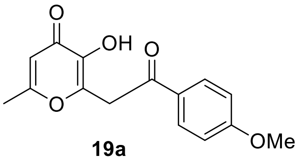
19a

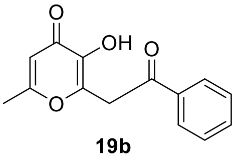
19b

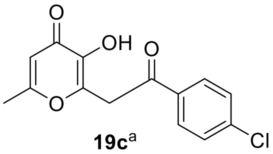
19c[a]

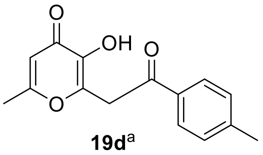
19d[a]

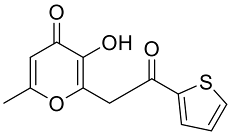
19e[a]

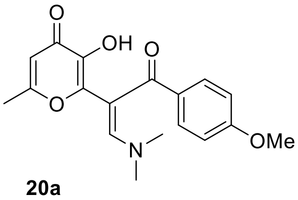
20a

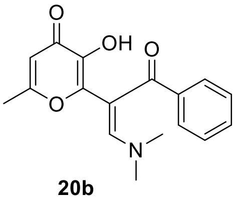
20b

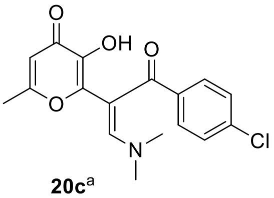
20c[a]

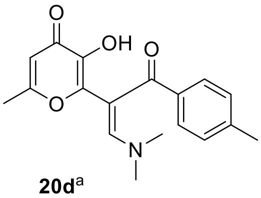
20d[a]

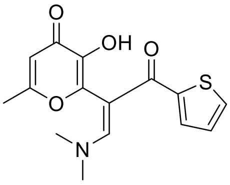
20e[a]

[a] New compounds

The synthesis of compounds **12**:

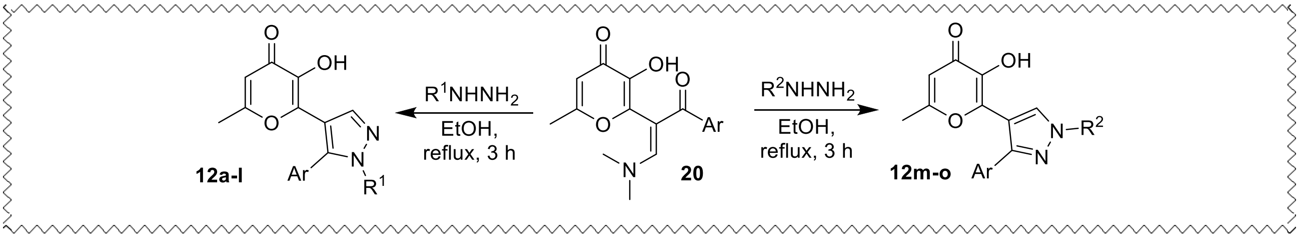

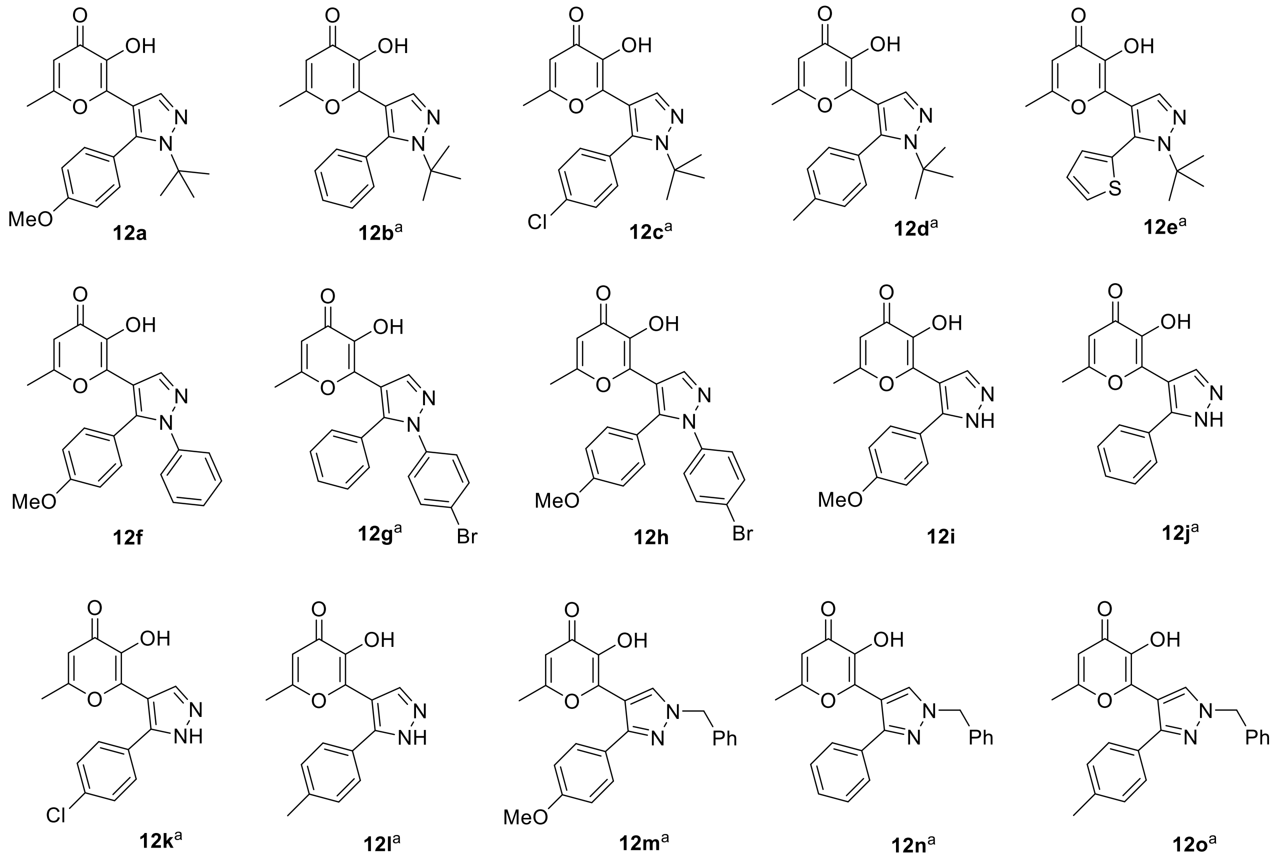

[a] New compounds

The synthesis of compound **16**:

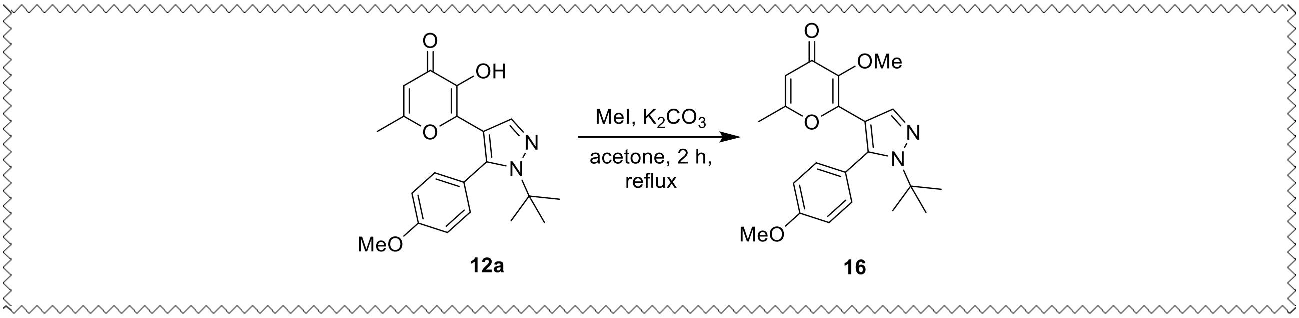

3. Characterization data of compounds **19**, **20**, **12** and **16**.

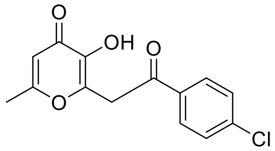

*2-(2-(4-Chlorophenyl)-2-oxoethyl)-3-hydroxy-6-methyl-4H-pyran-4-one (**19c**)*

Yellow powder; yield 31% (0.86 g); mp 201-203 °C. ^{1}H NMR (300 MHz, DMSO-*d*$_6$) δ 9.01 (br. s, 1H), 8.04 (d, *J* = 8.5 Hz, 2H), 7.62 (d, *J* = 8.4 Hz, 2H), 6.23 (s, 1H), 4.43 (s, 2H), 2.22 (s, 3H). ^{13}C NMR (75 MHz, DMSO-*d*$_6$) δ 193.53, 173.37, 164.59, 145.49, 142.88, 138.73, 134.46, 130.18, 128.97, 111.47, 38.63, 19.20. HRMS (ESI-TOF) *m/z*: [M+H]$^+$ Calcld for $C_{14}H_{11}ClO_4$ 279.0419; Found 279.0424.

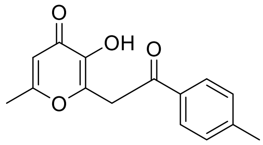

*3-Hydroxy-6-methyl-2-(2-oxo-2-(p-tolyl)ethyl)-4H-pyran-4-one (**19d**)*

Yellow powder; yield 33% (0.85 g); mp 180-182 °C. ^{1}H NMR (300 MHz, DMSO-*d*$_6$) δ 8.98 (br.s, 1H), 7.93 (d, *J* = 8.2 Hz, 2H), 7.35 (d, *J* = 7.9 Hz, 2H), 6.23 (s, 1H), 4.38 (s, 2H), 2.38 (s, 3H), 2.21 (s, 3H). ^{13}C NMR (75 MHz, DMSO-*d*$_6$) δ 193.99, 173.47, 164.65, 145.99, 144.37, 142.85, 133.38, 129.47, 128.43, 111.49, 38.54, 21.25, 19.26. HRMS (ESI-TOF) *m/z*: [M+H]$^+$ Calcld for $C_{15}H_{14}O_4$ 259.0965; Found 259.0964.

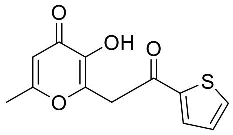

*3-Hydroxy-6-methyl-2-(2-oxo-2-(thiophen-2-yl)ethyl)-4H-pyran-4-one (**19e**)*

Yellow powder; yield 27% (0.68 g); mp 122-124 °C. ^{1}H NMR (300 MHz, DMSO-*d*$_6$) δ 9.04 (br.s, 1H), 8.09 (d, *J* = 3.9 Hz, 1H), 8.06 (d, *J* = 4.9 Hz, 1H), 7.33 – 7.22 (m, 1H), 6.23 (s, 1H), 4.37 (s, 2H), 2.22 (s, 3H). ^{13}C NMR (75 MHz, DMSO-*d*$_6$) δ 187.37, 173.46, 164.66, 145.33, 142.92, 142.71, 135.81, 134.39, 128.96, 111.52, 38.82, 19.25. HRMS (ESI-TOF) *m/z*: [M+H]$^+$ Calcld for $C_{12}H_{10}O_4S$ 251.0373; Found 251.0386.

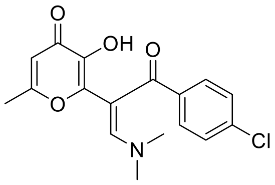

*2-(3-(4-Chlorophenyl)-1-(dimethylamino)-3-oxoprop-1-en-2-yl)-3-hydroxy-6-methyl-4H-pyran-4-one (**20c**)*

Yellow powder; yield 70% (0.23 g); mp 189-191 °C. ^{1}H NMR (300 MHz, DMSO-*d*$_6$) δ 8.61 (br.s, 1H), 7.52 (s, 1H), 7.43 – 7.31 (m, 4H), 6.16 (s, 1H), 3.16 (s, 3H), 2.72 (s, 3H), 2.16 (s, 3H). ^{13}C NMR (75 MHz, DMSO-*d*$_6$) δ

173.62, 164.46, 154.98, 146.43, 143.30, 140.23, 134.66, 129.49, 128.07, 111.40, 98.51, 19.72. HRMS (ESI-TOF) *m/z*: $[M+H]^+$ Calcld for $C_{17}H_{16}NO_4$ 332.0684; Found 332.0678.

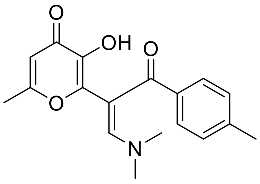

*2-(1-(Dimethylamino)-3-oxo-3-(p-tolyl)prop-1-en-2-yl)-3-hydroxy-6-methyl-4H-pyran-4-one (**20d**)*

Yellow powder; yield 82% (0.26 g); mp 174-176 °C. ^{1}H NMR (300 MHz, DMSO-d_6) δ 8.41 (br.s, 1H), 7.46 (s, 1H), 7.29 (d, *J* = 7.7 Hz, 2H), 7.13 (d, *J* = 7.8 Hz, 2H), 6.15 (s, 1H), 3.73 – 3.10 (m, 6H), 2.29 (s, 3H), 2.15 (s, 3H). ^{13}C NMR (75 MHz, DMSO-d_6) δ 190.65, 173.45, 164.22, 154.51, 146.41, 142.99, 139.64, 138.41, 129.52, 128.32, 127.66, 111.11, 98.68, 20.96, 19.49. HRMS (ESI-TOF) *m/z*: $[M-H]^+$ Calcld for $C_{18}H_{19}NO_4$ 312.1230; Found 312.1228.

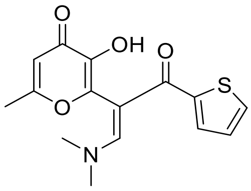

*2-(1-(Dimethylamino)-3-oxo-3-(thiophen-2-yl)prop-1-en-2-yl)-3-hydroxy-6-methyl-4H-pyran-4-one (**20e**)*

Brown powder; yield 77% (0.23 g); mp 167-169 °C. ^{1}H NMR (300 MHz, DMSO-d_6) δ 8.56 (br.s, 1H), 7.77 – 7.56 (m, 2H), 7.16 (s, 1H), 7.03 (s, 1H), 6.27 (s, 1H), 3.02 (s, 6H), 2.23 (s, 3H). ^{13}C NMR (75 MHz, DMSO-d_6) δ 180.49, 173.51, 164.61, 154.33, 146.29, 145.12, 143.81, 131.09, 129.55, 127.47, 111.54, 97.49, 19.60. HRMS (ESI-TOF) *m/z*: $[M+H]^+$ Calcld for $C_{15}H_{15}NO_4S$ 304.0638; Found 304.0632.

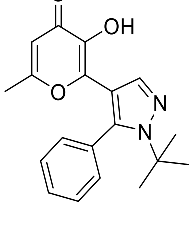

*2-(1-(tert-Butyl)-5-phenyl-1H-pyrazol-4-yl)-3-hydroxy-6-methyl-4H-pyran-4-one (**12b**)*

White powder; yield 74% (0.72 g); mp 246-248 °C. ^{1}H NMR (300 MHz, DMSO-d_6) δ 8.68 (br.s, 1H), 8.01 (s, 1H), 7.53 – 7.42 (m, 3H), 7.43 – 7.33 (m, 2H), 5.99 (s, 1H), 1.55 (s, 3H), 1.48 – 1.38 (m, 9H). ^{13}C NMR (75 MHz, DMSO-d_6) δ 173.07, 162.84, 140.23, 136.50, 133.07, 130.62, 128.62, 127.70, 113.09, 110.19, 61.94, 30.71, 18.05. HRMS (ESI-TOF) *m/z*: $[M+H]^+$ Calcld for $C_{19}H_{20}N_2O_3$ 325.1547; Found 325.1546.

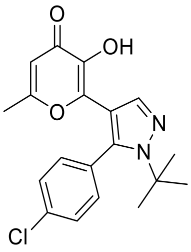

*2-(1-(tert-Butyl)-5-(4-chlorophenyl)-1H-pyrazol-4-yl)-3-hydroxy-6-methyl-4H-pyran-4-one (**12c**)*

White powder; yield 70% (0.75 g); mp 263-265 °C. ^{1}H NMR (300 MHz, DMSO-d_6) δ 8.79 (br.s, 1H), 8.02 (s, 1H), 7.52 (d, *J* = 8.5 Hz, 2H), 7.44 (d, *J* = 8.4 Hz, 2H), 6.02 (s, 1H), 1.62 (s, 3H), 1.48 – 1.36 (m, 9H). ^{13}C NMR (75 MHz, DMSO-d_6) δ 173.04, 162.79, 140.31, 138.82, 136.53, 133.60, 132.50, 131.95, 129.29, 127.79, 113.30, 110.28, 62.01, 30.70, 17.98. HRMS (ESI-TOF) *m/z*: $[M+H]^+$ Calcld for $C_{19}H_{19}ClN_2O_3$ 359.1157; Found 359.1148.

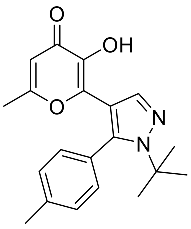

*2-(1-(tert-Butyl)-5-(p-tolyl)-1H-pyrazol-4-yl)-3-hydroxy-6-methyl-4H-pyran-4-one (**12d**)*

White powder; yield 68% (0.69 g); mp 207-209 °C. ^{1}H NMR (300 MHz, DMSO-d_6) δ 9.01 (br.s, 1H), 7.99 (s, 1H), 7.33 – 7.19 (m, 4H), 6.00 (s, 1H), 2.37 (s, 3H), 1.53 (s, 3H), 1.39 (s, 9H). ^{13}C NMR (126 MHz, DMSO-d_6) δ 172.71, 162.85, 142.16, 140.24, 139.85, 137.77, 136.26, 130.12, 129.62, 127.79, 112.72, 109.64, 61.54, 30.37, 20.33, 17.63. HRMS (ESI-TOF) *m/z*: $[M+H]^+$ Calcld for $C_{20}H_{22}N_2O_3$ 339.1703; Found 339.1700.

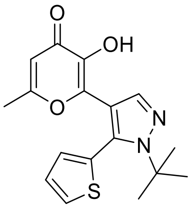

*2-(1-(tert-Butyl)-5-(thiophen-2-yl)-1H-pyrazol-4-yl)-3-hydroxy-6-methyl-4H-pyran-4-one (**12e**)*

Grey powder; yield 56% (0.55 g); mp 237-239 °C. ^{1}H NMR (300 MHz, DMSO-d_6) δ 9.17 (br.s, 1H), 8.01 (s, 1H), 7.85 – 7.71 (m, 1H), 7.25 (s, 1H), 7.20 – 7.12 (m, 1H), 6.08 (s, 1H), 1.71 (s, 3H), 1.58 – 1.34 (m, 9H). ^{13}C NMR (75 MHz, DMSO-d_6) δ 173.13, 163.12, 141.77, 140.77, 136.60, 132.54, 131.69, 131.11, 128.79, 126.83, 115.21, 110.37, 62.29, 30.38, 18.39. HRMS (ESI-TOF) *m/z*: $[M+H]^+$ Calcld for $C_{17}H_{18}N_2O_3S$ 331.1111; Found 331.1114.

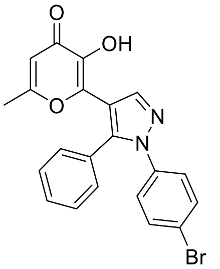

*2-(1-(4-Bromophenyl)-5-phenyl-1H-pyrazol-4-yl)-3-hydroxy-6-methyl-4H-pyran-4-one (**12g**)*

White powder; yield 75% (0.95 g); mp 245-247 °C. ^{1}H NMR (300 MHz, DMSO-d_6) δ 8.28 (s, 1H), 7.54 (d, *J* = 8.4 Hz, 2H), 7.47 – 7.35 (m, 3H),

7.35 – 7.27 (m, 2H), 7.22 (d, *J* = 8.4 Hz, 2H), 6.11 (s, 1H), 1.73 (s, 3H). ^{13}C NMR (75 MHz, DMSO-d_6) δ 173.29, 163.44, 141.45, 140.99, 140.54, 140.02, 138.29, 131.87, 130.19, 129.77, 128.93, 128.18, 127.30, 120.81, 112.99, 110.62, 18.35. HRMS (ESI-TOF) *m/z*: [M+H]$^+$ Calcld for $C_{21}H_{15}BrN_2O_3$ 423.0339; Found 423.0331.

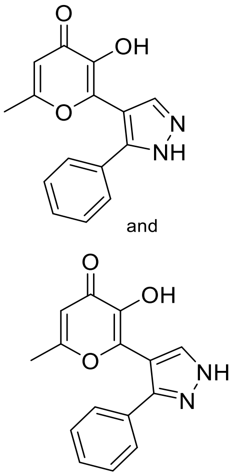

*A mixture of 3-hydroxy-6-methyl-2-(5-phenyl-1H-pyrazol-4-yl)-4H-pyran-4-one and 3-hydroxy-6-methyl-2-(3-phenyl-1H-pyrazol-4-yl)-4H-pyran-4-one isomers (**12j**)*

Yellow powder; yield 59% (0.47 g); mp 233-240 °C. ^{1}H NMR (300 MHz, DMSO-d_6) δ 13.64 – 13.31 (m, 1H), 9.14 (br.s, 1H), 8.23 (br.s, 0.5 H), 8.03 (br.s, 0.5 H) 7.56 – 7.28 (m, 5H), 6.21 (s, 1H), 1.92 (s, 3H). ^{13}C NMR (75 MHz, DMSO-d_6) δ 173.40, 163.71, 142.68, 140.66, 140.08, 134.10, 129.63, 128.70, 128.30, 127.92, 127.67, 110.83, 108.90, 108.51, 18.76. HRMS (ESI-TOF) *m/z*: [M+H]$^+$ Calcld for $C_{15}H_{12}N_2O_3$ 269.0921; Found 269.0917.

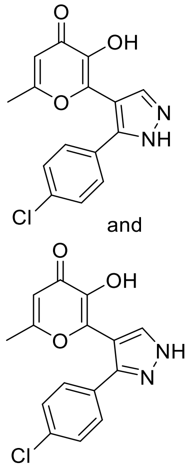

*A mixture of 2-(5-(4-chlorophenyl)-1H-pyrazol-4-yl)-3-hydroxy-6-methyl-4H-pyran-4-one and 2-(3-(4-chlorophenyl)-1H-pyrazol-4-yl)-3-hydroxy-6-methyl-4H-pyran-4- one isomers (**12k**)*

Yellow powder; yield 61% (0.55 g); mp 285-287 °C. ^{1}H NMR (300 MHz, DMSO-d_6) δ 13.48 (br.s, 1H), 9.13 (br.s, 1H), 8.18 (br.s, 1H), 7.59 – 7.38 (m, 4H), 6.22 (s, 1H), 1.98 (s, 3H). ^{13}C NMR (75 MHz, DMSO-d_6) δ 173.35, 163.77, 142.33, 140.64, 131.15, 130.00, 128.02, 110.82, 18.79. HRMS (ESI-TOF) *m/z*: [M+H]$^+$ Calcld for $C_{15}H_{11}ClN_2O_3$ 303.0531; Found 303.0530.

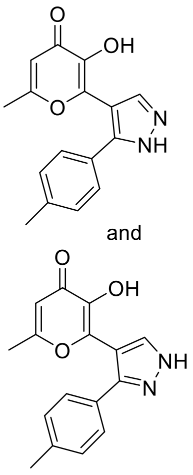

*A mixture of 3-hydroxy-6-methyl-2-(5-(p-tolyl)-1H-pyrazol-4-yl)-4H-pyran-4-one and 3-hydroxy-6-methyl-2-(3-(p-tolyl)-1H-pyrazol-4-yl)-4H-pyran-4-one isomers (**12l**)*

Pale yellow powder; yield 63% (0.53 g); mp 253-255 °C. ^{1}H NMR (300 MHz, DMSO-d_6) δ 13.37 (br.s, 1H), 9.04 (br.s, 1H), 8.09 (br.s, 1H), 7.38 (d, *J* = 7.7 Hz, 2H), 7.23 (d, *J* = 7.7 Hz, 2H), 6.21 (s, 1H), 2.33 (s, 3H), 1.96 (s, 3H). ^{13}C NMR (75 MHz, DMSO-d_6) δ 173.42, 163.79, 148.29, 142.78, 140.72, 140.13, 138.30, 137.83, 136.91, 131.08, 128.86, 128.53, 128.16, 126.44, 110.82, 108.67, 108.34, 20.90, 18.86. HRMS (ESI-TOF) *m/z*: [M+H]$^+$ Calcld for $C_{16}H_{14}N_2O_3$ 283.1077; Found 283.1078.

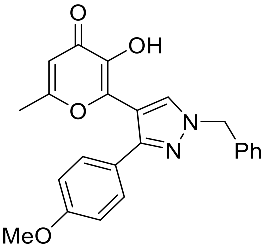

*2-(1-Benzyl-3-(4-methoxyphenyl)-1H-pyrazol-4-yl)-3-hydroxy-6-methyl-4H-pyran-4-one (**12m**)*

White powder; yield 68% (0.79 g); mp 194-196 °C. ^{1}H NMR (300 MHz, DMSO-d_6) δ 9.19 (br.s, 1H), 8.34 (s, 1H), 7.47 – 7.24 (m, 7H), 6.95 (d, *J* = 8.7 Hz, 2H), 6.21 (s, 1H), 5.43 (s, 2H), 3.76 (s, 3H), 1.95 (s, 3H). ^{13}C NMR (75 MHz, DMSO-d_6) δ 173.30, 163.66, 159.01, 148.91, 142.30, 140.52, 137.05, 132.52, 129.52, 128.65, 127.82, 125.96, 113.32, 110.87, 108.88, 55.02, 55.14, 18.87. HRMS (ESI-TOF) *m/z*: [M+H]$^+$ Calcld for $C_{23}H_{20}N_2O_4$ 389.1496; Found 389.1503.

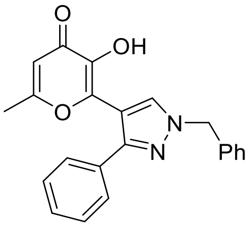

*2-(1-Benzyl-3-phenyl-1H-pyrazol-4-yl)-3-hydroxy-6-methyl-4H-pyran-4-one (**12n**)*

Pale brown powder; yield 63% (0.68 g); mp 156-158 °C. ^{1}H NMR (300 MHz, DMSO-d_6) δ 9.24 (br.s, 1H), 8.39 (s, 1H), 7.52 – 7.44 (m, 2H), 7.41 – 7.25 (m, 8H), 6.21 (s, 1H), 5.46 (s, 2H), 1.89 (s, 3H). ^{13}C NMR (75 MHz, DMSO-d_6) δ 173.26, 163.54, 149.08, 142.08, 140.48, 136.97, 133.61, 132.52, 128.63, 128.30, 127.82, 127.79, 110.84, 109.23, 55.04, 18.67. HRMS (ESI-TOF) *m/z*: [M+H]$^+$ Calcld for $C_{22}H_{18}N_2O_3$ 359.1390; Found 359.1384.

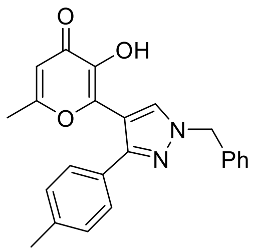

*2-(1-Benzyl-3-(p-tolyl)-1H-pyrazol-4-yl)-3-hydroxy-6-methyl-4H-pyran-4-one (**12o**)*

White powder; yield 72% (0.8 g); mp 211-213 °C. ^{1}H NMR (300 MHz, DMSO-d_6) δ 9.18 (br.s, 1H), 8.35 (s, 1H), 7.43 – 7.26 (m, 7H), 7.19 (d, J = 7.9 Hz, 2H), 6.21 (s, 1H), 5.45 (s, 2H), 2.32 (s, 3H), 1.93 (s, 3H). ^{13}C NMR (75 MHz, DMSO-d_6) δ 173.26, 163.61, 149.05, 142.16, 140.53, 137.01, 132.52, 130.69, 128.62, 128.40, 128.10, 127.79, 110.84, 109.02, 55.00, 20.83, 18.77. HRMS (ESI-TOF) *m/z*: [M+H]$^+$ Calcld for $C_{23}H_{20}N_2O_3$ 373.1547; Found 373.1542.

*Experimental procedure and characterization data of compound **16**.*

A mixture of compound **12a** (1 mmol, 0.35 g), K_2CO_3 (2 mmol, 0.27 g) and MeI (3 mmol, 0.43 g) in acetone (10 ml) was refluxed for 2 h. Then the solvent was removed under reduced pressure, H_2O (20 ml) was added, and the mixture was left overnight. The resulting precipitate was filtered off and washed H_2O (3 x 10 ml) to give the corresponding product **16**.

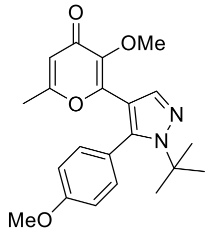

*2-(1-(tert-Butyl)-5-(4-methoxyphenyl)-1H-pyrazol-4-yl)-3-methoxy-6-methyl-4H-pyran-4-one (**16**)*

White powder; yield 94% (0.35 g); mp 149-151 °C. ^{1}H NMR (300 MHz, DMSO-d_6) δ 7.96 (s, 1H), 7.30 (d, J = 8.4 Hz, 2H), 7.02 (d, J = 8.1 Hz, 2H), 6.01 (s, 1H), 3.81 (s, 3H), 3.75 (s, 3H), 1.58 (s, 3H), 1.41 (s, 9H). ^{13}C NMR (75 MHz, DMSO-d_6) δ 174.39, 163.16, 159.59, 151.33, 142.08, 141.44, 136.70, 131.76, 124.34, 113.32, 113.22, 112.38, 61.98, 58.92, 55.25, 30.67, 17.99. HRMS (ESI-TOF) *m/z*: [M+H]$^+$ Calcld for $C_{21}H_{24}N_2O_4$ 369.1809; Found 369.1809.

4. Experimental procedure and characterization data of compounds **14a**, **15** and **18**.

*Experimental procedure for the synthesis of photoproduct **14a***

A solution of compound **12a** (0.5 mmol, 0.18 g) in AcOH (25 ml) was irradiated in common glassware with a Vilber Lourmat VL-6.LM (365 nm, 6 W) for 24 h. Then the solvent was removed under reduced pressure and the residue was triturated with Et_2O (10 ml) to give the corresponding photoproduct **14a**.

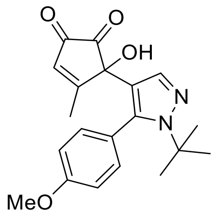

*5-(1-(tert-Butyl)-5-(4-methoxyphenyl)-1H-pyrazol-4-yl)-5-hydroxy-4-methylcyclopent-3-ene-1,2-dione (**14a**)*

Orange powder; yield 47% (0.08 g); mp 161-163 °C. ^{1}H NMR (300 MHz, DMSO-d_6) δ 7.62 (s, 1H), 7.02 (dd, *J* = 8.5, 2.2 Hz, 1H), 6.86 (dd, *J* = 8.5, 2.7 Hz, 1H), 6.78 (dd, *J* = 8.4, 2.7 Hz, 1H), 6.68 (dd, *J* = 8.4, 2.2 Hz, 1H), 6.52 – 6.41 (m, 2H), 3.75 (s, 3H), 1.94 (s, 3H), 1.30 (s, 9H). ^{13}C NMR (75 MHz, DMSO-d_6) δ 201.02, 186.03, 176.73, 159.64, 137.56, 135.47, 135.23, 132.70, 132.25, 122.48, 119.40, 113.47, 72.41, 61.14, 55.21, 30.55, 14.57. HRMS (ESI-TOF) *m/z*: $[M+H]^+$ Calcld for $C_{20}H_{22}N_2O_4$ 355.1652; Found 355.1655.

*Experimental procedure for the synthesis of compounds **15.***

A solution of compounds **12** (0.5 mmol) in AcOH (25 ml) was irradiated in common glassware with a Vilber Lourmat VL-6.LM (365 nm, 6 W) for 24 h. 1,2-Phenylendiamine (0.6 mmol, 0.07 g) was added to reaction mixture and obtained solution was refluxed for 2 h. Then the solvent was removed under reduced pressure and the residue was recrystallized from EtOH (5 ml). The resulting precipitate was filtered off and washed EtOH (3 x 5 ml) to give the corresponding product **15**.

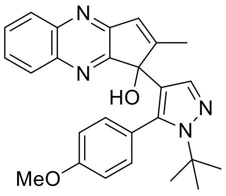

*1-(1-(tert-Butyl)-5-(4-methoxyphenyl)-1H-pyrazol-4-yl)-2-methyl-1H-cyclopenta[b]quinoxalin-1-ol (**15a**)*

White powder ; yield 81% (0.17 g); mp 243-245 °C. ^{1}H NMR (300 MHz, DMSO-d_6) δ 7.98 – 7.90 (m, 1H), 7.82 – 7.75 (m, 1H), 7.73 (s, 1H), 7.71

– 7.59 (m, 2H), 6.86 (dd, J = 8.5, 2.2 Hz, 1H), 6.69 (dd, J = 8.5, 2.7 Hz, 1H), 6.21 (s, 1H), 6.08 (s, 1H), 5.86 (dd, J = 8.5, 2.2 Hz, 1H), 5.70 (dd, J = 8.5, 2.7 Hz, 1H), 3.52 (s, 3H), 1.88 (s, 3H), 1.25 (s, 9H). ^{13}C NMR (75 MHz, DMSO-d_6) δ 164.62, 162.14, 158.69, 157.04, 141.52, 139.10, 136.85, 136.10, 131.90, 131.38, 128.89, 128.79, 127.99, 127.79, 125.86, 122.67, 120.24, 113.35, 111.02, 78.12, 60.76, 54.84, 30.57, 12.99. HRMS (ESI-TOF) *m/z*: $[M+H]^+$ Calcld for $C_{26}H_{26}N_4O_2$ 427.2129; Found 427.2128.

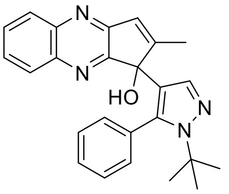

*1-(1-(tert-Butyl)-5-phenyl-1H-pyrazol-4-yl)-2-methyl-1H-cyclopenta[b]quinoxalin-1-ol (**15b**)*

Pale yellow powder; yield 72% (0.14 g); mp 255-257 °C. ^{1}H NMR (300 MHz, DMSO-d_6) δ 7.99 – 7.90 (m, 1H), 7.80 (s, 1H), 7.79 – 7.72 (m, 1H), 7.69 – 7.59 (m, 2H), 7.22 – 7.12 (m, 1H), 7.04 – 6.95 (m, 2H), 6.28 – 6.21 (m, 1H), 6.14 (d, J = 1.7 Hz, 1H), 6.10 (s, 1H), 5.95 (d, J = 8.0 Hz, 1H), 1.89 (s, 3H), 1.24 (s, 9H). ^{13}C NMR (75 MHz, DMSO-d_6) δ 164.54, 162.10, 157.11, 141.53, 139.11, 137.15, 136.31, 131.09, 130.56, 130.40, 129.11, 128.89, 128.15, 127.94, 127.45, 126.09, 119.90, 78.14, 61.08, 30.64, 13.13. HRMS (ESI-TOF) *m/z*: $[M+H]^+$ Calcld for $C_{25}H_{24}N_4O$ 397.2023; Found 397.2013.

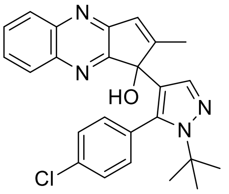

*1-(1-(tert-Butyl)-5-(4-chlorophenyl)-1H-pyrazol-4-yl)-2-methyl-1H-cyclopenta[b]quinoxalin-1-ol (**15c**)*

Pale yellow powder; yield 74% (0.16 g); mp 228-230 °C. ^{1}H NMR (300 MHz, DMSO-d_6) δ 7.99 – 7.88 (m, 1H), 7.81 (s, 1H), 7.77 (d, J = 6.0 Hz, 1H), 7.71 – 7.59 (m, 2H), 7.22 (d, J = 8.6 Hz, 1H), 7.03 (d, J = 7.8 Hz, 1H), 6.26 (s, 1H), 6.26 – 6.13 (m, 2H), 6.03 (d, J = 7.6 Hz, 1H), 1.91 (s, 3H), 1.24 (s, 9H). ^{13}C NMR (75 MHz, DMSO-d_6) δ 164.29, 162.38, 156.99, 141.58, 138.99, 136.34, 135.73, 133.14, 132.53, 132.09, 129.88, 129.14, 128.85, 128.06, 128.01, 127.46, 126.19, 125.97, 120.08, 78.06, 61.14, 30.62, 13.04. HRMS (ESI-TOF) *m/z*: $[M+H]^+$ Calcld for $C_{25}H_{23}ClN_4O$ 431.1633; Found 431.1634.

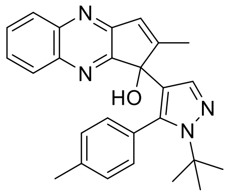

*1-(1-(tert-Butyl)-5-(p-tolyl)-1H-pyrazol-4-yl)-2-methyl-1H-cyclopenta[b]quinoxalin-1-ol (**15d**)*

White powder; yield 78% (0.16 g); mp 243-245 °C. ^{1}H NMR (300 MHz, DMSO-d_6) δ 7.91 (s, 1H), 7.84 – 7.72 (m, 2H), 7.70 – 7.60 (m, 2H), 6.95 (d, *J* = 7.7 Hz, 1H), 6.83 (d, *J* = 7.9 Hz, 1H), 6.19 (s, 1H), 6.09 (s, 1H), 5.97 (d, *J* = 7.8 Hz, 1H), 5.86 (d, *J* = 7.9 Hz, 1H), 2.04 (s, 3H), 1.89 (s, 3H), 1.24 (s, 9H). ^{13}C NMR (75 MHz, DMSO-d_6) δ 164.52, 162.24, 157.06, 141.66, 139.12, 137.45, 137.16, 136.26, 130.73, 130.04, 129.02, 128.87, 127.94, 126.74, 125.97, 119.98, 78.18, 60.97, 30.65, 20.76, 13.08. HRMS (ESI-TOF) *m/z*: [M+H]$^+$ Calcld for $C_{26}H_{26}N_4O$ 411.2179; Found 411.2189.

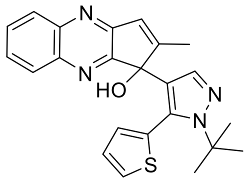

*1-(1-(tert-Butyl)-5-(thiophen-2-yl)-1H-pyrazol-4-yl)-2-methyl-1H-cyclopenta[b]quinoxalin-1-ol (**15e**)*

Grey powder; yield 61% (0.12 g); mp 235-237 °C. A mixture of isomers: ^{1}H NMR (300 MHz, DMSO-d_6) δ 7.95 (d, *J* = 6.8 Hz, 1H), 7.86 (s, 1H), 7.83 – 7.76 (m, 1H), 7.73 – 7.59 (m, 2H), 7.47 (br.s, 1H), 7.22 (br.s, 0.5 H), 6.90 (br.s, 0.5 H), 6.28 (s, 1H), 6.23 – 6.12 (m, 1.5 H), 5.59 (br.s, 0.5 H), 1.88 (s, 3H), 1.32 (s, 9H). ^{13}C NMR (75 MHz, DMSO-d_6) δ 157.30, 141.54, 139.26, 136.64, 130.60, 129.74, 129.05, 128.88, 128.63, 128.21, 127.88, 126.37, 77.98, 61.41, 30.23, 13.20. HRMS (ESI-TOF) *m/z*: [M+H]$^+$ Calcld for $C_{23}H_{22}N_4OS$ 403.1587; Found 403.1596

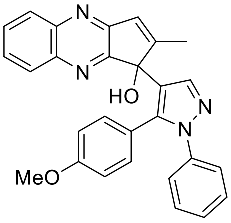

*1-(5-(4-Methoxyphenyl)-1-phenyl-1H-pyrazol-4-yl)-2-methyl-1H-cyclopenta[b]quinoxalin-1-ol (**15f**)*

Pale brown powder; yield 69% (0.15 g); mp 213-215 °C. ^{1}H NMR (300 MHz, DMSO-d_6) δ 8.06 (s, 1H), 7.88 (dd, *J* = 7.8, 1.9 Hz, 1H), 7.81 (dd, *J* = 7.9, 1.8 Hz, 1H), 7.71 – 7.58 (m, 2H), 7.29 – 7.16 (m, 3H), 7.16 – 7.08 (m, 2H), 6.48 – 6.36 (m, 3H), 6.32 – 6.23 (m, 3H), 3.48 (s, 3H), 1.97 (s, 3H). ^{13}C NMR (75 MHz, DMSO-d_6) δ 164.00, 162.52, 158.79, 157.29, 141.59, 139.97, 139.57, 139.08, 137.94, 130.94, 129.13, 128.90, 128.70, 128.13, 127.97, 127.09, 126.40, 124.58, 121.07, 120.59, 112.86, 78.01, 54.86, 13.18. HRMS (ESI-TOF) *m/z*: [M+H]$^+$ Calcld for $C_{28}H_{22}N_4O_2$ 447.1816; Found 447.1825.

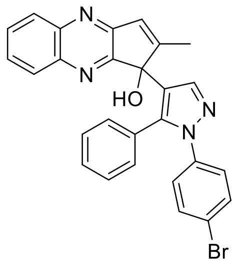

*1-(1-(4-Bromophenyl)-5-phenyl-1H-pyrazol-4-yl)-2-methyl-1H-cyclopenta[b]quinoxalin-1-ol (**15g**)*

Beige powder; yield 73% (0.18 g); mp 228-230 °C. ^{1}H NMR (300 MHz, DMSO-d_6) δ 8.11 (s, 1H), 7.89 (dd, *J* = 7.6, 2.0 Hz, 1H), 7.81 (dd, *J* = 7.7, 1.9 Hz, 1H), 7.71 – 7.57 (m, 2H), 7.42 (d, *J* = 8.8 Hz, 2H), 7.07 (d, *J* = 8.8 Hz, 2H), 7.01 – 6.92 (m, 1H), 6.84 – 6.74 (m, 2H), 6.54 (d, *J* = 7.5 Hz, 2H), 6.40 (d, *J* = 1.7 Hz, 1H), 6.35 (s, 1H), 1.95 (s, 3H). ^{13}C NMR (75 MHz, DMSO-d_6) δ 163.95, 162.25, 157.26, 141.55, 140.40, 138.99, 138.65, 138.11, 131.63, 129.62, 129.22, 128.89, 128.34, 128.20, 128.00, 127.47, 126.56, 126.42, 120.78, 119.97, 77.91, 13.16. HRMS (ESI-TOF) *m/z*: $[M+H]^+$ Calcld for $C_{27}H_{19}BrN_4O$ 495.0815; Found 495.0817.

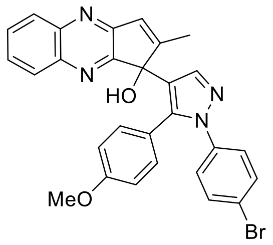

*1-(1-(4-Bromophenyl)-5-(4-methoxyphenyl)-1H-pyrazol-4-yl)-2-methyl-1H-cyclopenta[b]quinoxalin-1-ol (**15h**)*

Pale brown powder; yield 80% (0.21 g); mp 193-195 °C. ^{1}H NMR (300 MHz, DMSO-d_6) δ 8.08 (s, 1H), 7.92 – 7.78 (m, 2H), 7.73 – 7.57 (m, 2H), 7.44 (d, *J* = 8.4 Hz, 2H), 7.08 (d, *J* = 8.7 Hz, 2H), 6.52 – 6.38 (m, 3H), 6.35 – 6.25 (m, 3H), 3.49 (s, 3H), 1.96 (s, 3H). ^{13}C NMR (75 MHz, DMSO-d_6) δ 163.91, 162.48, 158.95, 157.29, 141.60, 140.41, 139.79, 139.09, 138.80, 138.04, 131.69, 130.95, 129.21, 128.93, 128.16, 128.03, 126.44, 121.00, 120.76, 119.93, 113.03, 77.99, 54.91, 13.20. HRMS (ESI-TOF) *m/z*: $[M+H]^+$ Calcld for $C_{28}H_{21}BrN_4O_2$ 525.0921; Found 525.0916.

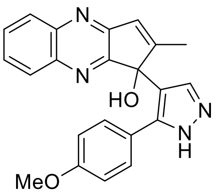

*1-(5-(4-Methoxyphenyl)-1H-pyrazol-4-yl)-2-methyl-1H-cyclopenta[b]quinoxalin-1-ol (**15i**)*

Pale brown powder; yield 65% (0.12 g); mp 222-224 °C. ^{1}H NMR (300 MHz, DMSO-d_6) δ 12.81 (s, 1H), 7.92 – 7.73 (m, 3H), 7.73 – 7.55 (m, 2H), 6.76 (d, *J* = 8.2 Hz, 2H), 6.56 (s, 1H), 6.49 (d, *J* = 8.1 Hz, 2H), 6.16 (s, 1H), 3.58 (s, 3H), 1.89 (s, 3H). ^{13}C NMR (75 MHz, DMSO-d_6) δ 164.41, 163.13, 158.97, 158.64, 141.64, 139.11, 129.88, 129.21, 128.93, 128.24, 128.06, 126.51, 116.87, 112.75, 78.22, 55.01, 13.25. HRMS (ESI-TOF) *m/z*: $[M+H]^+$ Calcld for $C_{22}H_{18}N_4O_2$ 371.1503; Found 371.1496.

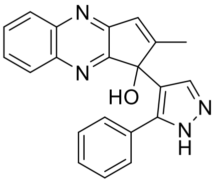

2-Methyl-1-(5-phenyl-1H-pyrazol-4-yl)-1H-cyclopenta[b]quinoxalin-1-ol (***15j***)

Pale brown powder; yield 61% (0.1 g); mp 282-284 °C. ^{1}H NMR (300 MHz, DMSO-*d*$_6$) δ 12.53 (br.s, 1H), 7.93 – 7.79 (m, 3H), 7.72 – 7.56 (m, 2H), 7.11 – 7.01 (m, 1H), 6.99 – 6.77 (m, 4H), 6.53 (s, 1H), 6.19 (s, 1H), 1.89 (s, 3H). ^{13}C NMR (75 MHz, DMSO-*d*$_6$) δ 164.46, 163.02, 157.59, 141.64, 139.10, 129.30, 128.95, 128.67, 128.31, 128.13, 127.53, 127.27, 126.65, 117.05, 78.23, 13.26. HRMS (ESI-TOF) *m/z*: [M+H]$^+$ Calcld for $C_{21}H_{16}N_4O$ 341.1397; Found 341.1394.

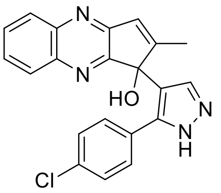

1-(5-(4-Chlorophenyl)-1H-pyrazol-4-yl)-2-methyl-1H-cyclopenta[b]quinoxalin-1-ol (***15k***)

Beige powder; yield 68% (0.13 g); mp 275-277 °C. ^{1}H NMR (300 MHz, DMSO-*d*$_6$) δ 13.00 (s, 1H), 8.04 – 7.76 (m, 3H), 7.71 – 7.53 (m, 2H), 7.24 – 6.86 (m, 4H), 6.63 (s, 1H), 6.21 (s, 1H), 1.93 (s, 3H). ^{13}C NMR (75 MHz, DMSO-*d*$_6$) δ 164.19, 163.06, 157.36, 141.60, 138.96, 130.48, 129.28, 128.87, 128.24, 128.11, 127.16, 126.63, 117.16, 78.15, 13.22. HRMS (ESI-TOF) *m/z*: [M+H]$^+$ Calcld for $C_{21}H_{15}ClN_4O$ 375.1007; Found 375.1001.

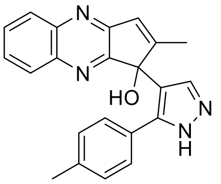

2-Methyl-1-(5-(p-tolyl)-1H-pyrazol-4-yl)-1H-cyclopenta[b]quinoxalin-1-ol (***15l***)

Pale brown powder; yield 63% (0.11 g); mp 278-280 °C. ^{1}H NMR (300 MHz, DMSO-*d*$_6$) δ 12.86 (s, 1H), 8.07 – 7.81 (m, 2H), 7.77 – 7.53 (m, 3H), 6.87 – 6.50 (m, 5H), 6.16 (s, 1H), 2.09 (s, 3H), 1.89 (s, 3H). ^{13}C NMR (75 MHz, DMSO-*d*$_6$) δ 164.35, 162.99, 157.56, 141.66, 139.08, 129.17, 128.92, 128.44, 128.23, 128.01, 127.82, 126.56, 78.17, 20.63, 13.23. HRMS (ESI-TOF) *m/z*: [M+H]$^+$ Calcld for $C_{22}H_{18}N_4O$ 355.1553; Found 355.1548.

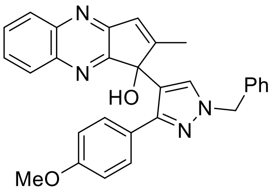

1-(1-Benzyl-3-(4-methoxyphenyl)-1H-pyrazol-4-yl)-2-methyl-1H-cyclopenta[b]quinoxalin-1-ol (***15m***)

Brown powder; yield 83% (0.19 g); mp 167-169 °C. ^{1}H NMR (300 MHz, DMSO-*d*$_6$) δ 8.02 (s, 1H), 7.87 (d, *J* = 7.9 Hz, 2H), 7.73 – 7.56

(m, 2H), 7.47 – 7.26 (m, 5H), 6.69 (d, *J* = 8.1 Hz, 2H), 6.55 (s, 1H), 6.41 (d, *J* = 8.2 Hz, 2H), 6.20 (s, 1H), 5.36 (s, 2H), 3.55 (s, 3H), 1.88 (s, 3H). ^{13}C NMR (75 MHz, DMSO-d_6) δ 164.17, 162.62, 158.25, 157.53, 147.94, 141.62, 139.05, 137.39, 131.49, 129.65, 129.14, 128.90, 128.58, 128.19, 127.95, 127.72, 126.70, 125.99, 118.11, 112.43, 78.09, 54.87, 13.13. HRMS (ESI-TOF) *m/z*: [M+H]$^+$ Calcld for $C_{29}H_{24}N_4O_2$ 461.1972; Found 461.1966.

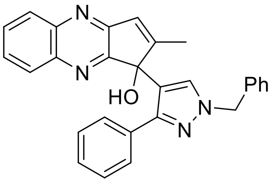

*1-(1-Benzyl-3-phenyl-1H-pyrazol-4-yl)-2-methyl-1H-cyclopenta[b]quinoxalin-1-ol (**15n**)*

Brown powder; yield 69% (0.15 g); mp 162-164 °C. ^{1}H NMR (300 MHz, DMSO-d_6) δ 8.05 (s, 1H), 7.91 – 7.81 (m, 2H), 7.73 – 7.54 (m, 2H), 7.45 – 7.26 (m, 5H), 7.03 – 6.92 (m, 1H), 6.92 – 6.81 (m, 2H), 6.78 (d, *J* = 7.5 Hz, 2H), 6.53 (d, *J* = 2.0 Hz, 1H), 6.22 (s, 1H), 5.38 (s, 2H), 1.87 (s, 3H). ^{13}C NMR (75 MHz, DMSO-d_6) δ 164.16, 162.52, 157.55, 148.17, 141.59, 139.00, 137.34, 133.68, 131.65, 129.20, 128.90, 128.60, 128.45, 128.23, 127.96, 127.76, 127.06, 126.97, 126.78, 118.13, 78.07, 54.96, 13.12. HRMS (ESI-TOF) *m/z*: [M+H]$^+$ Calcld for $C_{28}H_{22}N_4O$ 431.1866; Found 431.1872 .

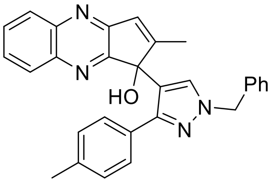

*1-(1-Benzyl-3-(p-tolyl)-1H-pyrazol-4-yl)-2-methyl-1H-cyclopenta[b]quinoxalin-1-ol (**15o**)*

Whate powder; yield 82% (0.19 g); mp 184-186 °C. ^{1}H NMR (300 MHz, DMSO-d_6) δ 8.05 (s, 1H), 7.92 – 7.81 (m, 2H), 7.72 – 7.58 (m, 2H), 7.44 – 7.28 (m, 5H), 6.72 – 6.59 (m, 4H), 6.55 (s, 1H), 6.22 (s, 1H), 5.37 (s, 2H), 2.06 (s, 3H), 1.87 (s, 3H). ^{13}C NMR (75 MHz, DMSO-d_6) δ 164.13, 162.53, 157.59, 148.12, 141.66, 139.04, 137.39, 136.20, 131.66, 130.80, 129.17, 128.92, 128.61, 128.30, 128.20, 127.96, 127.75, 127.58, 126.78, 118.10, 78.08, 54.95, 20.57, 13.13. HRMS (ESI-TOF) *m/z*: [M+H]$^+$ Calcld for $C_{29}H_{24}N_4O$ 445.2023; Found 445.2034.

*Experimental procedure for the synthesis of compound **18**.*

A mixture of compound **12a** (1 mmol, 0.35 g) and $NaBH_3CN$ (5 mmol, 0.31 g) in $MeOH_{abs}$ (10 ml) was stirred for 24 h at room temperature. The obtained mixture was poured into

H_2O (100 ml) and AcOH (10 mmol, 0.6 g) was added. Then solution was extracted with $CHCl_3$ (3×25 ml). The combined $CHCl_3$ extract was dried over Na_2SO_4. The organic layer was evaporated in vacuum and the residue was recrystallized from Et_2O (5 ml). The resulting precipitate was filtered off and washed Et_2O (3 x 5 ml) to give the corresponding product **18**.

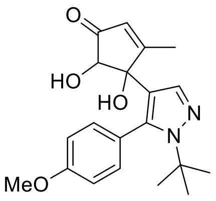

*4-(1-(tert-Butyl)-5-(4-methoxyphenyl)-1H-pyrazol-4-yl)-4,5-dihydroxy-3-methylcyclopent-2-en-1-one (**18**).*

White powder; yield 67% (0.24 g); mp 170-172 °C. ^{1}H NMR (300 MHz, DMSO-d_6) δ 7.39 (s, 1H), 7.09 (dd, J = 8.4, 2.3 Hz, 1H), 7.04 – 6.98 (m, 1H), 6.84 (dd, J = 8.4, 2.8 Hz, 1H), 6.72 (dd, J = 8.5, 2.7 Hz, 1H), 5.52 (s, 1H), 5.37 – 5.29 (m, 2H), 3.96 (s, 1H), 3.76 (s, 3H), 1.77 (s, 3H), 1.27 (s, 9H). ^{13}C NMR (75 MHz, DMSO-d_6) δ 202.56, 173.37, 159.13, 138.16, 136.39, 133.43, 131.95, 127.54, 124.42, 120.25, 112.52, 112.23, 82.17, 80.73, 60.61, 55.05, 30.77, 14.68. HRMS (ESI-TOF) *m/z*: $[M+H]^+$ Calcld for $C_{20}H_{24}N_2O_4$ 357.1809; Found 357.1807.

5. Copies of ^{1}H and ^{13}C NMR spectra for compounds **19**, **20**, **12** and **16**.

^{1}H NMR spectrum (300 MHz) of **19c** in DMSO-d_6

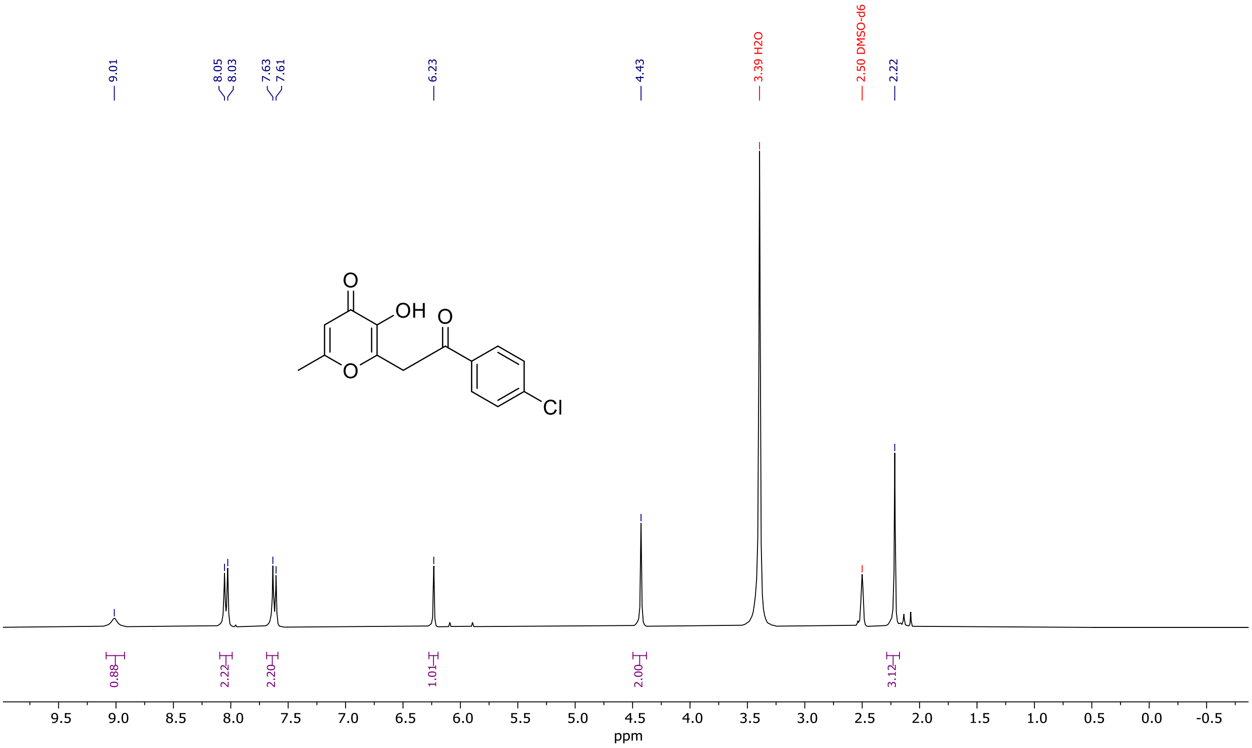

^{13}C {^{1}H} NMR spectrum (75 MHz) of **19c** in DMSO-d_6

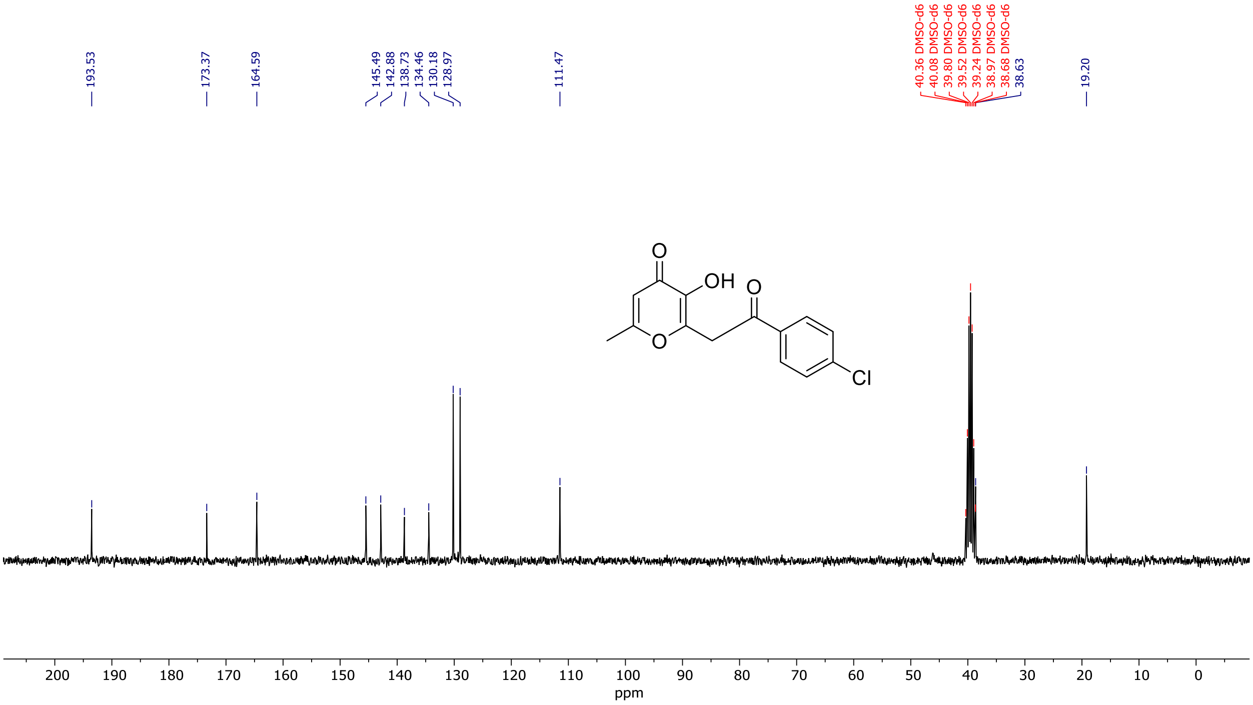

^{1}H NMR spectrum (300 MHz) of **19d** in DMSO-d_6

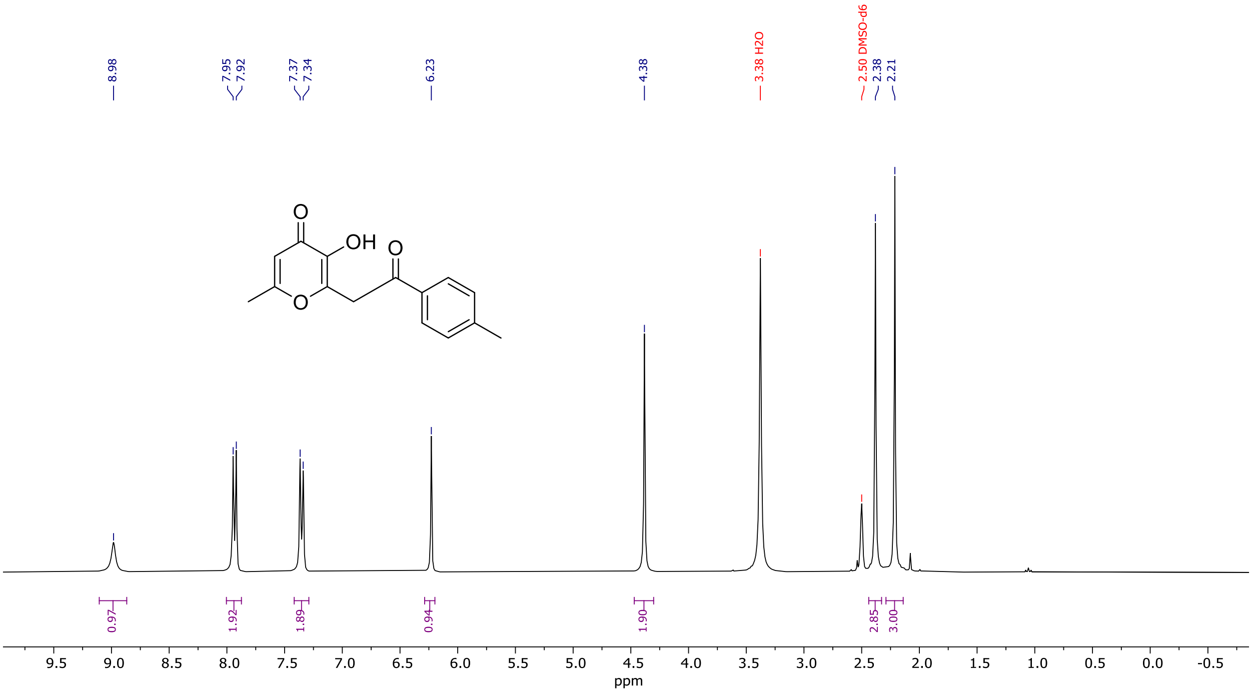

^{13}C {^{1}H} NMR spectrum (75 MHz) of **19d** in DMSO-d_6

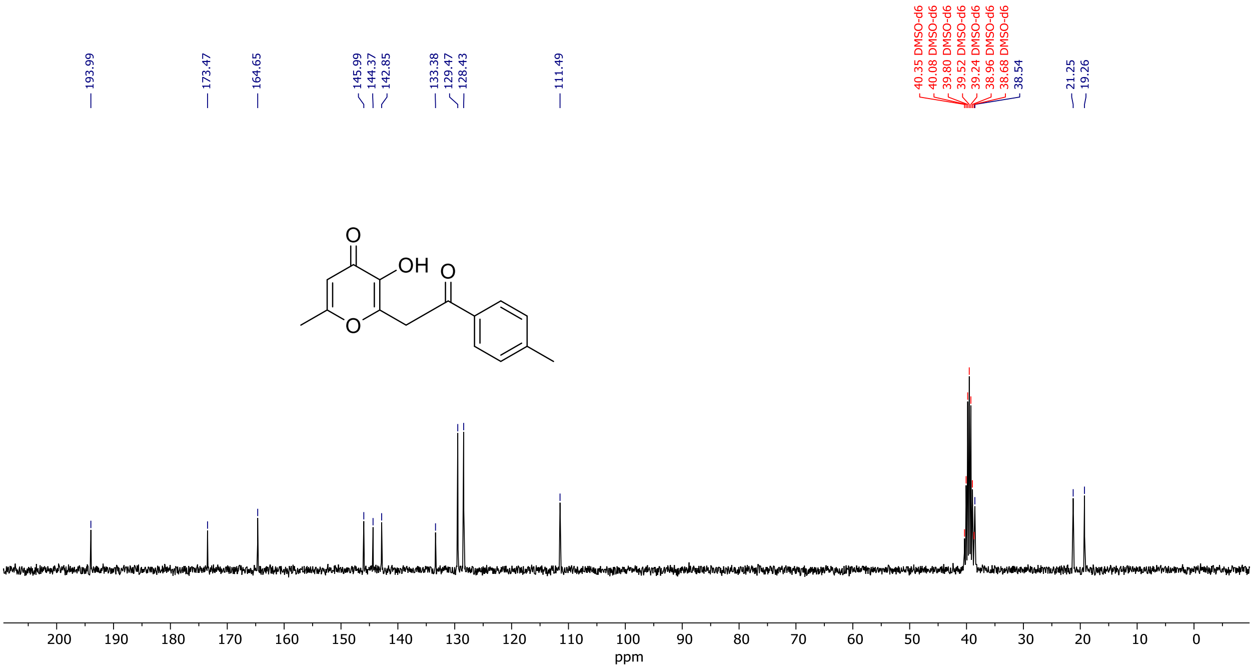

^{1}H NMR spectrum (300 MHz) of **19e** in DMSO-d_6

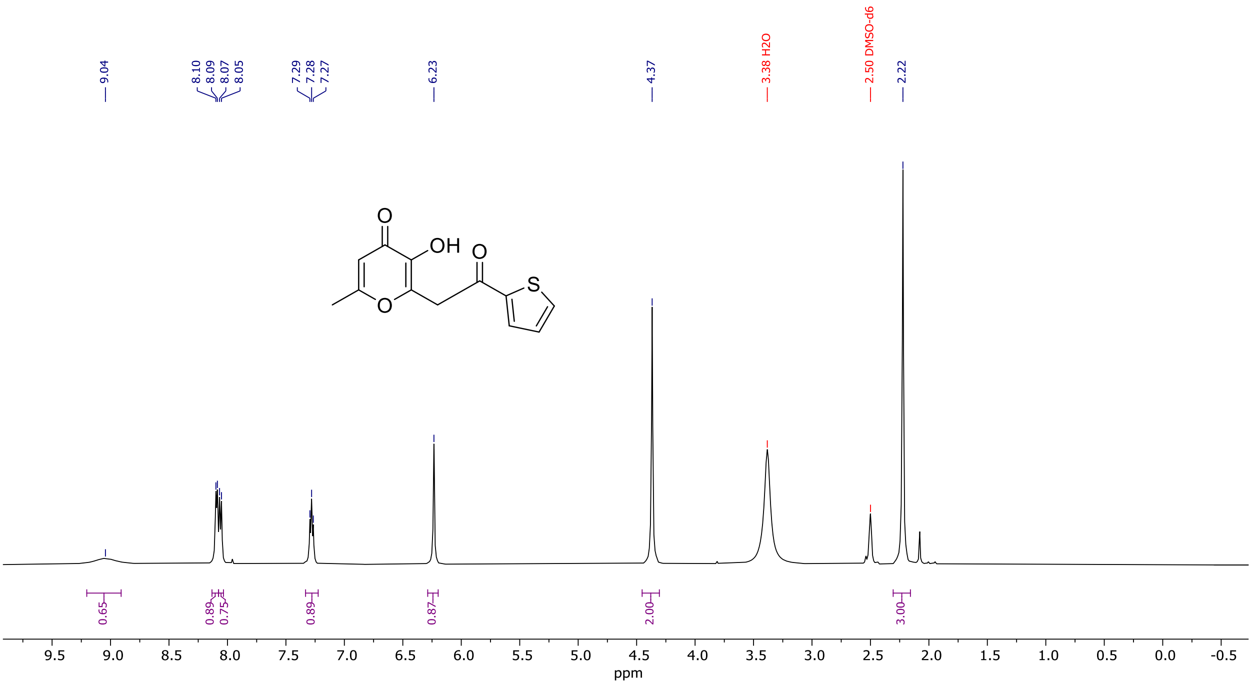

^{13}C {^{1}H} NMR spectrum (75 MHz) of **19e** in DMSO-d_6

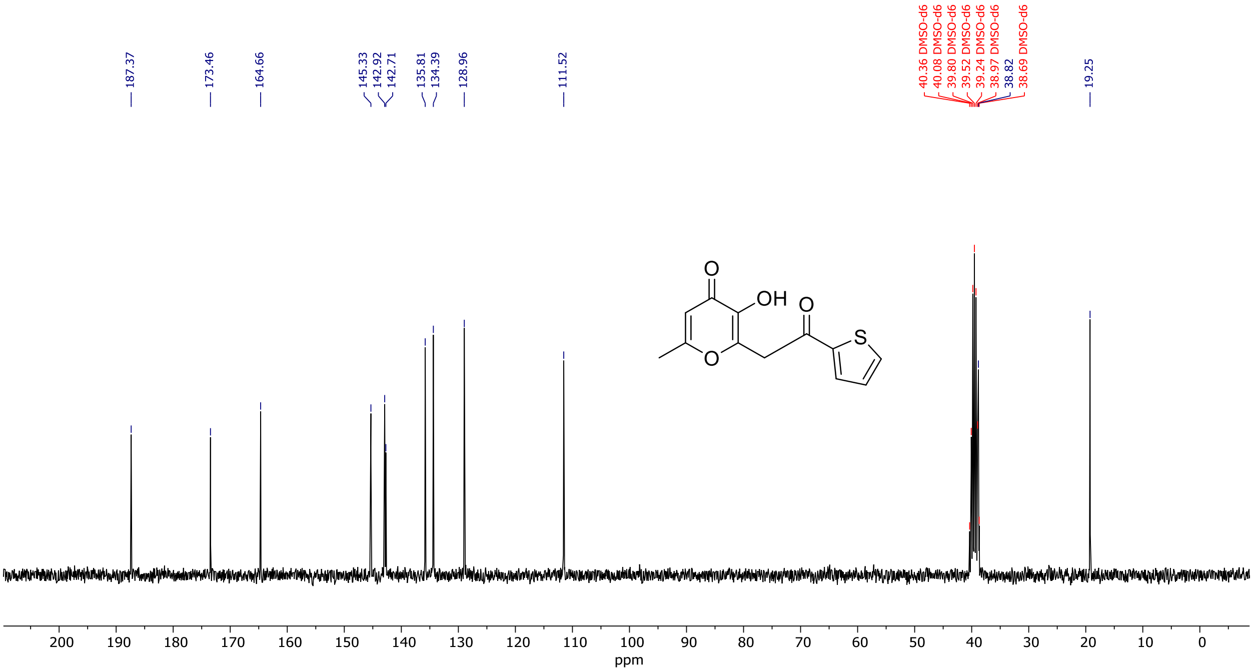

^{1}H NMR spectrum (300 MHz) of **20c** in DMSO-d_6

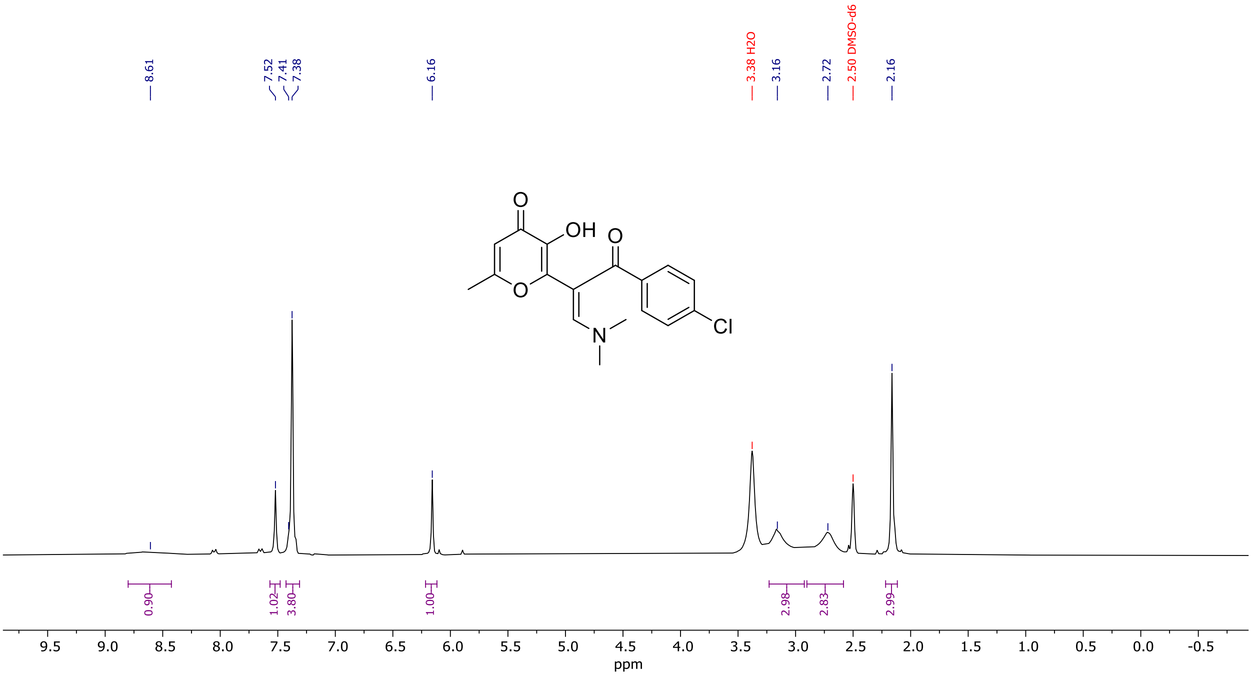

^{13}C {^{1}H} NMR spectrum (75 MHz) of **20c** in DMSO-d_6

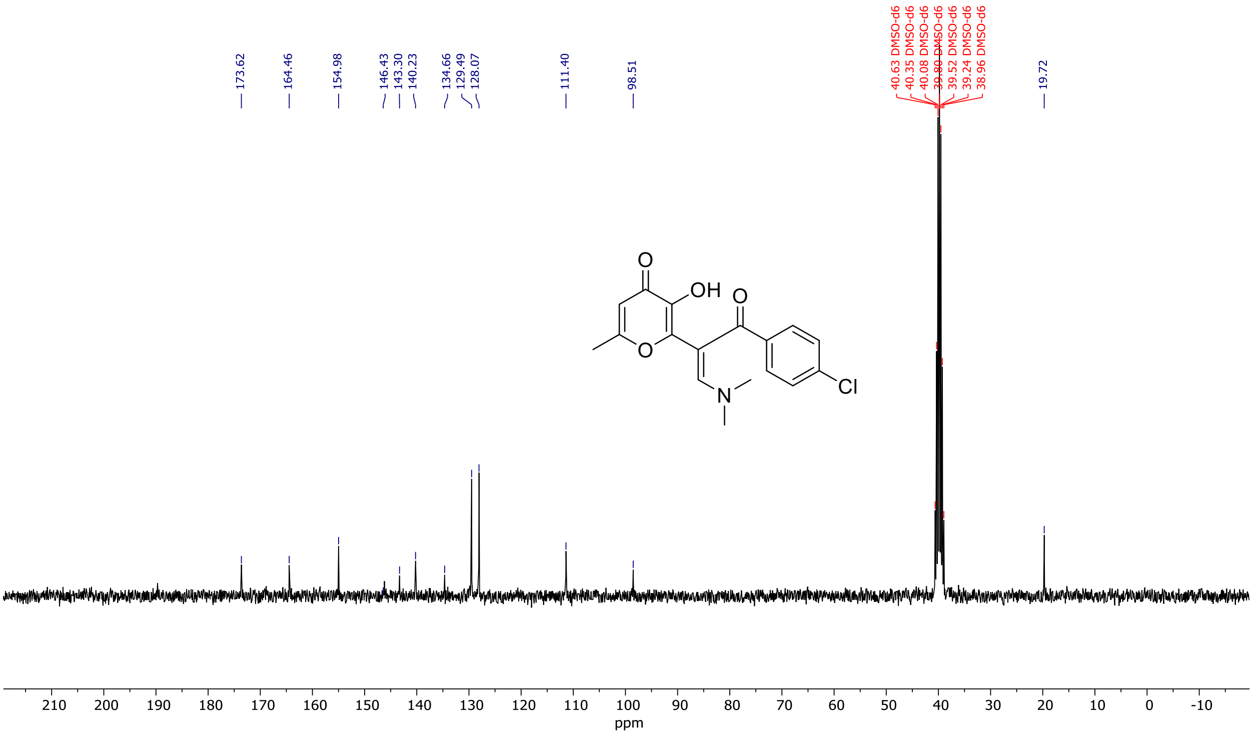

^{1}H NMR spectrum (300 MHz) of **20d** in DMSO-d_6

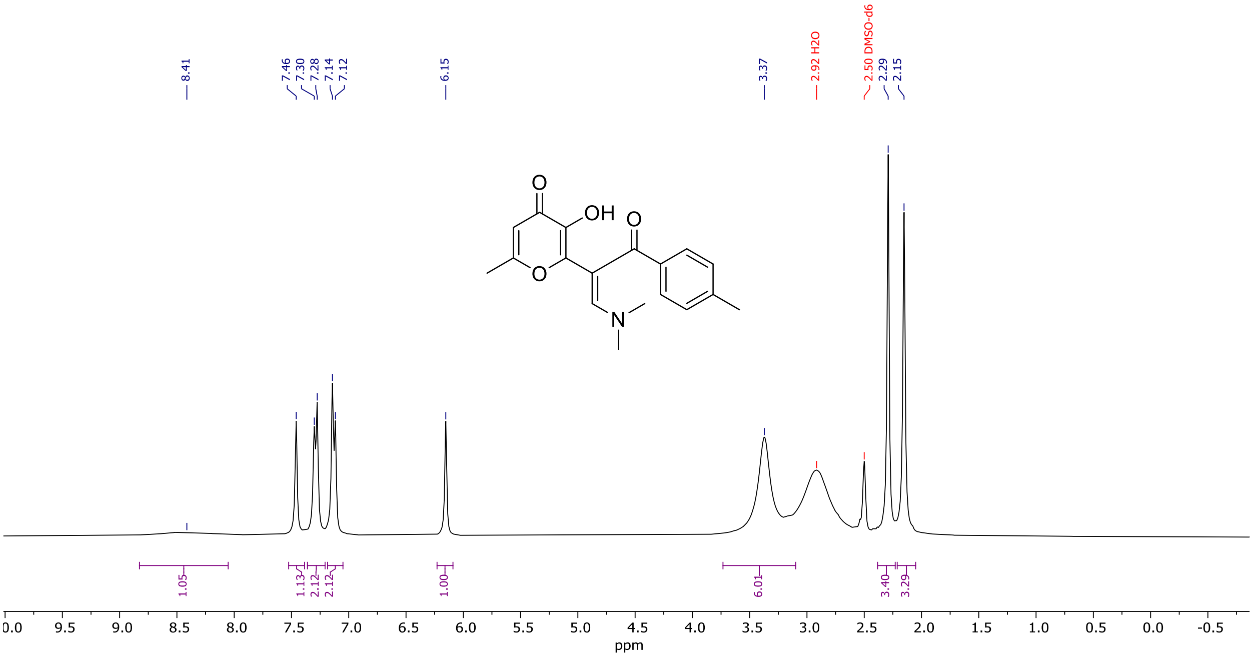

^{13}C {^{1}H} NMR spectrum (75 MHz) of **20d** in DMSO-d_6

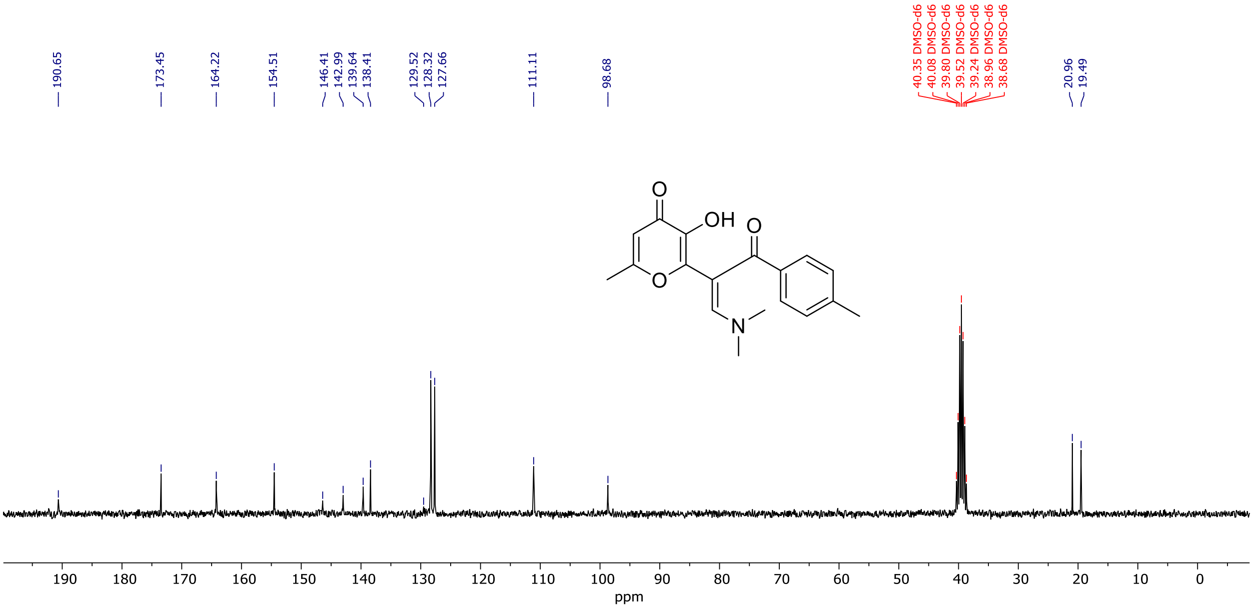

^1H NMR spectrum (300 MHz) of **20e** in DMSO-d_6

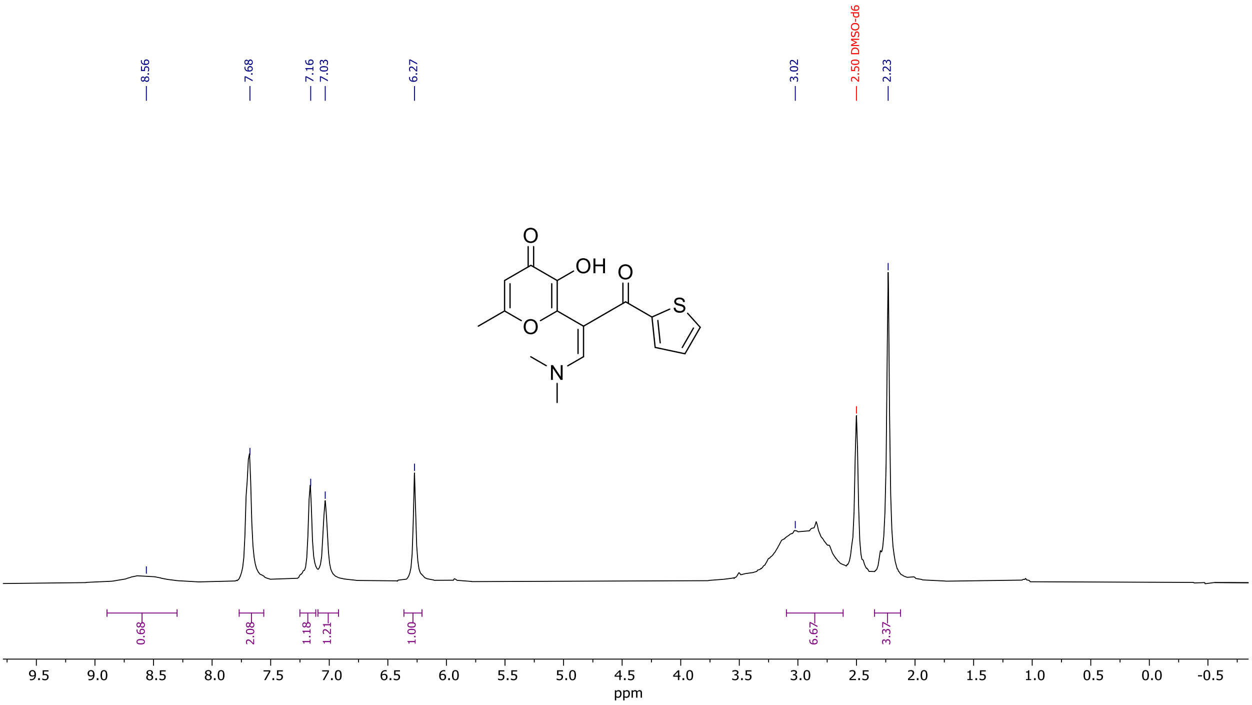

^{13}C {^1H} NMR spectrum (75 MHz) of **20e** in DMSO-d_6

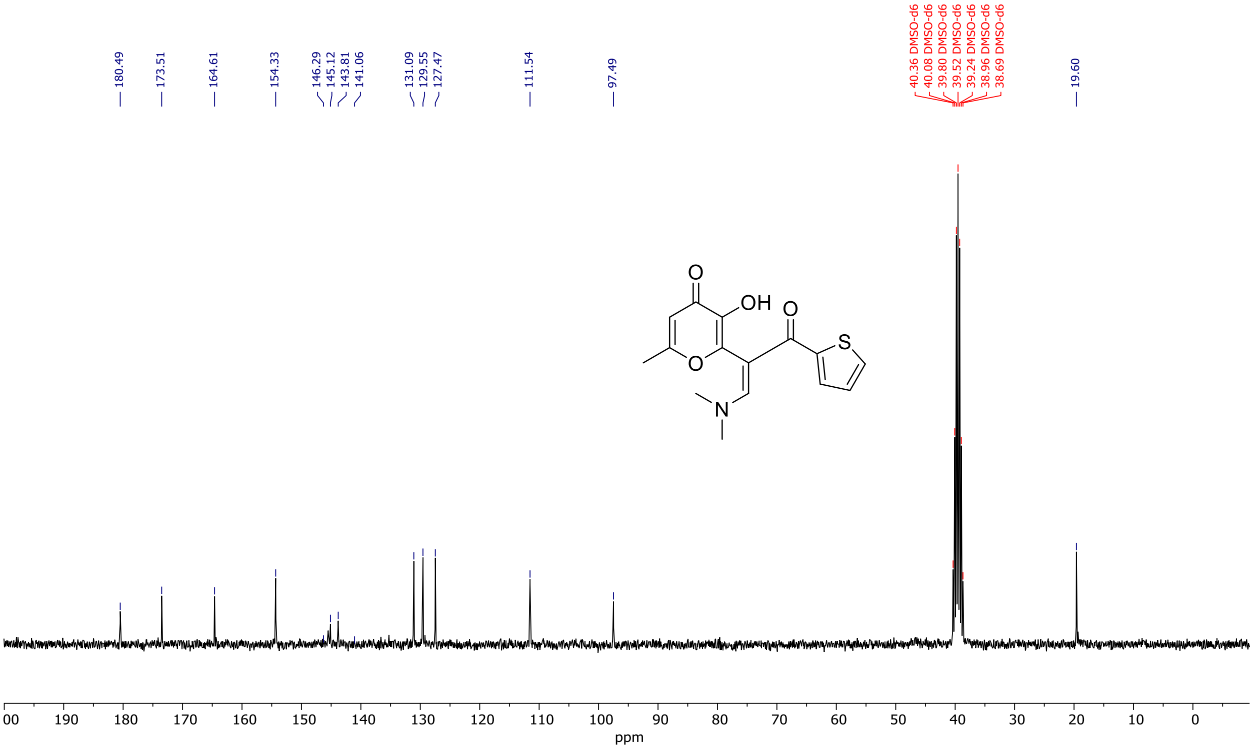

^{1}H NMR spectrum (300 MHz) of **12b** in DMSO-d_6

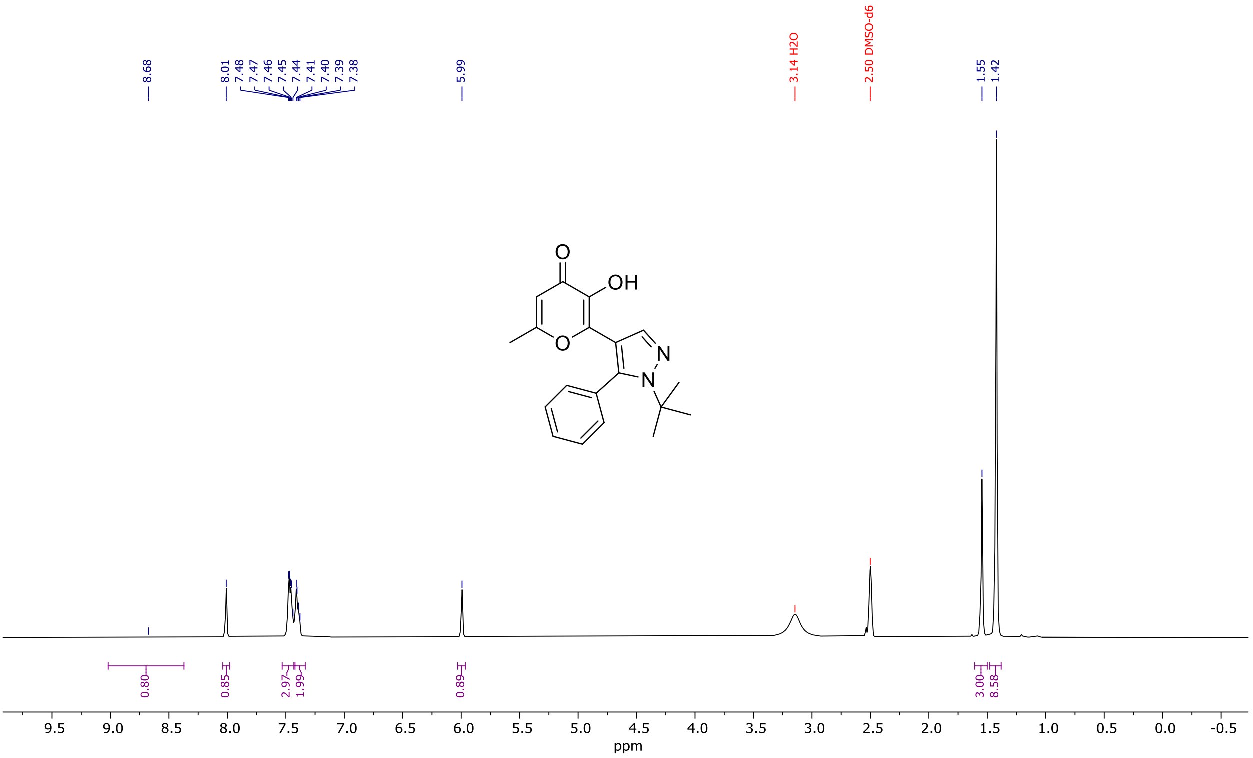

^{13}C {^{1}H} NMR spectrum (75 MHz) of **12b** in DMSO-d_6

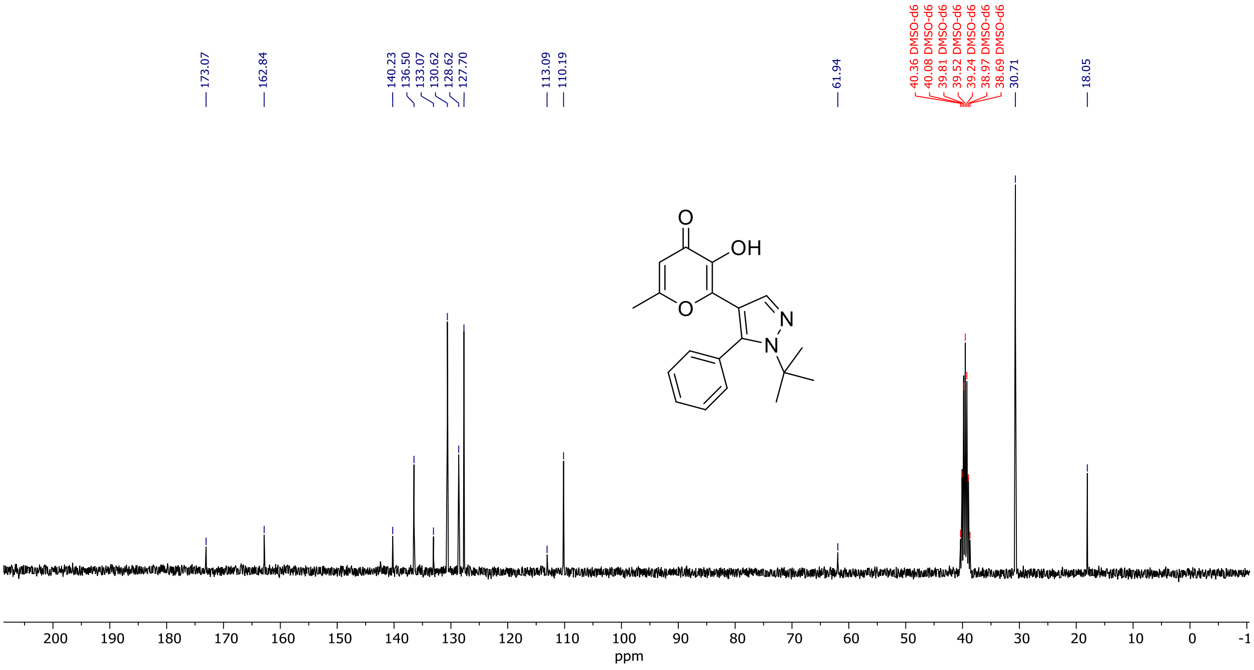

^{1}H NMR spectrum (300 MHz) of **12c** in DMSO-d_6

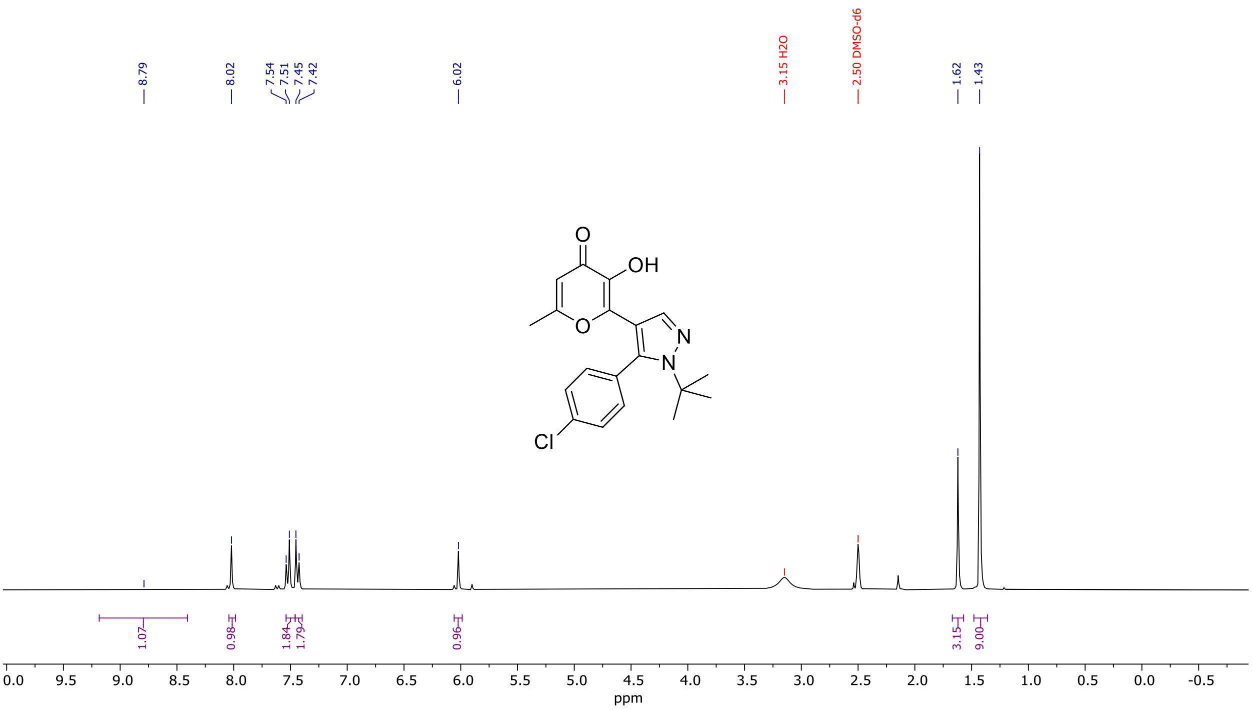

^{13}C {^{1}H} NMR spectrum (75 MHz) of **12c** in DMSO-d_6

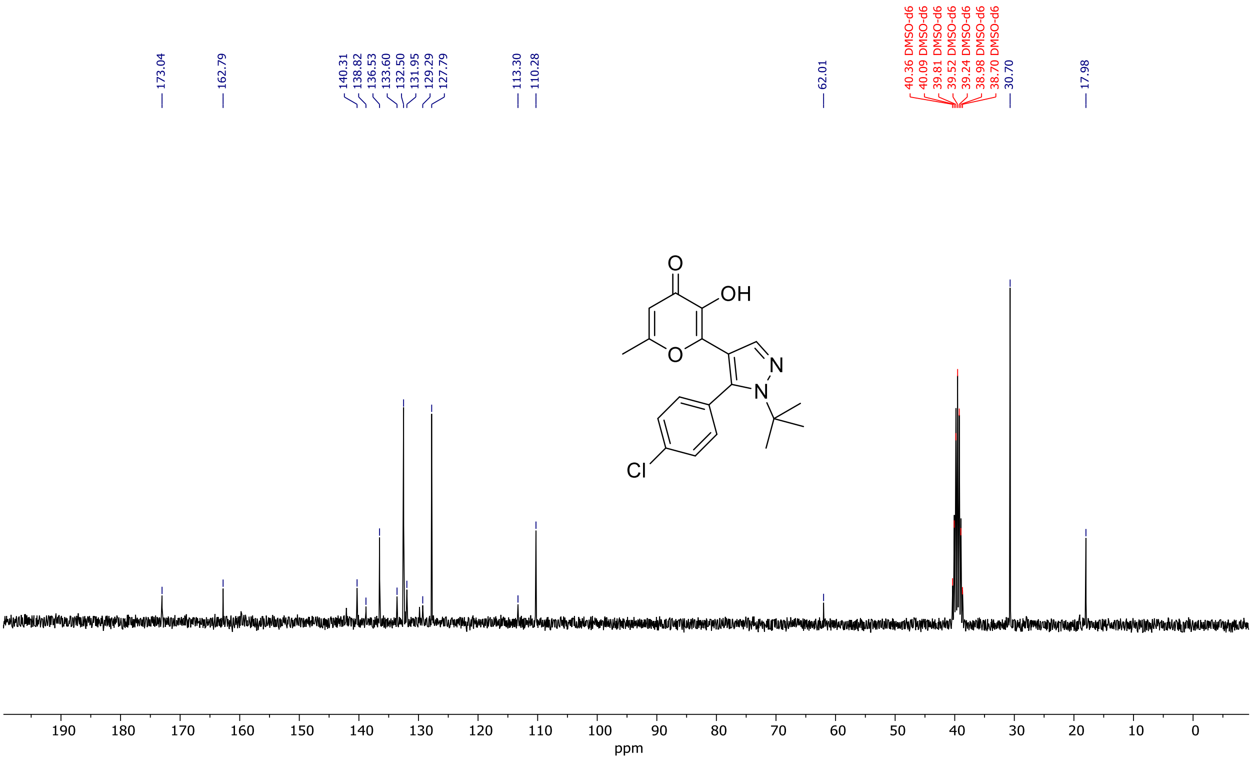

^{1}H NMR spectrum (300 MHz) of **12d** in DMSO-d_6

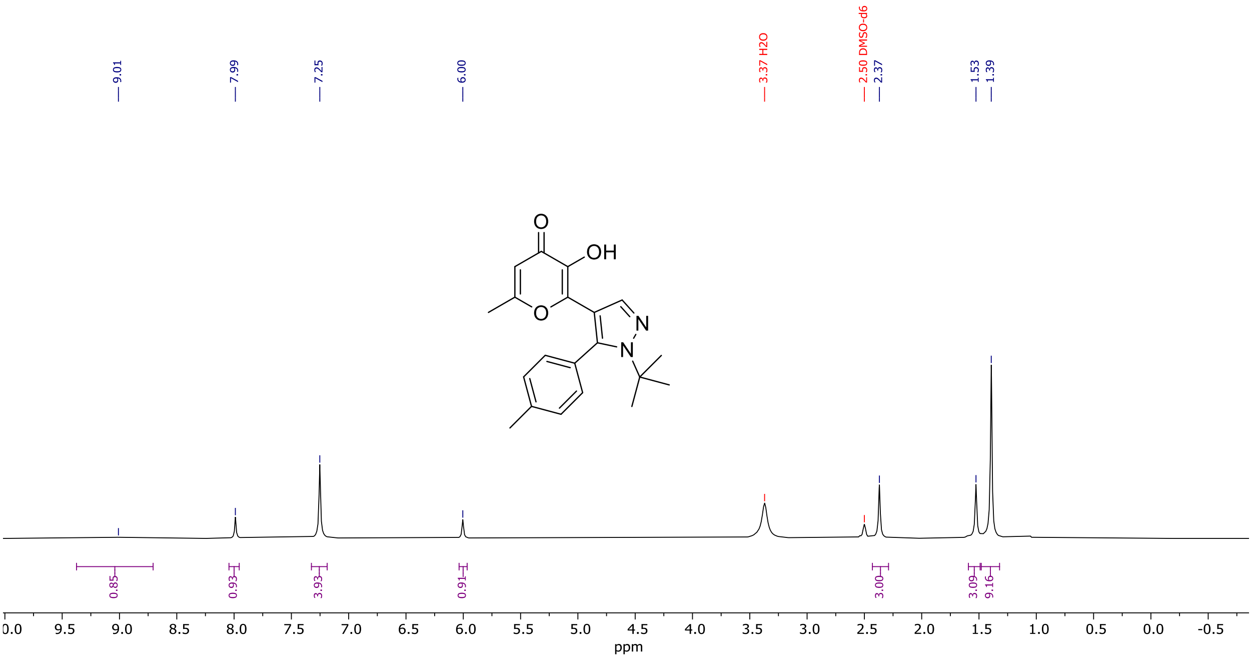

^{13}C {^{1}H} NMR spectrum (126 MHz) of **12d** in DMSO-d_6

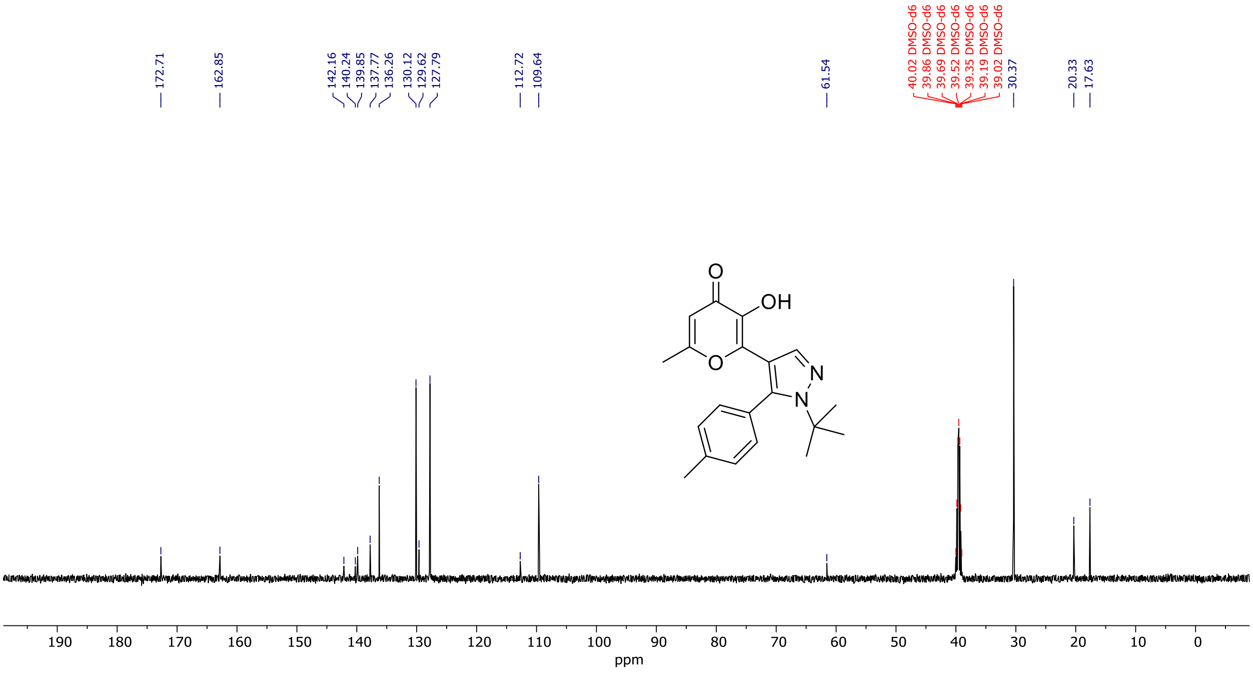

^{1}H NMR spectrum (300 MHz) of **12e** in DMSO-d_6

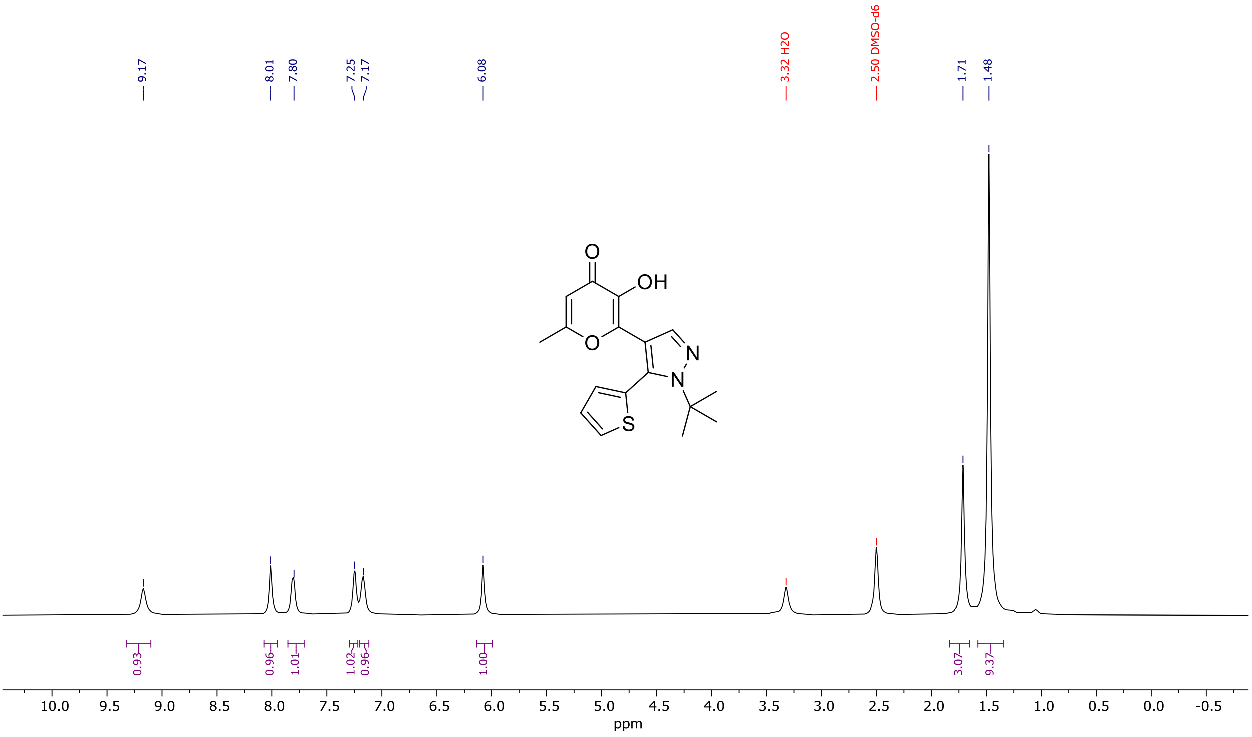

^{13}C {^{1}H} NMR spectrum (75 MHz) of **12e** in DMSO-d_6

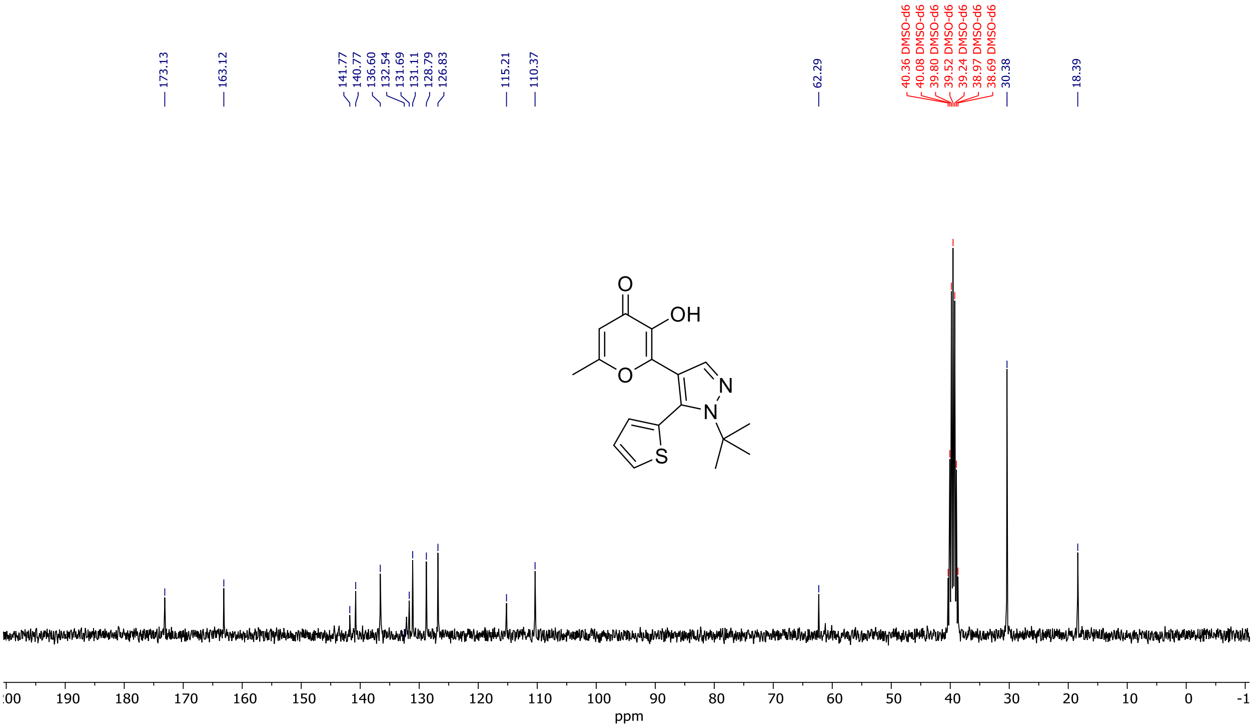

^{1}H NMR spectrum (300 MHz) of **12g** in DMSO-d_6

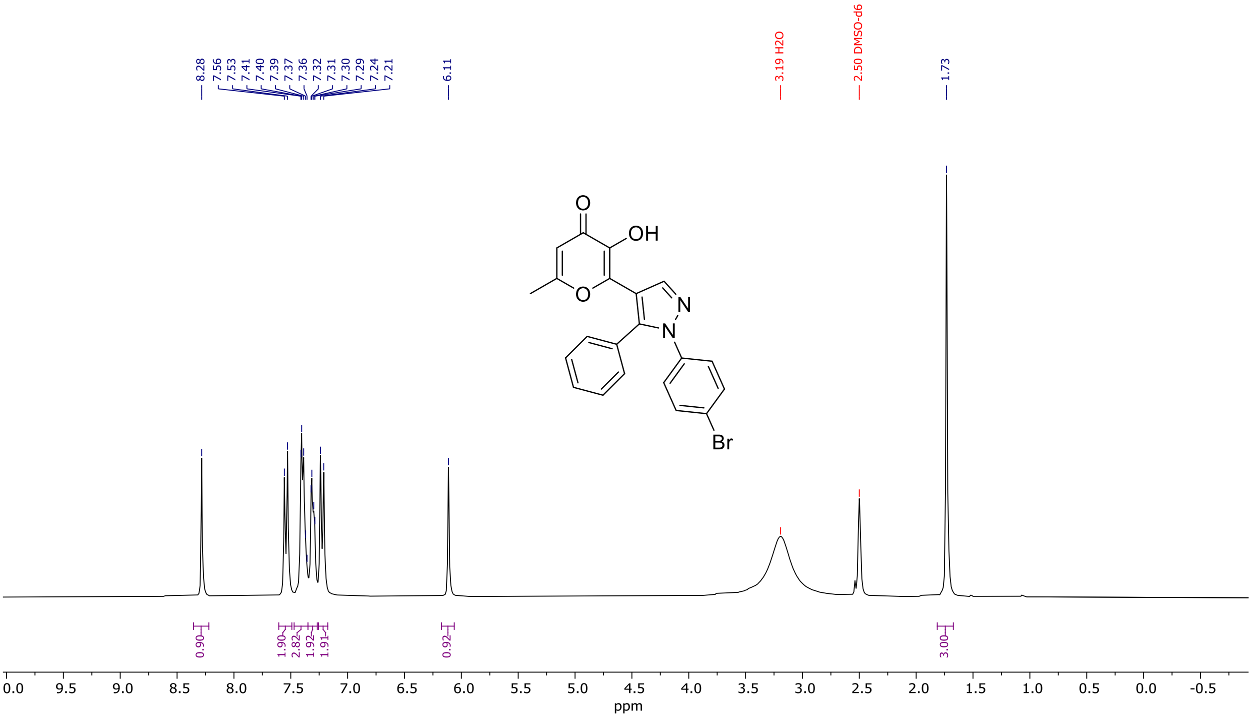

^{13}C {^{1}H} NMR spectrum (75 MHz) of **12g** in DMSO-d_6

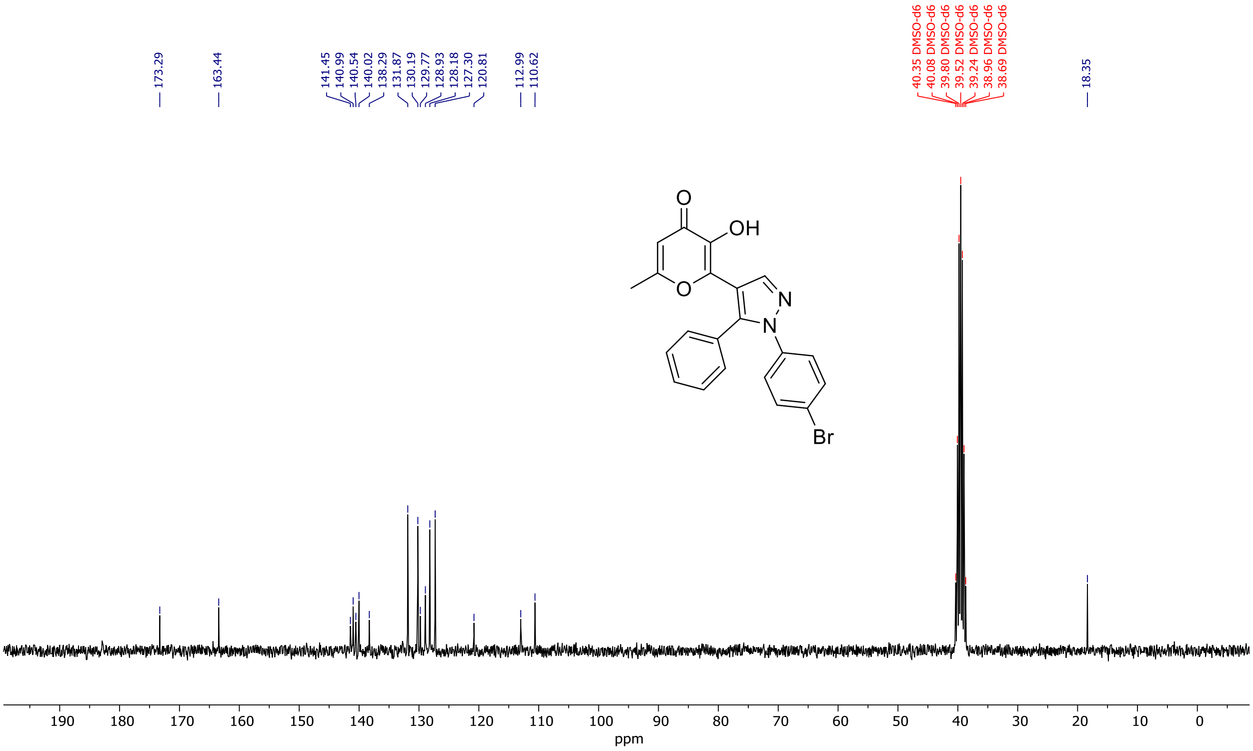

^{1}H NMR spectrum (300 MHz) of **12j** in DMSO-d_6

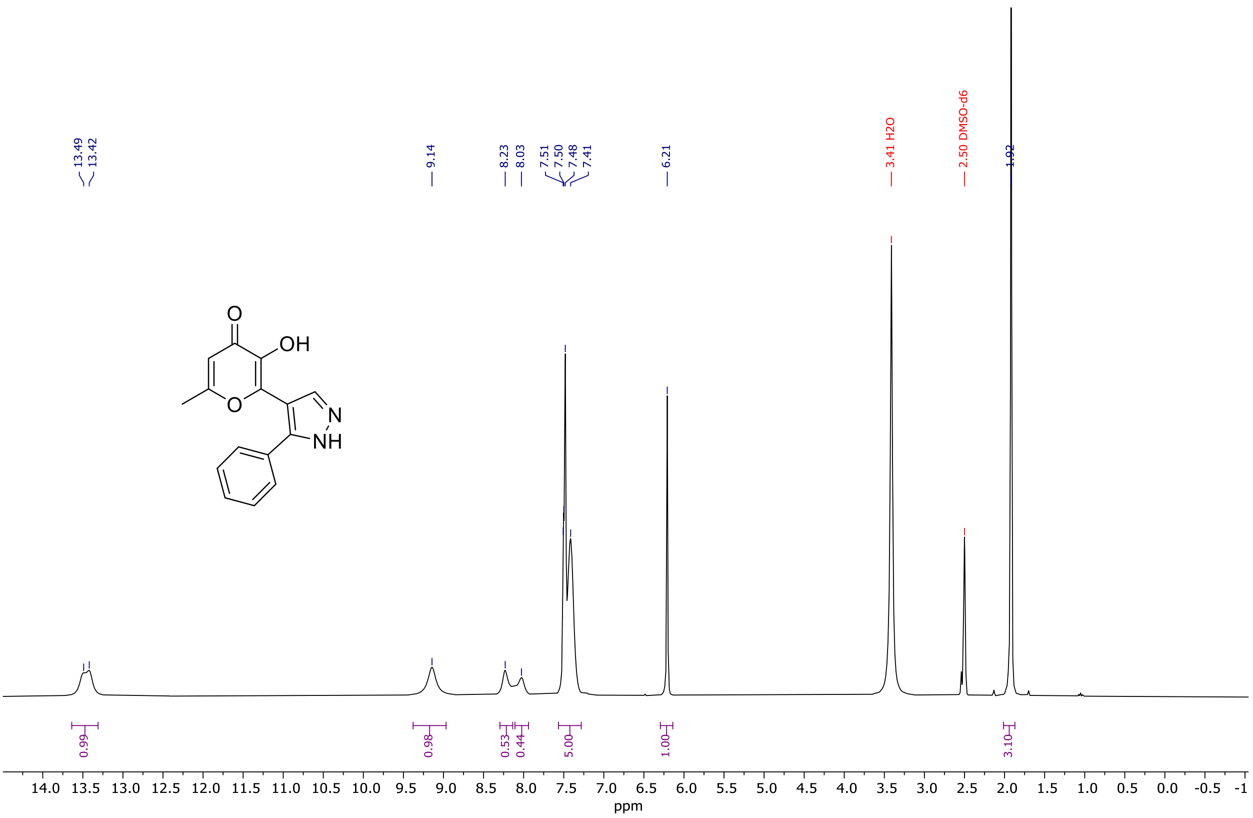

^{13}C {^{1}H} NMR spectrum (75 MHz) of **12j** in DMSO-d_6

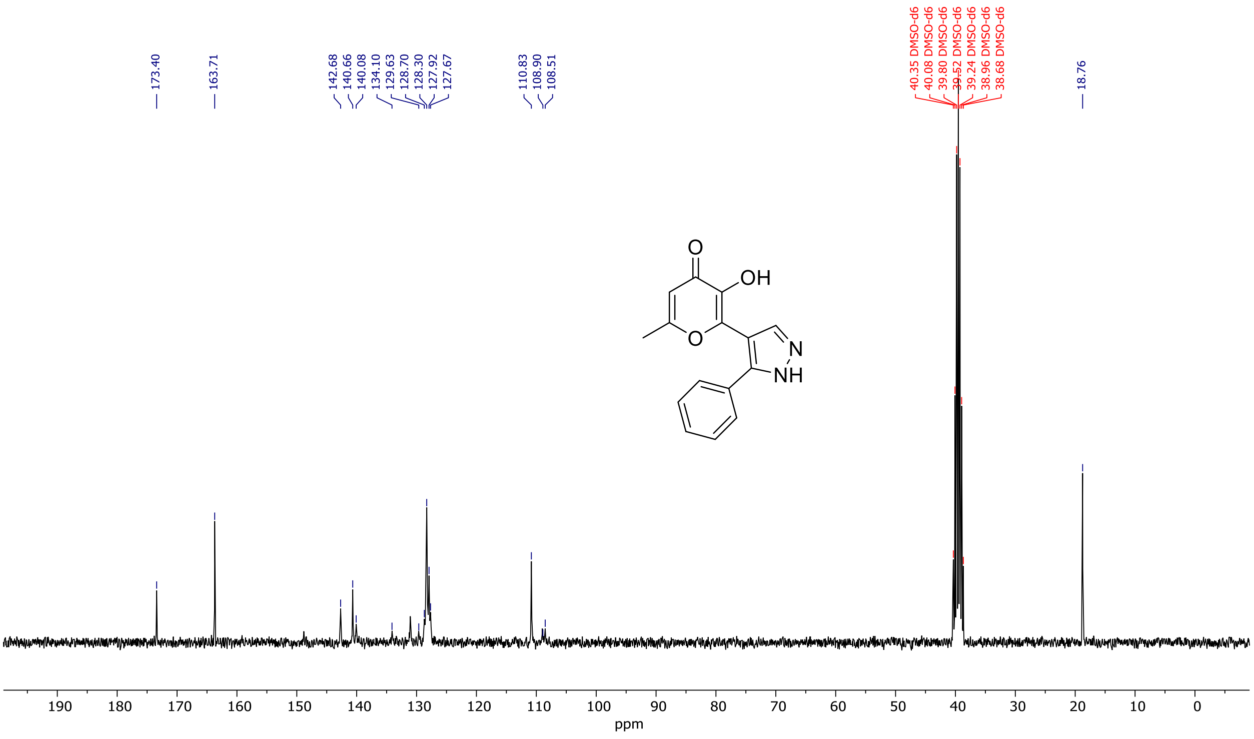

^{1}H NMR spectrum (300 MHz) of **12k** in DMSO-d_6

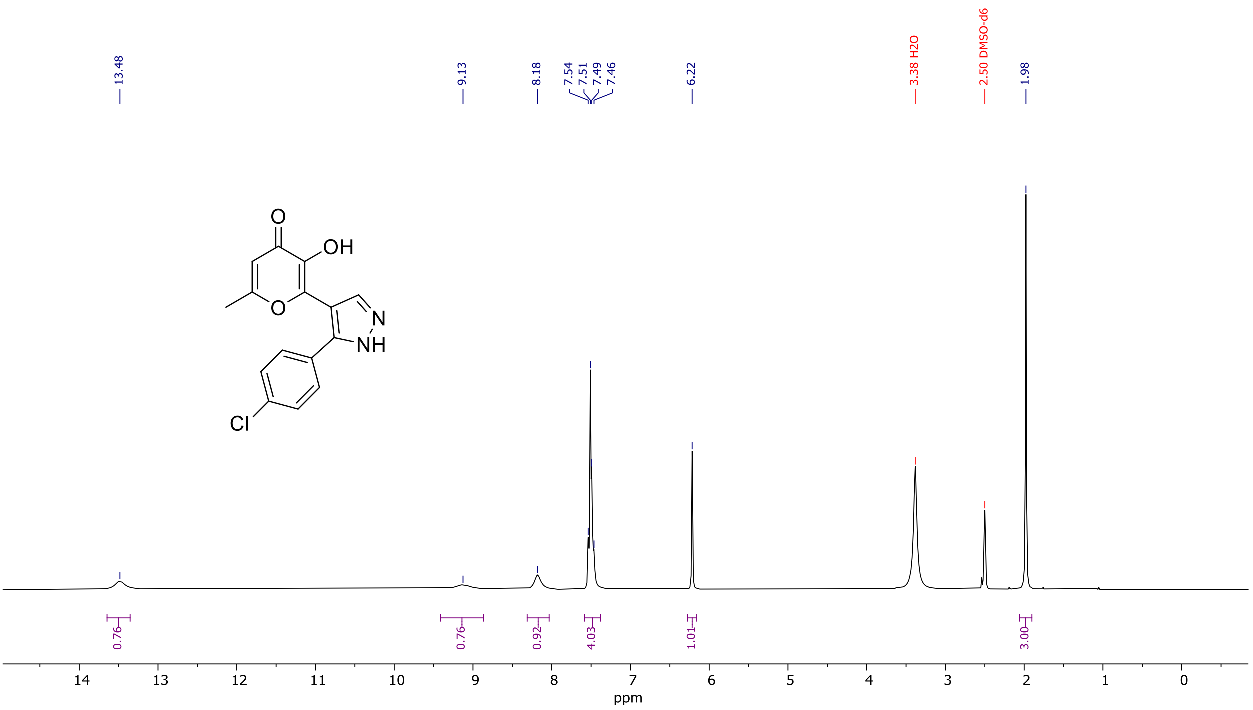

^{13}C {^{1}H} NMR spectrum (75 MHz) of **12k** in DMSO-d_6

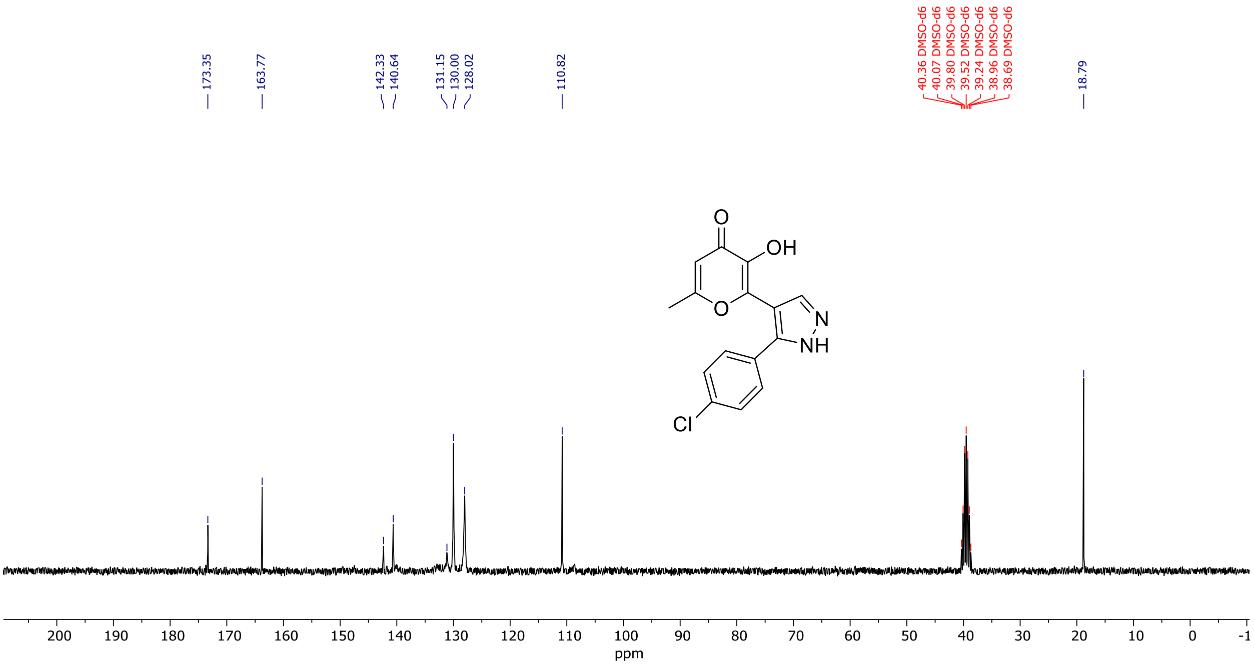

^{1}H NMR spectrum (300 MHz) of **12l** in DMSO-d_6

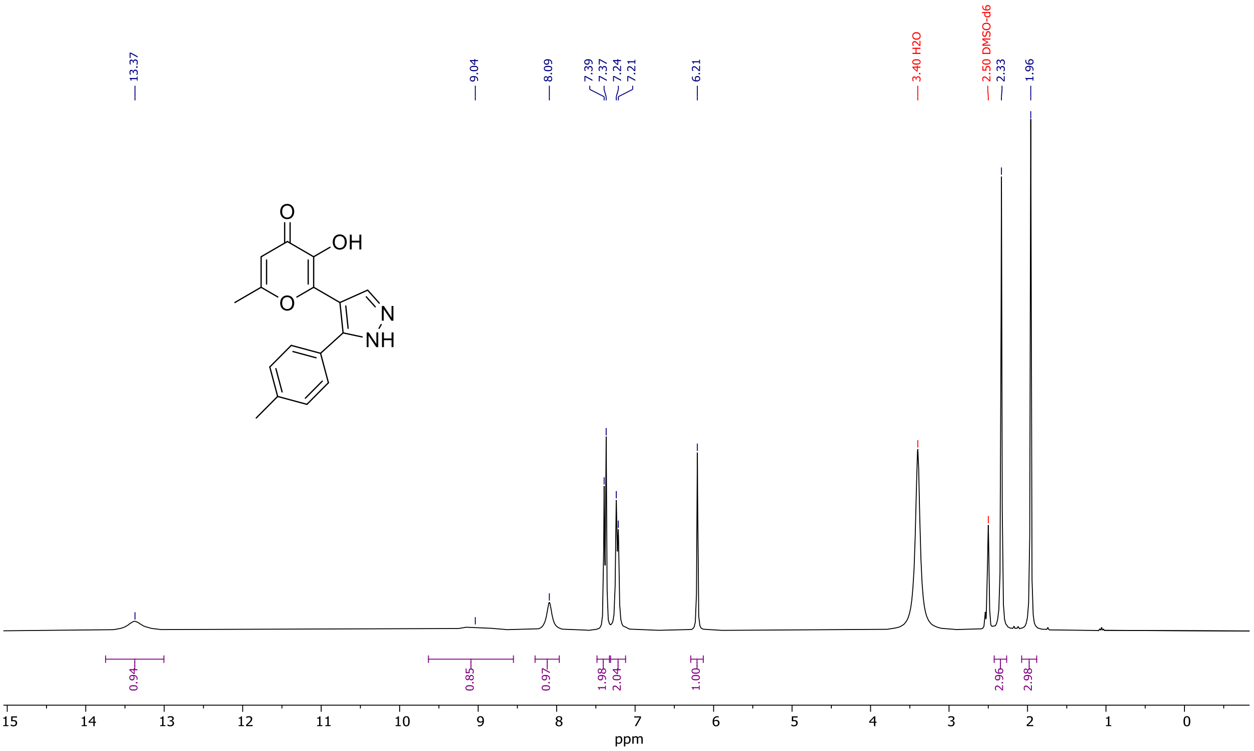

^{13}C {^{1}H} NMR spectrum (75 MHz) of **12l** in DMSO-d_6

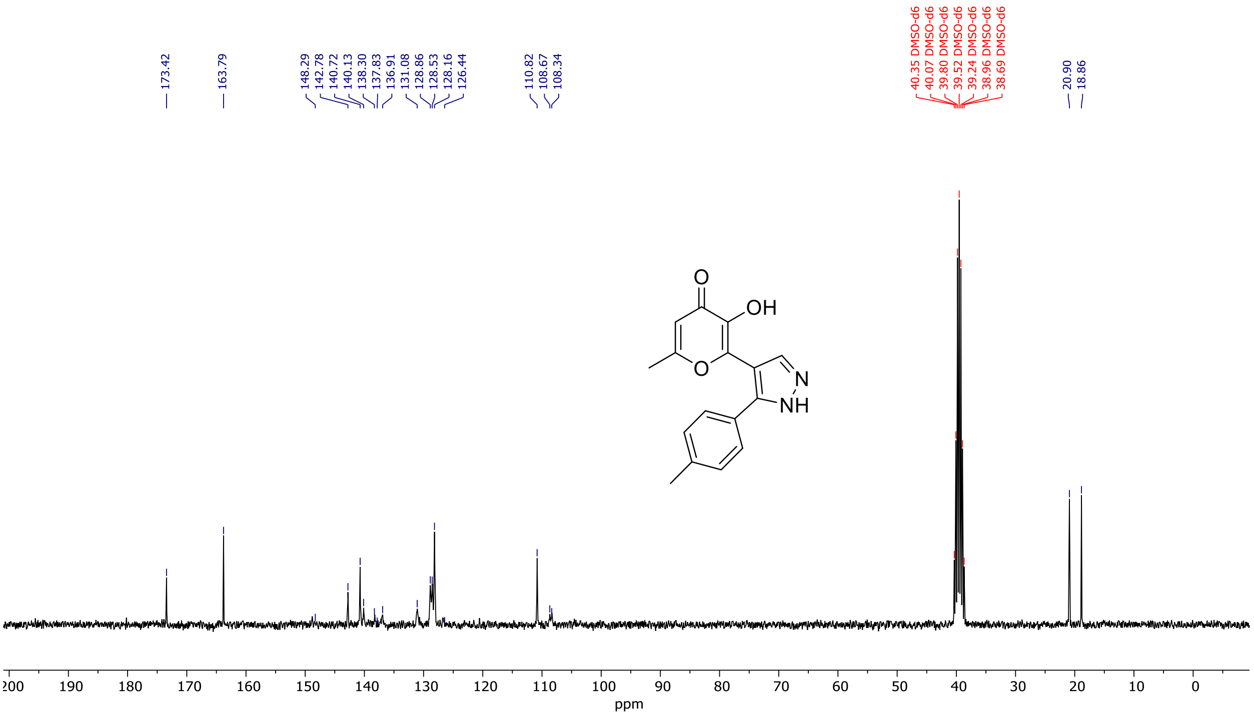

^1H NMR spectrum (300 MHz) of **12m** in DMSO-d_6

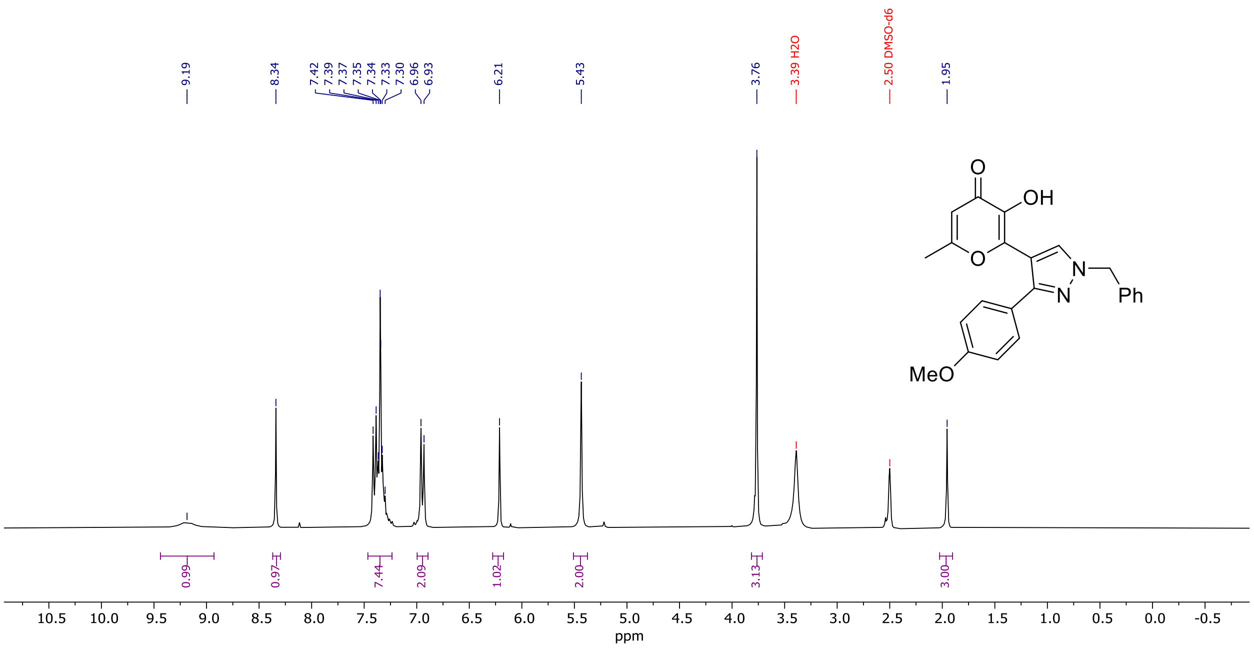

^{13}C {^1H} NMR spectrum (75 MHz) of **12m** in DMSO-d_6

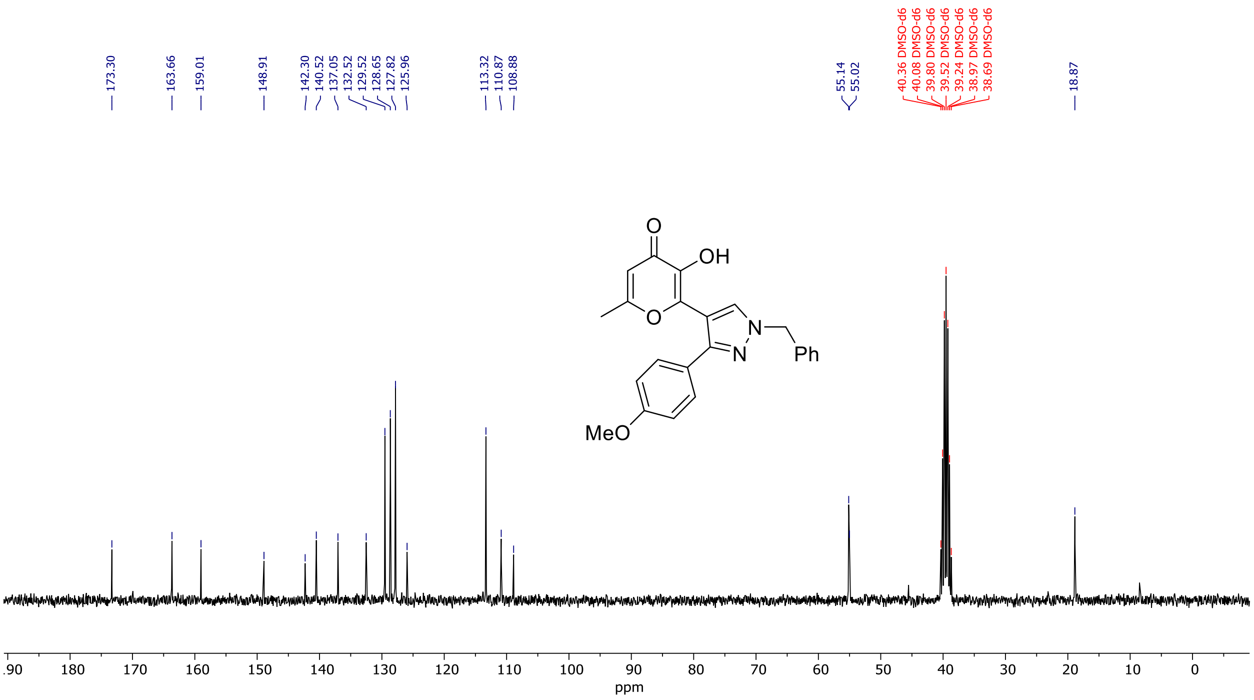

^{1}H NMR spectrum (300 MHz) of **12n** in DMSO-d_6

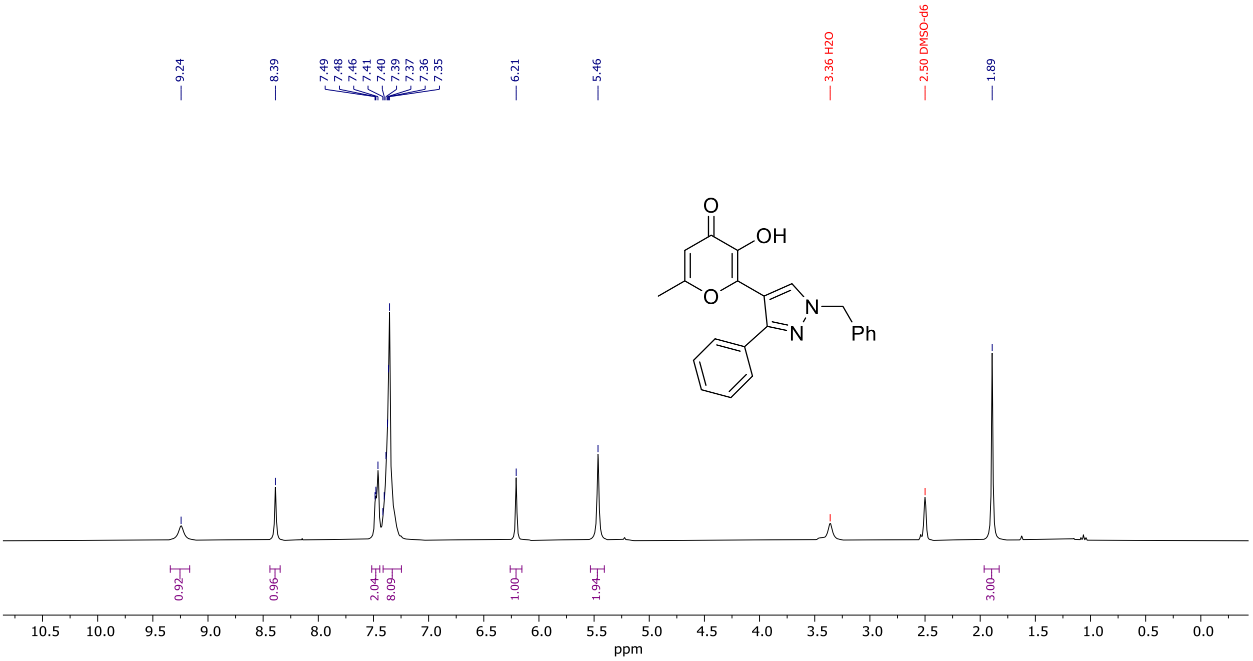

^{13}C {^{1}H} NMR spectrum (75 MHz) of **12n** in DMSO-d_6

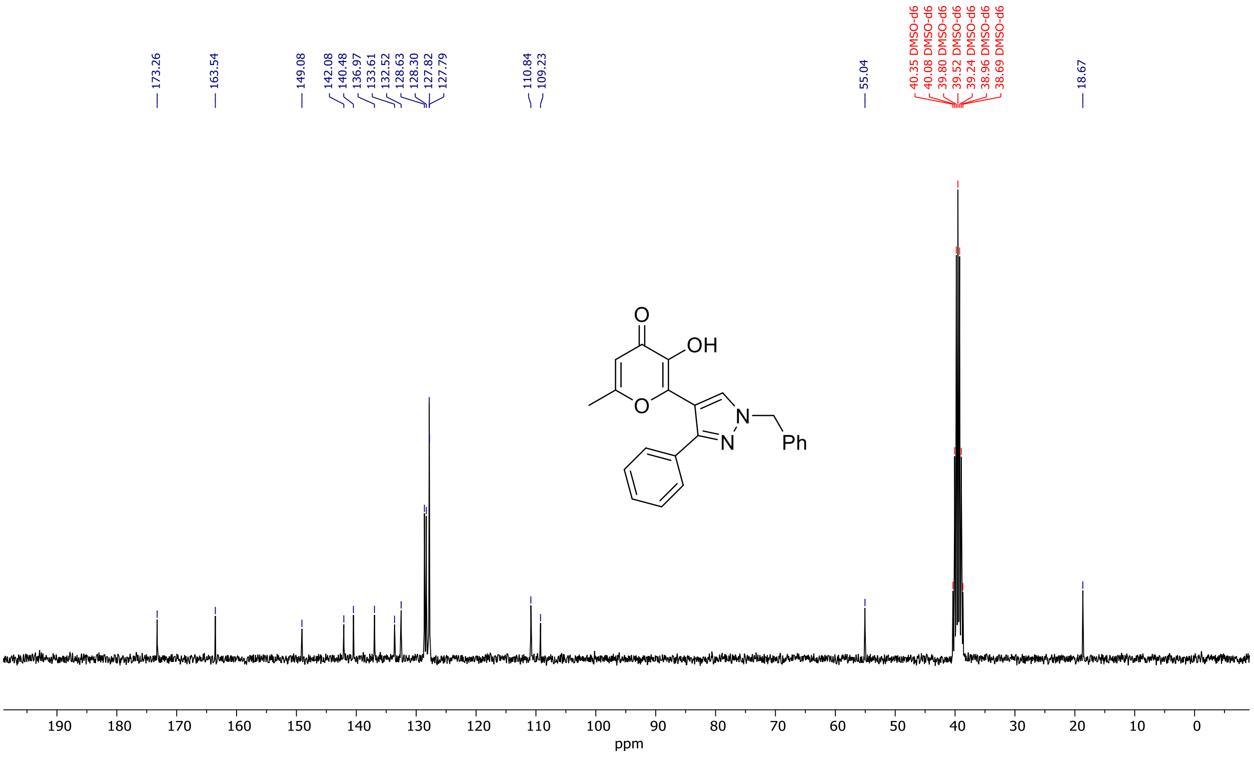

^{1}H NMR spectrum (300 MHz) of **12o** in DMSO-d_6

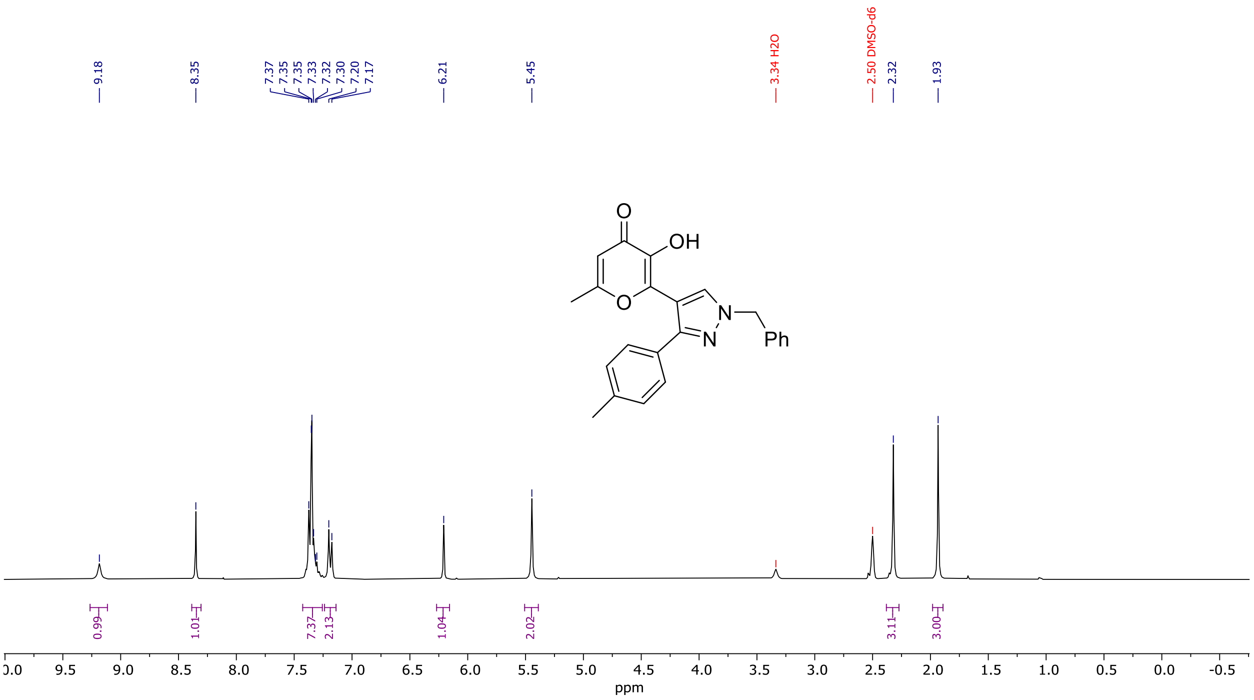

^{13}C {^{1}H} NMR spectrum (75 MHz) of **12o** in DMSO-d_6

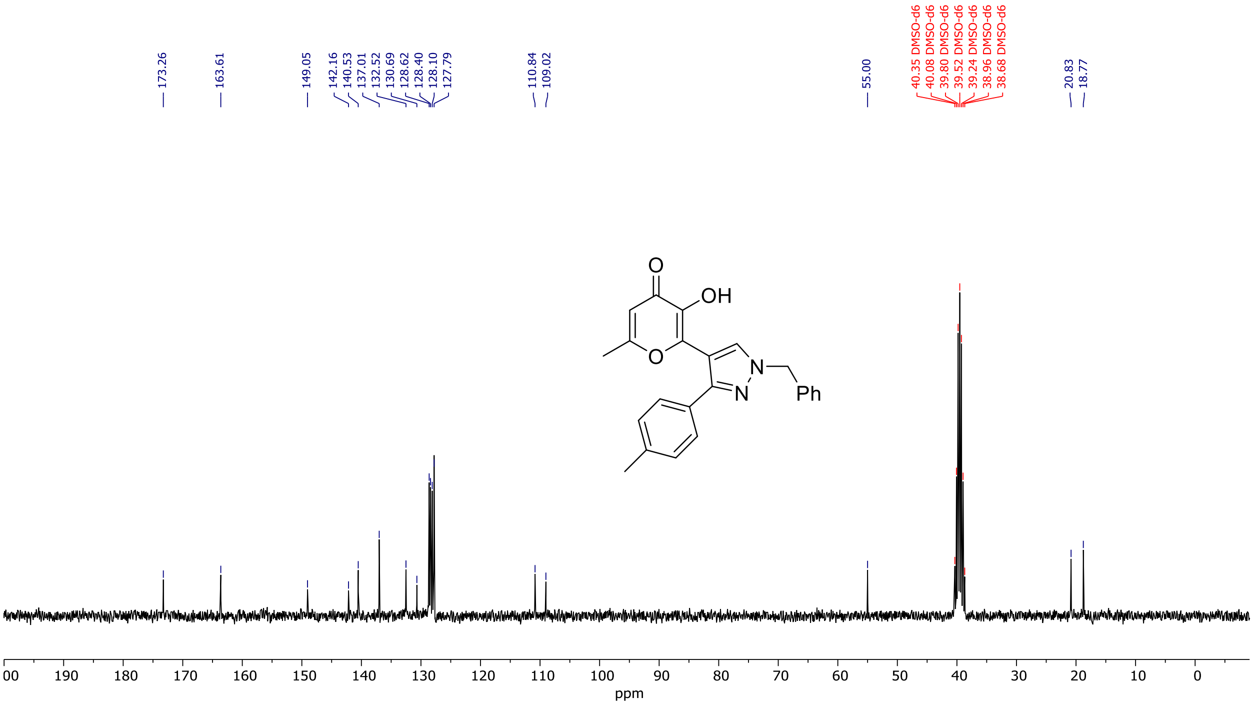

^{1}H NMR spectrum (300 MHz) of **16** in DMSO-d_6

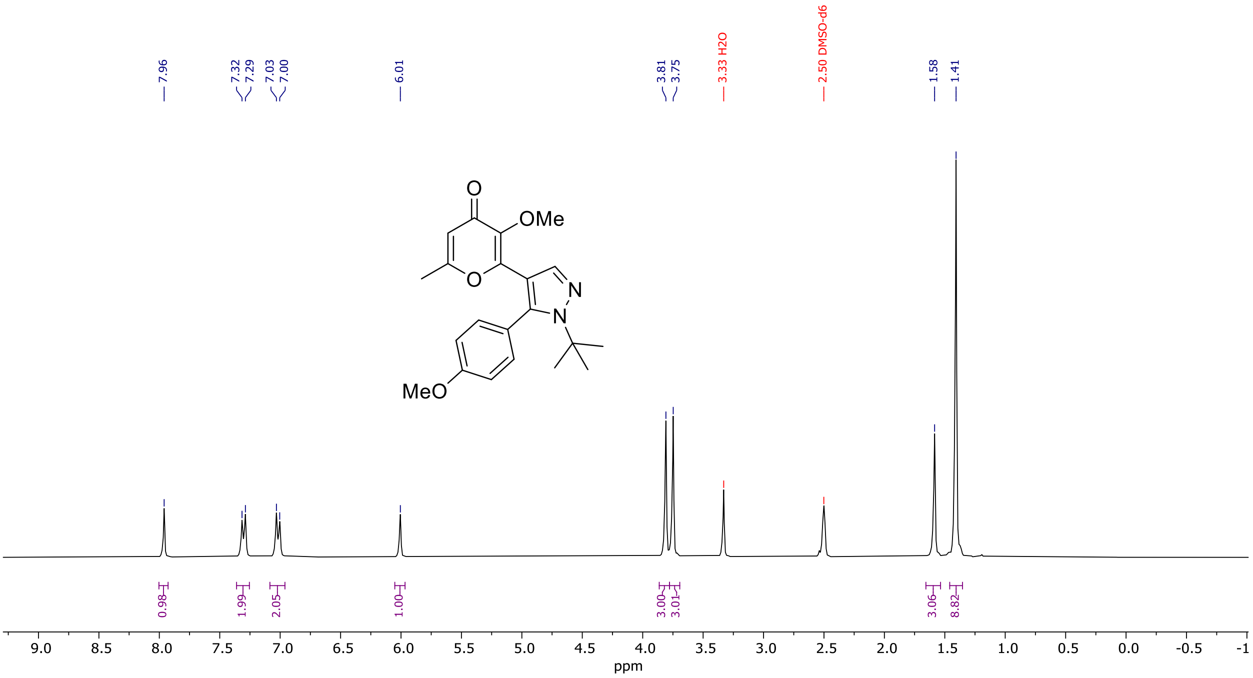

^{13}C {^{1}H} NMR spectrum (75 MHz) of **16** in DMSO-d_6

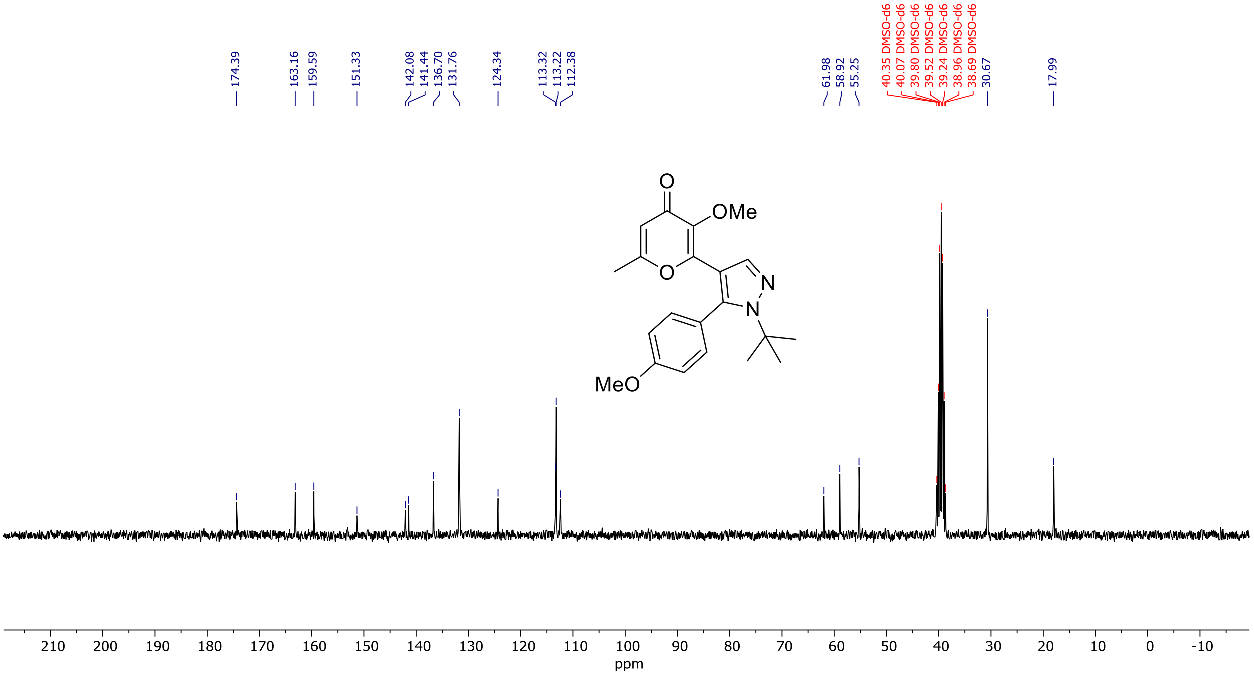

6. Copies of ^{1}H and ^{13}C NMR spectra for compounds **14a**, **15** and **18.**

^{1}H NMR spectrum (300 MHz) of **14a** in DMSO-d_6

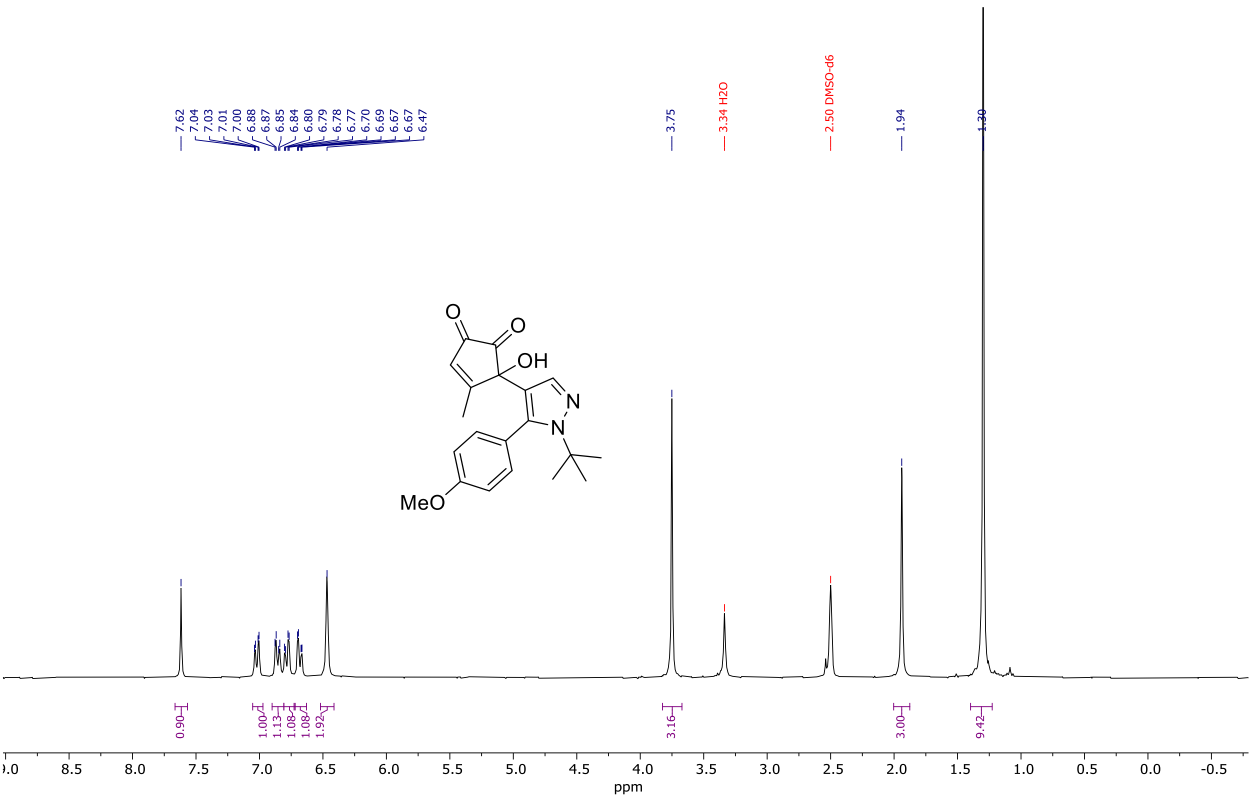

^{13}C {^{1}H} NMR spectrum (75 MHz) of **14a** in DMSO-d_6

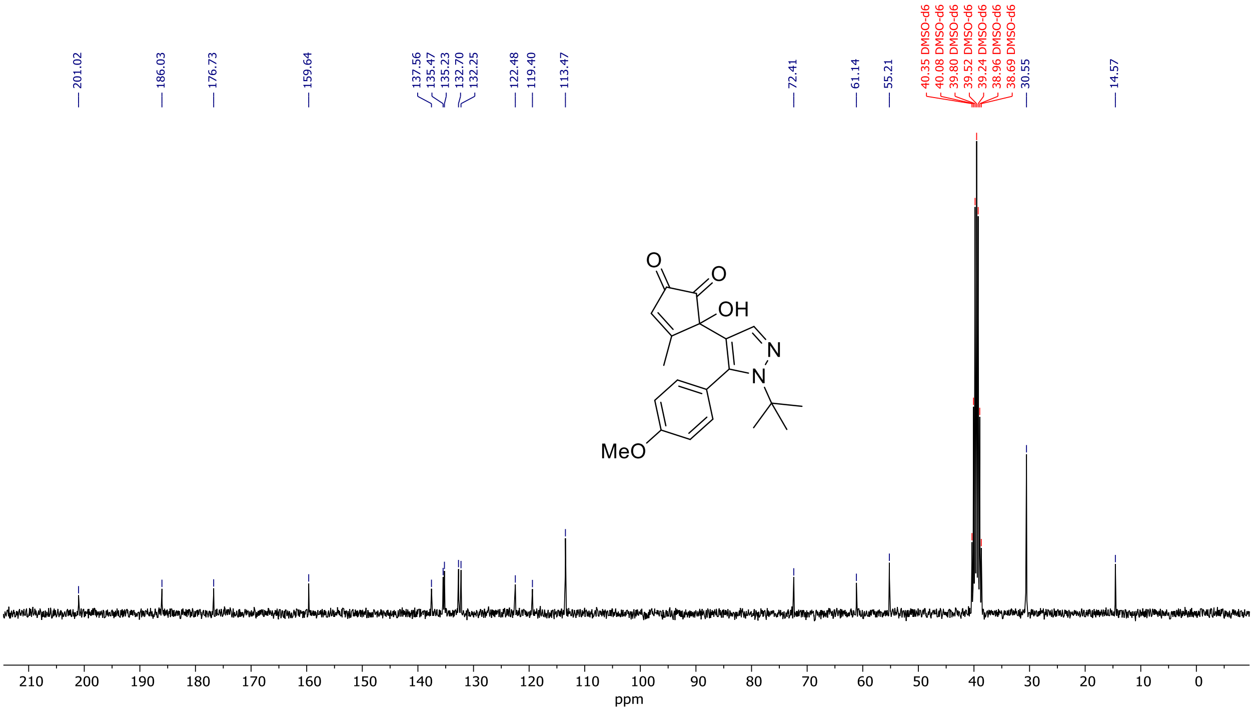

^{1}H NMR spectrum (300 MHz) of **15a** in DMSO-d_6

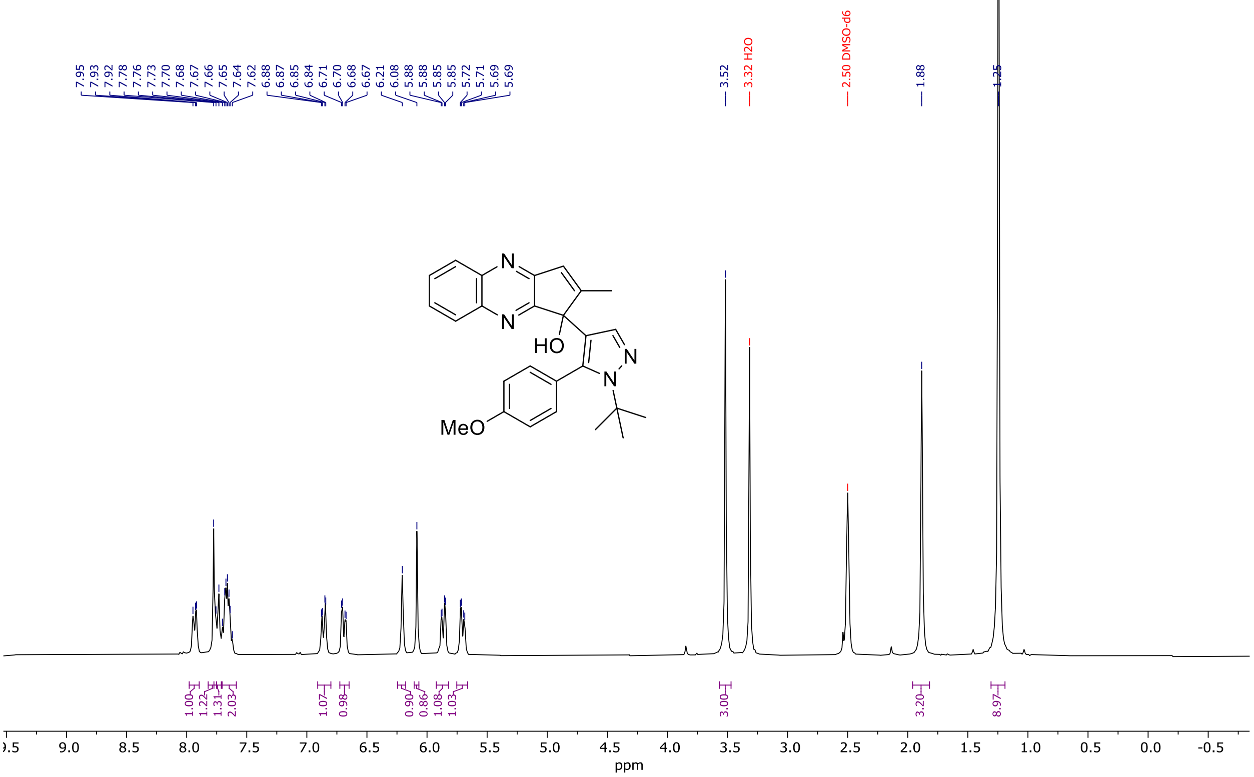

^{13}C {^{1}H} NMR spectrum (75 MHz) of **15a** in DMSO-d_6

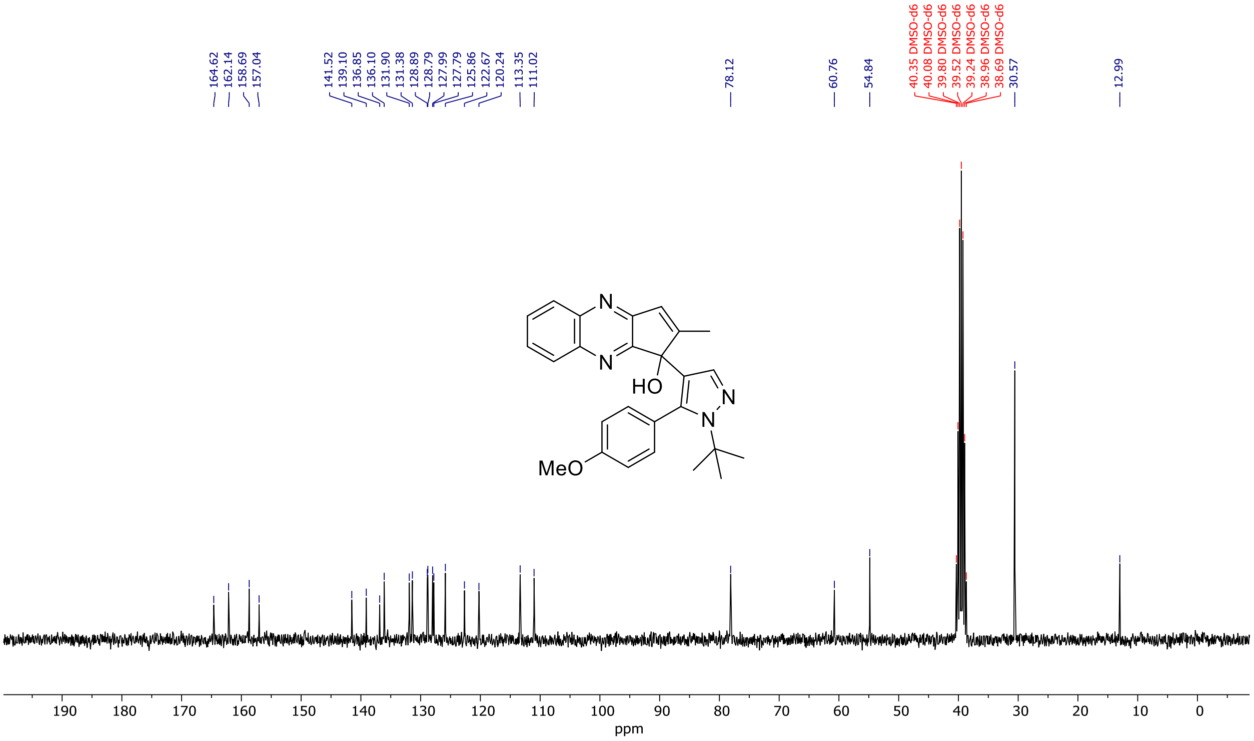

^{1}H NMR spectrum (300 MHz) of **15b** in DMSO-d_6

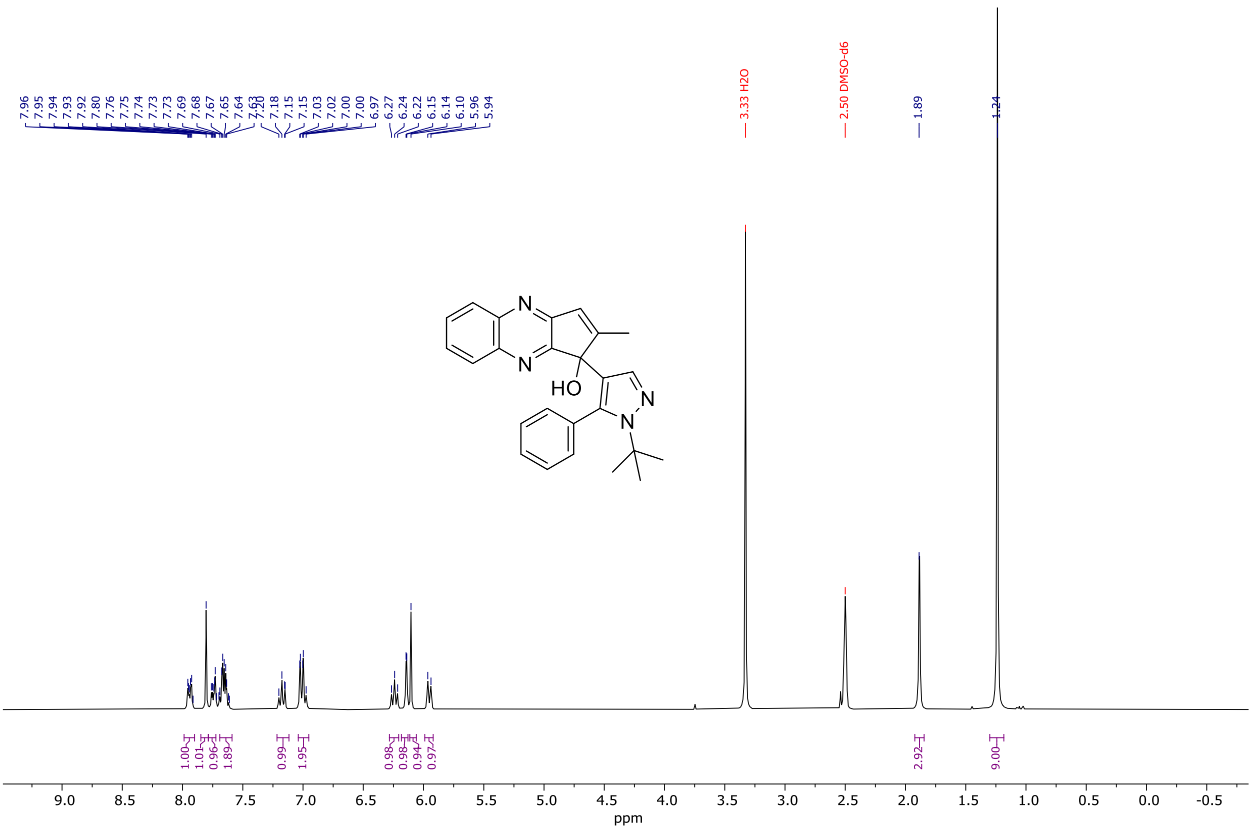

^{13}C {^{1}H} NMR spectrum (75 MHz) of **15b** in DMSO-d_6

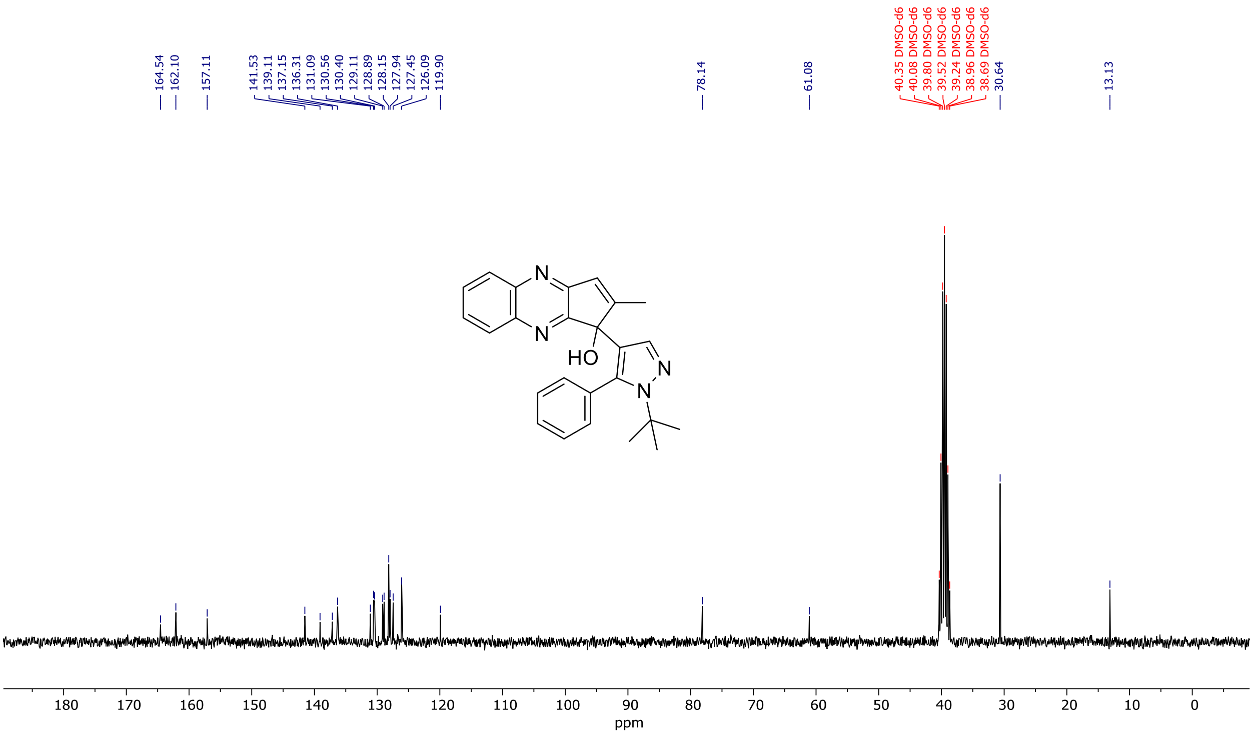

^1H NMR spectrum (300 MHz) of **15c** in DMSO-d_6

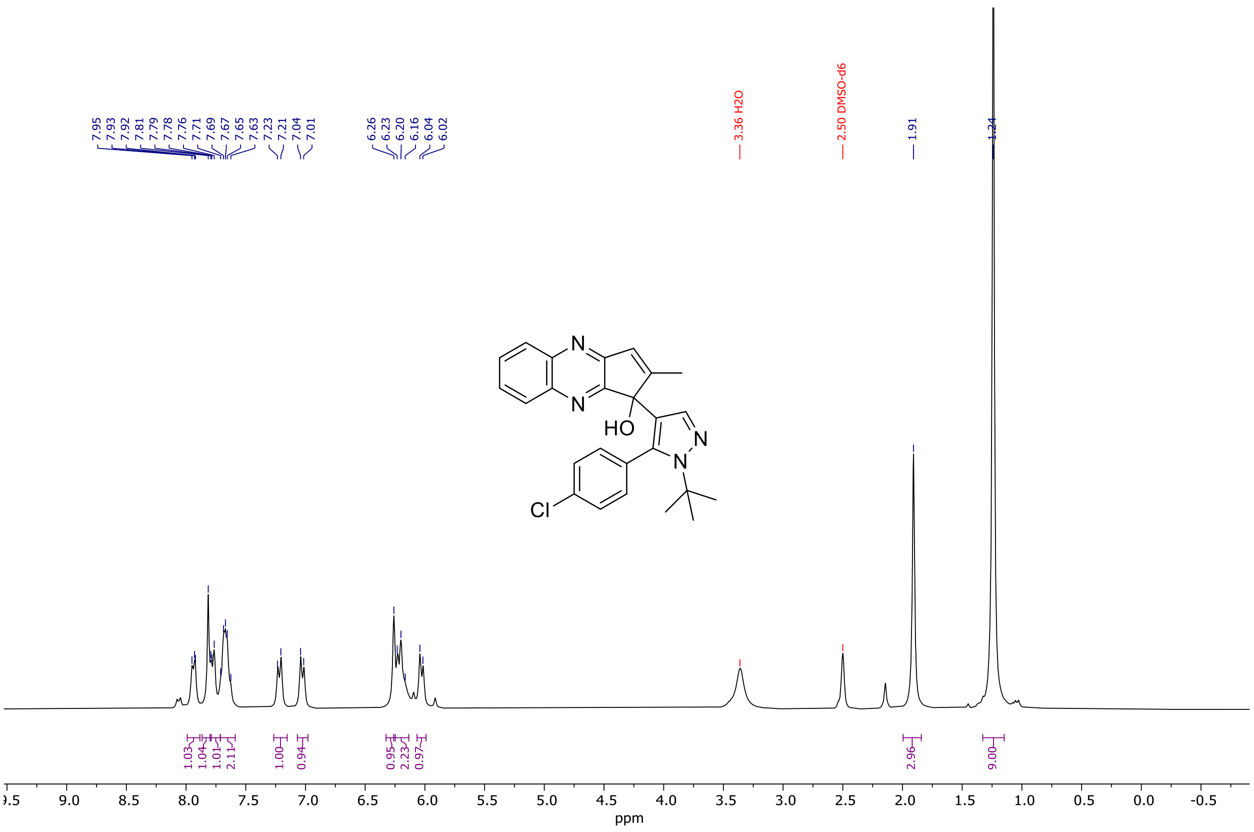

^{13}C {^1H} NMR spectrum (75 MHz) of **15c** in DMSO-d_6

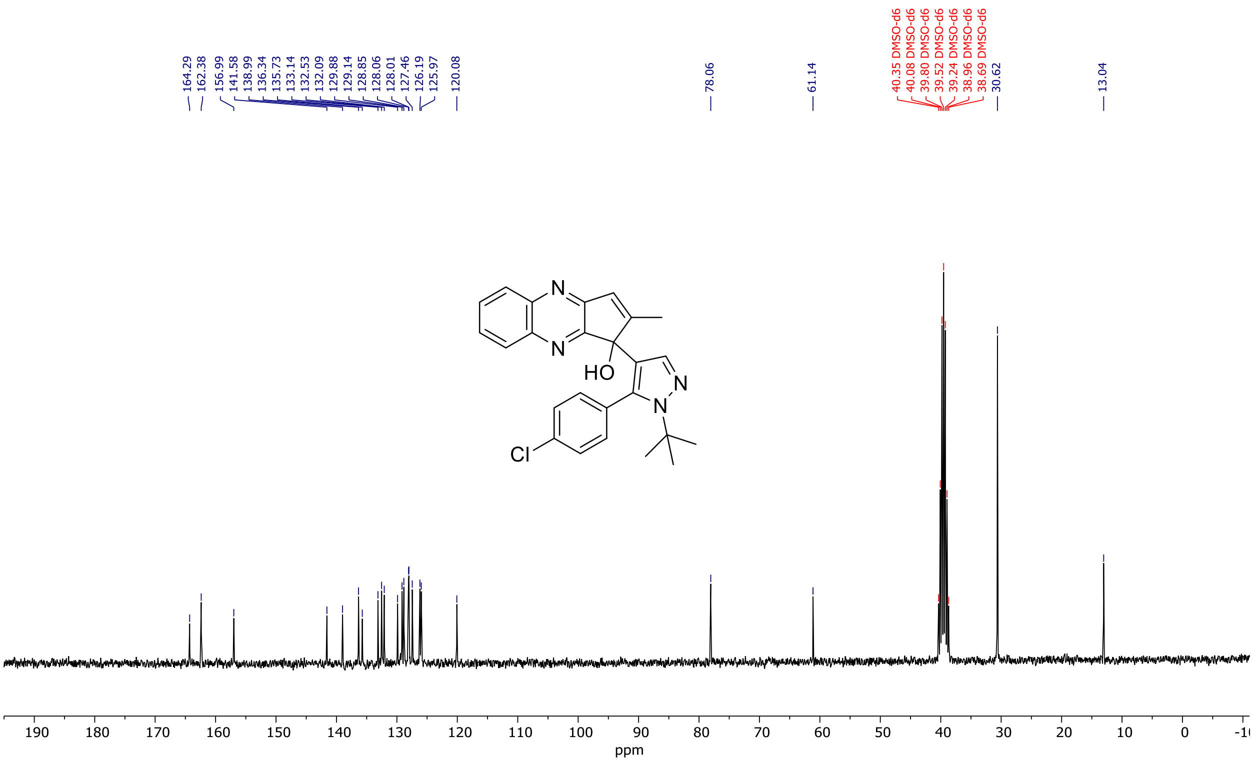

^{1}H NMR spectrum (300 MHz) of **15d** in DMSO-d_6

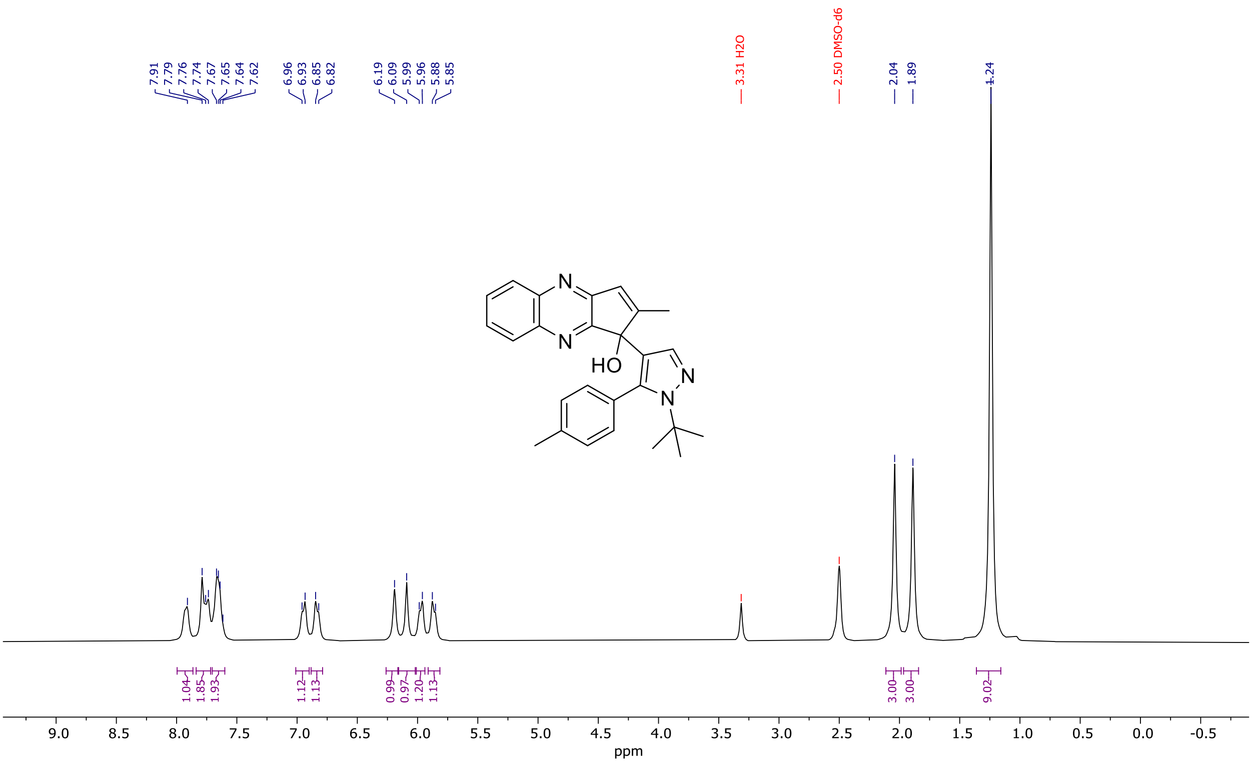

^{13}C {^{1}H} NMR spectrum (75 MHz) of **15d** in DMSO-d_6

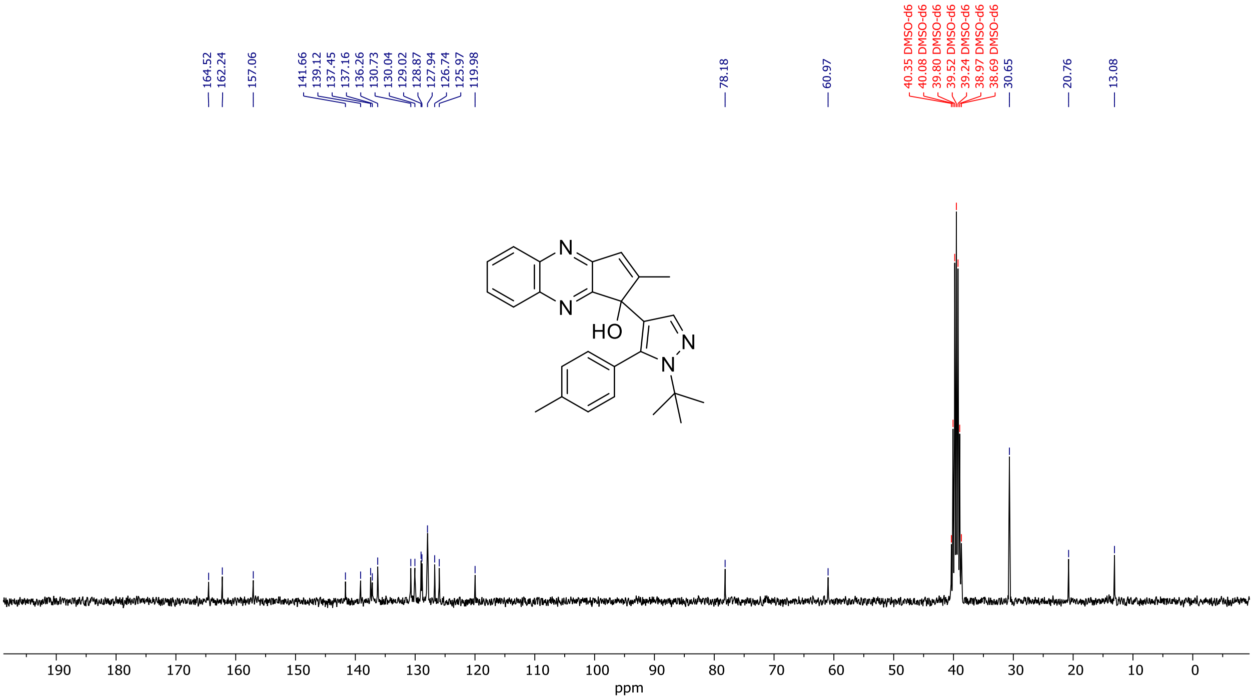

^{1}H NMR spectrum (300 MHz) of **15e** in DMSO-d_6

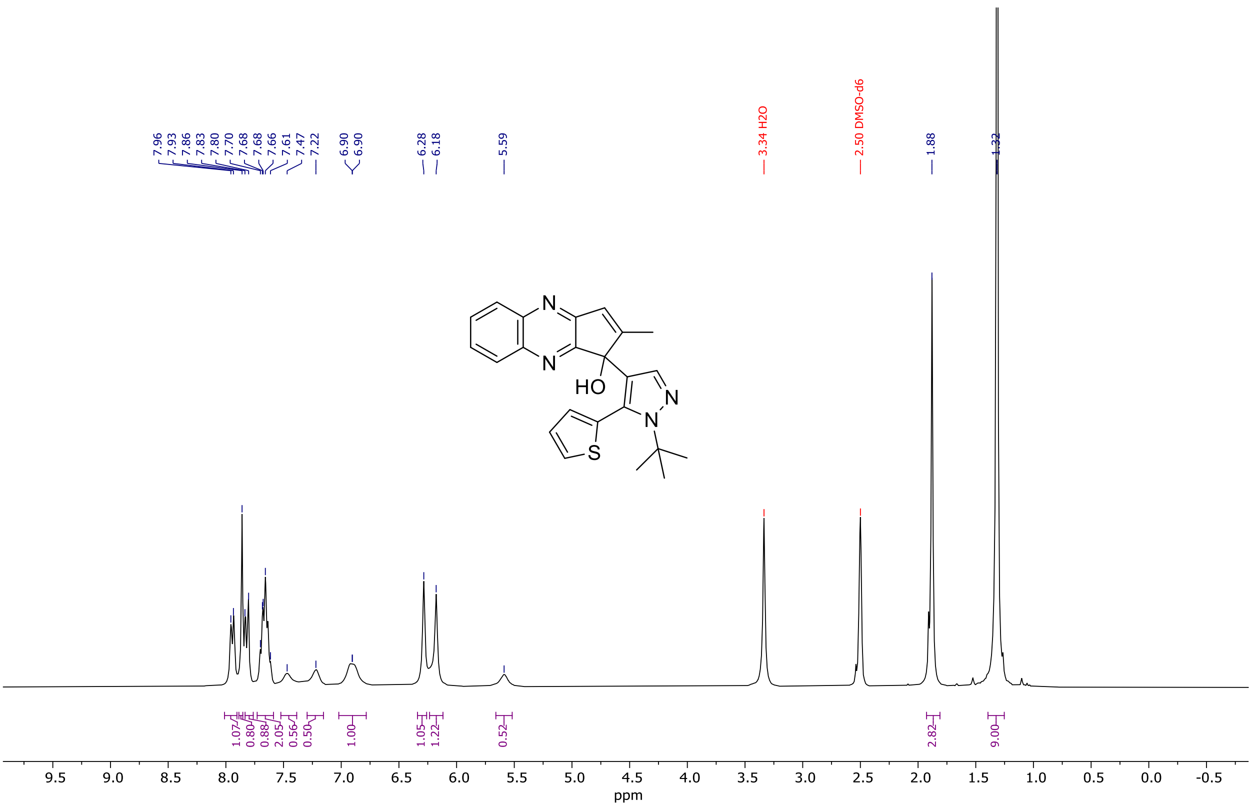

^{13}C {^{1}H} NMR spectrum (75 MHz) of **15e** in DMSO-d_6

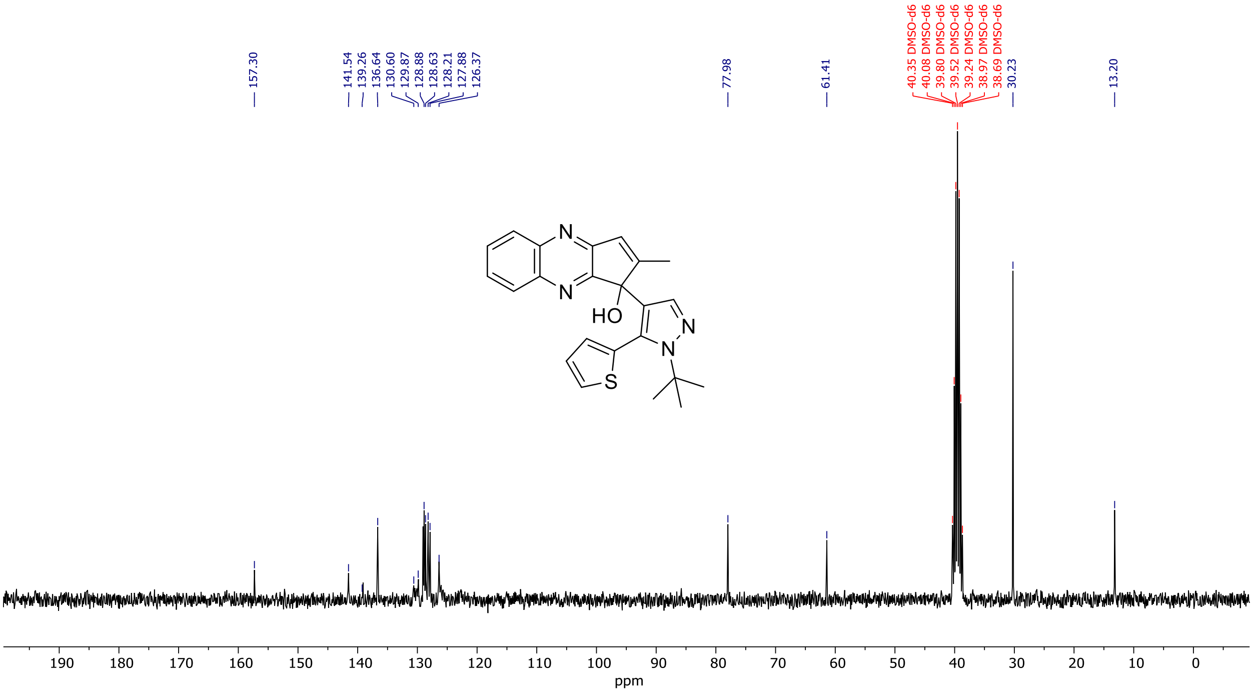

^{1}H NMR spectrum (300 MHz) of **15f** in DMSO-d_6

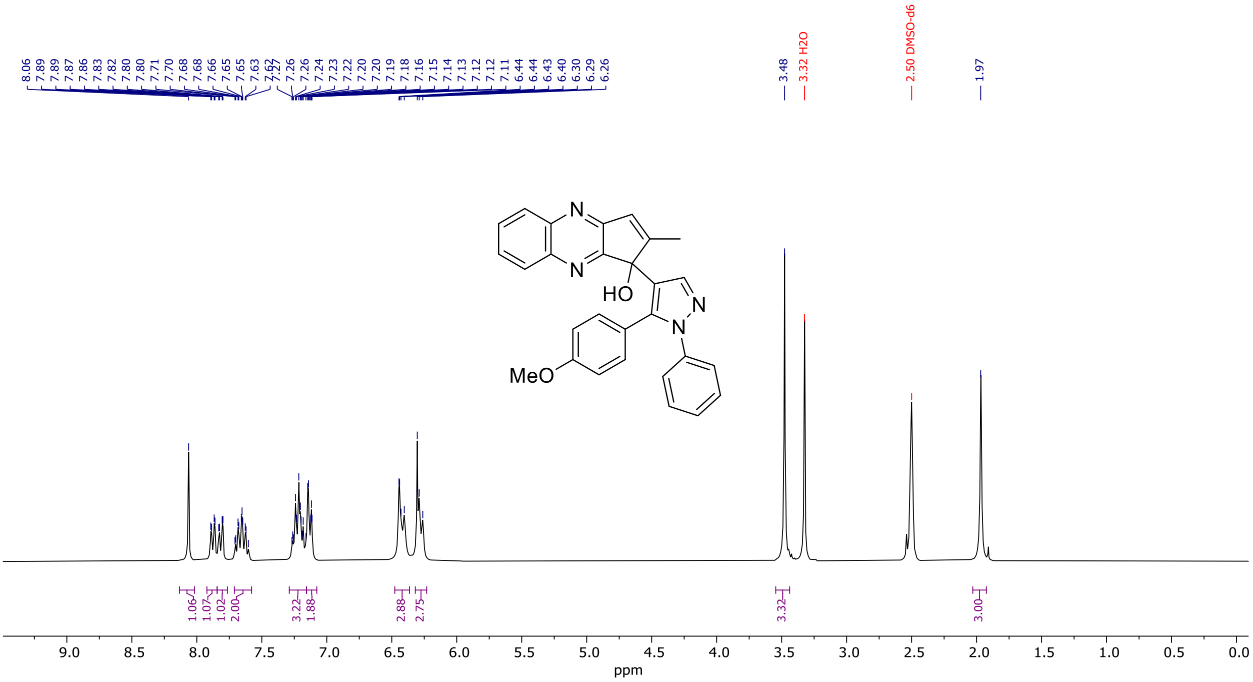

^{13}C {^{1}H} NMR spectrum (75 MHz) of **15f** in DMSO-d_6

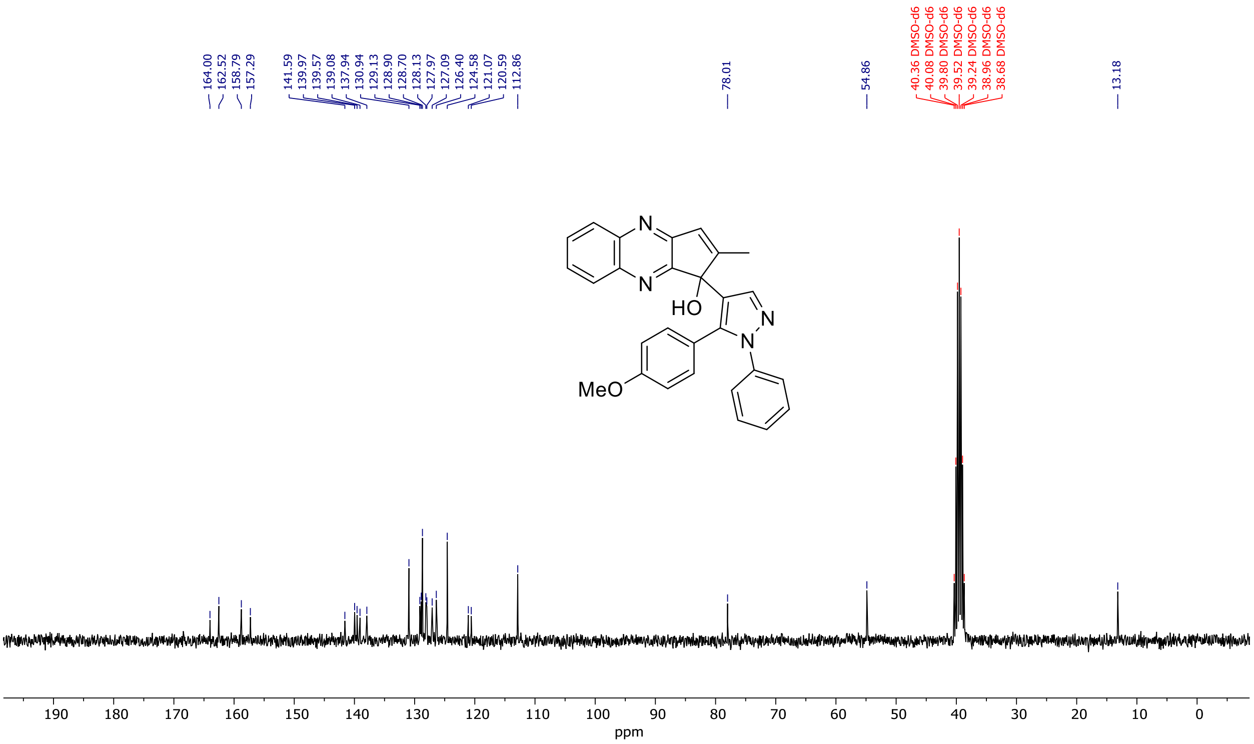

^{1}H NMR spectrum (300 MHz) of **15g** in DMSO-d_6

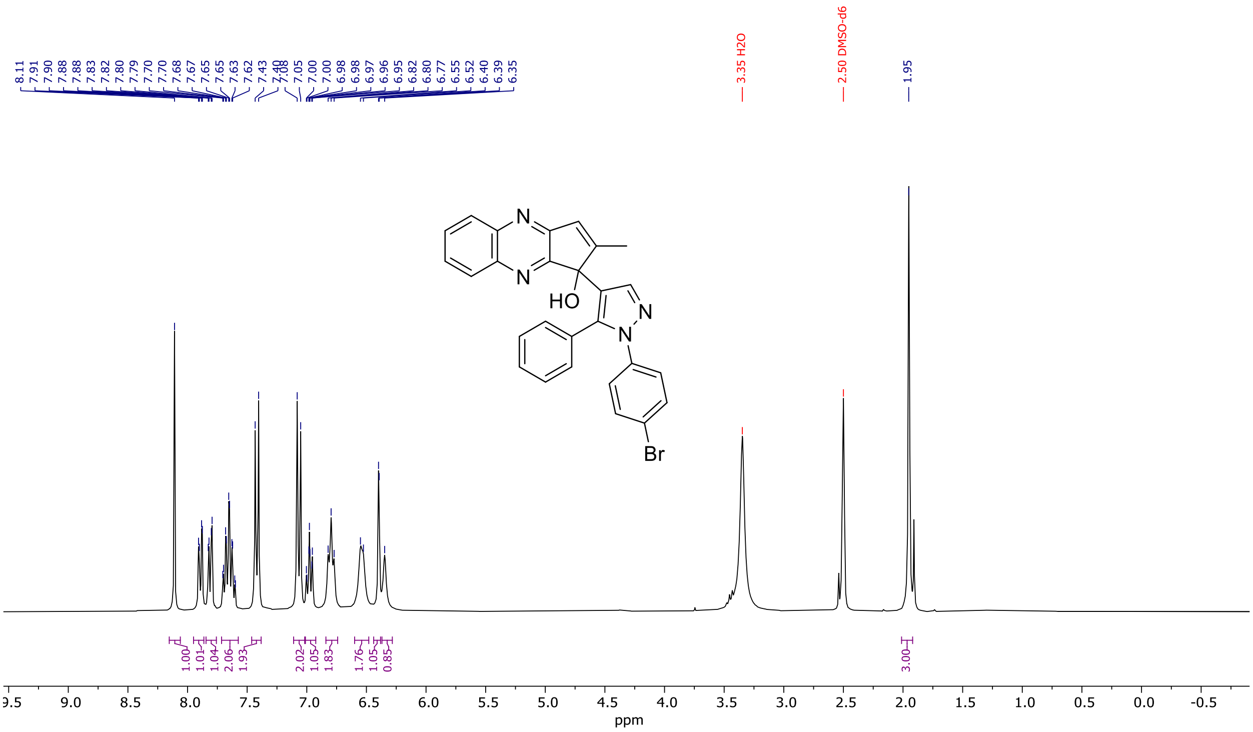

^{13}C {^{1}H} NMR spectrum (75 MHz) of **15g** in DMSO-d_6

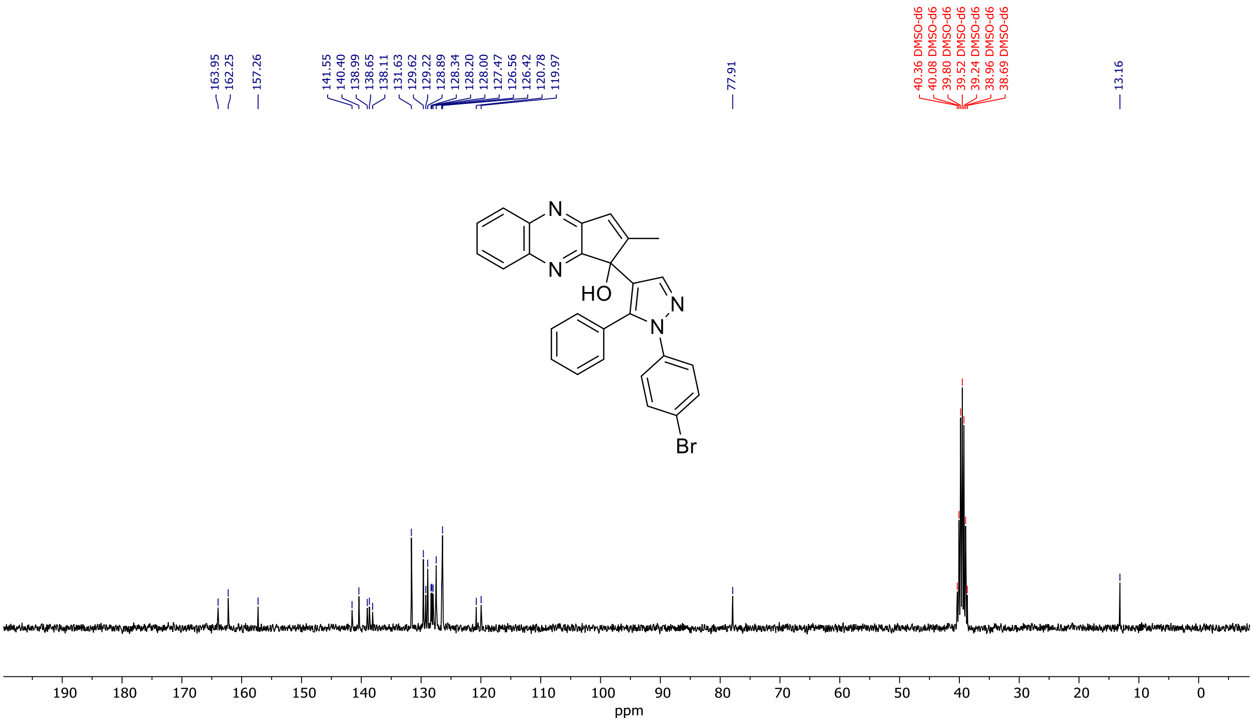

^{1}H NMR spectrum (300 MHz) of **15h** in DMSO-d_6

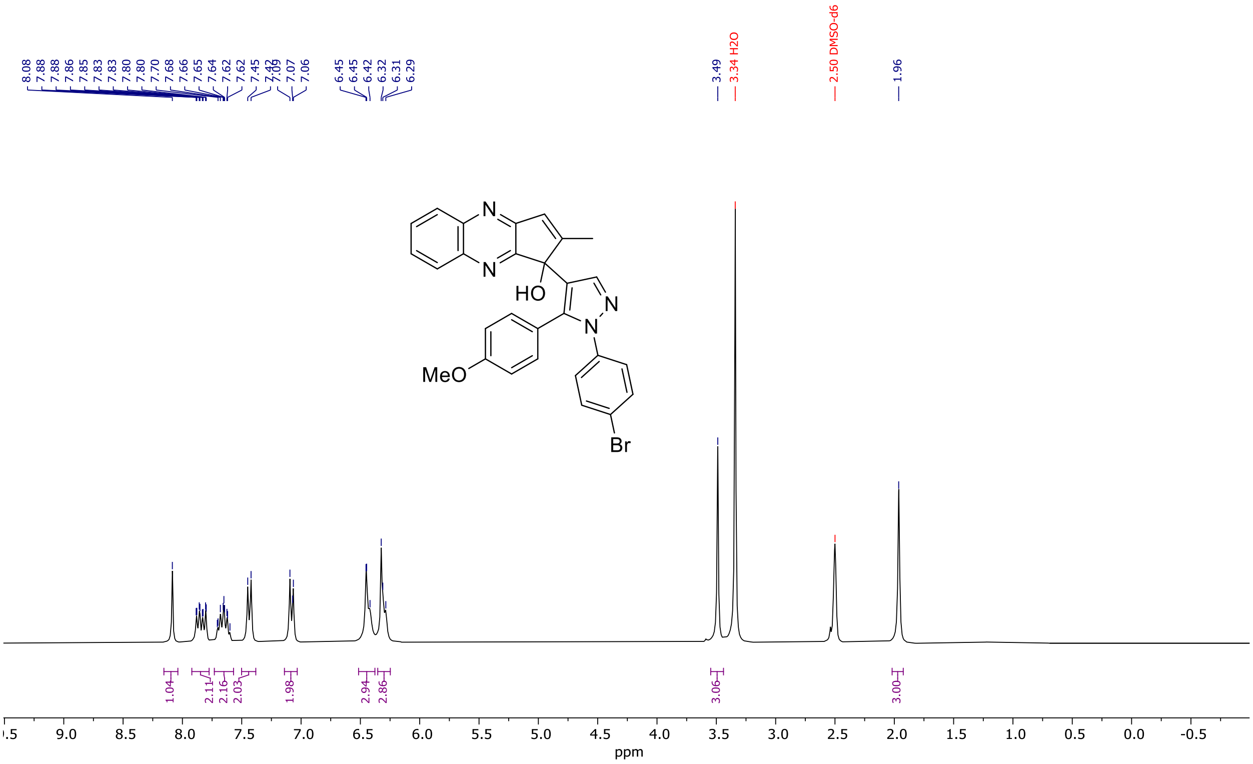

$^{13}C\ \{^{1}H\}$ NMR spectrum (75 MHz) of **15h** in DMSO-d_6

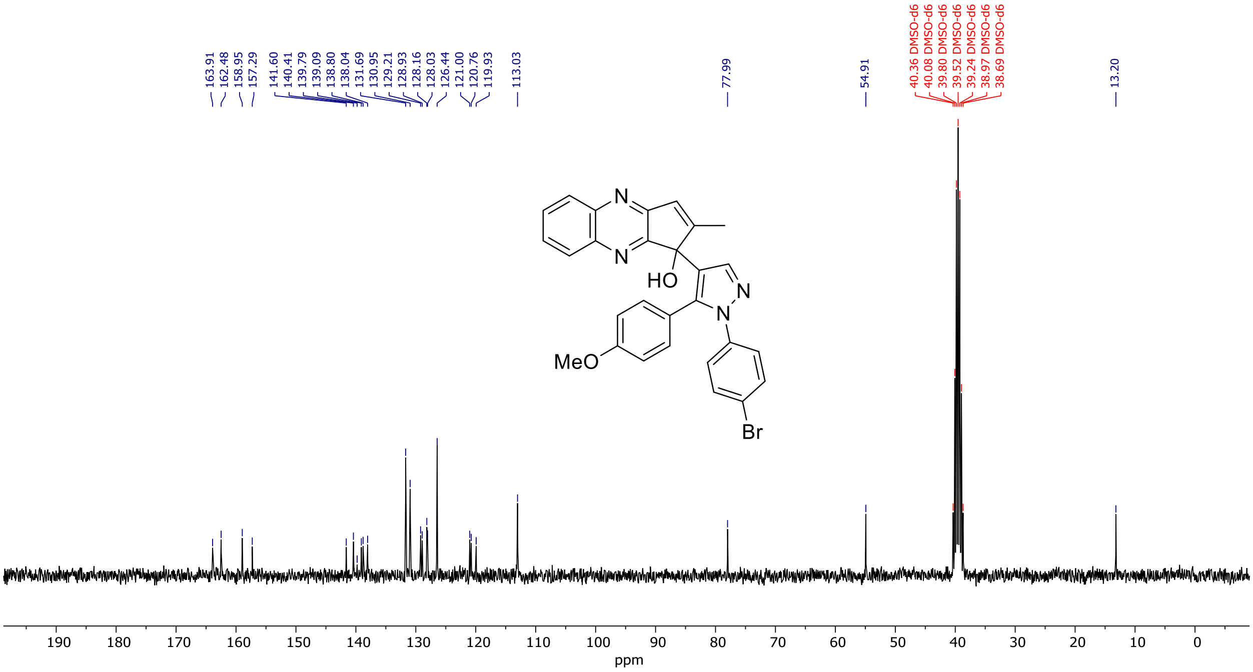

^{1}H NMR spectrum (300 MHz) of **15i** in DMSO-d_6

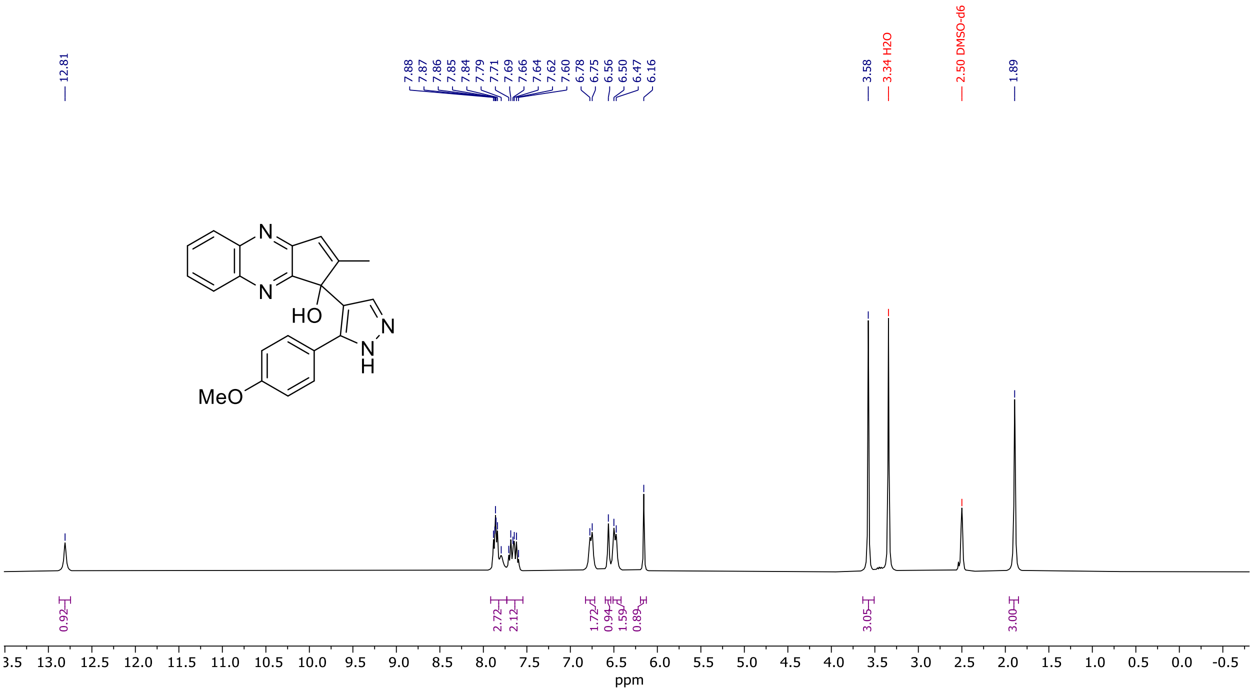

^{13}C {^{1}H} NMR spectrum (75 MHz) of **15i** in DMSO-d_6

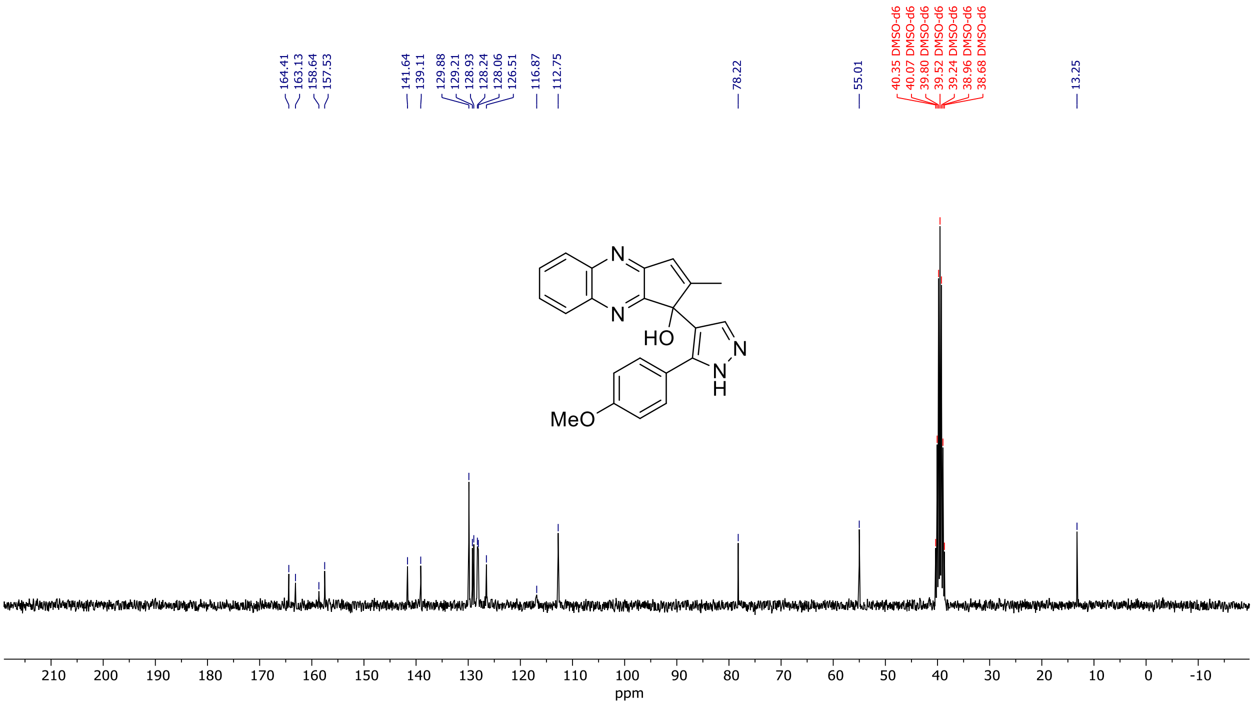

^{1}H NMR spectrum (300 MHz) of **15j** in DMSO-d_6

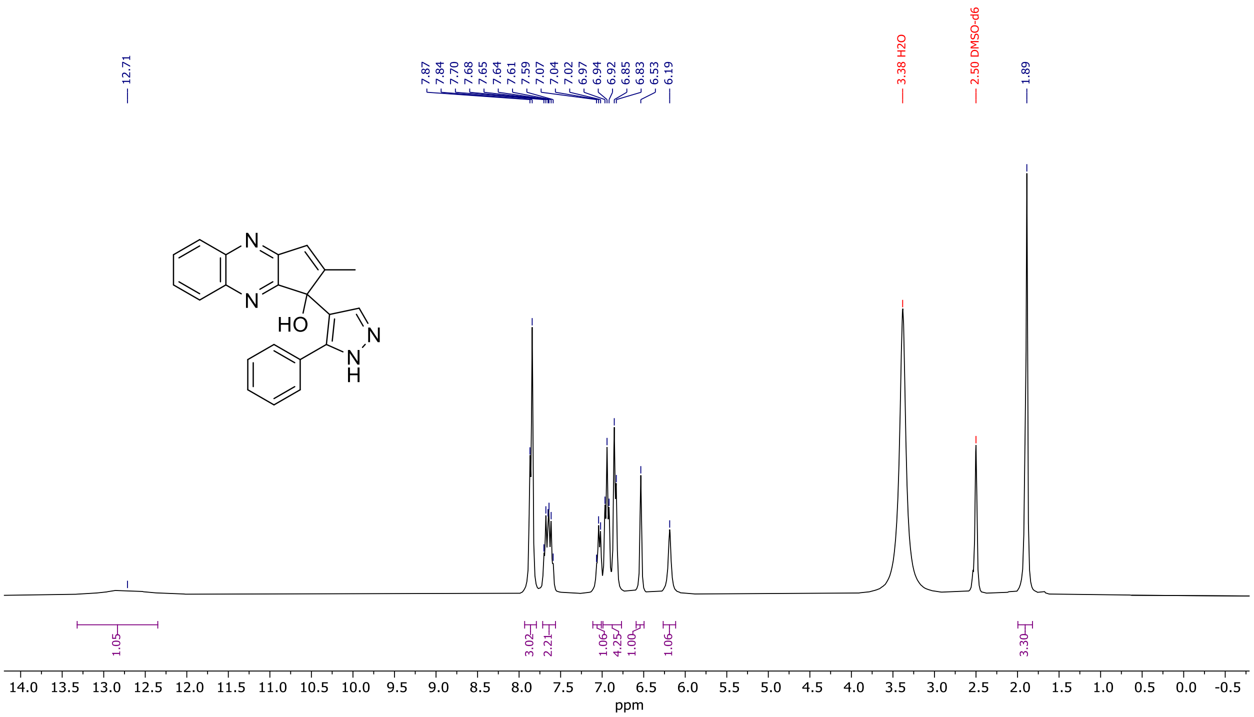

^{13}C {^{1}H} NMR spectrum (75 MHz) of **15j** in DMSO-d_6

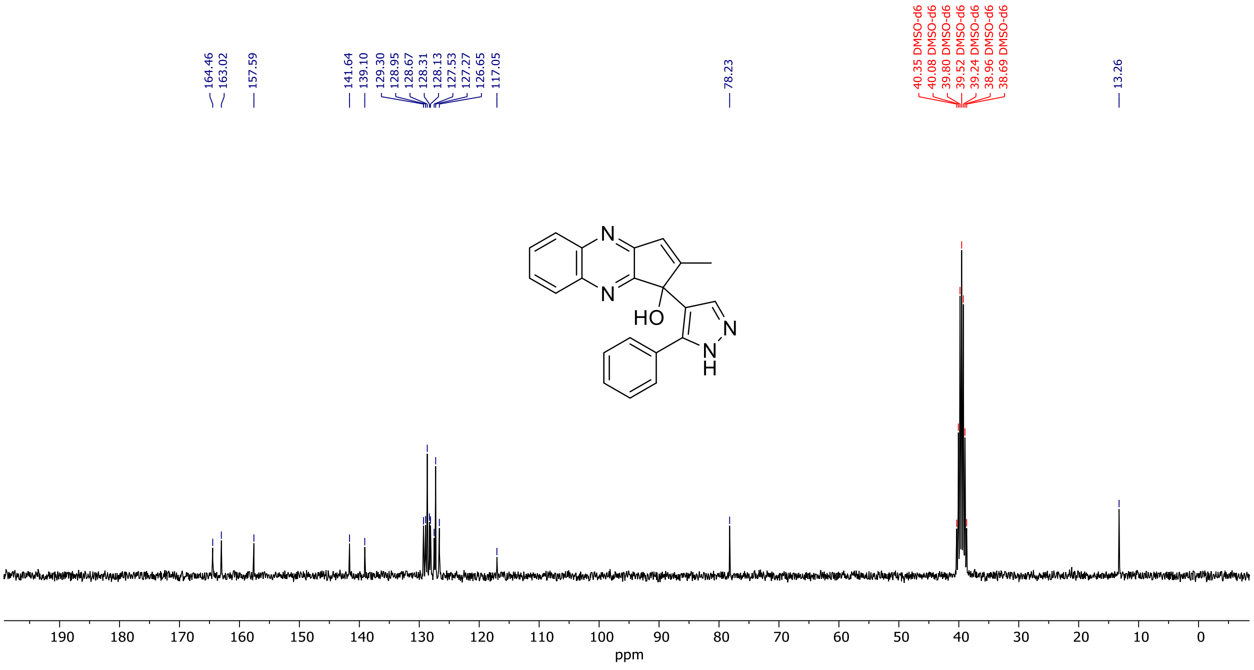

^{1}H NMR spectrum (300 MHz) of **15k** in DMSO-d_6

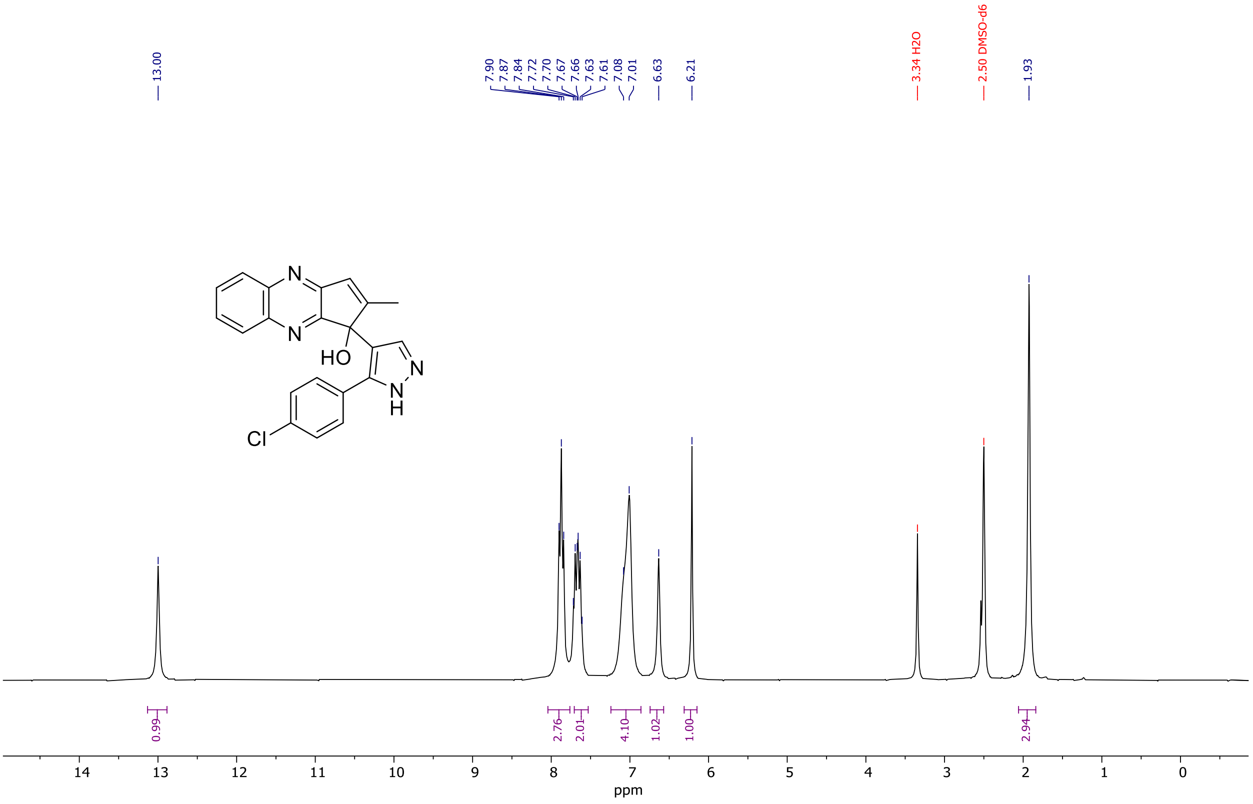

^{13}C {^{1}H} NMR spectrum (75 MHz) of **15k** in DMSO-d_6

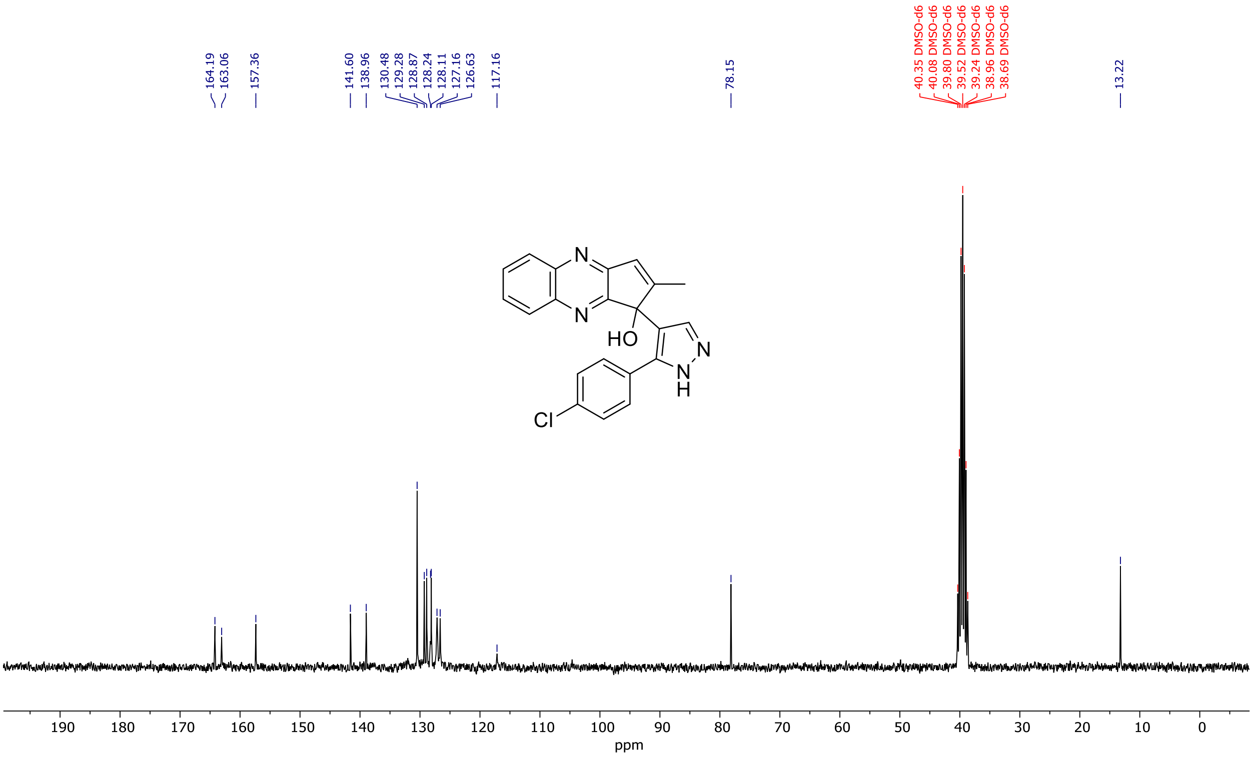

^{1}H NMR spectrum (300 MHz) of **15l** in DMSO-d_6

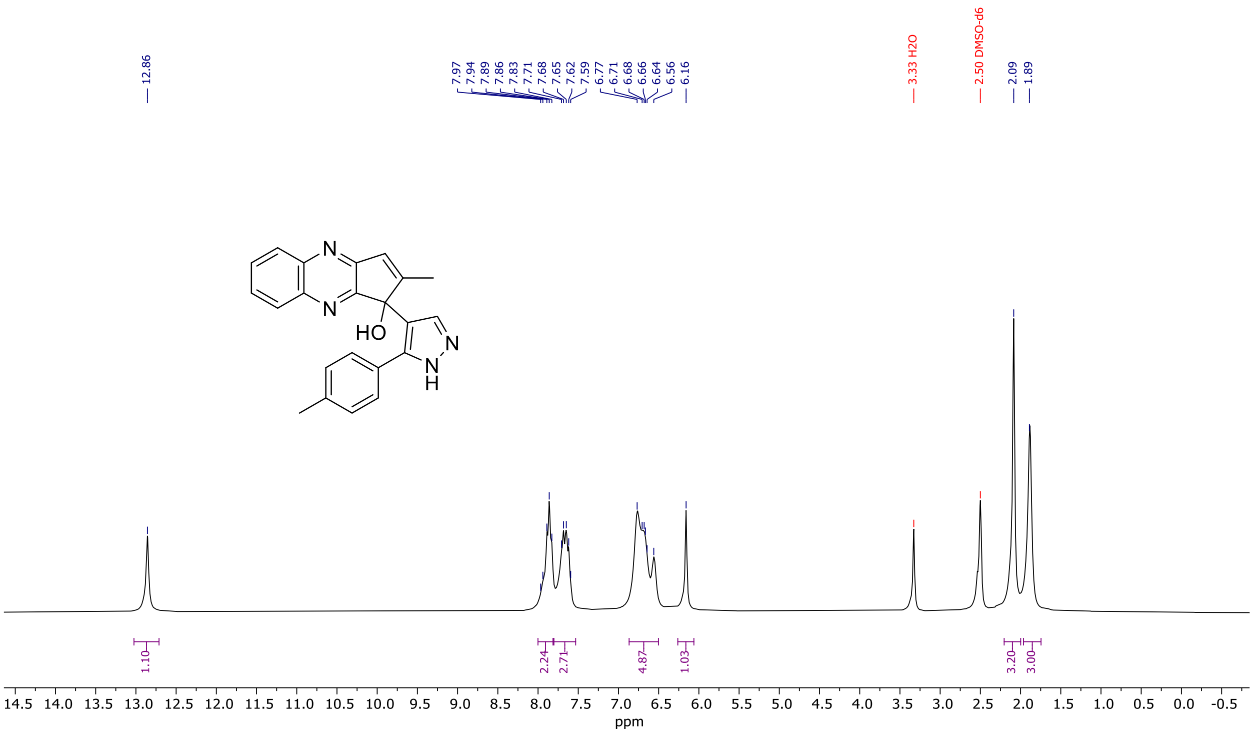

^{13}C {^{1}H} NMR spectrum (75 MHz) of **15l** in DMSO-d_6

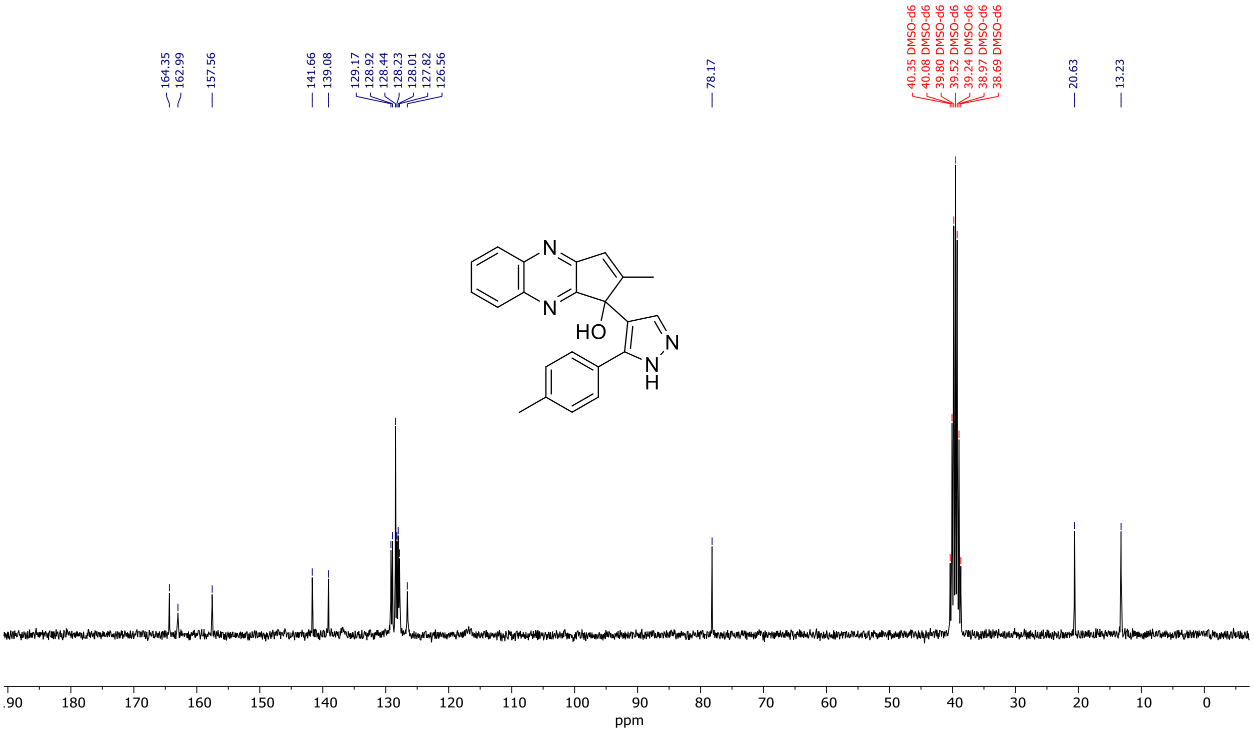

^{1}H NMR spectrum (300 MHz) of **15m** in DMSO-d_6

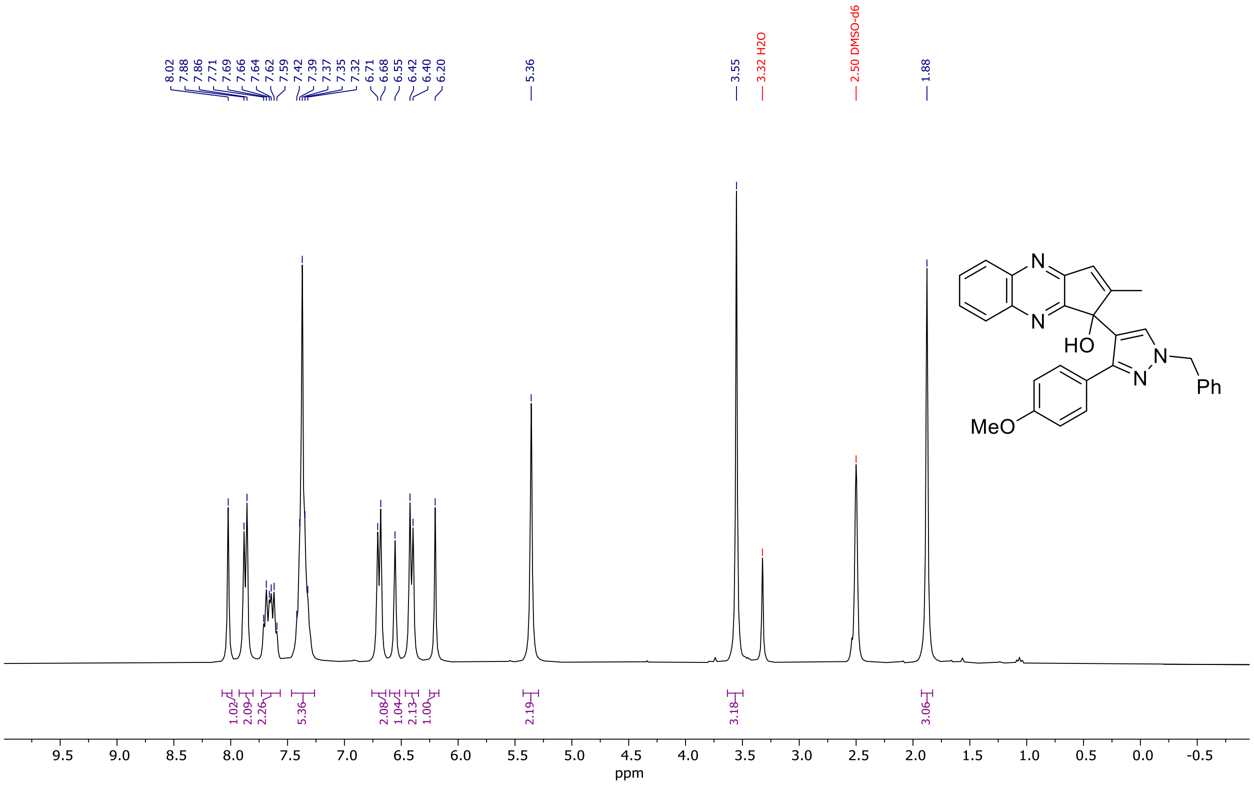

^{13}C {^{1}H} NMR spectrum (75 MHz) of **15m** in DMSO-d_6

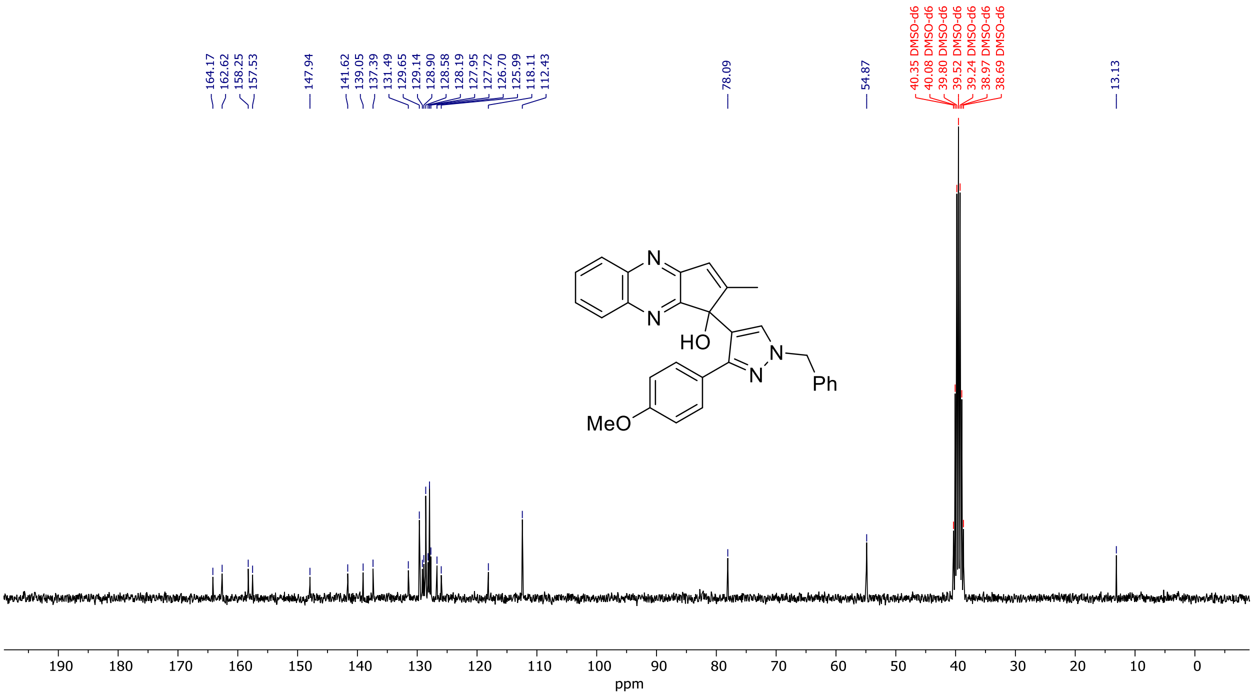

^{1}H NMR spectrum (300 MHz) of **15n** in DMSO-d_6

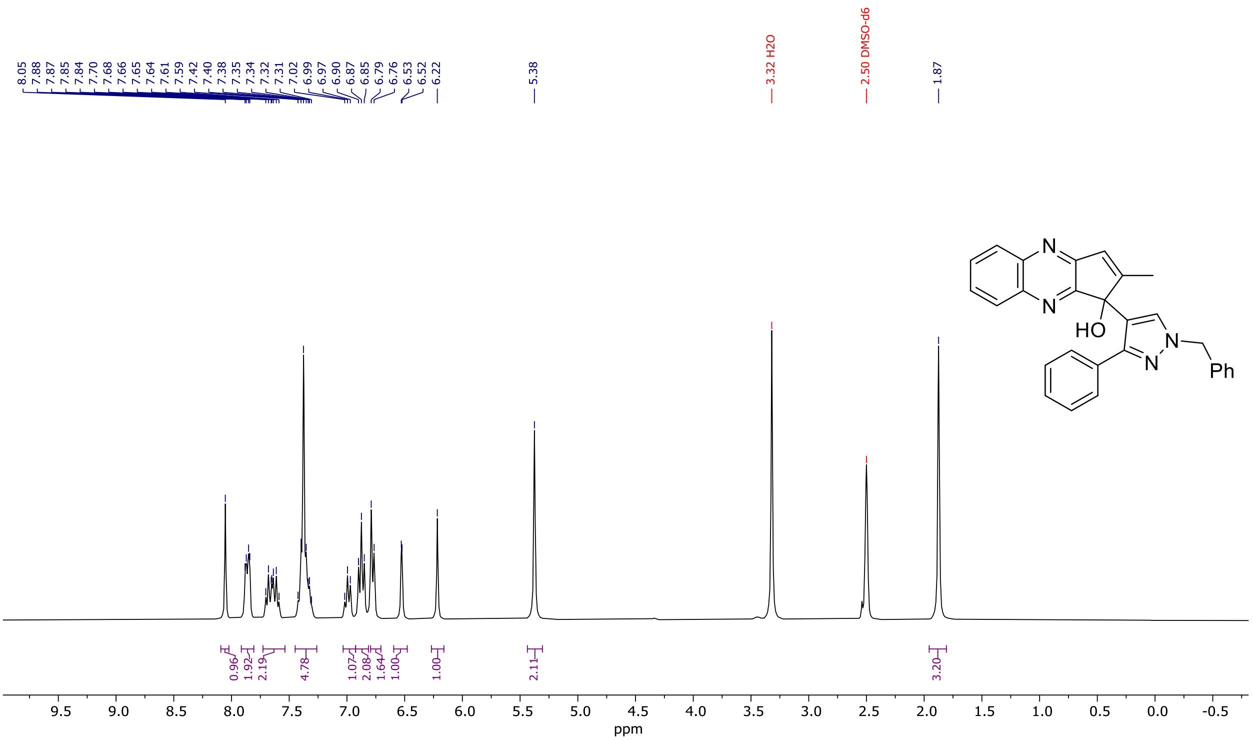

^{13}C {^{1}H} NMR spectrum (75 MHz) of **15n** in DMSO-d_6

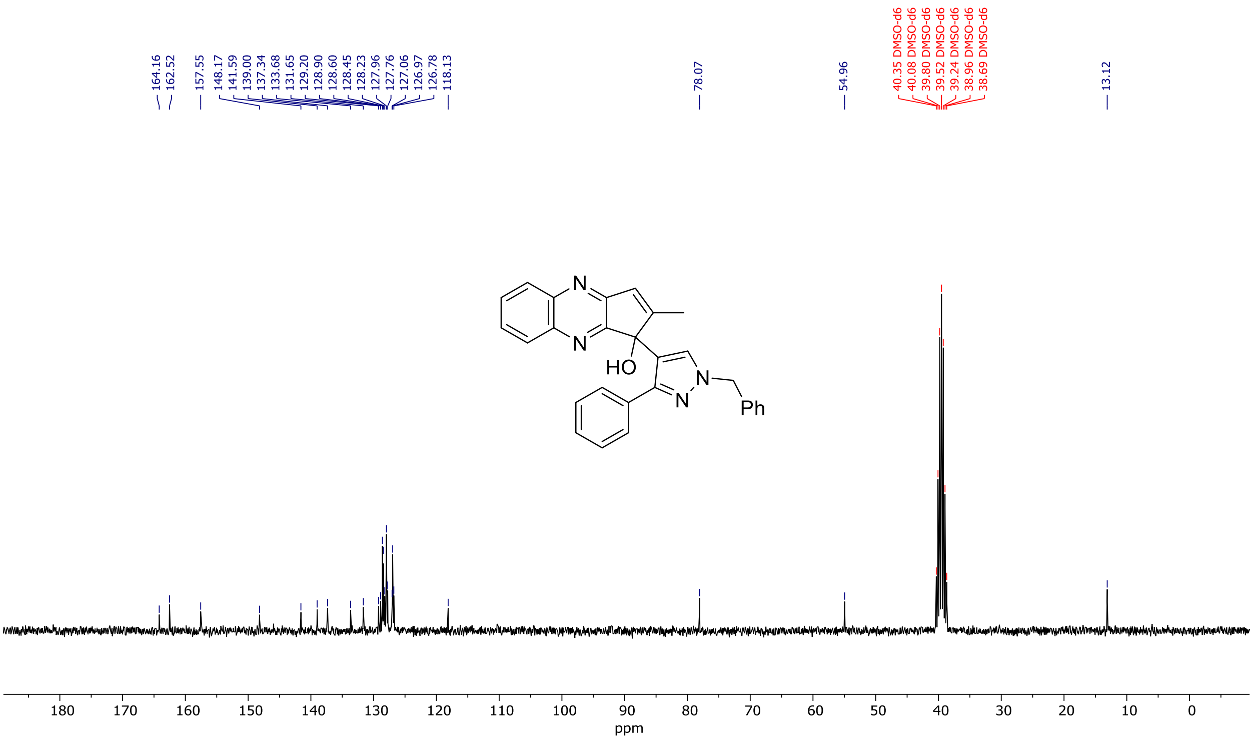

^{1}H NMR spectrum (300 MHz) of **15o** in DMSO-d_6

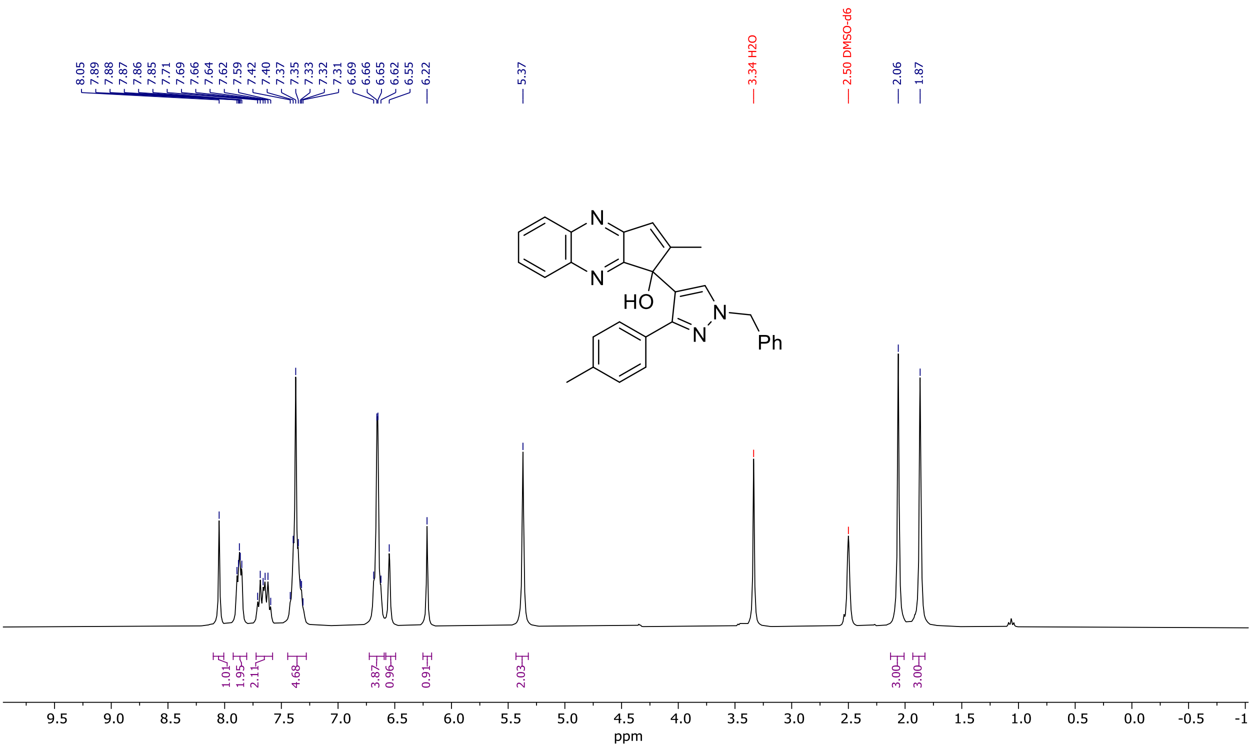

^{13}C {^{1}H} NMR spectrum (75 MHz) of **15o** in DMSO-d_6

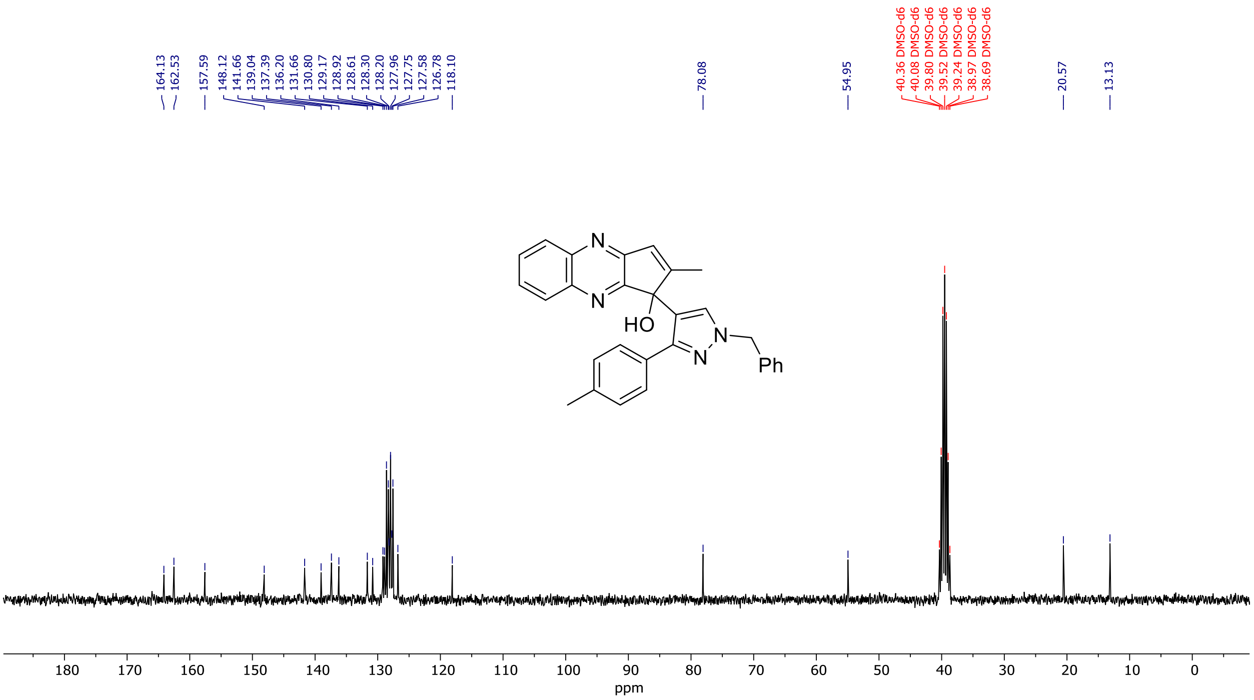

^{1}H NMR spectrum (300 MHz) of **18** in DMSO-d_6

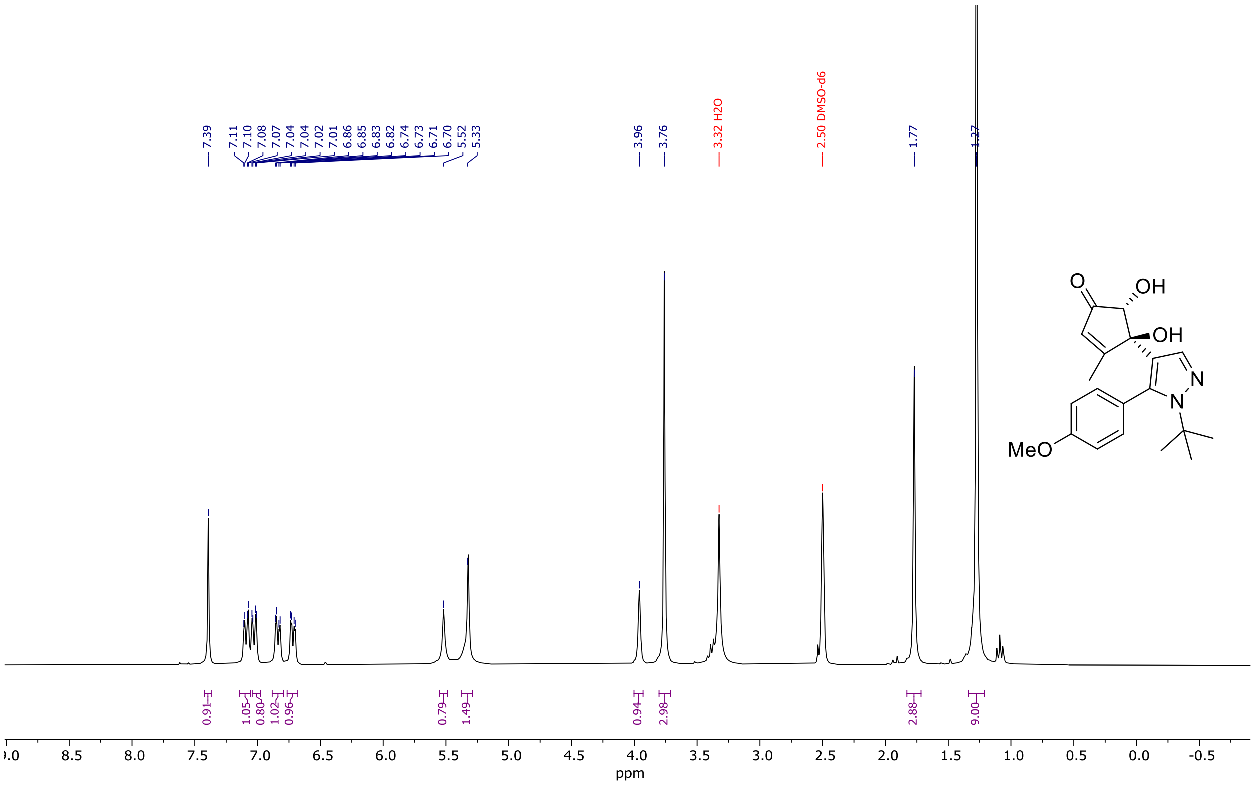

^{13}C {^{1}H} NMR spectrum (75 MHz) of **18** in DMSO-d_6

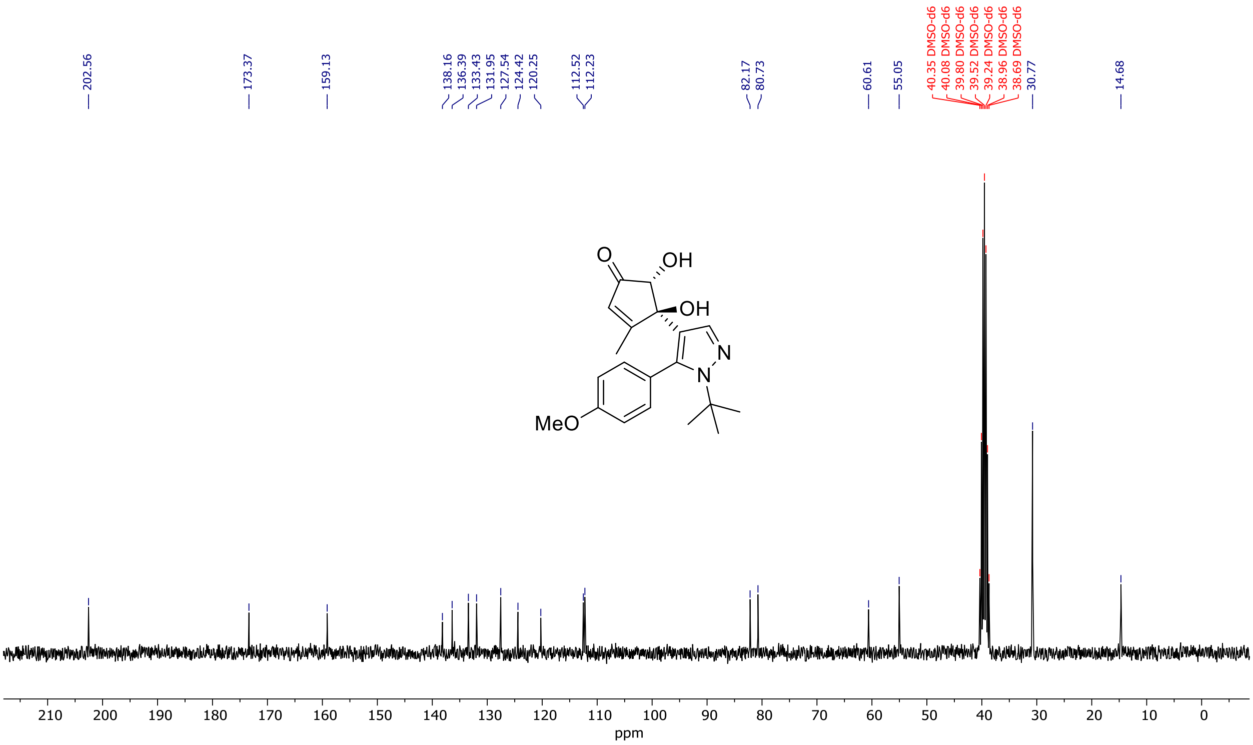

7. Copies of HRMS for all compounds.

HRMS for compound **19c**

Acquisition Parameter

Source Type	ESI	Ion Polarity	Positive	Set Nebulizer	0.4 Bar
Focus	Not active			Set Dry Heater	180 °C
Scan Begin	50 m/z	Set Capillary	4500 V	Set Dry Gas	4.0 l/min
Scan End	1600 m/z	Set End Plate Offset	-500 V	Set Divert Valve	Waste

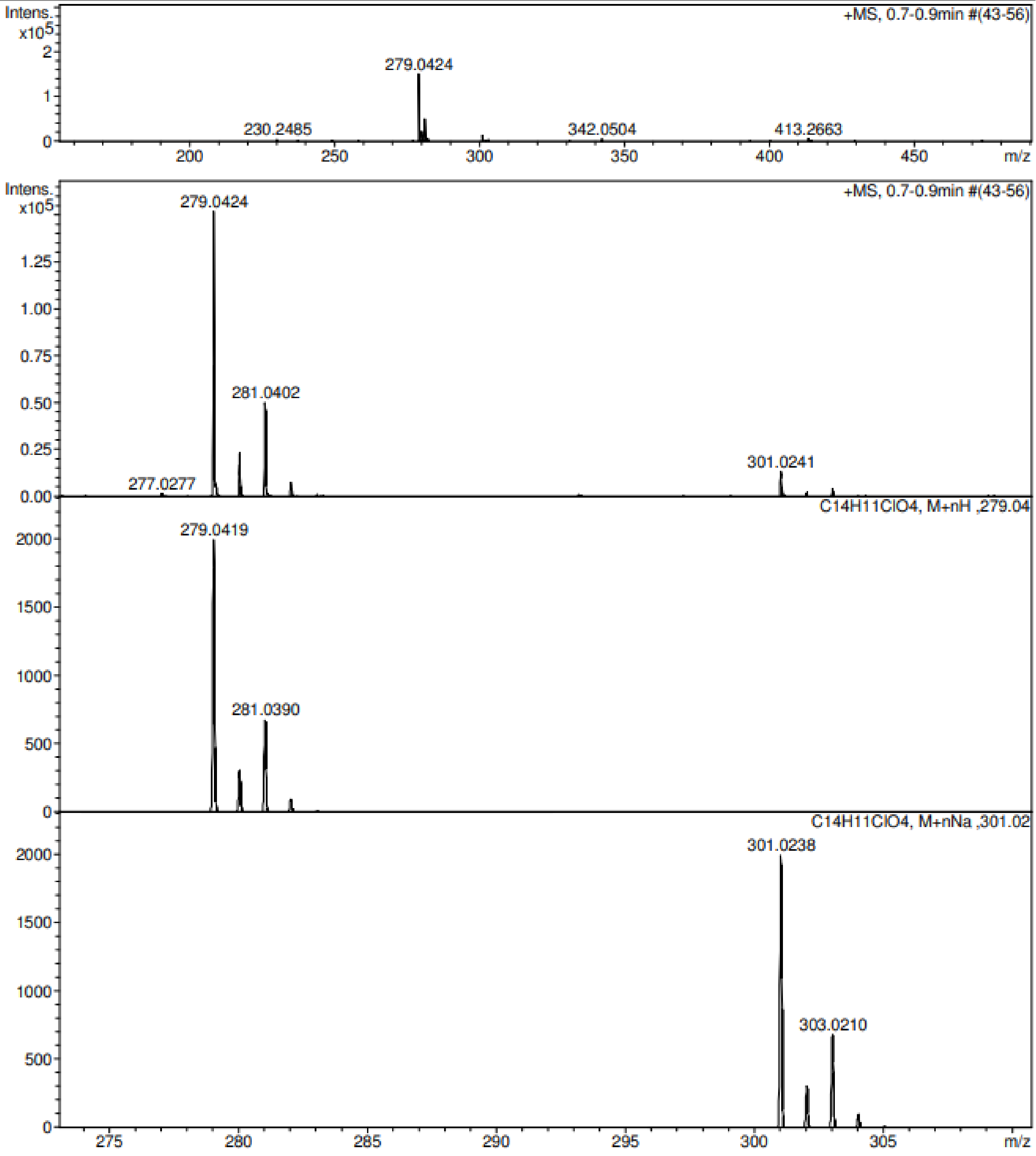

Bruker Compass DataAnalysis 4.0 printed: 15.02.2022 13:49:00 Page 1 of 1

HRMS for compound **19d**

Acquisition Parameter

Source Type	ESI	Ion Polarity	Positive	Set Nebulizer	0.4 Bar
Focus	Not active			Set Dry Heater	180 °C
Scan Begin	50 m/z	Set Capillary	4500 V	Set Dry Gas	4.0 l/min
Scan End	3000 m/z	Set End Plate Offset	-500 V	Set Divert Valve	Waste

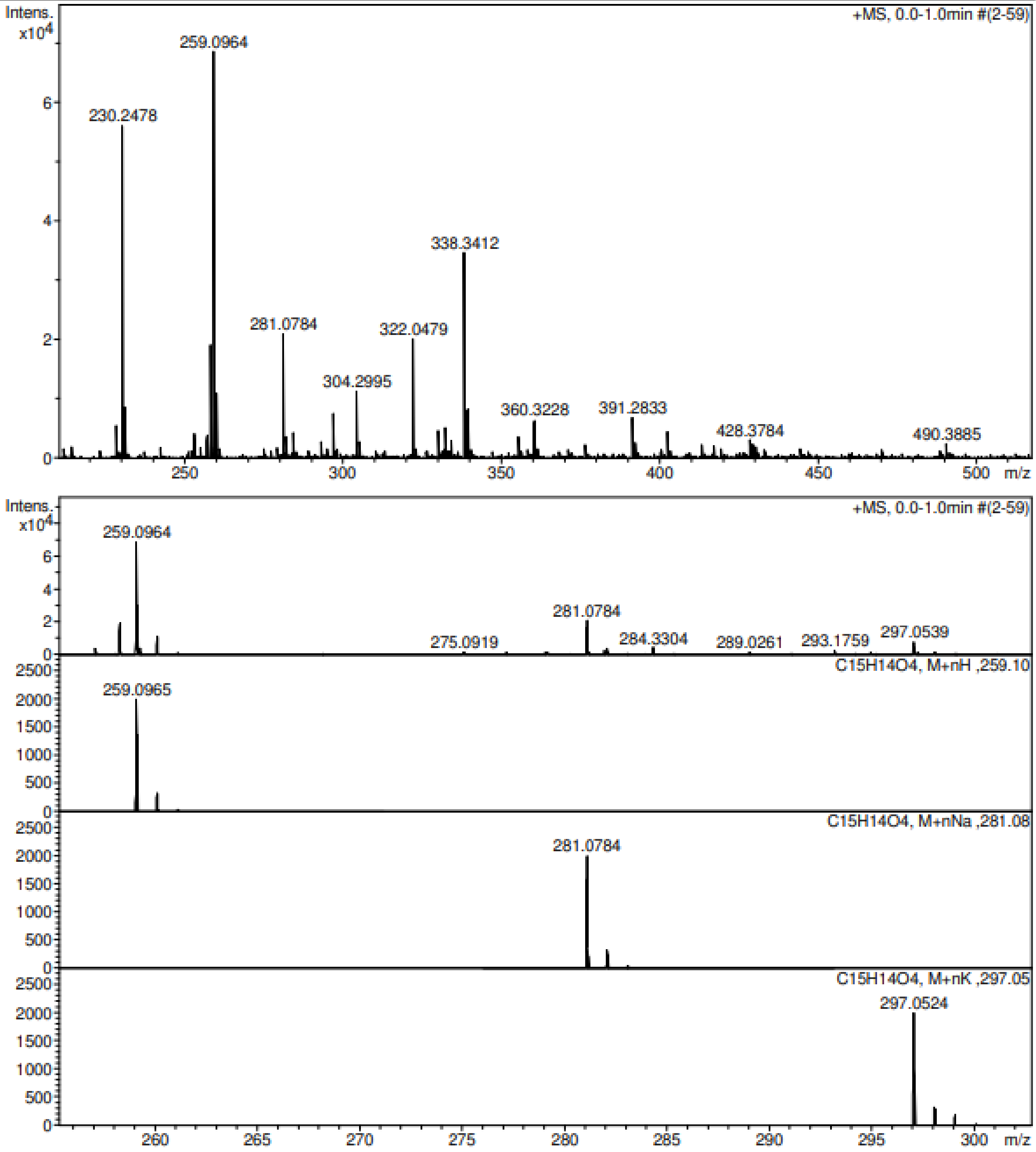

Bruker Compass DataAnalysis 4.0 printed: 11.02.2022 14:27:44 Page 1 of 1

HRMS for compound **19e**

Acquisition Parameter

Source Type	ESI	Ion Polarity	Positive	Set Nebulizer	0.4 Bar
Focus	Not active			Set Dry Heater	180 °C
Scan Begin	50 m/z	Set Capillary	4500 V	Set Dry Gas	4.0 l/min
Scan End	3000 m/z	Set End Plate Offset	-500 V	Set Divert Valve	Waste

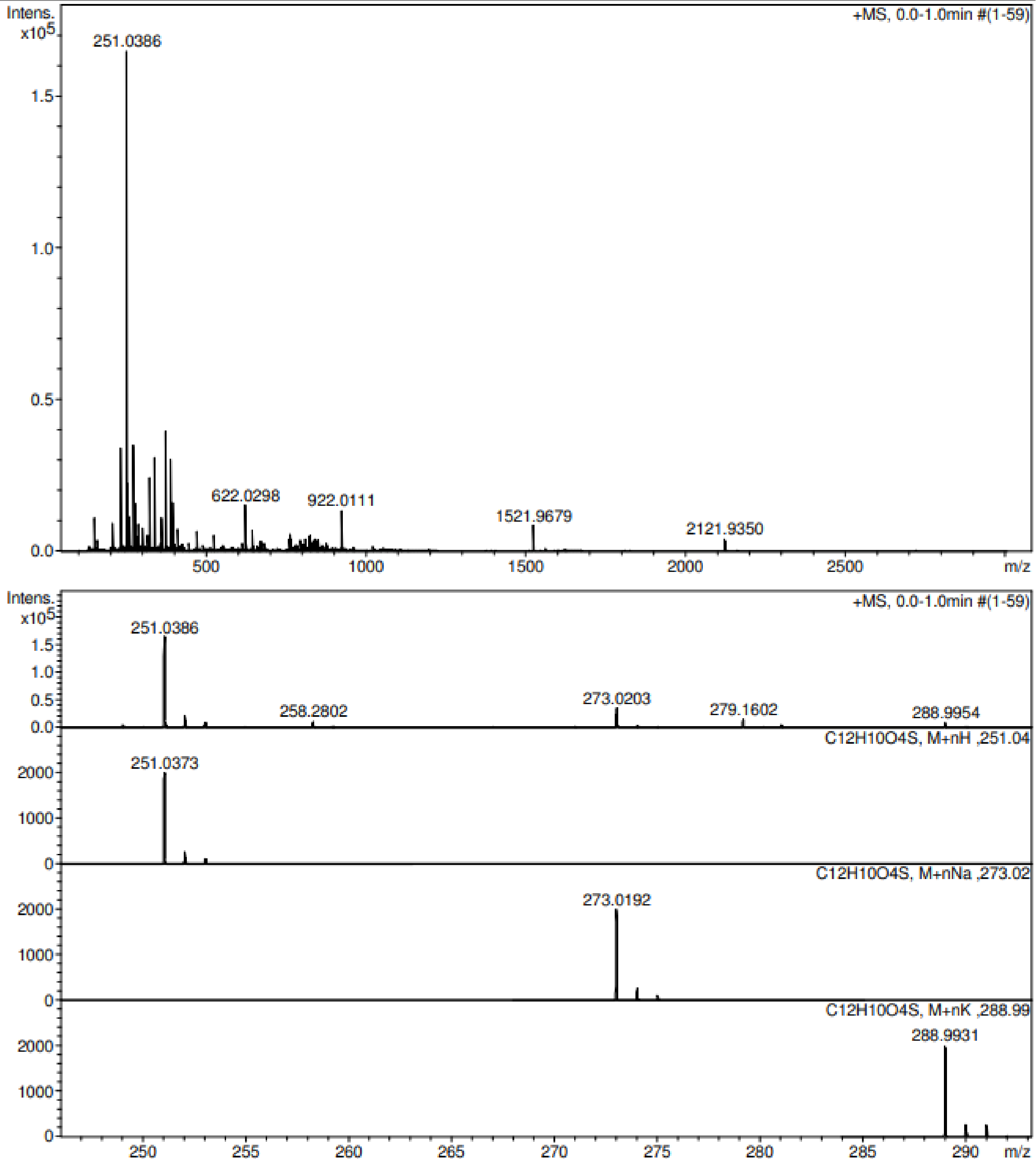

Bruker Compass DataAnalysis 4.0 printed: 04.02.2022 14:11:58 Page 1 of 1

HRMS for compound **20c**

Acquisition Parameter

Source Type	ESI	Ion Polarity	Positive	Set Nebulizer	0.4 Bar
Focus	Not active			Set Dry Heater	180 °C
Scan Begin	50 m/z	Set Capillary	4500 V	Set Dry Gas	4.0 l/min
Scan End	3000 m/z	Set End Plate Offset	-500 V	Set Divert Valve	Waste

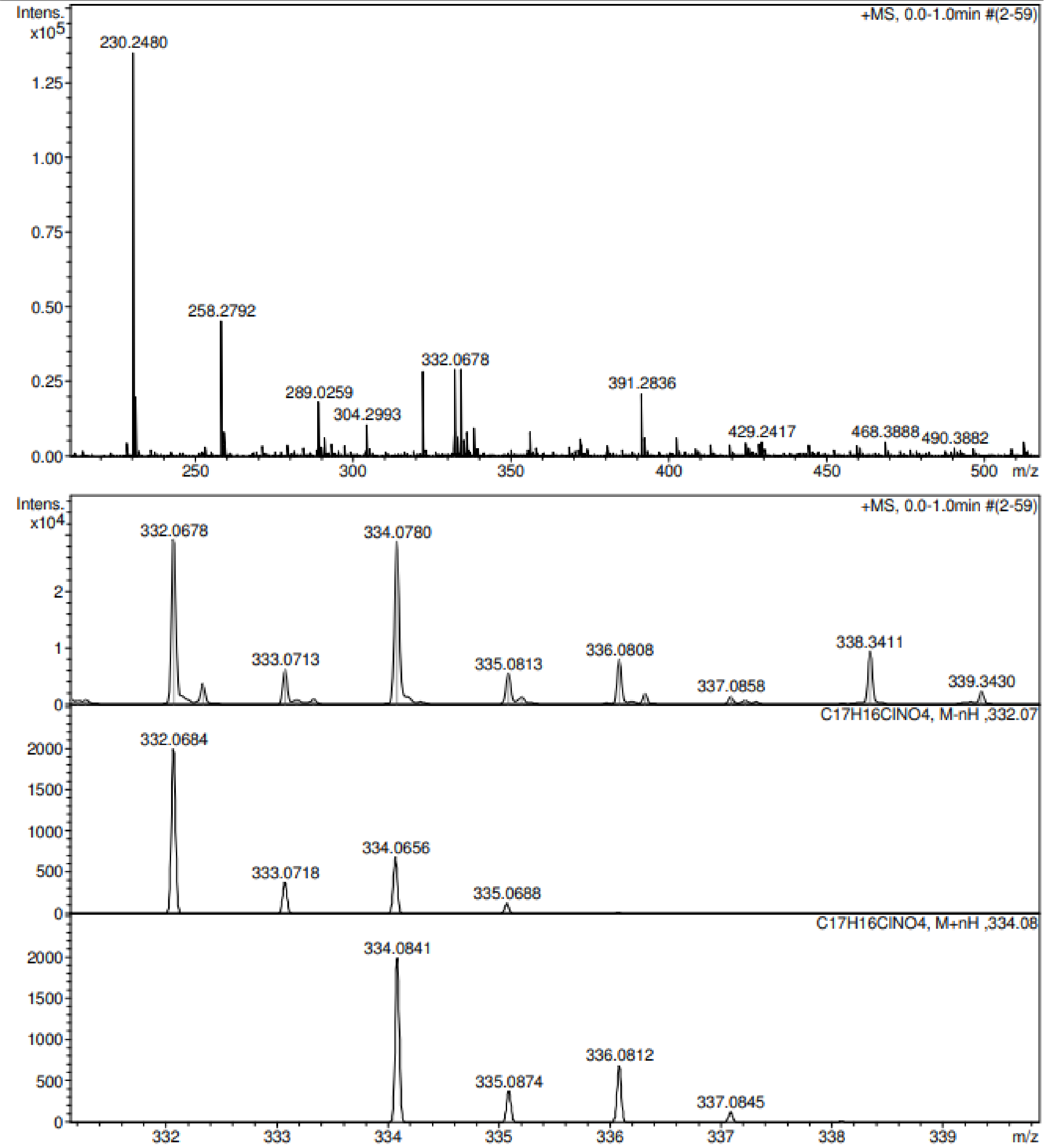

Bruker Compass DataAnalysis 4.0 printed: 11.02.2022 14:23:46 Page 1 of 1

HRMS for compound **20d**

Acquisition Parameter

Source Type	ESI	Ion Polarity	Positive	Set Nebulizer	0.4 Bar
Focus	Not active			Set Dry Heater	180 °C
Scan Begin	50 m/z	Set Capillary	4500 V	Set Dry Gas	4.0 l/min
Scan End	3000 m/z	Set End Plate Offset	-500 V	Set Divert Valve	Waste

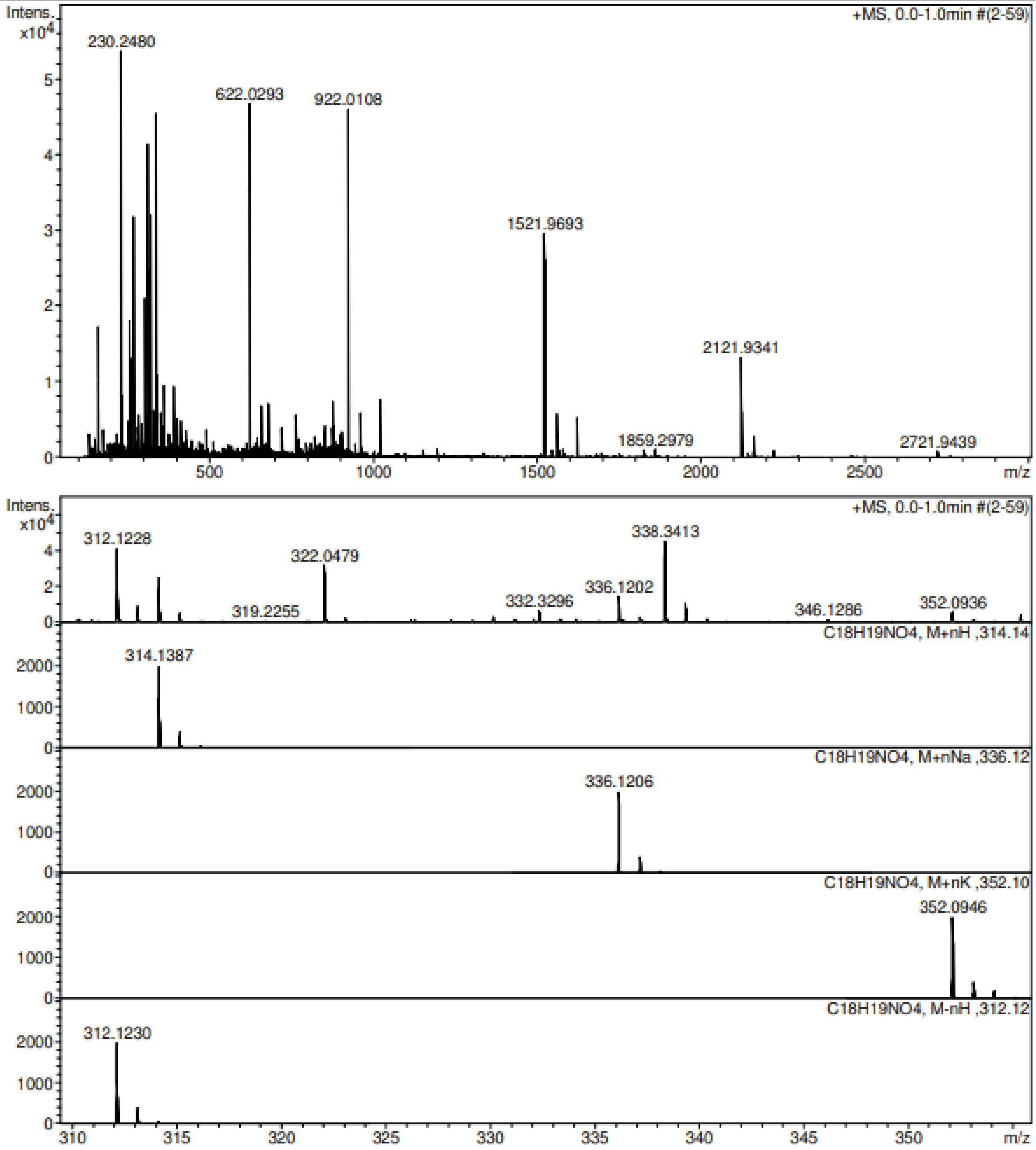

Bruker Compass DataAnalysis 4.0 printed: 11.02.2022 14:31:49 Page 1 of 1

HRMS for compound **20e**

Acquisition Parameter

Source Type	ESI	Ion Polarity	Positive	Set Nebulizer	0.4 Bar
Focus	Not active			Set Dry Heater	180 °C
Scan Begin	50 m/z	Set Capillary	4500 V	Set Dry Gas	4.0 l/min
Scan End	1600 m/z	Set End Plate Offset	-500 V	Set Divert Valve	Waste

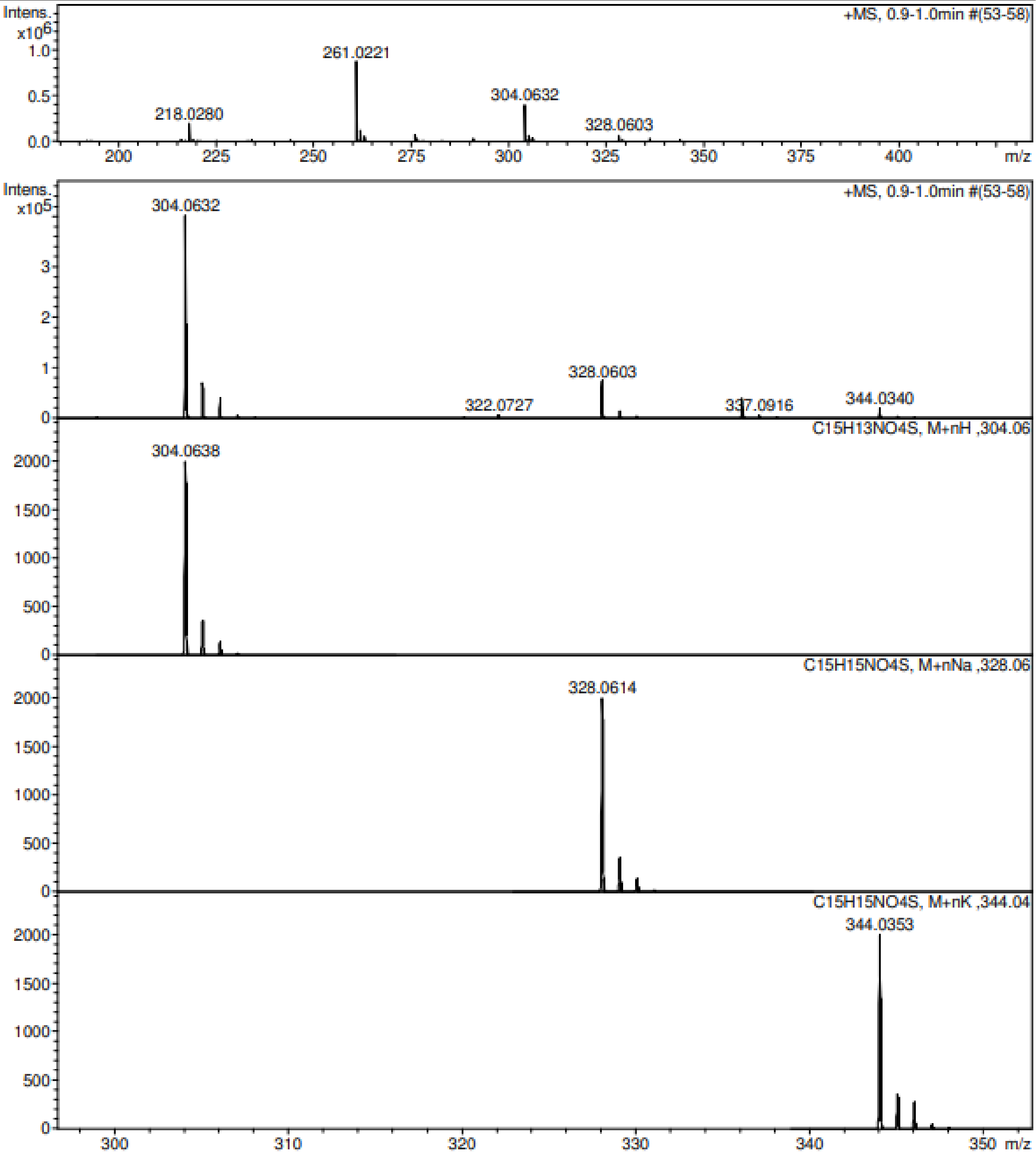

Bruker Compass DataAnalysis 4.0 printed: 15.02.2022 13:58:24 Page 1 of 1

HRMS for compound **12b**

Acquisition Parameter

Source Type	ESI	Ion Polarity	Positive	Set Nebulizer	0.4 Bar
Focus	Not active			Set Dry Heater	180 °C
Scan Begin	50 m/z	Set Capillary	4500 V	Set Dry Gas	4.0 l/min
Scan End	1600 m/z	Set End Plate Offset	-500 V	Set Divert Valve	Waste

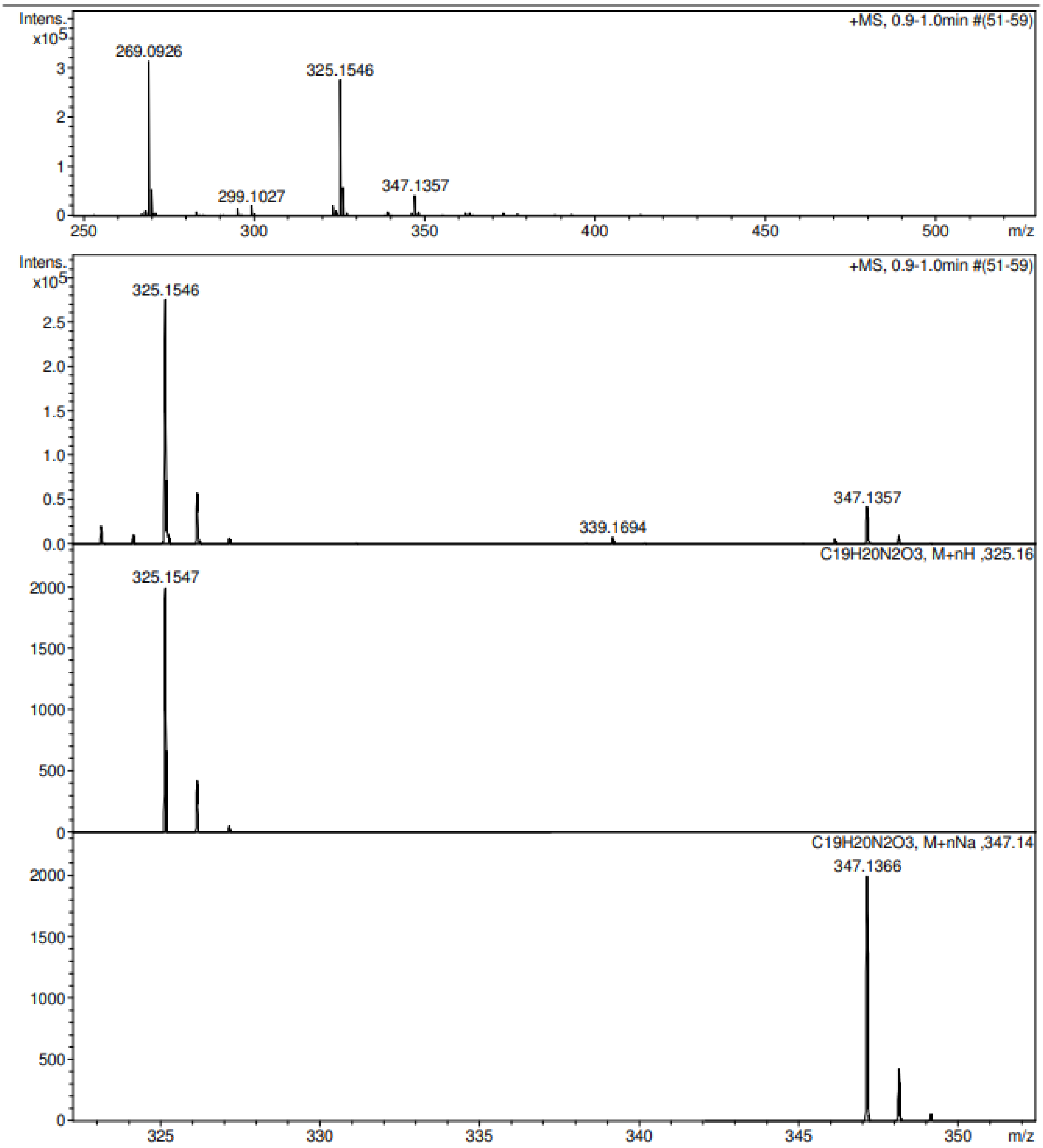

Bruker Compass DataAnalysis 4.0 printed: 08.02.2022 17:44:30 Page 1 of 1

HRMS for compound **12c**

Acquisition Parameter

Source Type	ESI	Ion Polarity	Positive	Set Nebulizer	0.4 Bar
Focus	Not active			Set Dry Heater	180 °C
Scan Begin	50 m/z	Set Capillary	4500 V	Set Dry Gas	4.0 l/min
Scan End	1600 m/z	Set End Plate Offset	-500 V	Set Divert Valve	Waste

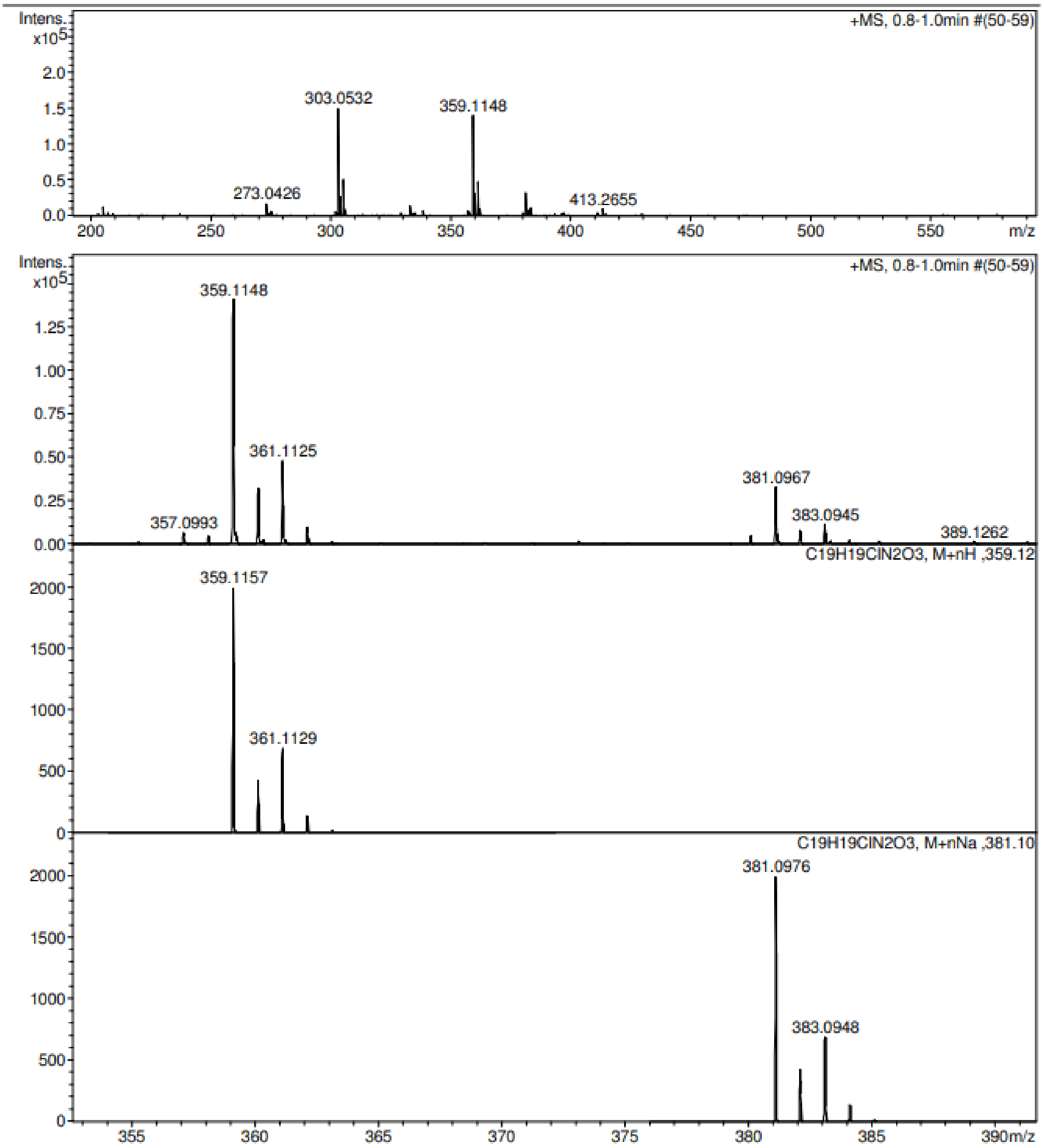

HRMS for compound **12d**

Acquisition Parameter

Source Type	ESI	Ion Polarity	Positive	Set Nebulizer	0.4 Bar
Focus	Not active			Set Dry Heater	180 °C
Scan Begin	50 m/z	Set Capillary	4500 V	Set Dry Gas	4.0 l/min
Scan End	1600 m/z	Set End Plate Offset	-500 V	Set Divert Valve	Waste

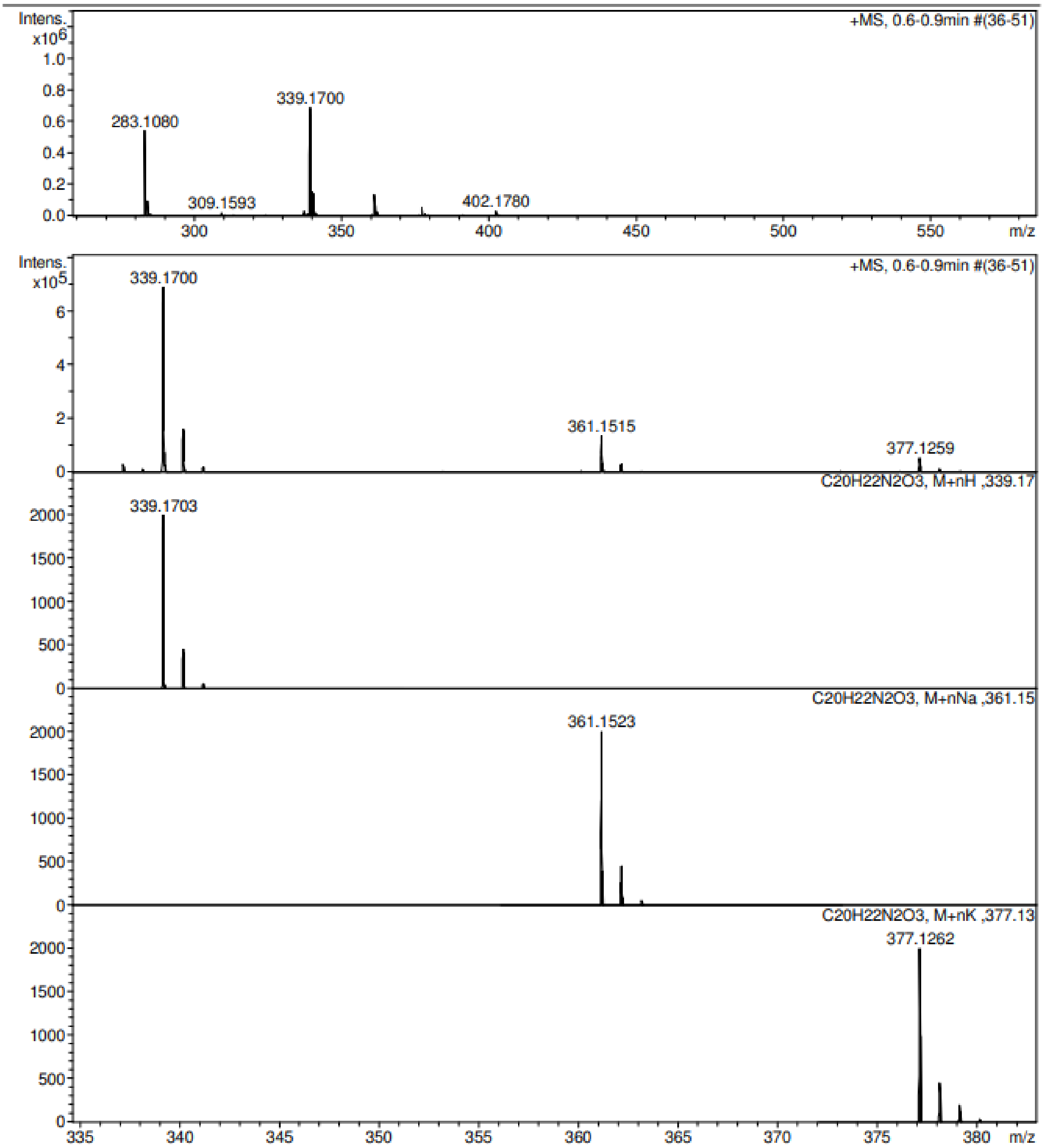

Bruker Compass DataAnalysis 4.0 printed: 08.02.2022 17:35:22 Page 1 of 1

HRMS for compound **12e**

Acquisition Parameter

Source Type	ESI	Ion Polarity	Positive	Set Nebulizer	0.4 Bar
Focus	Not active			Set Dry Heater	180 °C
Scan Begin	50 m/z	Set Capillary	4500 V	Set Dry Gas	4.0 l/min
Scan End	3000 m/z	Set End Plate Offset	-500 V	Set Divert Valve	Waste

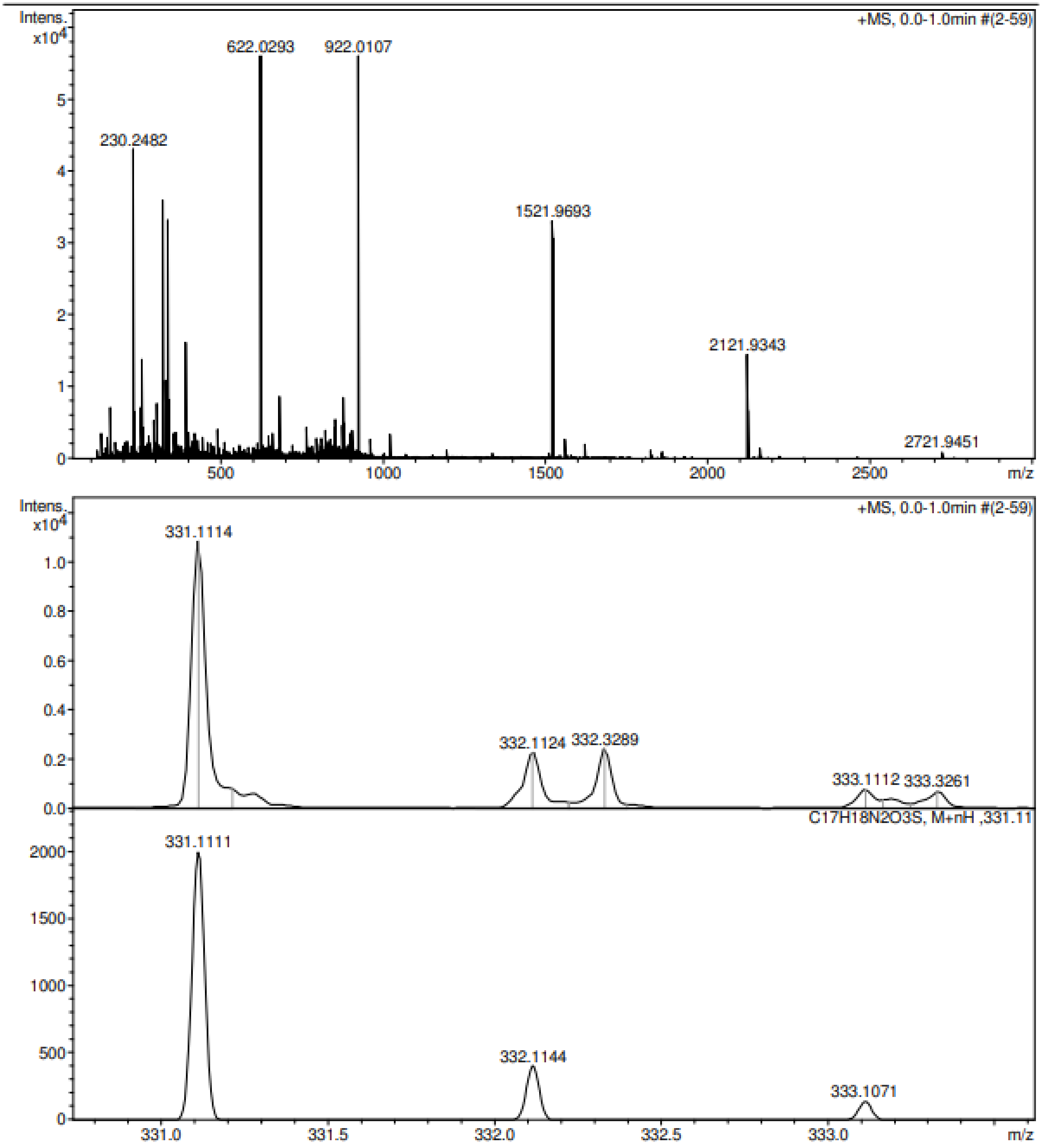

Bruker Compass DataAnalysis 4.0 printed: 11.02.2022 14:33:43 Page 1 of 1

HRMS for compound **12g**

Acquisition Parameter

Source Type	ESI	Ion Polarity	Positive	Set Nebulizer	0.4 Bar
Focus	Not active			Set Dry Heater	180 °C
Scan Begin	50 m/z	Set Capillary	4500 V	Set Dry Gas	4.0 l/min
Scan End	3000 m/z	Set End Plate Offset	-500 V	Set Divert Valve	Waste

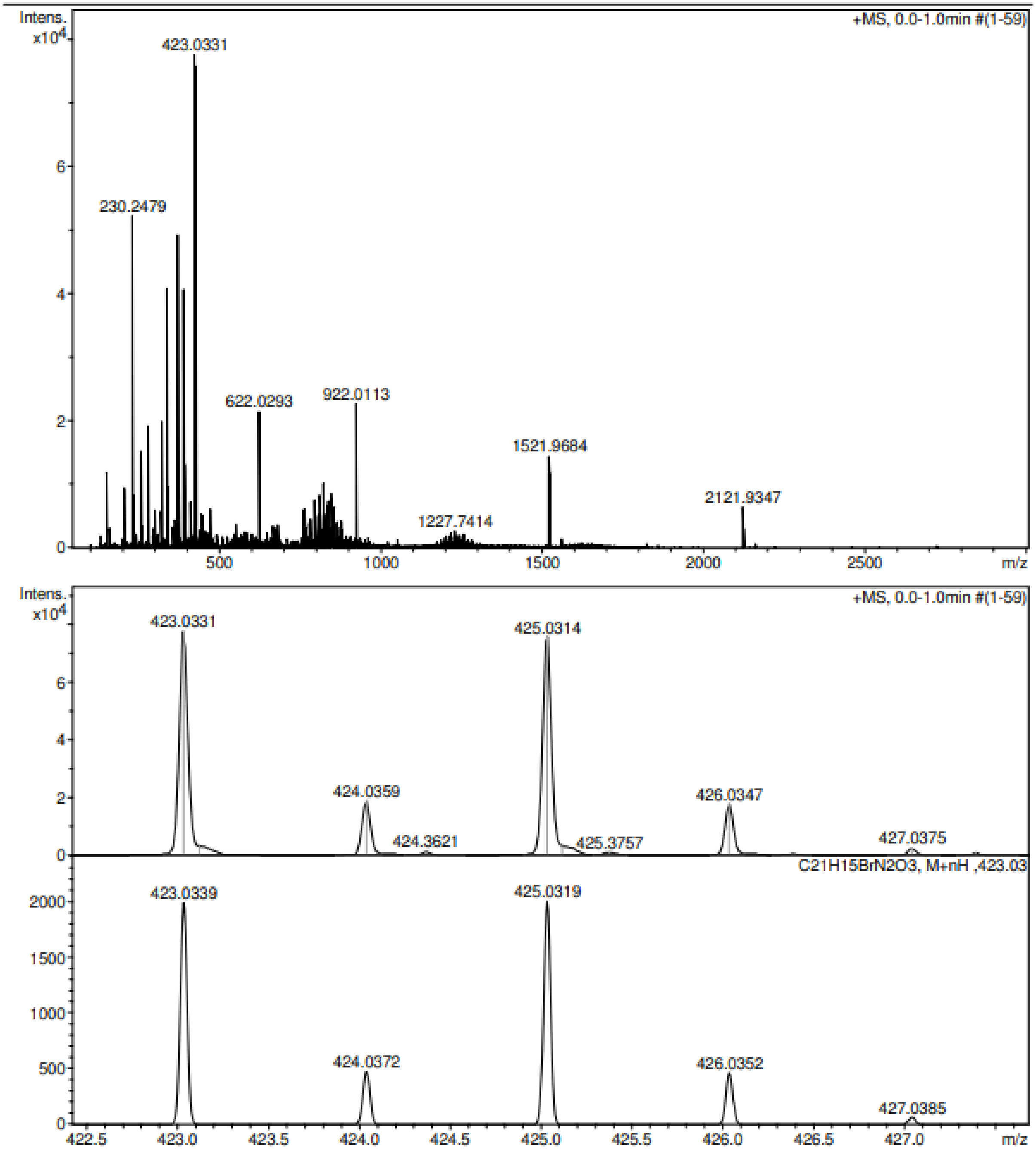

Bruker Compass DataAnalysis 4.0 printed: 04.02.2022 13:59:43 Page 1 of 1

HRMS for compound **12j**

Acquisition Parameter

Source Type	ESI	Ion Polarity	Positive	Set Nebulizer	0.4 Bar
Focus	Not active			Set Dry Heater	180 °C
Scan Begin	50 m/z	Set Capillary	4500 V	Set Dry Gas	4.0 l/min
Scan End	1600 m/z	Set End Plate Offset	-500 V	Set Divert Valve	Waste

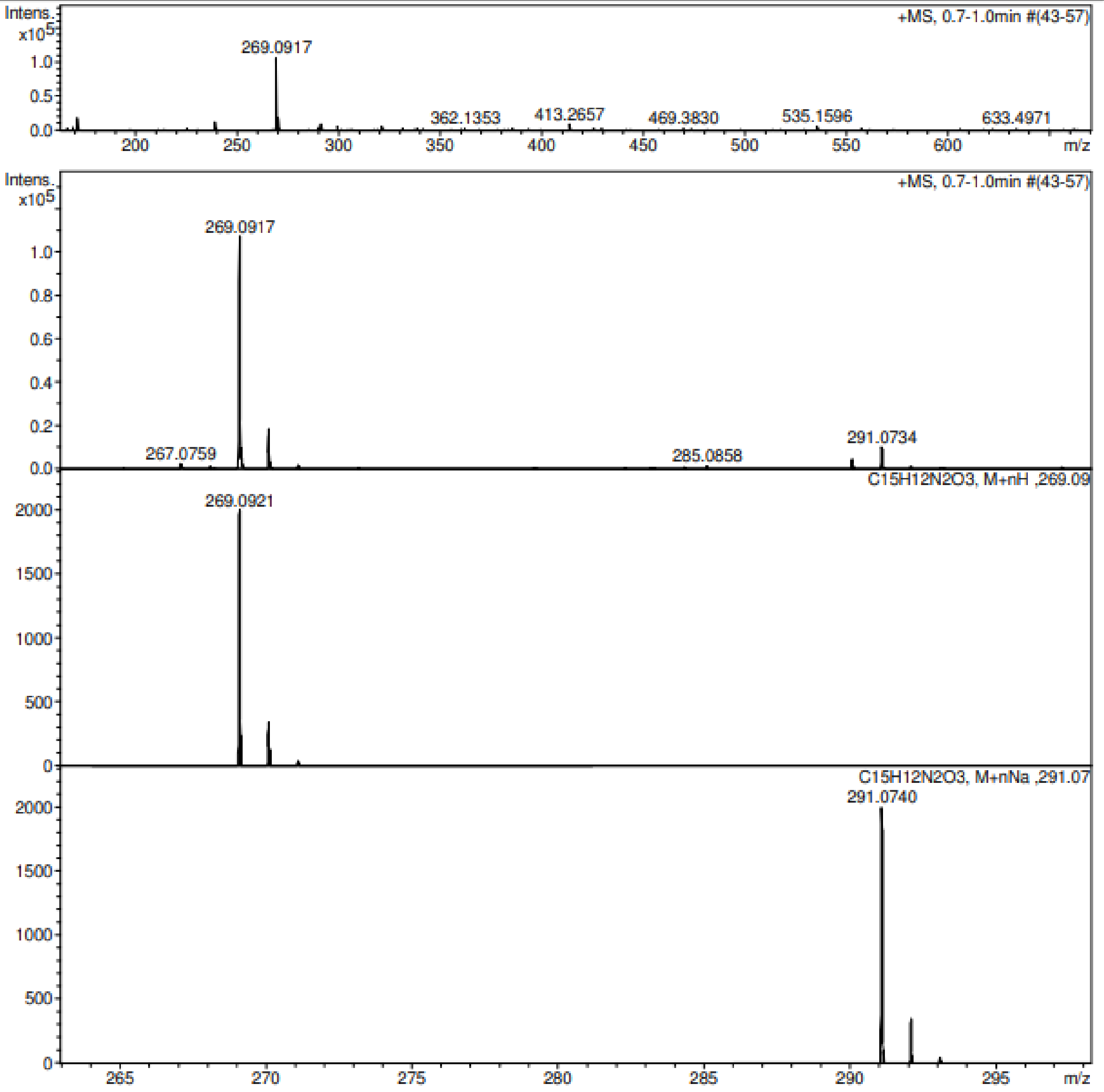

Bruker Compass DataAnalysis 4.0 printed: 17.03.2022 16:53:19 Page 1 of 1

HRMS for compound **12k**

Acquisition Parameter

Source Type	ESI	Ion Polarity	Positive	Set Nebulizer	0.4 Bar
Focus	Not active			Set Dry Heater	180 °C
Scan Begin	50 m/z	Set Capillary	4500 V	Set Dry Gas	4.0 l/min
Scan End	1600 m/z	Set End Plate Offset	-500 V	Set Divert Valve	Waste

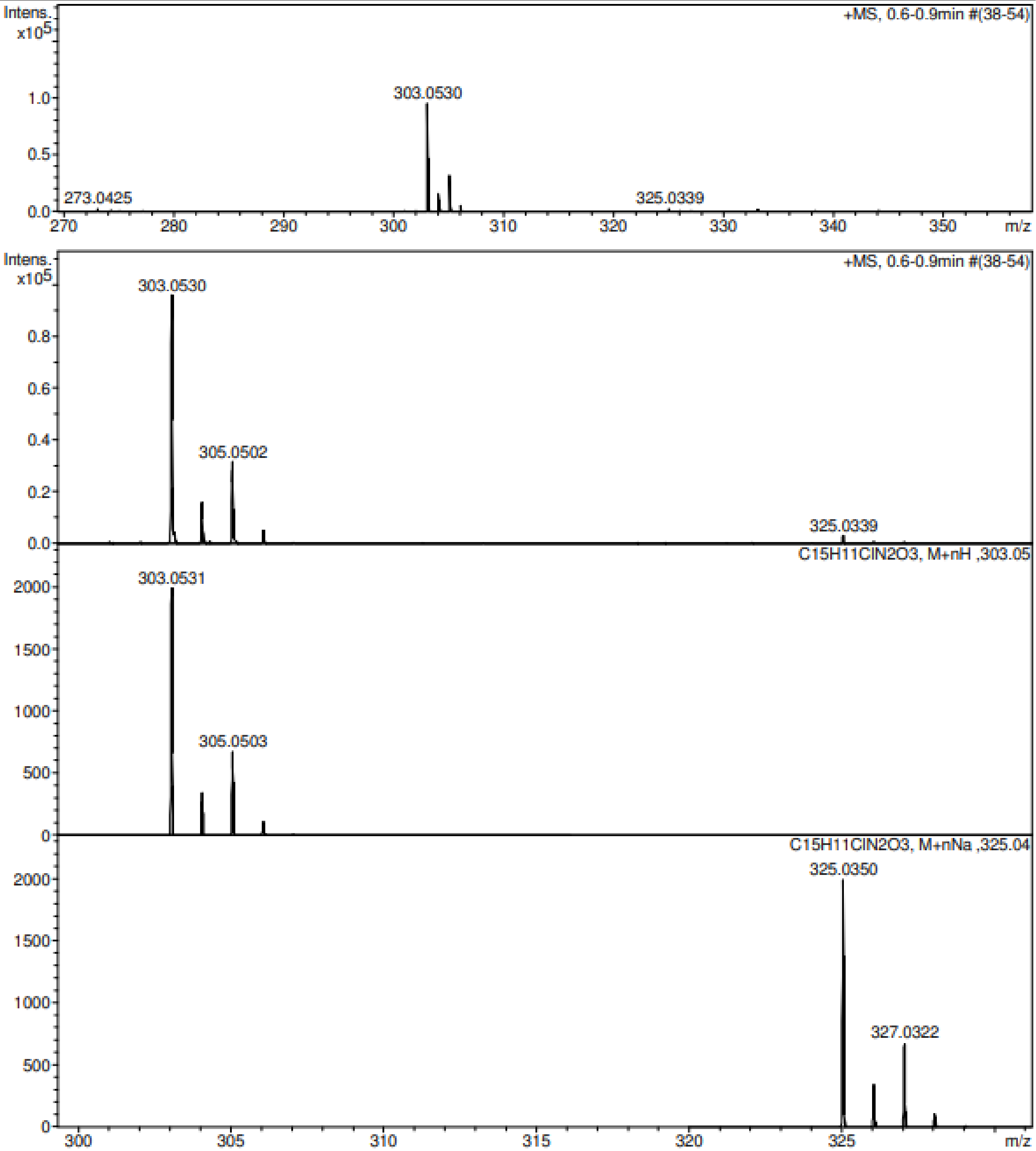

Bruker Compass DataAnalysis 4.0 printed: 08.02.2022 17:26:21 Page 1 of 1

HRMS for compound **12l**

Acquisition Parameter

Source Type	ESI	Ion Polarity	Positive	Set Nebulizer	0.4 Bar
Focus	Not active			Set Dry Heater	180 °C
Scan Begin	50 m/z	Set Capillary	4500 V	Set Dry Gas	4.0 l/min
Scan End	1600 m/z	Set End Plate Offset	-500 V	Set Divert Valve	Waste

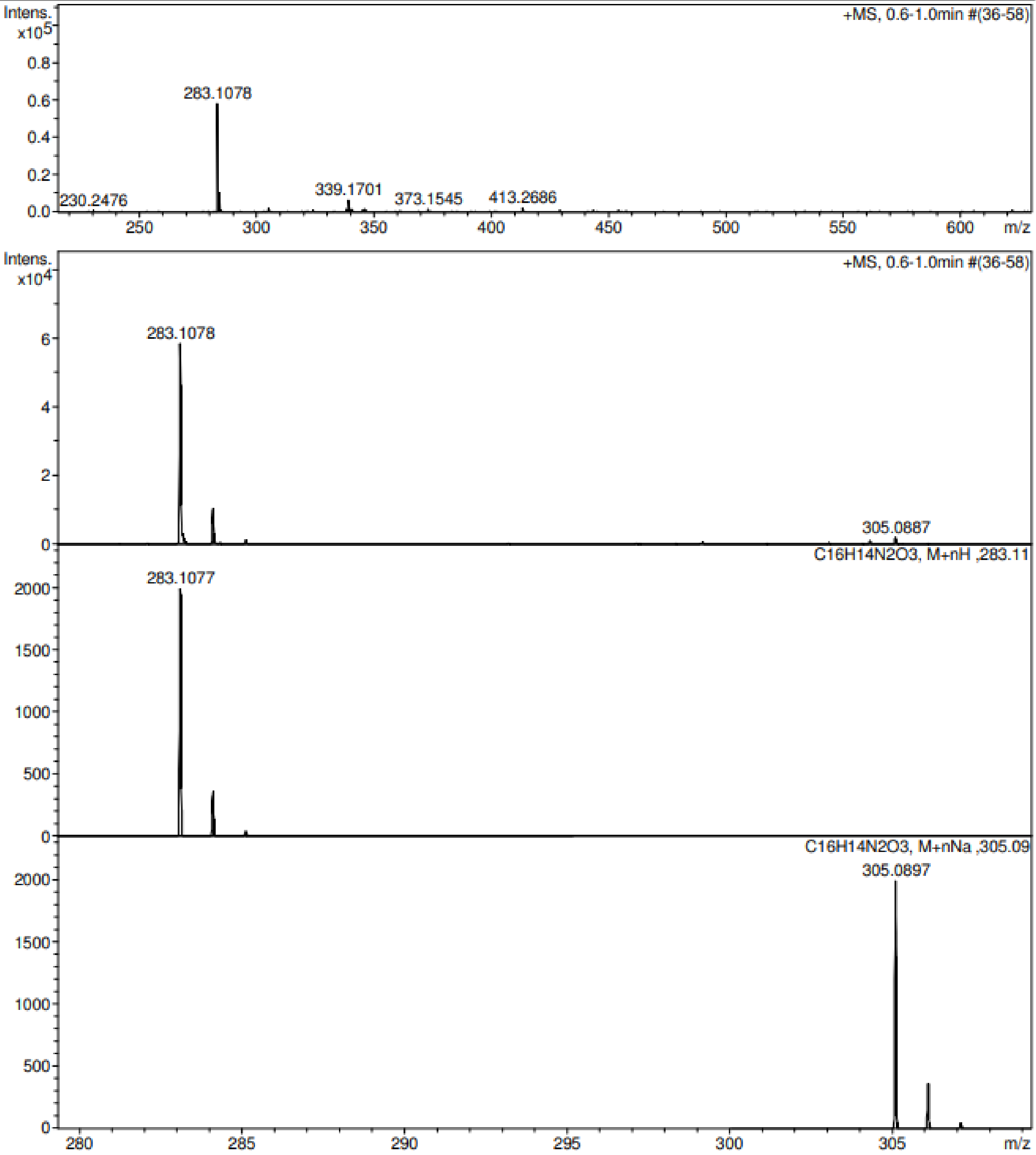

HRMS for compound **12m**

Acquisition Parameter

Source Type	ESI	Ion Polarity	Positive	Set Nebulizer	0.4 Bar
Focus	Not active			Set Dry Heater	180 °C
Scan Begin	50 m/z	Set Capillary	4500 V	Set Dry Gas	4.0 l/min
Scan End	3000 m/z	Set End Plate Offset	-500 V	Set Divert Valve	Waste

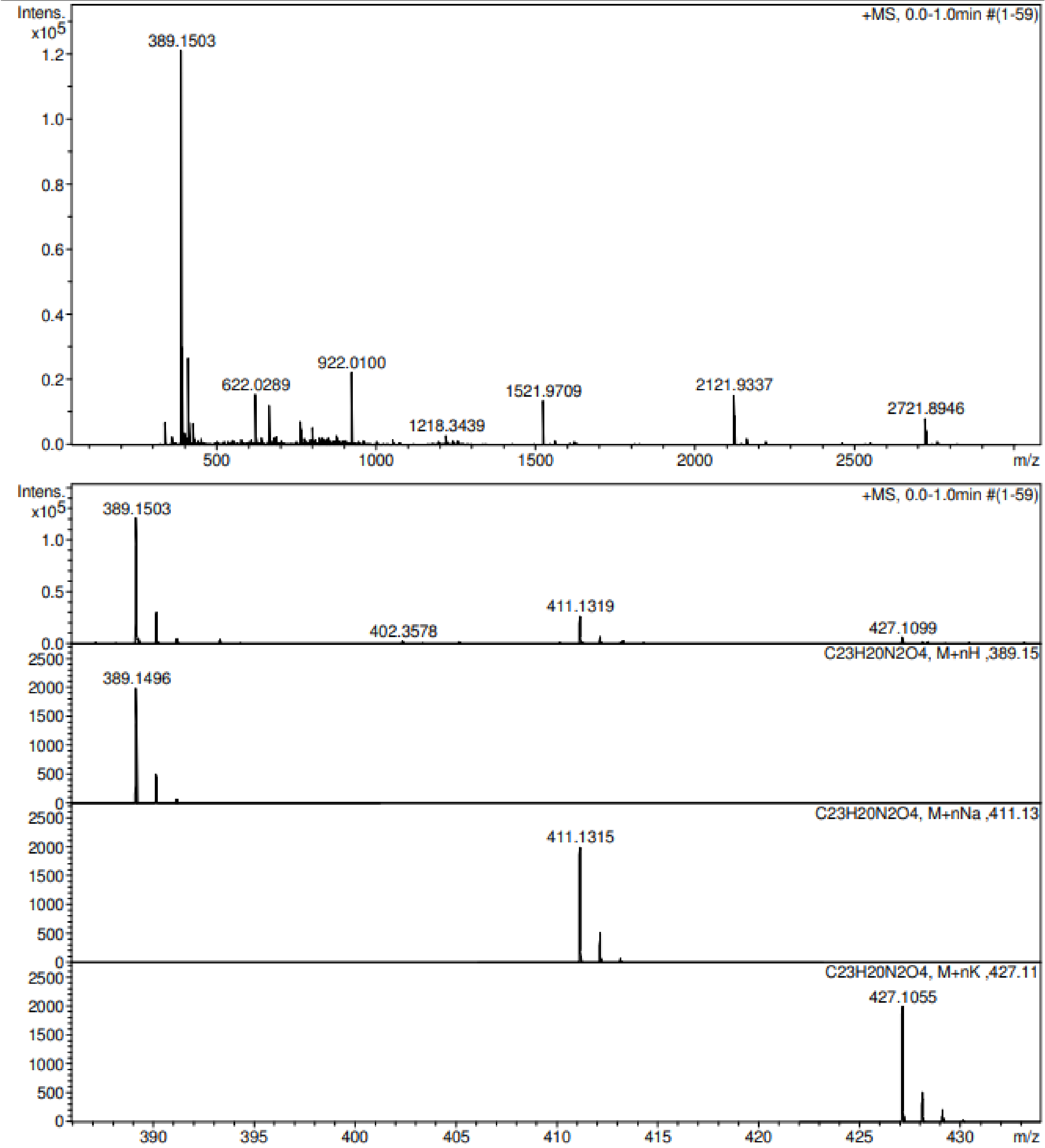

Bruker Compass DataAnalysis 4.0 printed: 27.01.2022 14:44:56 Page 1 of 1

HRMS for compound **12n**

Acquisition Parameter

Source Type	ESI	Ion Polarity	Positive	Set Nebulizer	0.4 Bar
Focus	Not active			Set Dry Heater	180 °C
Scan Begin	50 m/z	Set Capillary	4500 V	Set Dry Gas	4.0 l/min
Scan End	3000 m/z	Set End Plate Offset	-500 V	Set Divert Valve	Waste

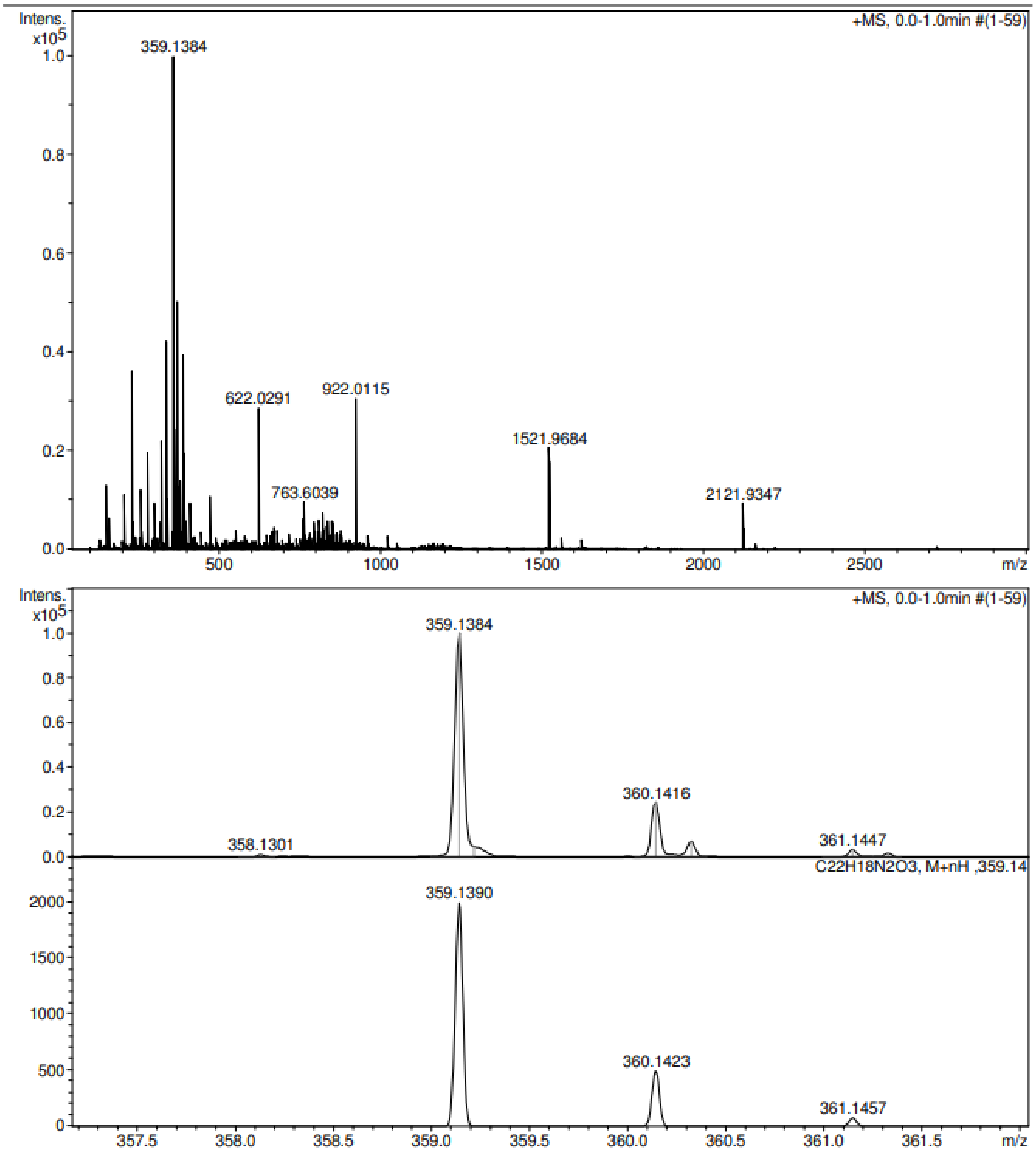

Bruker Compass DataAnalysis 4.0 printed: 04.02.2022 14:03:17 Page 1 of 1

HRMS for compound **12o**

Acquisition Parameter

Source Type	ESI	Ion Polarity	Positive	Set Nebulizer	0.4 Bar
Focus	Not active			Set Dry Heater	180 °C
Scan Begin	50 m/z	Set Capillary	4500 V	Set Dry Gas	4.0 l/min
Scan End	1600 m/z	Set End Plate Offset	-500 V	Set Divert Valve	Waste

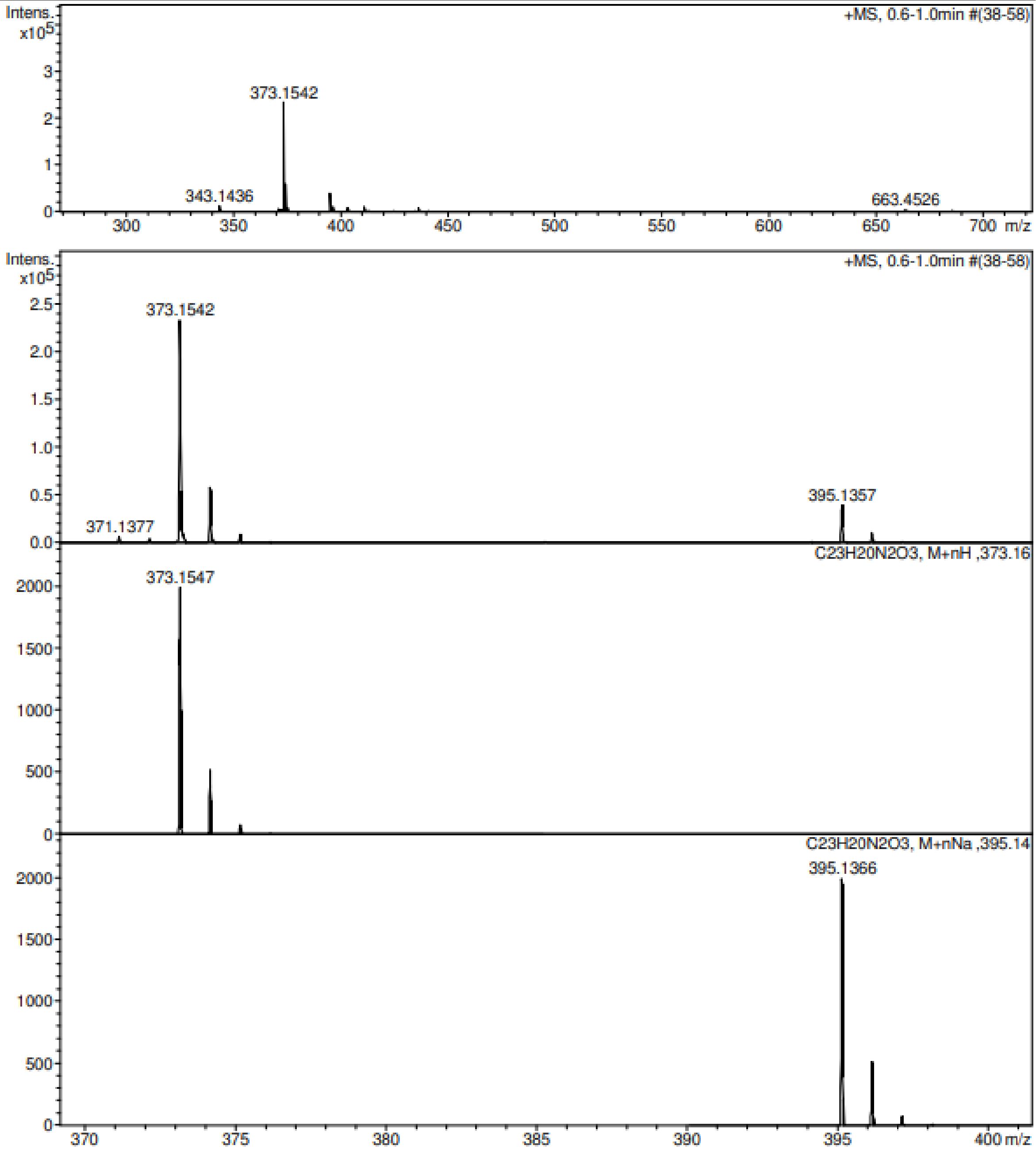

Bruker Compass DataAnalysis 4.0 printed: 08.02.2022 17:31:11 Page 1 of 1

HRMS for compound **16**

Acquisition Parameter

Source Type	ESI	Ion Polarity	Positive	Set Nebulizer	0.4 Bar
Focus	Not active			Set Dry Heater	180 °C
Scan Begin	50 m/z	Set Capillary	4500 V	Set Dry Gas	4.0 l/min
Scan End	3000 m/z	Set End Plate Offset	-500 V	Set Divert Valve	Waste

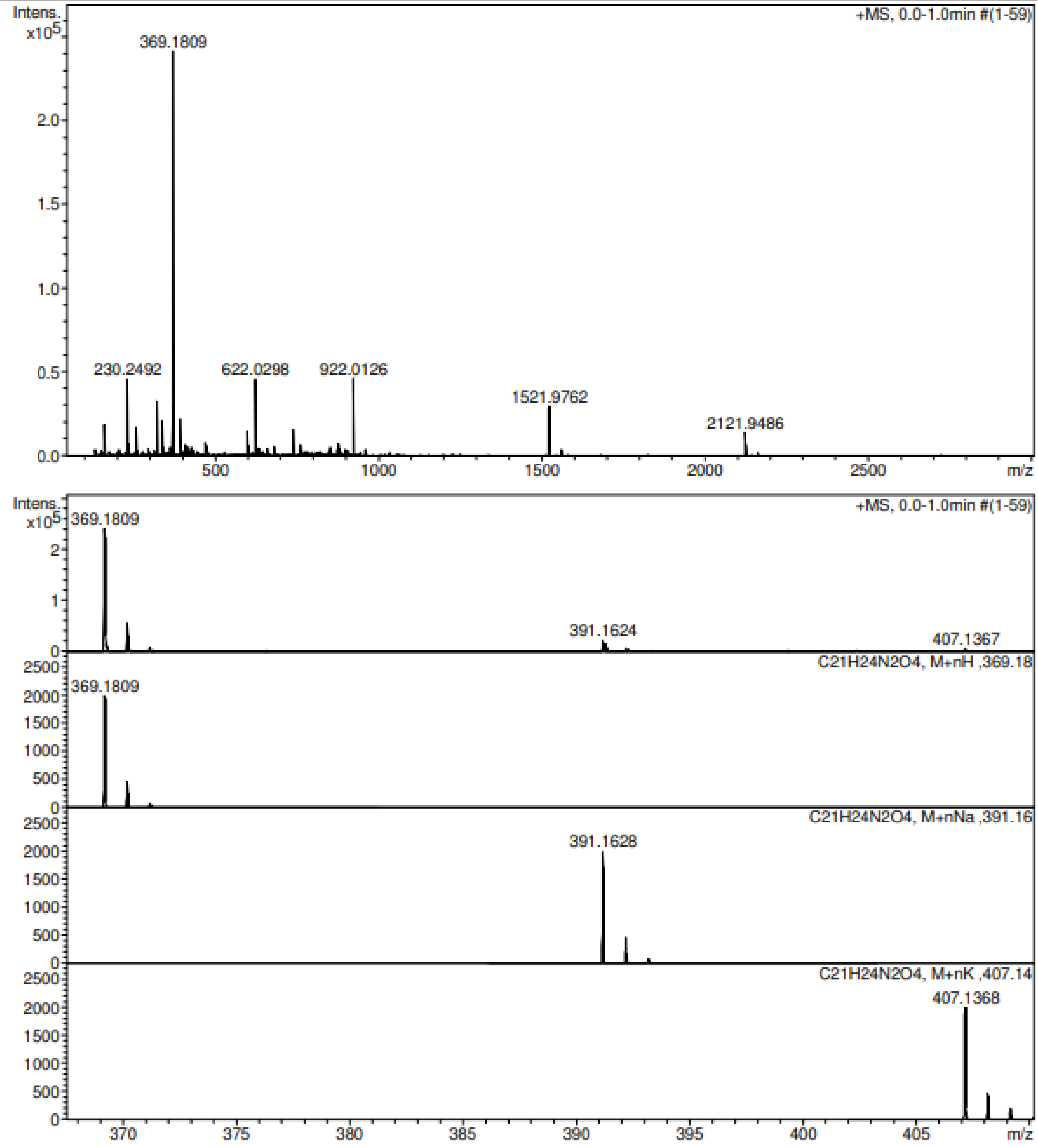

Bruker Compass DataAnalysis 4.0 printed: 11.02.2022 14:09:12 Page 1 of 1

HRMS for compound **14a**

Acquisition Parameter

Source Type	ESI	Ion Polarity	Positive	Set Nebulizer	0.4 Bar
Focus	Not active			Set Dry Heater	180 °C
Scan Begin	50 m/z	Set Capillary	4500 V	Set Dry Gas	4.0 l/min
Scan End	3000 m/z	Set End Plate Offset	-500 V	Set Divert Valve	Waste

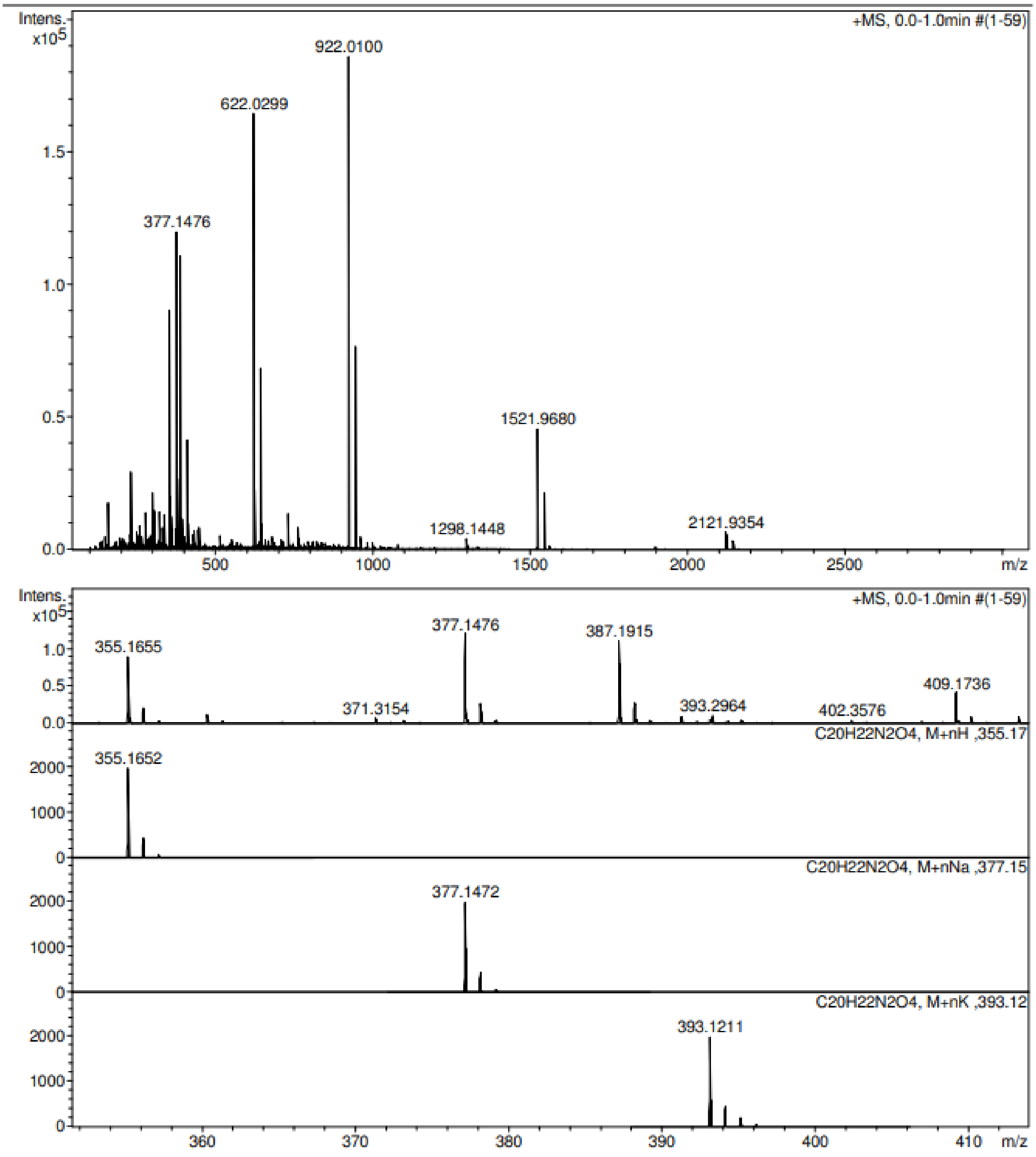

Bruker Compass DataAnalysis 4.0 printed: 04.06.2021 17:01:16 Page 1 of 1

HRMS for compound **145a**

Acquisition Parameter

Source Type	ESI	Ion Polarity	Positive	Set Nebulizer	0.4 Bar
Focus	Not active			Set Dry Heater	180 °C
Scan Begin	50 m/z	Set Capillary	4500 V	Set Dry Gas	4.0 l/min
Scan End	3000 m/z	Set End Plate Offset	-500 V	Set Divert Valve	Waste

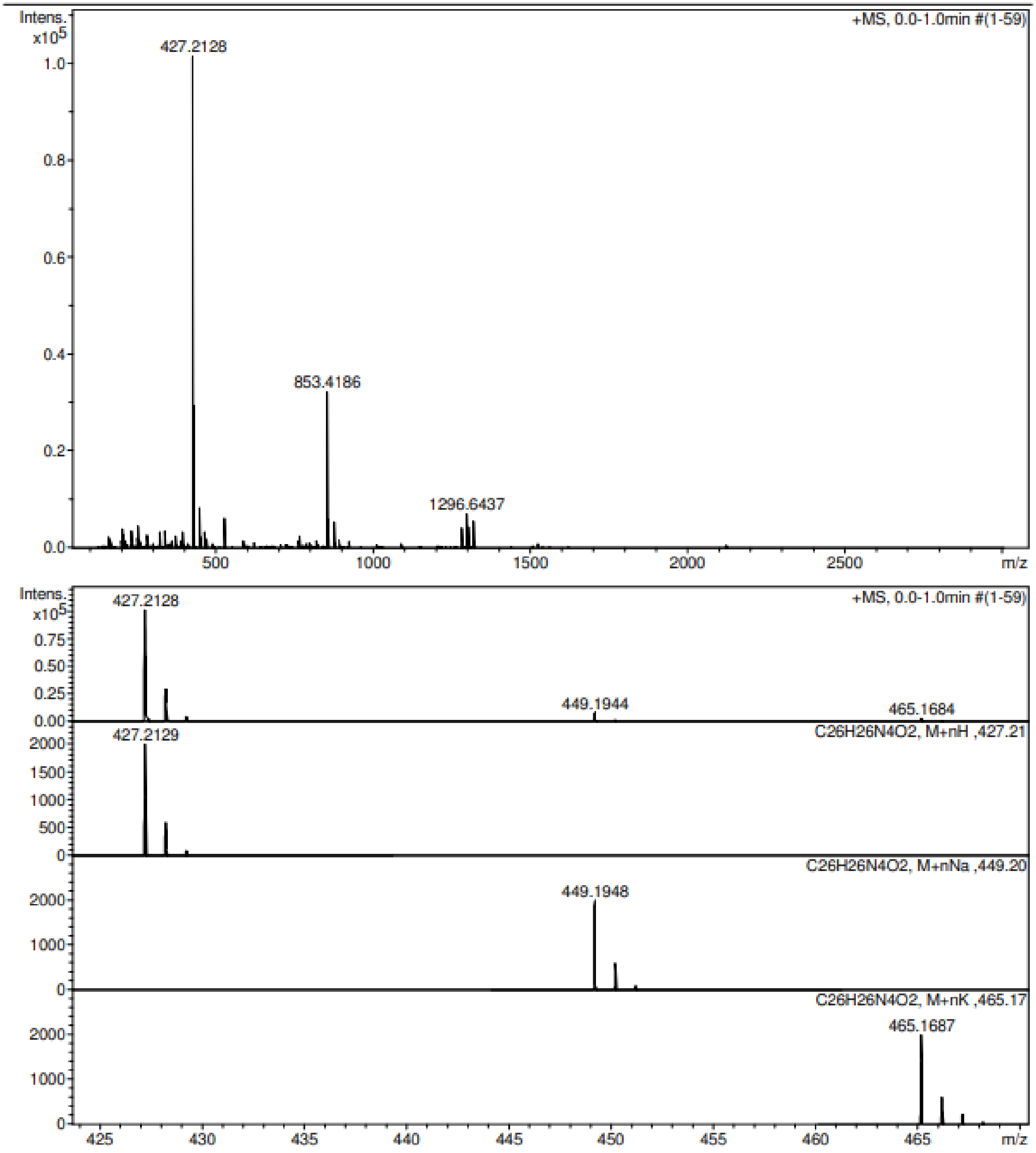

Bruker Compass DataAnalysis 4.0 printed: 01.02.2022 17:03:52 Page 1 of 1

HRMS for compound **15b**

Acquisition Parameter

Source Type	ESI	Ion Polarity	Positive	Set Nebulizer	0.4 Bar
Focus	Not active			Set Dry Heater	180 °C
Scan Begin	50 m/z	Set Capillary	4500 V	Set Dry Gas	4.0 l/min
Scan End	3000 m/z	Set End Plate Offset	-500 V	Set Divert Valve	Waste

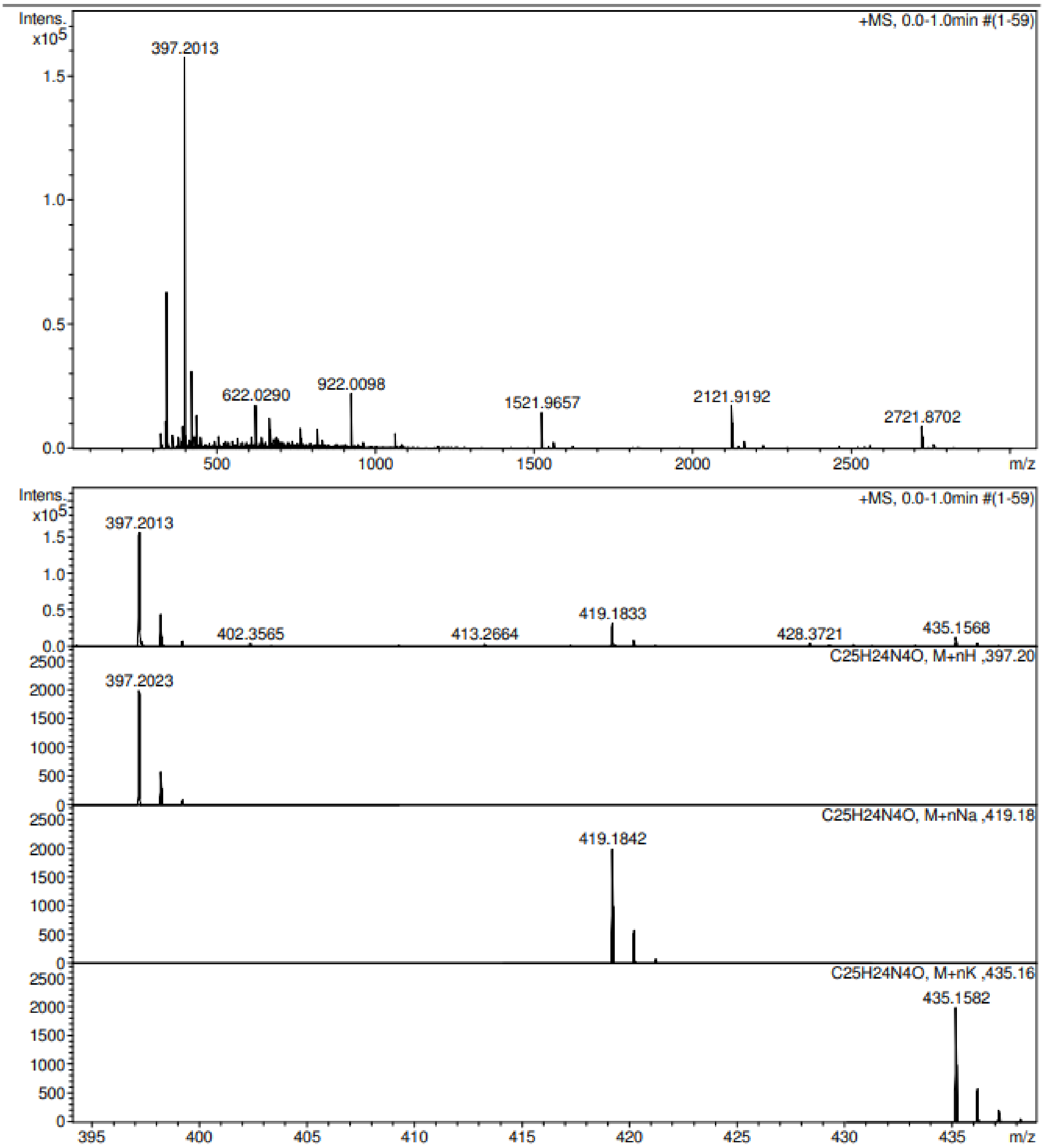

Bruker Compass DataAnalysis 4.0 printed: 27.01.2022 14:36:41 Page 1 of 1

HRMS for compound **15c**

Acquisition Parameter

Source Type	ESI	Ion Polarity	Positive	Set Nebulizer	0.4 Bar
Focus	Not active			Set Dry Heater	180 °C
Scan Begin	50 m/z	Set Capillary	4500 V	Set Dry Gas	4.0 l/min
Scan End	3000 m/z	Set End Plate Offset	-500 V	Set Divert Valve	Waste

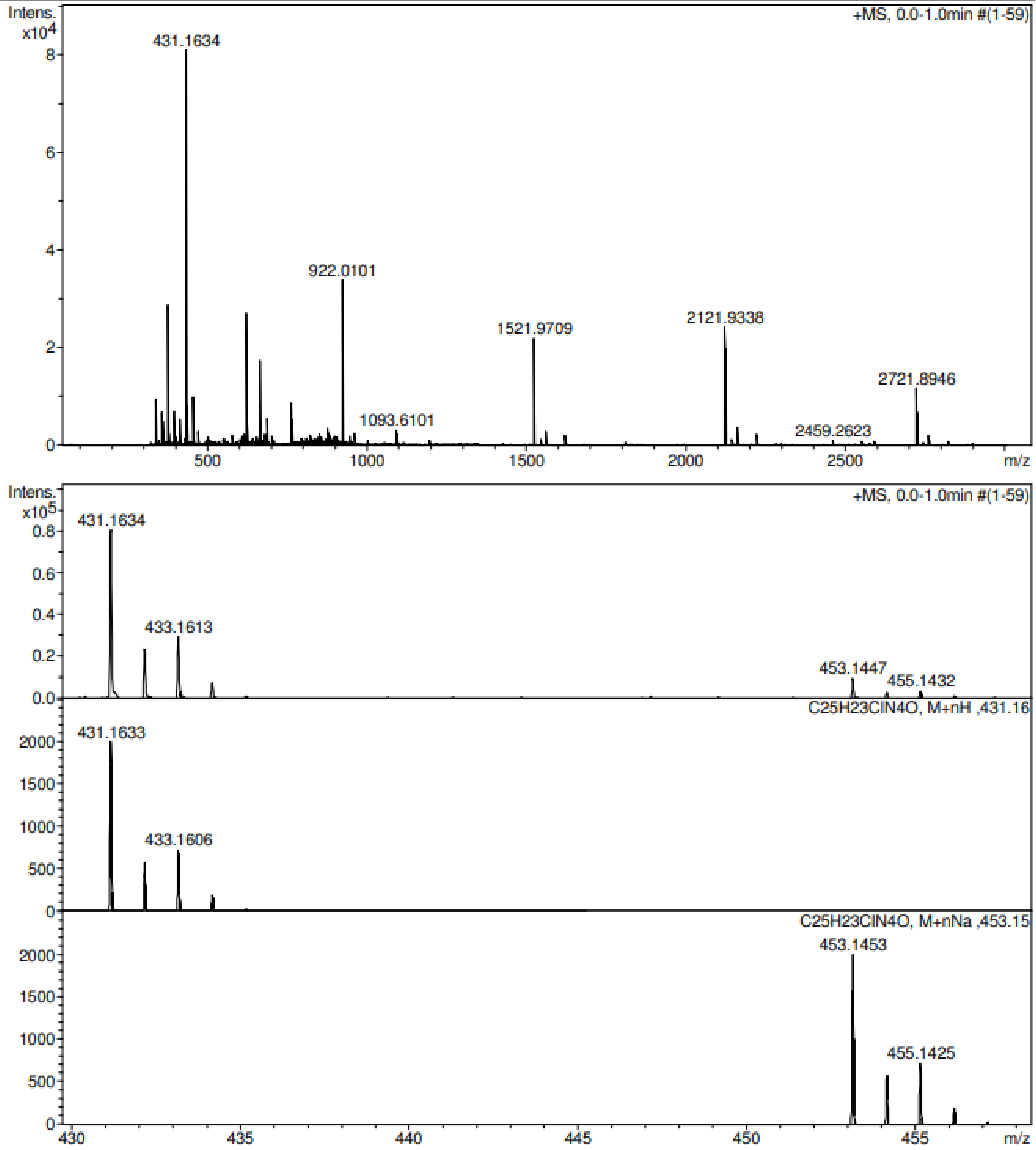

Bruker Compass DataAnalysis 4.0 printed: 27.01.2022 14:42:18 Page 1 of 1

HRMS for compound **15d**

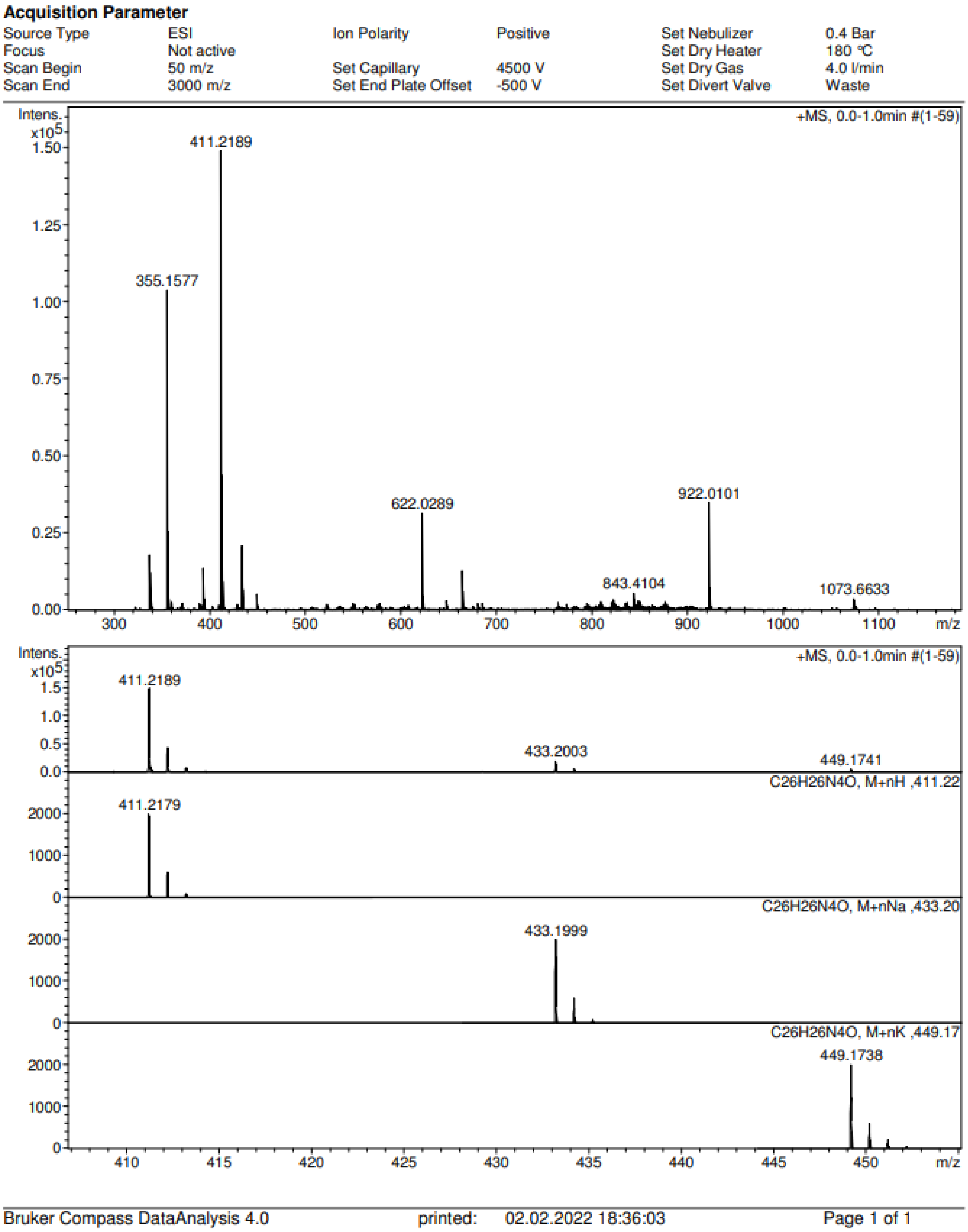

HRMS for compound **15e**

Acquisition Parameter

Source Type	ESI	Ion Polarity	Positive	Set Nebulizer	0.4 Bar
Focus	Not active			Set Dry Heater	180 °C
Scan Begin	50 m/z	Set Capillary	4500 V	Set Dry Gas	4.0 l/min
Scan End	3000 m/z	Set End Plate Offset	-500 V	Set Divert Valve	Waste

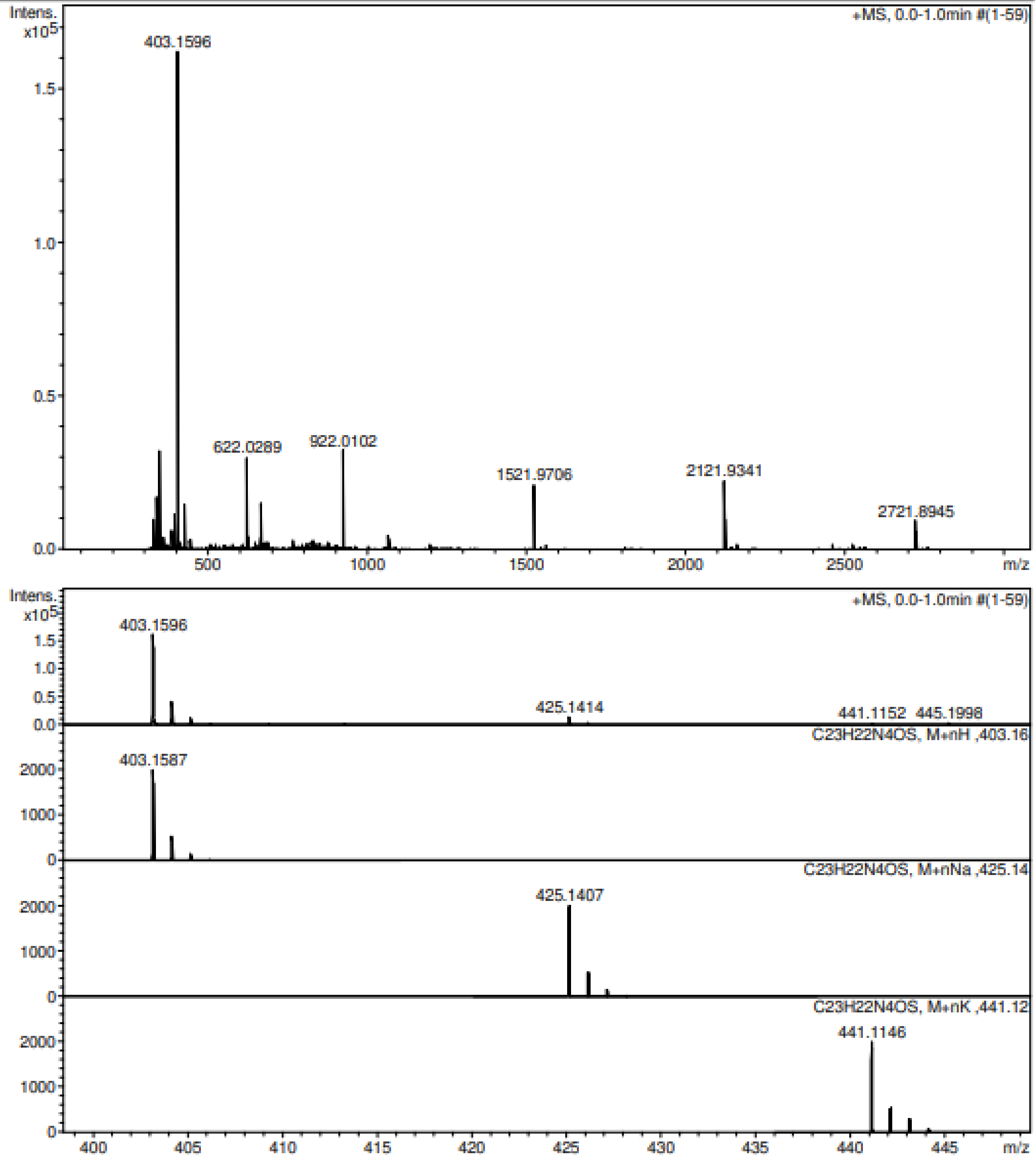

Bruker Compass DataAnalysis 4.0 printed: 02.02.2022 18:40:08 Page 1 of 1

HRMS for compound **15f**

Acquisition Parameter

Source Type	ESI	Ion Polarity	Positive	Set Nebulizer	1.0 Bar
Focus	Not active			Set Dry Heater	200 °C
Scan Begin	50 m/z	Set Capillary	4500 V	Set Dry Gas	4.0 l/min
Scan End	1600 m/z	Set End Plate Offset	-500 V	Set Divert Valve	Waste

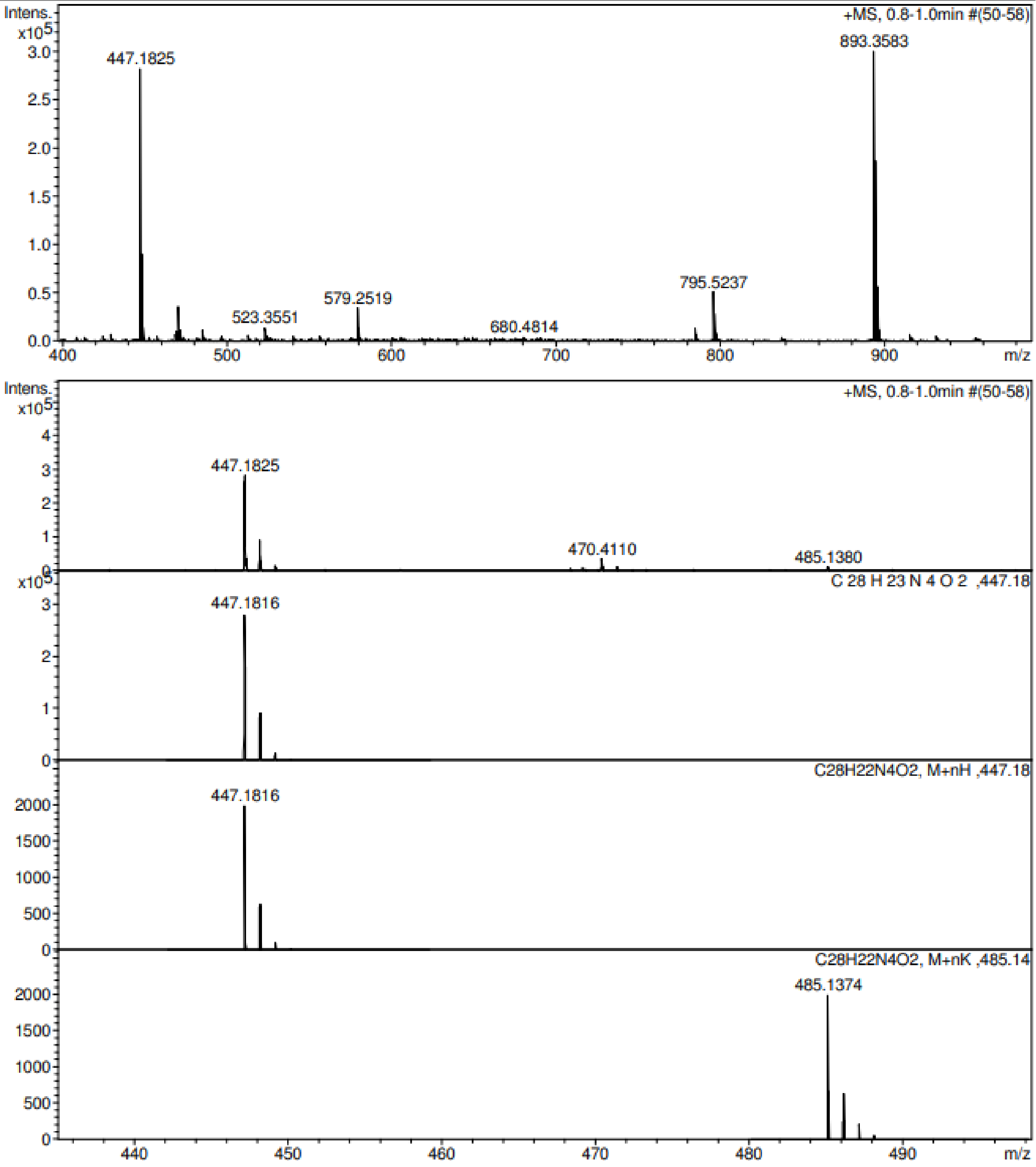

Bruker Compass DataAnalysis 4.0 printed: 04.03.2020 11:48:20 Page 1 of 1

HRMS for compound **15g**

Acquisition Parameter

Source Type	ESI	Ion Polarity	Positive	Set Nebulizer	0.4 Bar
Focus	Not active			Set Dry Heater	180 °C
Scan Begin	50 m/z	Set Capillary	4500 V	Set Dry Gas	4.0 l/min
Scan End	3000 m/z	Set End Plate Offset	-500 V	Set Divert Valve	Waste

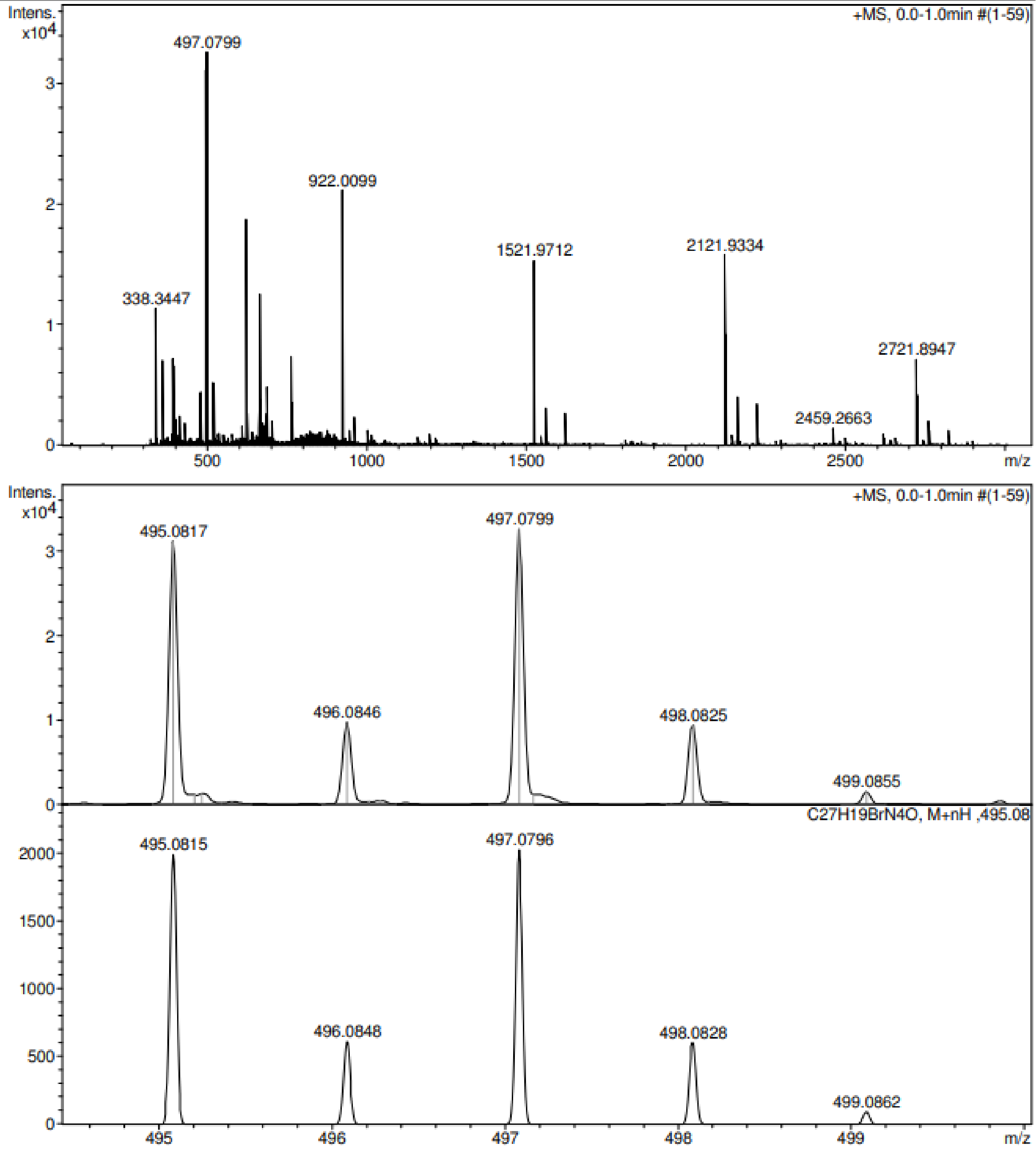

Bruker Compass DataAnalysis 4.0 printed: 27.01.2022 14:38:58 Page 1 of 1

HRMS for compound **15h**

Acquisition Parameter

Source Type	ESI	Ion Polarity	Positive	Set Nebulizer	0.4 Bar
Focus	Not active			Set Dry Heater	180 °C
Scan Begin	50 m/z	Set Capillary	4500 V	Set Dry Gas	4.0 l/min
Scan End	3000 m/z	Set End Plate Offset	-500 V	Set Divert Valve	Waste

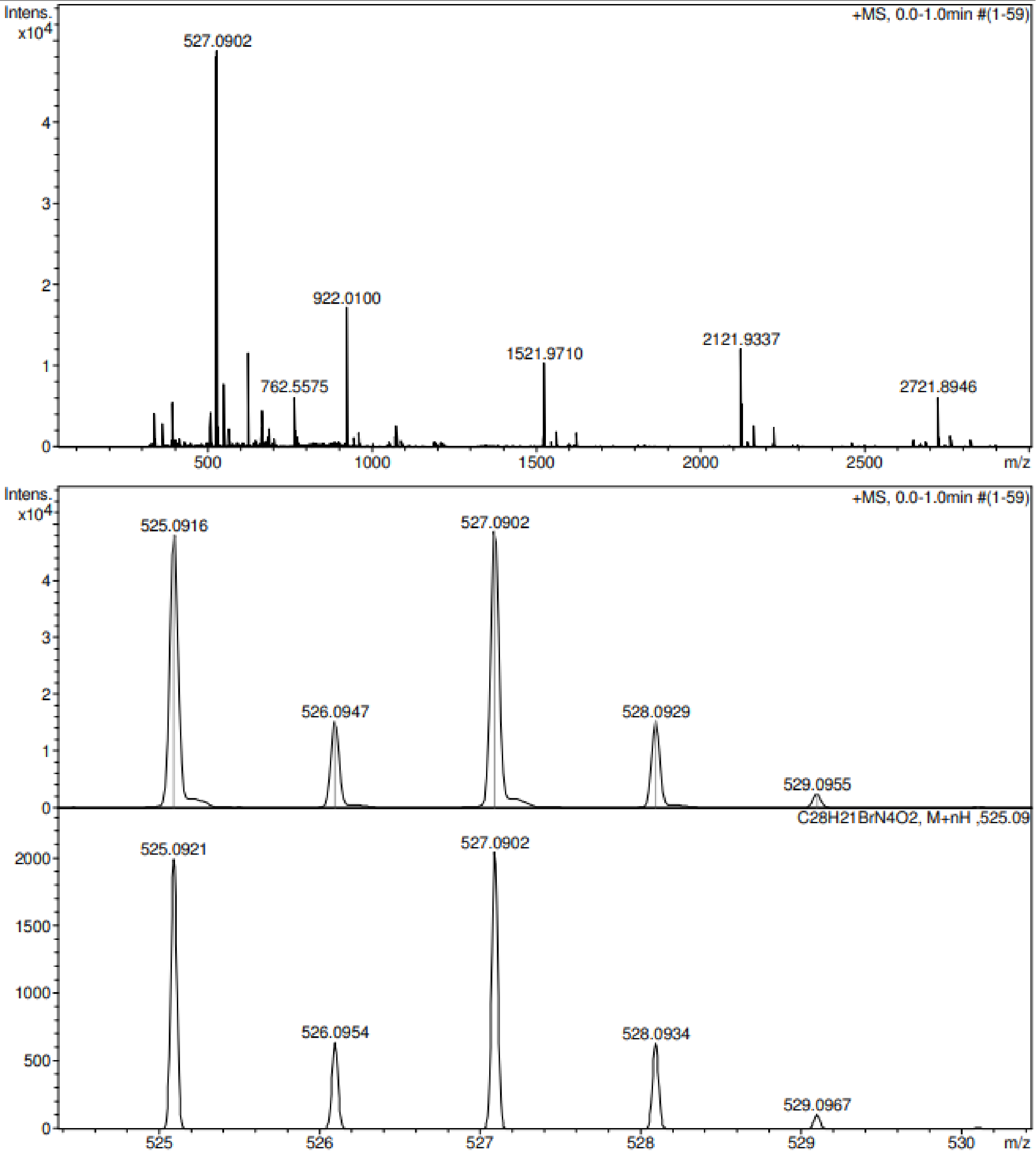

Bruker Compass DataAnalysis 4.0 printed: 27.01.2022 14:32:21 Page 1 of 1

HRMS for compound **15i**

Acquisition Parameter

Source Type	ESI	Ion Polarity	Positive	Set Nebulizer	0.4 Bar
Focus	Not active			Set Dry Heater	180 °C
Scan Begin	50 m/z	Set Capillary	4500 V	Set Dry Gas	4.0 l/min
Scan End	3000 m/z	Set End Plate Offset	-500 V	Set Divert Valve	Waste

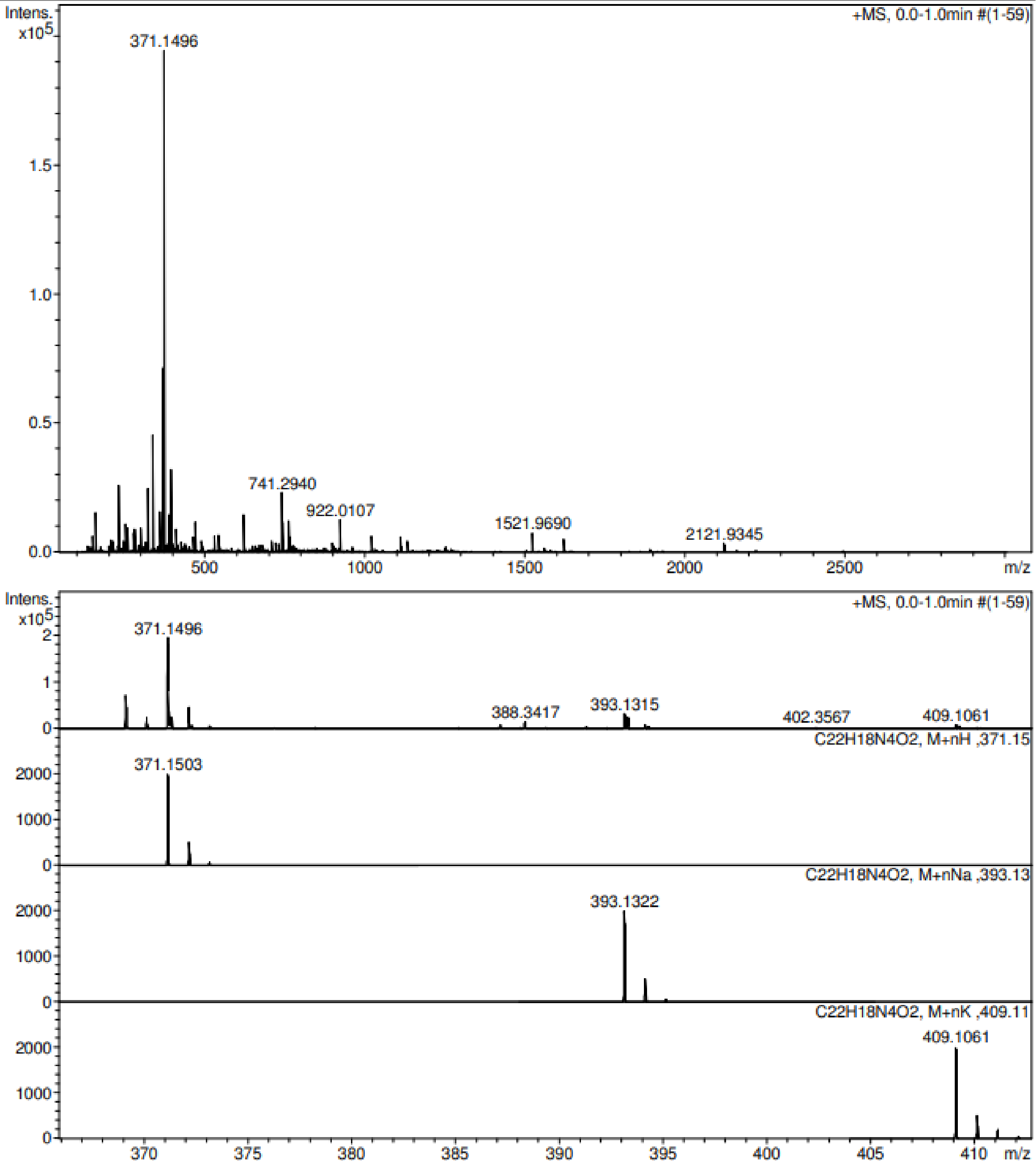

Bruker Compass DataAnalysis 4.0 printed: 01.02.2022 17:05:55 Page 1 of 1

HRMS for compound **15j**

Acquisition Parameter

Source Type	ESI	Ion Polarity	Positive	Set Nebulizer	0.4 Bar
Focus	Not active			Set Dry Heater	180 °C
Scan Begin	50 m/z	Set Capillary	4500 V	Set Dry Gas	4.0 l/min
Scan End	3000 m/z	Set End Plate Offset	-500 V	Set Divert Valve	Waste

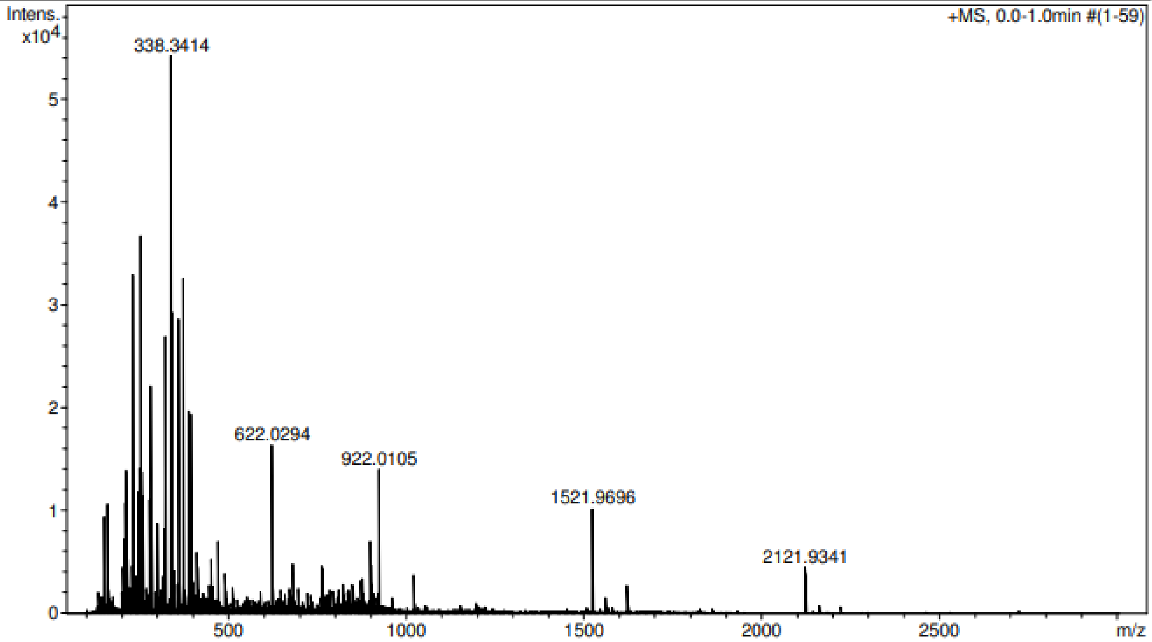

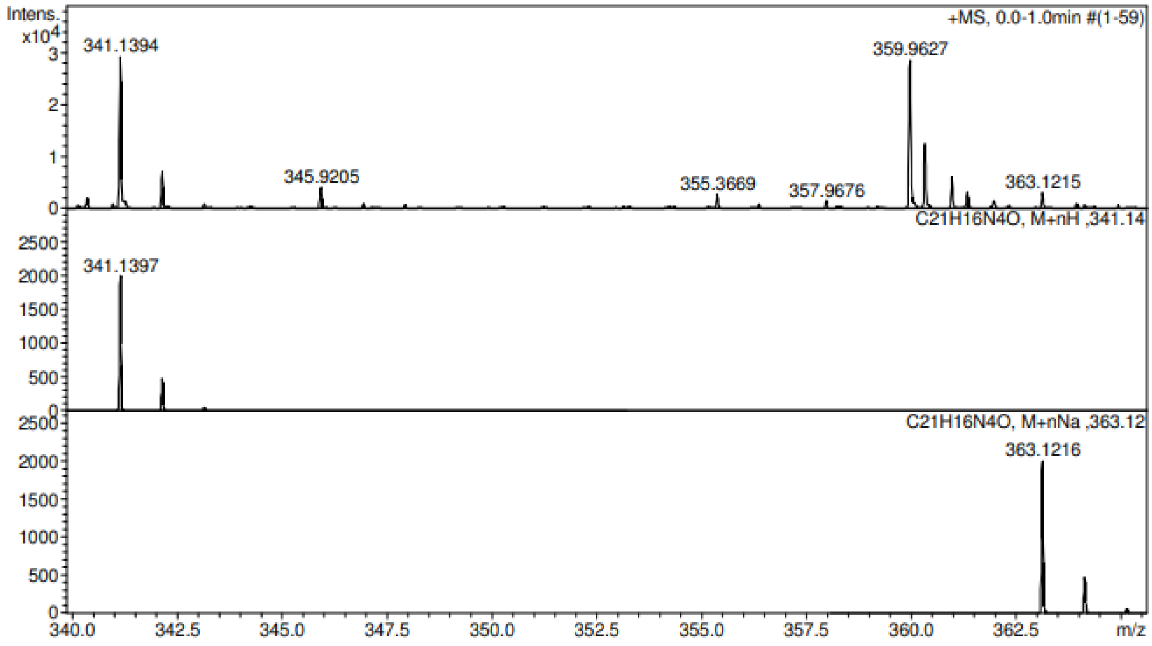

HRMS for compound **15k**

Acquisition Parameter

Source Type	ESI	Ion Polarity	Positive	Set Nebulizer	0.4 Bar
Focus	Not active			Set Dry Heater	180 °C
Scan Begin	50 m/z	Set Capillary	4500 V	Set Dry Gas	4.0 l/min
Scan End	3000 m/z	Set End Plate Offset	-500 V	Set Divert Valve	Waste

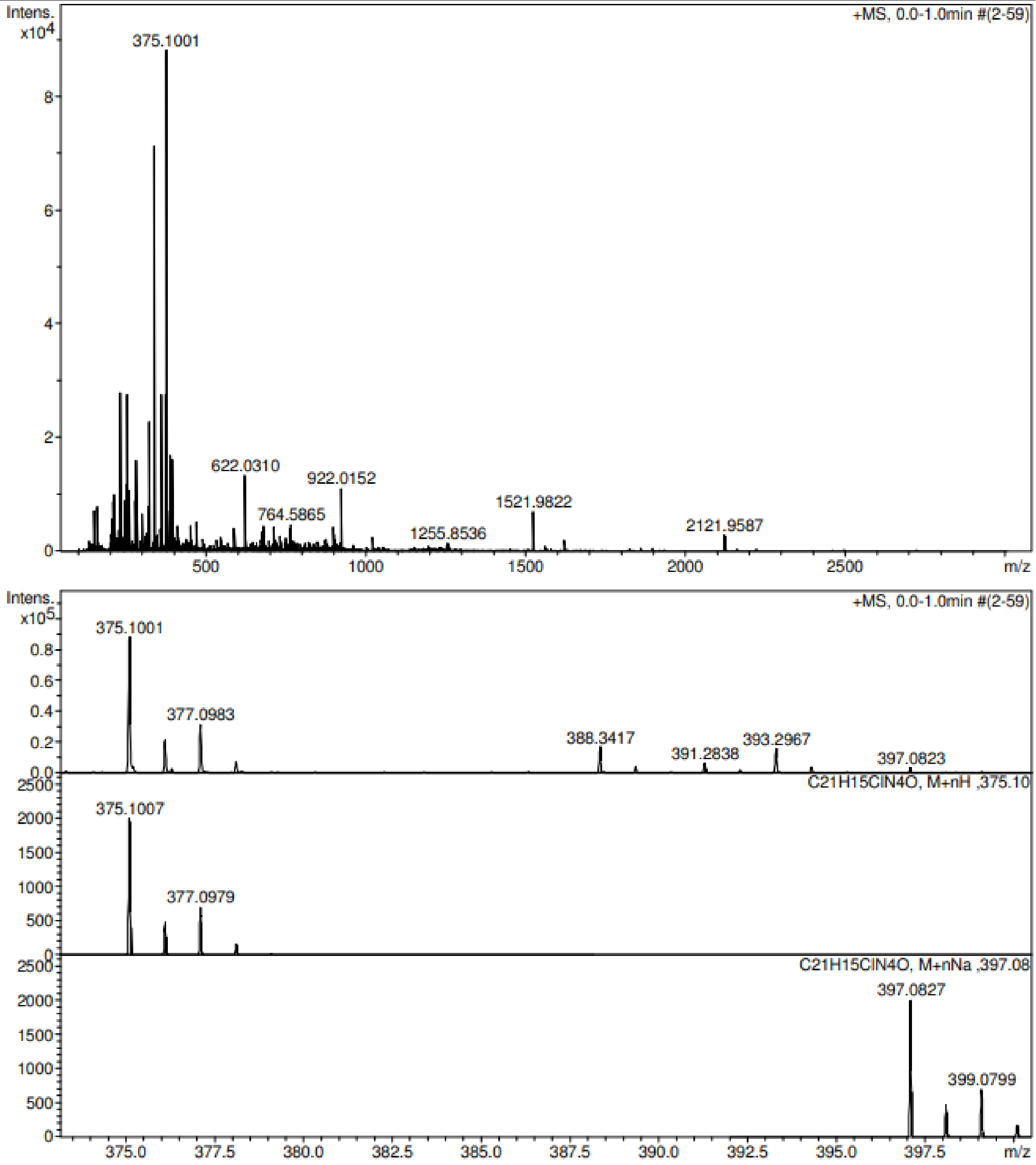

Bruker Compass DataAnalysis 4.0 printed: 01.02.2022 17:10:04 Page 1 of 1

HRMS for compound **15l**

Acquisition Parameter

Source Type	ESI	Ion Polarity	Positive	Set Nebulizer	0.4 Bar
Focus	Not active			Set Dry Heater	180 °C
Scan Begin	50 m/z	Set Capillary	4500 V	Set Dry Gas	4.0 l/min
Scan End	3000 m/z	Set End Plate Offset	-500 V	Set Divert Valve	Waste

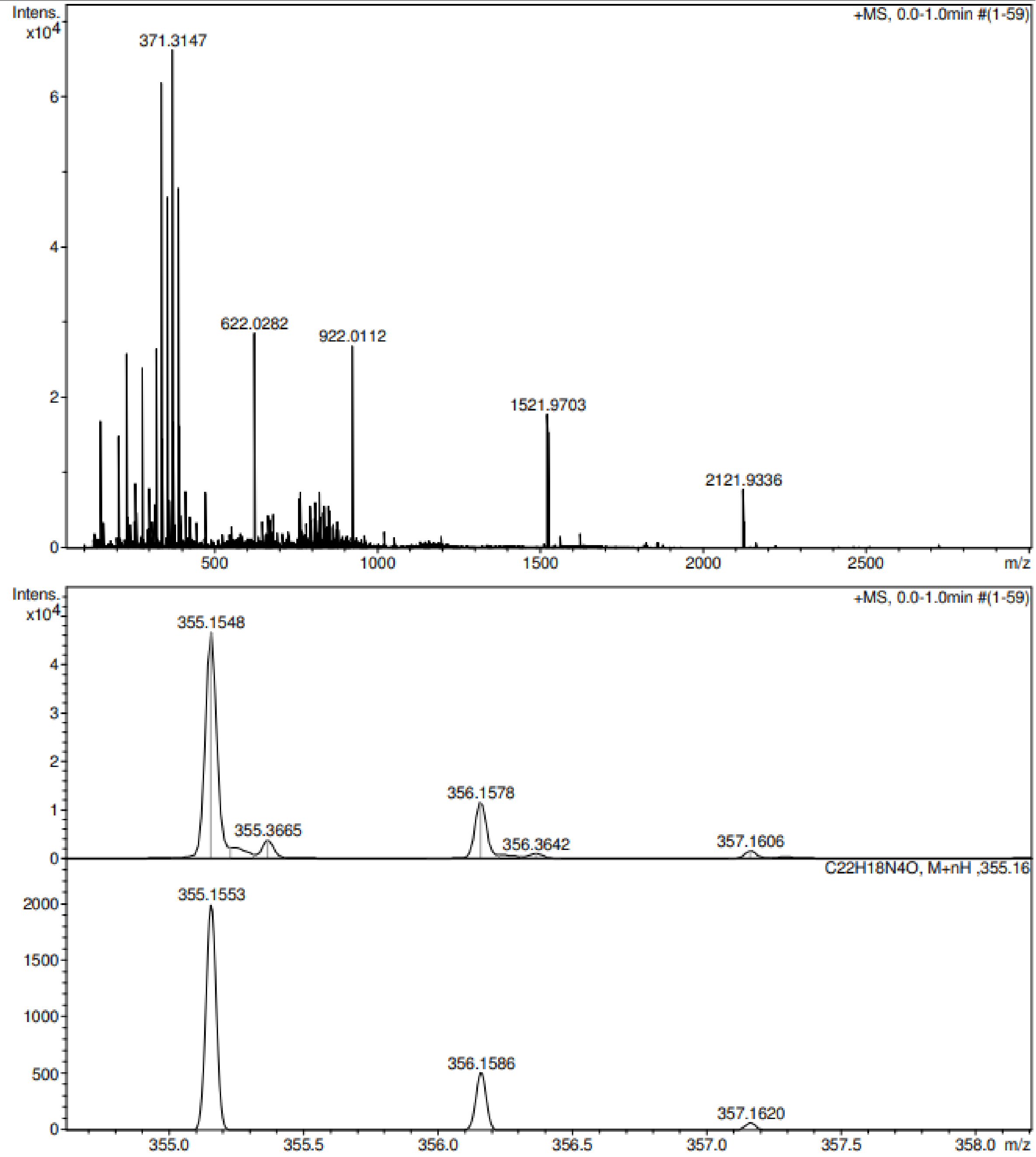

Bruker Compass DataAnalysis 4.0 printed: 04.02.2022 14:01:14 Page 1 of 1

HRMS for compound **15m**

Acquisition Parameter

Source Type	ESI	Ion Polarity	Positive	Set Nebulizer	0.4 Bar
Focus	Not active			Set Dry Heater	180 °C
Scan Begin	50 m/z	Set Capillary	4500 V	Set Dry Gas	4.0 l/min
Scan End	3000 m/z	Set End Plate Offset	-500 V	Set Divert Valve	Waste

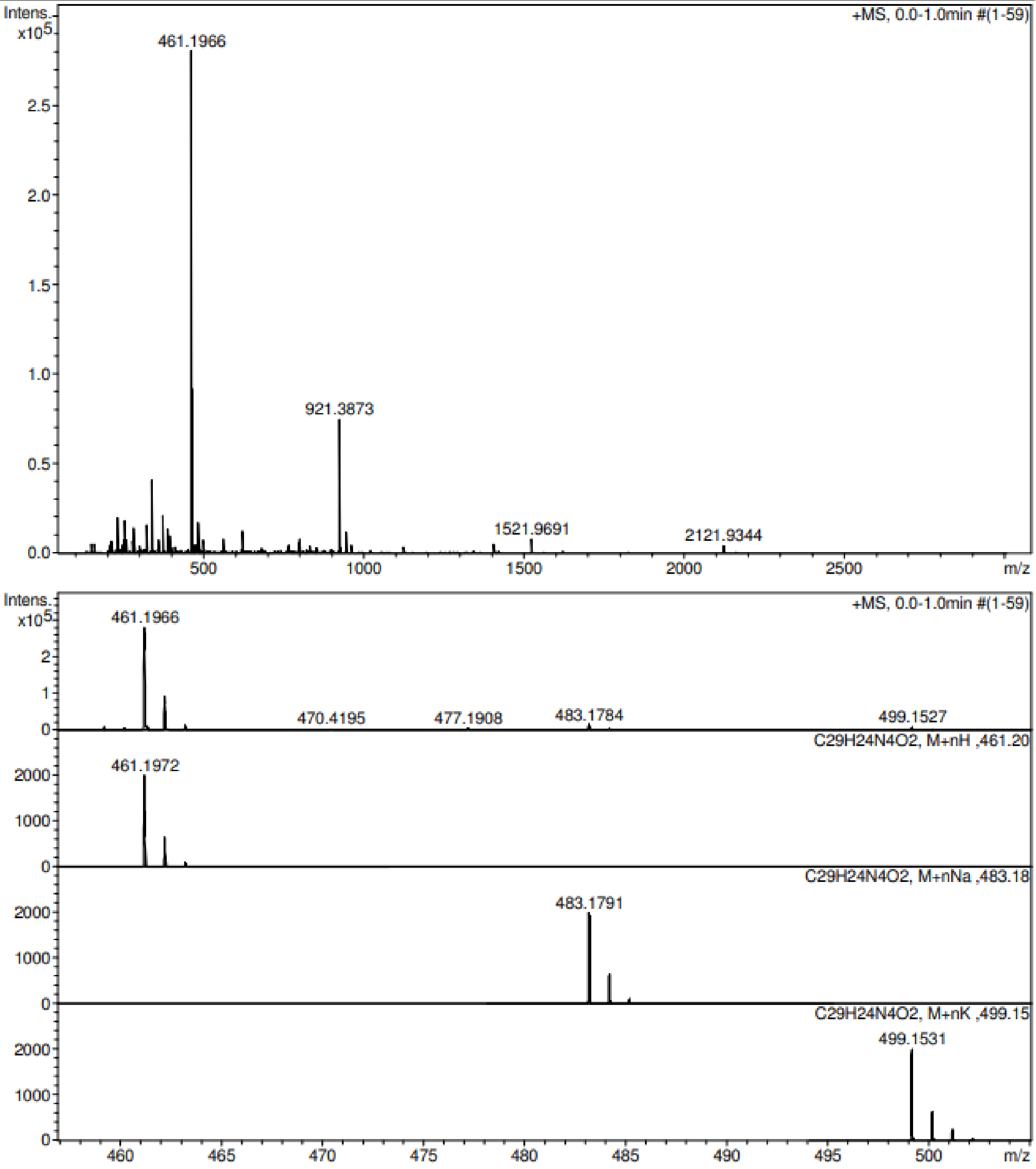

Bruker Compass DataAnalysis 4.0 printed: 01.02.2022 17:12:12 Page 1 of 1

HRMS for compound **15n**

Acquisition Parameter

Source Type	ESI	Ion Polarity	Positive	Set Nebulizer	0.4 Bar
Focus	Not active			Set Dry Heater	180 °C
Scan Begin	50 m/z	Set Capillary	4500 V	Set Dry Gas	4.0 l/min
Scan End	3000 m/z	Set End Plate Offset	-500 V	Set Divert Valve	Waste

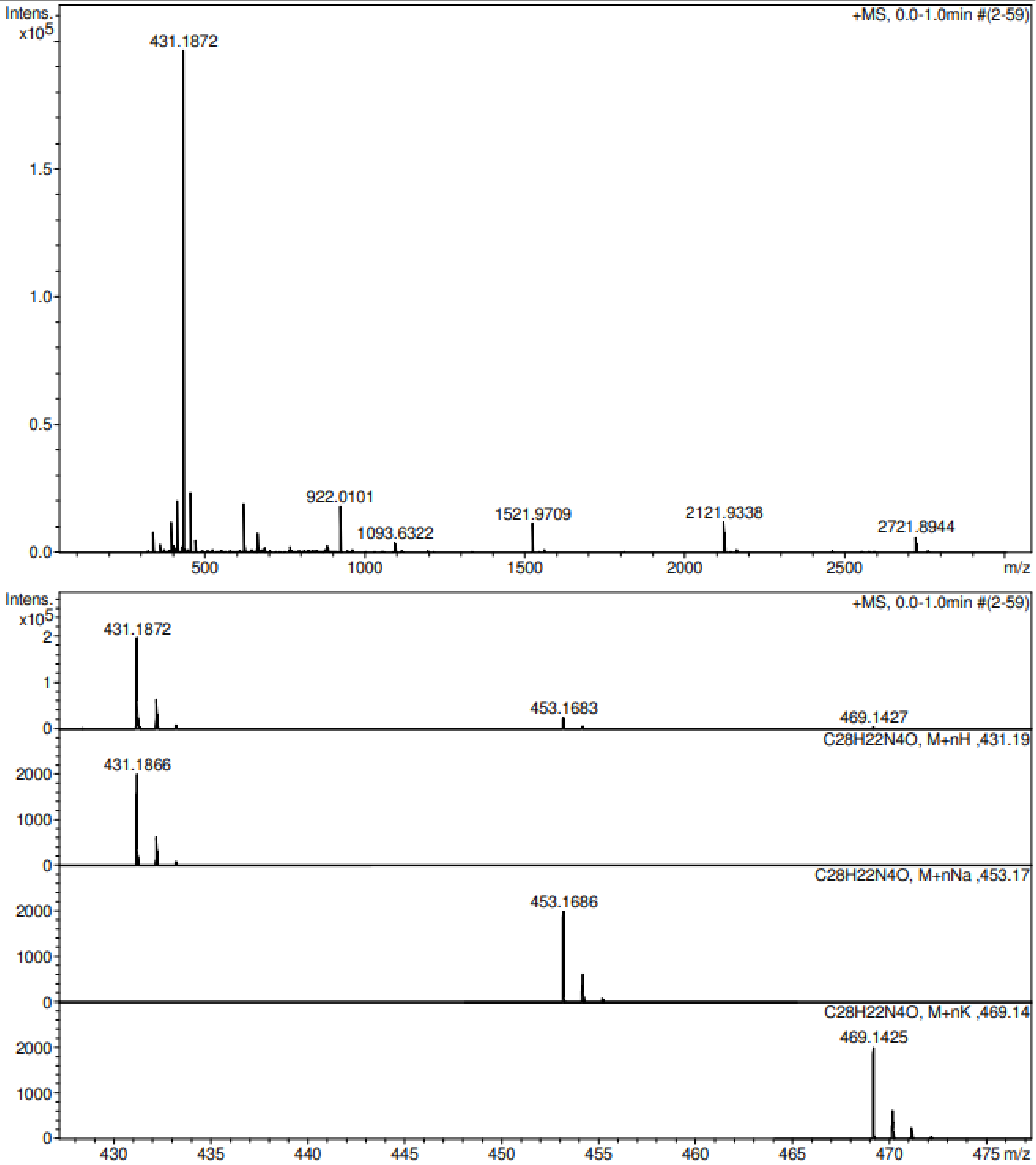

Bruker Compass DataAnalysis 4.0 printed: 02.02.2022 18:42:35 Page 1 of 1

HRMS for compound **15o**

Analysis Info

Analysis Name	D:\Data\Chizhov\Krayushkin\Milyutin\Feb_02_2022\425qhi_&clb.d	Acquisition Date	02.02.2022 18:16:45
Method	tune_wide.m	Operator	BDAL@DE
Sample Name	/BALT 425.QHI	Instrument / Ser#	micrOTOF 10248
Comment	CH3CN 100 %, dil. 2000, calibrant added		

Acquisition Parameter

Source Type	ESI	Ion Polarity	Positive	Set Nebulizer	0.4 Bar
Focus	Not active			Set Dry Heater	180 °C
Scan Begin	50 m/z	Set Capillary	4500 V	Set Dry Gas	4.0 l/min
Scan End	3000 m/z	Set End Plate Offset	-500 V	Set Divert Valve	Waste

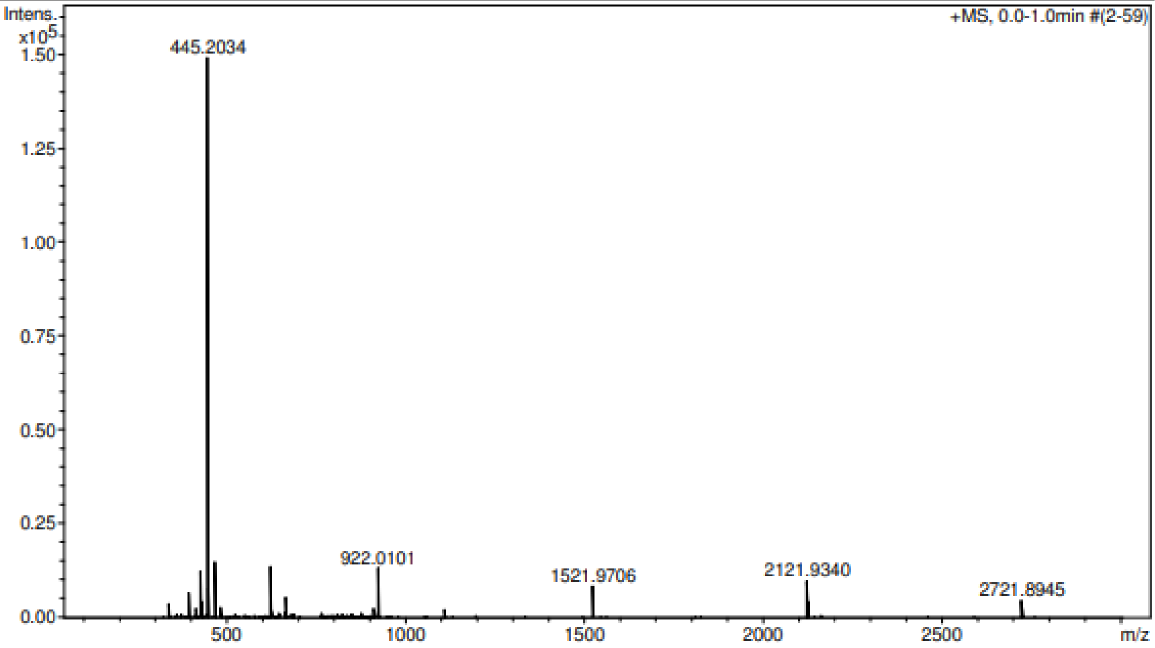

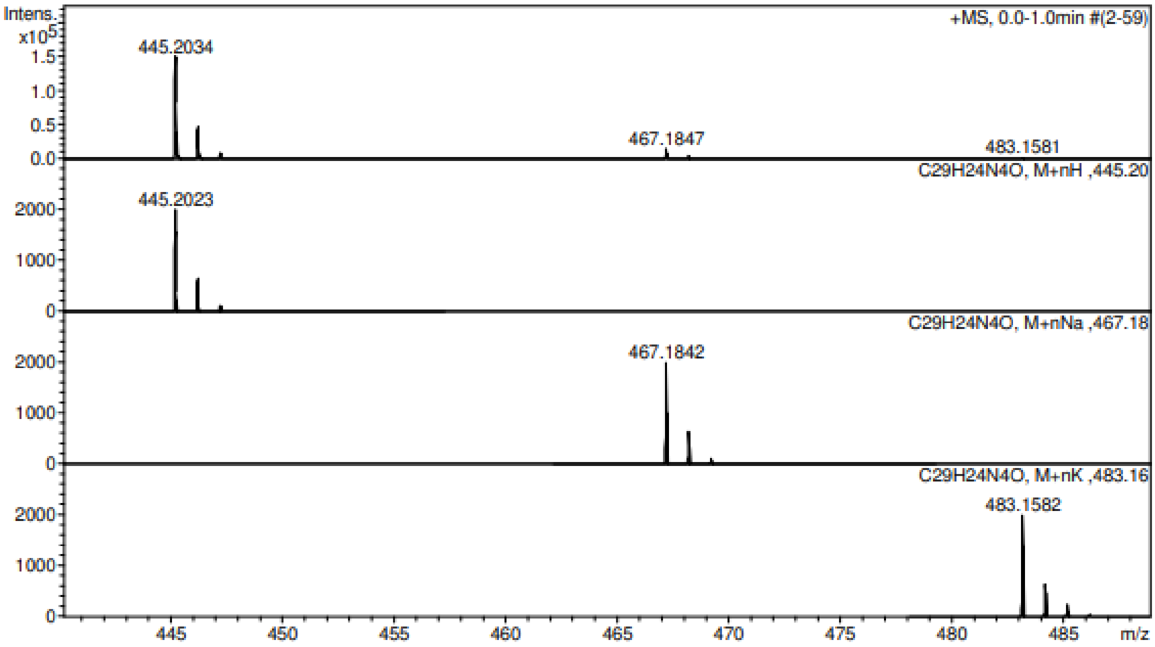

HRMS for compound **18**

Acquisition Parameter

Source Type	ESI	Ion Polarity	Positive	Set Nebulizer	0.4 Bar
Focus	Not active			Set Dry Heater	180 °C
Scan Begin	50 m/z	Set Capillary	4500 V	Set Dry Gas	4.0 l/min
Scan End	3000 m/z	Set End Plate Offset	-500 V	Set Divert Valve	Waste

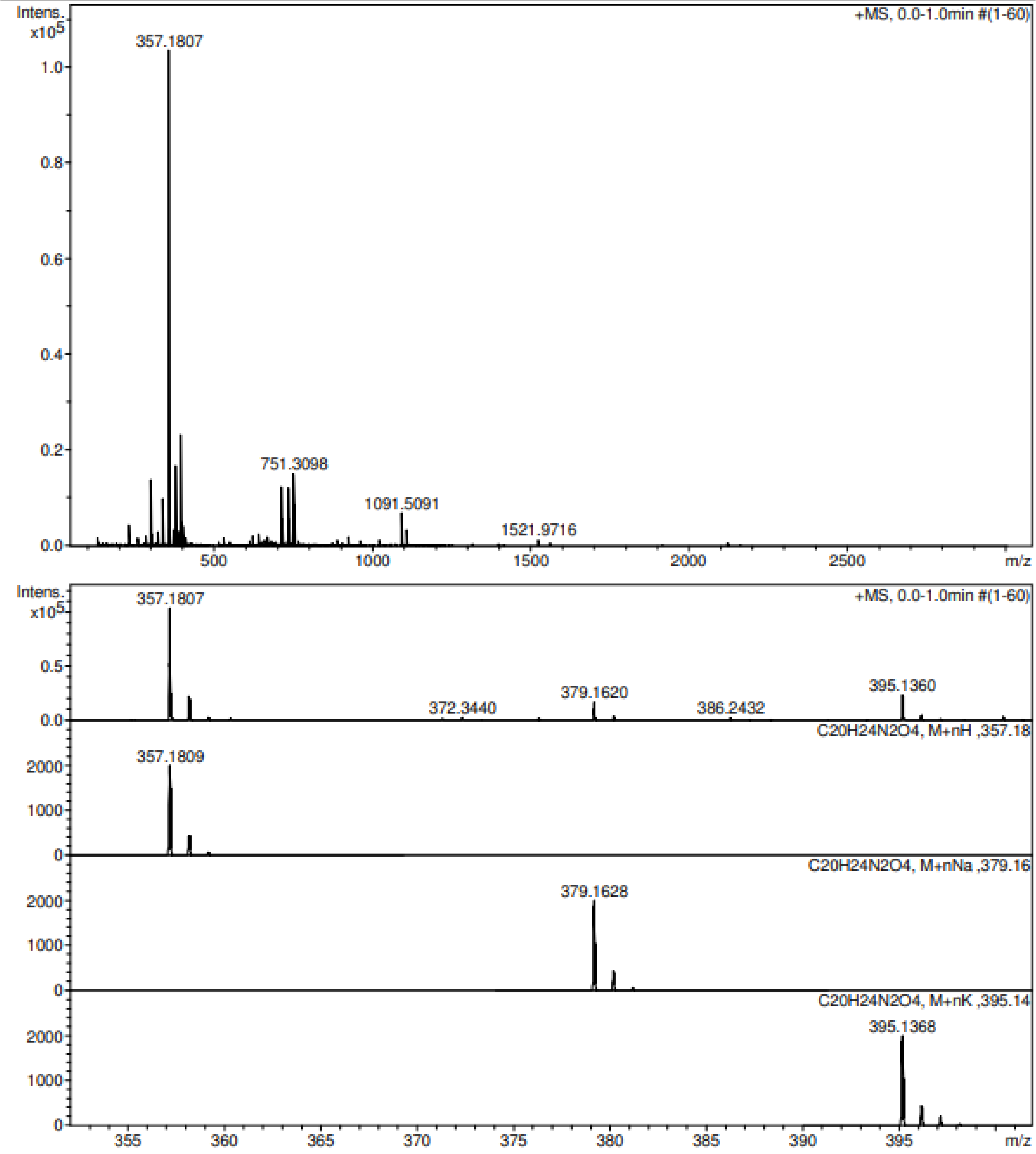

Bruker Compass DataAnalysis 4.0 printed: 28.02.2022 14:29:15 Page 1 of 1

8. 2D NMR spectra for compound **14a**

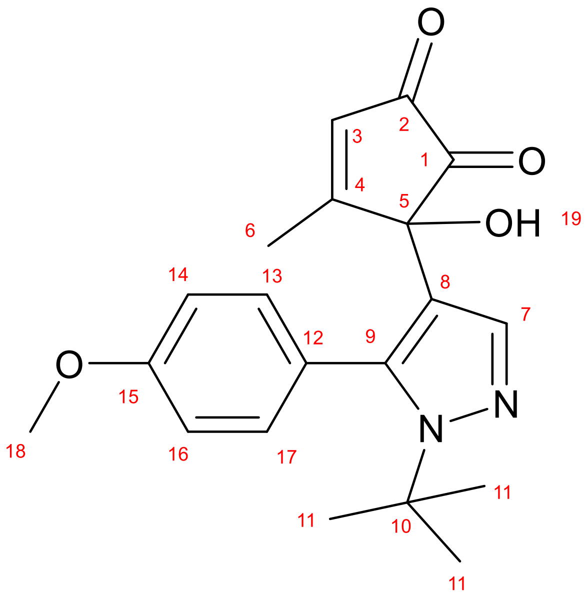

Table S1. Assignment of NMR signals and 2D NMR (HMBC) correlations for compound **13a**

№	^{1}H	^{13}C	HMBC
1		201.1	3, 19
2		186.1	3, 6
3	6.47 (q, *J* = 1.4 Hz, 1H)	135.3	6
4		176.8	3, 6
5		72.43	3, 6, 19
6	1.94 (d, *J* = 1.3 Hz, 3H)	14.6	2, 3, 4, 5
7	7.62 (s, 1H)	135.5	----
8		119.4	7, 19
9		137.6	7, 13, 17, 19
10		61.2	11
11	1.30 (s, 9H)	30.6	11
12		122.5	14, 16
13	7.02 (dd, *J* = 8.5, 2.3 Hz, 1H)	132.3	17
14	6.86 (dd, *J* = 8.5, 2.8 Hz, 1H)	113.5	16

15		159.7	13, 17, 18
16	6.78 (dd, *J* = 8.5, 2.8 Hz, 1H)	113.5	14
17	6.68 (dd, *J* = 8.4, 2.3 Hz, 1H)	132.7	13
18	3.75 (s, 3H)	55.3	
19	6.48 (s, 1H)	----	------

HMBC spectrum for compound **14a**

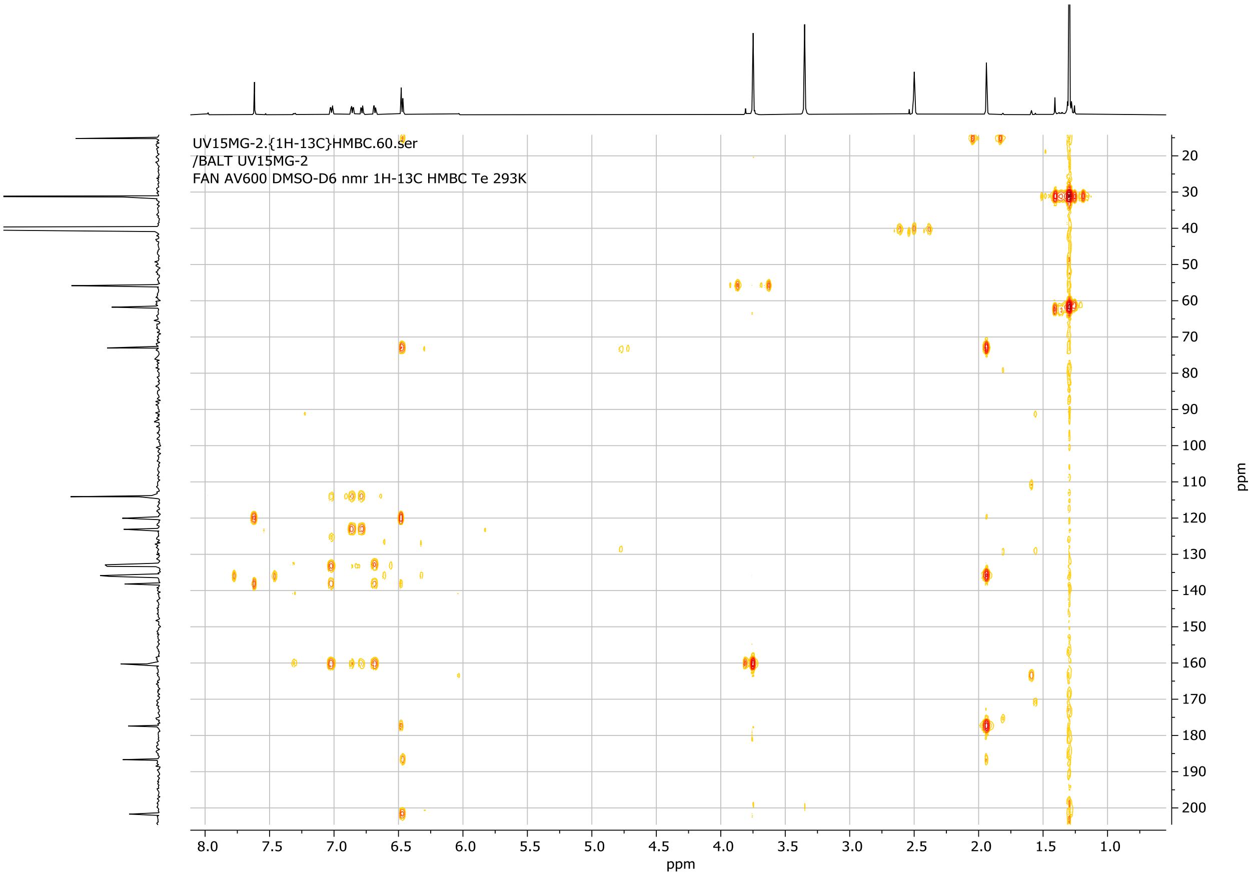

HSQC spectrum for compound **14a**

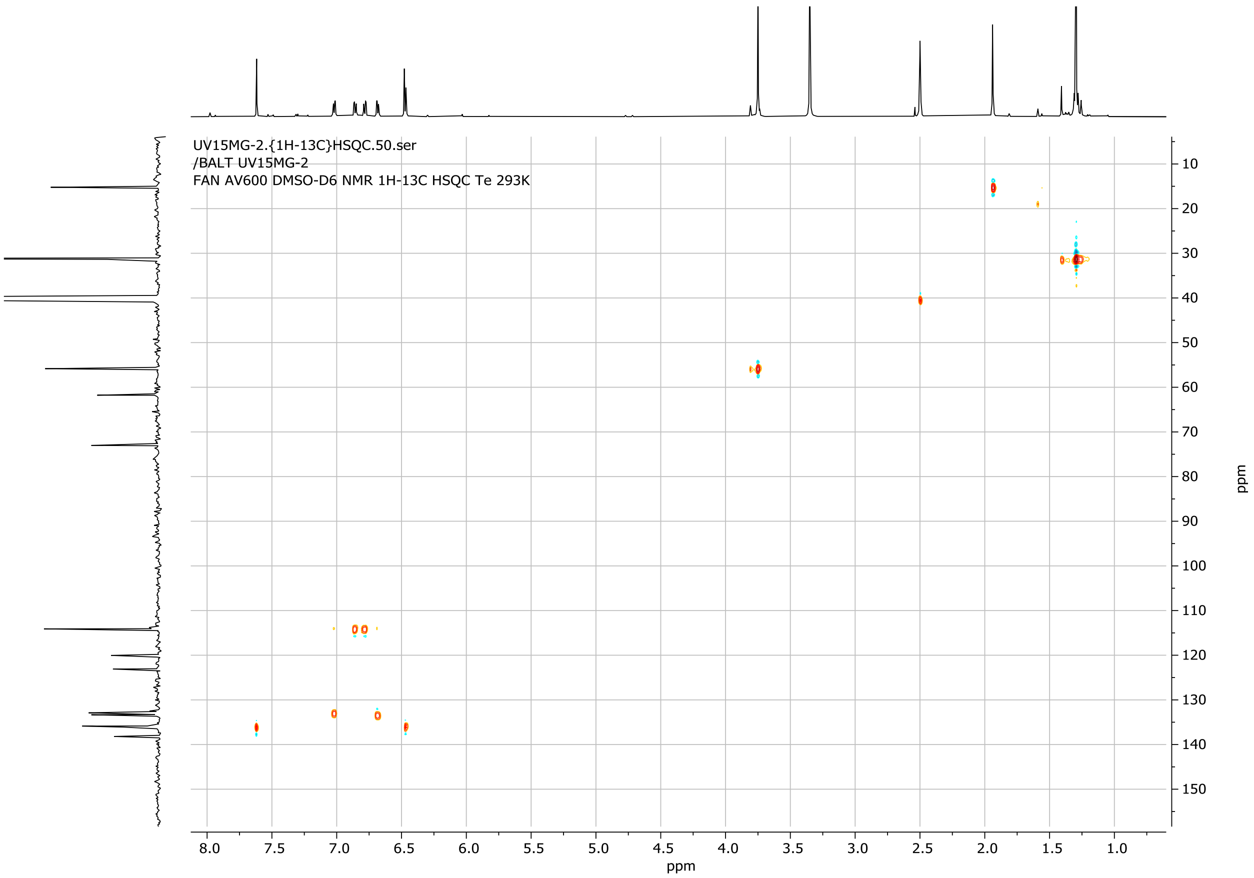

COSY spectrum for compound **14a**

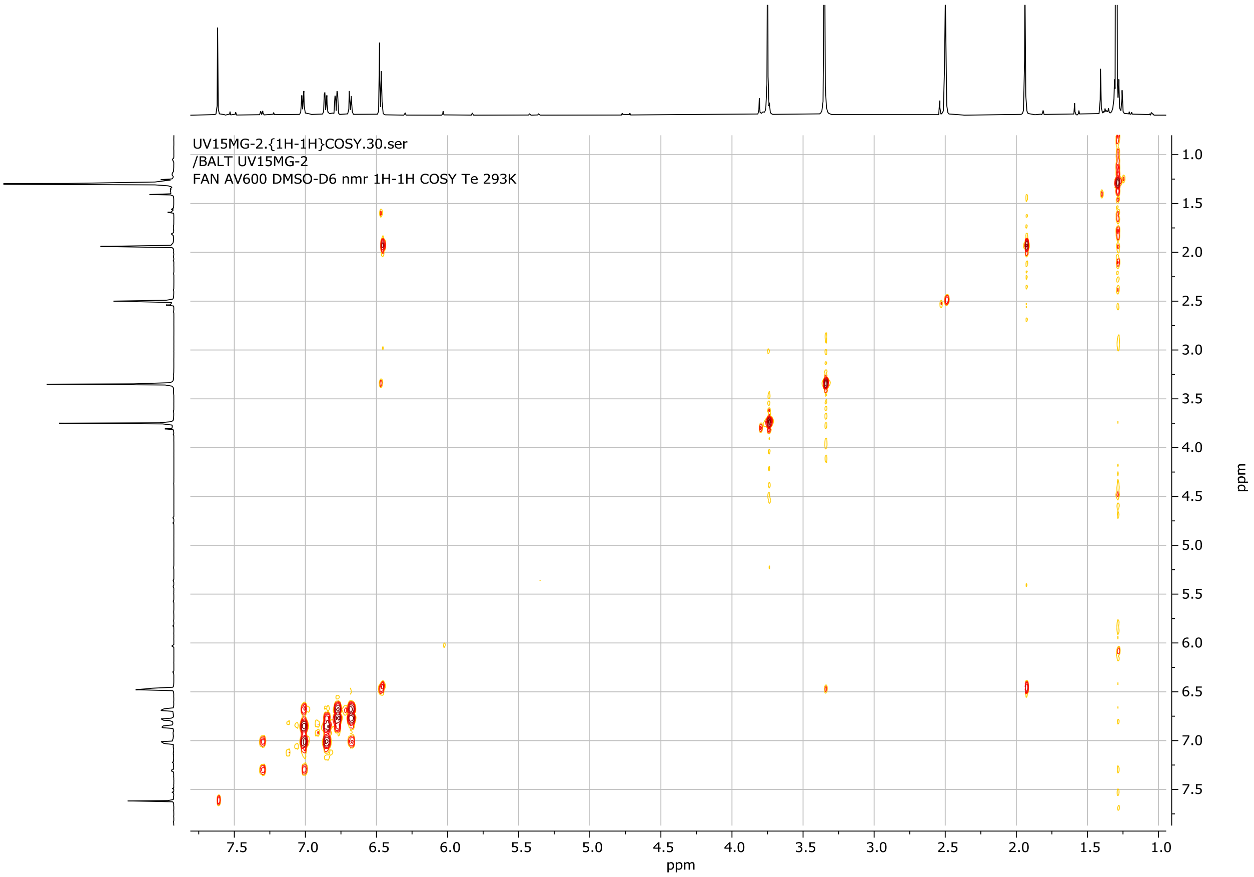

9. X-ray crystallographic data and refinement details

Crystal structure determination was performed in the Department of Structural Studies of Zelinsky Institute of Organic Chemistry, Moscow.

X-ray crystallographic data for compound 15a:

X-ray diffraction data were collected at 100K on a four-circle Rigaku Synergy S diffractometer equipped with a HyPix600HE area-detector (kappa geometry, shutterless ω-scan technique), using monochromatized Cu K□-radiation. The intensity data were integrated and corrected for absorption and decay by the CrysAlisPro program[3]. The structure was solved by direct methods using SHELXT[4] and refined on F^4 using SHELXL-2018[5] in the OLEX2 program.[5] All non-hydrogen atoms were refined with individual anisotropic displacement parameters. Locations of hydrogen atoms at oxygen atoms O1, O3, O5 and O6 were found from the electron density-difference map; these hydrogen atoms were refined with individual isotropic displacement parameters. All other hydrogen atoms were placed in ideal calculated positions and refined as riding atoms with relative isotropic displacement parameters. Site occupancies for peroxide molecules are 0.882(7) for O5-O6 and 0.103(6) for O7-O8.

Table S2. Crystal data and structure refinement for **15a**.

Identification code	**15a**
Empirical formula	C26 H26.99 N4 O2.99
Formula weight	443.26
Temperature	100.0(1) K
Wavelength	1.54184 Å
Crystal system	Triclinic
Space group	$P\bar{1}$
Unit cell dimensions	a = 6.59886(6) Å □=64.7444(15)°.<br>b = 19.0031(3) Å □= 85.5729(9)°.<br>c = 20.1060(3) Å □ = 88.7649(9)°.
Volume	2273.28(6) $Å^3$
Z	4
Density (calculated)	1.295 g/cm^3
Absorption coefficient	0.696 mm^{-1}
F(000)	939.5
Crystal size	0.468 x 0.074 x 0.061 mm^3
Theta range for data collection	2.437 to 78.973°.
Index ranges	-8<=h<=7, -24<=k<=24, -25<=l<=25
Reflections collected	11269
Independent reflections	11269 [R(int) = 0]
Observed reflections	9934
Completeness to theta = 67.684°	99.9 %
Absorption correction	Analytical
Max. and min. transmission	0.964 and 0.767
Refinement method	Full-matrix least-squares on F^2
Data / restraints / parameters	11269 / 7 / 642
Goodness-of-fit on F^2	1.088
Final R indices [I>2sigma(I)]	R1 = 0.0650, wR2 = 0.1804
R indices (all data)	R1 = 0.0716, wR2 = 0.1839
Largest diff. peak and hole	0.392 and -0.352 $e.Å^{-3}$
CCDC	2159683

Table S3. Atomic coordinates (x 10^4) and equivalent isotropic displacement parameters ($Å^2$x 10^3) for **15a**. U(eq) is defined as one third of the trace of the orthogonalized U^{ij} tensor.

	x	y	z	U(eq)
O(1)	7420(3)	1558(1)	4321(1)	22(1)
O(2)	13803(4)	180(2)	7865(2)	59(1)
N(1)	7382(4)	2752(1)	5833(1)	27(1)
N(2)	6291(4)	3091(1)	5234(1)	28(1)
N(3)	10631(3)	-440(1)	6155(1)	22(1)
N(4)	6679(3)	261(1)	5896(1)	22(1)
C(1)	8382(4)	2110(1)	5826(1)	24(1)
C(2)	7850(4)	2041(1)	5204(1)	22(1)
C(3)	6571(4)	2665(1)	4860(1)	25(1)
C(4)	8562(4)	1437(1)	4939(1)	21(1)
C(5)	10869(4)	1494(1)	4738(1)	23(1)
C(6)	11814(4)	834(1)	5147(1)	23(1)
C(7)	10330(4)	269(1)	5664(1)	20(1)
C(8)	8893(4)	-822(1)	6576(1)	22(1)
C(9)	9070(4)	-1570(2)	7148(1)	26(1)
C(10)	7367(4)	-1971(2)	7566(1)	27(1)
C(11)	5434(4)	-1639(2)	7417(2)	29(1)
C(12)	5219(4)	-903(2)	6869(1)	26(1)
C(13)	6941(4)	-481(1)	6439(1)	22(1)
C(14)	8358(4)	610(1)	5544(1)	20(1)
C(15)	11821(4)	2231(2)	4171(2)	27(1)
C(16)	7538(5)	3180(2)	6303(2)	33(1)
C(17)	8198(6)	2645(2)	7063(2)	47(1)
C(18)	9038(7)	3846(2)	5904(2)	55(1)
C(19)	5418(6)	3490(2)	6400(2)	49(1)
C(20)	9747(4)	1594(2)	6386(1)	27(1)
C(21)	11822(5)	1769(2)	6306(2)	35(1)
C(22)	13116(5)	1279(2)	6809(2)	44(1)
C(23)	12374(5)	608(2)	7398(2)	42(1)
C(24)	10330(6)	418(2)	7483(2)	40(1)
C(25)	9027(5)	913(2)	6972(2)	32(1)
C(26)	13131(7)	-550(2)	8449(2)	60(1)
O(3)	2434(3)	3486(1)	10777(1)	23(1)
O(4)	-1002(4)	5140(1)	6826(1)	41(1)
N(5)	2161(4)	1961(1)	9590(1)	33(1)
N(6)	2811(4)	1611(1)	10279(1)	38(1)

N(7)	-437(3)	5391(1)	8784(1)	22(1)
N(8)	3386(3)	4614(1)	9146(1)	23(1)
C(27)	1555(4)	2709(2)	9423(1)	26(1)
C(28)	1867(4)	2839(1)	10036(1)	23(1)
C(29)	2639(5)	2141(2)	10544(2)	32(1)
C(30)	1314(4)	3543(1)	10176(1)	22(1)
C(31)	-1002(4)	3561(1)	10343(1)	23(1)
C(32)	-1823(4)	4218(1)	9867(1)	23(1)
C(33)	-250(4)	4709(1)	9334(1)	21(1)
C(34)	1349(4)	5704(1)	8354(1)	22(1)
C(35)	1272(4)	6407(2)	7718(1)	26(1)
C(36)	3014(4)	6734(2)	7277(2)	28(1)
C(37)	4881(4)	6360(2)	7469(2)	30(1)
C(38)	5002(4)	5675(2)	8087(2)	28(1)
C(39)	3231(4)	5326(1)	8540(1)	22(1)
C(40)	1657(4)	4322(1)	9504(1)	21(1)
C(41)	-2093(4)	2889(2)	10952(2)	29(1)
C(42)	2122(6)	1501(2)	9145(2)	43(1)
C(43)	-86(7)	1446(2)	8987(3)	56(1)
C(44)	3450(9)	1895(3)	8434(3)	71(2)
C(45)	2852(11)	687(2)	9600(3)	71(2)
C(43A)	800(50)	1820(20)	8489(15)	56(1)
C(44A)	4346(19)	1500(30)	8900(20)	71(2)
C(45A)	1410(60)	697(12)	9698(18)	71(2)
C(46)	840(4)	3284(2)	8712(1)	27(1)
C(47)	-1204(4)	3470(2)	8611(2)	29(1)
C(48)	-1766(5)	4085(2)	7977(2)	33(1)
C(49)	-294(5)	4530(2)	7427(2)	31(1)
C(50)	1735(5)	4347(2)	7509(2)	34(1)
C(51)	2273(5)	3731(2)	8151(2)	32(1)
C(52)	481(6)	5618(2)	6258(2)	49(1)
O(5)	4353(6)	4494(2)	4421(2)	62(1)
O(6)	4730(5)	4398(2)	3735(2)	52(1)
O(7)	6880(40)	9545(14)	8207(13)	55(6)
O(8)	7100(40)	9892(14)	8725(13)	55(6)

Table S4. Bond lengths [Å] and angles [°] for **15a**.

O(1)-H(1O)	0.89(3)	C(16)-C(18)	1.513(5)
O(1)-C(4)	1.434(3)	C(16)-C(19)	1.538(4)
O(2)-C(23)	1.373(4)	C(17)-H(17A)	0.9800
O(2)-C(26)	1.433(5)	C(17)-H(17B)	0.9800
N(1)-N(2)	1.354(3)	C(17)-H(17C)	0.9800
N(1)-C(1)	1.377(3)	C(18)-H(18A)	0.9800
N(1)-C(16)	1.498(3)	C(18)-H(18B)	0.9800
N(2)-C(3)	1.323(3)	C(18)-H(18C)	0.9800
N(3)-C(7)	1.309(3)	C(19)-H(19A)	0.9800
N(3)-C(8)	1.386(3)	C(19)-H(19B)	0.9800
N(4)-C(13)	1.384(3)	C(19)-H(19C)	0.9800
N(4)-C(14)	1.296(3)	C(20)-C(21)	1.397(4)
C(1)-C(2)	1.385(4)	C(20)-C(25)	1.388(4)
C(1)-C(20)	1.489(4)	C(21)-H(21)	0.9500
C(2)-C(3)	1.394(3)	C(21)-C(22)	1.383(4)
C(2)-C(4)	1.511(3)	C(22)-H(22)	0.9500
C(3)-H(3)	0.9500	C(22)-C(23)	1.384(5)
C(4)-C(5)	1.538(3)	C(23)-C(24)	1.382(5)
C(4)-C(14)	1.523(3)	C(24)-H(24)	0.9500
C(5)-C(6)	1.343(4)	C(24)-C(25)	1.399(4)
C(5)-C(15)	1.490(3)	C(25)-H(25)	0.9500
C(6)-H(6)	0.9500	C(26)-H(26A)	0.9800
C(6)-C(7)	1.458(3)	C(26)-H(26B)	0.9800
C(7)-C(14)	1.432(3)	C(26)-H(26C)	0.9800
C(8)-C(9)	1.404(3)	O(3)-H(3O)	0.85(4)
C(8)-C(13)	1.422(3)	O(3)-C(30)	1.428(3)
C(9)-H(9)	0.9500	O(4)-C(49)	1.376(3)
C(9)-C(10)	1.377(4)	O(4)-C(52)	1.438(4)
C(10)-H(10)	0.9500	N(5)-N(6)	1.355(3)
C(10)-C(11)	1.406(4)	N(5)-C(27)	1.372(3)
C(11)-H(11)	0.9500	N(5)-C(42)	1.496(4)
C(11)-C(12)	1.375(4)	N(6)-C(29)	1.324(4)
C(12)-H(12)	0.9500	N(7)-C(33)	1.307(3)
C(12)-C(13)	1.406(4)	N(7)-C(34)	1.388(3)
C(15)-H(15A)	0.9800	N(8)-C(39)	1.390(3)
C(15)-H(15B)	0.9800	N(8)-C(40)	1.299(3)
C(15)-H(15C)	0.9800	C(27)-C(28)	1.386(4)
C(16)-C(17)	1.521(4)	C(27)-C(46)	1.487(4)

C(28)-C(29)	1.399(4)
C(28)-C(30)	1.512(3)
C(29)-H(29)	0.9500
C(30)-C(31)	1.542(3)
C(30)-C(40)	1.526(3)
C(31)-C(32)	1.342(4)
C(31)-C(41)	1.489(3)
C(32)-H(32)	0.9500
C(32)-C(33)	1.454(3)
C(33)-C(40)	1.433(3)
C(34)-C(35)	1.404(3)
C(34)-C(39)	1.415(4)
C(35)-H(35)	0.9500
C(35)-C(36)	1.377(4)
C(36)-H(36)	0.9500
C(36)-C(37)	1.404(4)
C(37)-H(37)	0.9500
C(37)-C(38)	1.370(4)
C(38)-H(38)	0.9500
C(38)-C(39)	1.411(4)
C(41)-H(41A)	0.9800
C(41)-H(41B)	0.9800
C(41)-H(41C)	0.9800
C(42)-C(43)	1.531(6)
C(42)-C(44)	1.514(6)
C(42)-C(45)	1.513(5)
C(42)-C(43A)	1.531(7)
C(42)-C(44A)	1.515(7)
C(42)-C(45A)	1.513(6)
C(43)-H(43A)	0.9800
C(43)-H(43B)	0.9800
C(43)-H(43C)	0.9800
C(44)-H(44A)	0.9800
C(44)-H(44B)	0.9800
C(44)-H(44C)	0.9800
C(45)-H(45A)	0.9800
C(45)-H(45B)	0.9800
C(45)-H(45C)	0.9800
C(43A)-H(43D)	0.9800
C(43A)-H(43E)	0.9800
C(43A)-H(43F)	0.9800
C(44A)-H(44D)	0.9800
C(44A)-H(44E)	0.9800
C(44A)-H(44F)	0.9800
C(45A)-H(45D)	0.9800
C(45A)-H(45E)	0.9800
C(45A)-H(45F)	0.9800
C(46)-C(47)	1.395(4)
C(46)-C(51)	1.392(4)
C(47)-H(47)	0.9500
C(47)-C(48)	1.382(4)
C(48)-H(48)	0.9500
C(48)-C(49)	1.398(4)
C(49)-C(50)	1.380(4)
C(50)-H(50)	0.9500
C(50)-C(51)	1.387(4)
C(51)-H(51)	0.9500
C(52)-H(52A)	0.9800
C(52)-H(52B)	0.9800
C(52)-H(52C)	0.9800
O(5)-H(5O)	0.90(7)
O(5)-O(6)	1.468(4)
O(6)-H(6O)	0.95(6)
O(7)-H(7O)	0.8400
O(7)-O(8)	1.468(5)
O(8)-H(8O)	0.8400
C(4)-O(1)-H(1O)	108(2)
C(23)-O(2)-C(26)	116.6(3)
N(2)-N(1)-C(1)	110.6(2)
N(2)-N(1)-C(16)	116.9(2)
C(1)-N(1)-C(16)	131.8(2)
C(3)-N(2)-N(1)	106.1(2)
C(7)-N(3)-C(8)	114.4(2)
C(14)-N(4)-C(13)	114.0(2)
N(1)-C(1)-C(2)	106.4(2)
N(1)-C(1)-C(20)	126.3(2)
C(2)-C(1)-C(20)	127.3(2)
C(1)-C(2)-C(3)	105.3(2)
C(1)-C(2)-C(4)	127.5(2)

C(3)-C(2)-C(4)	127.3(2)	C(5)-C(15)-H(15A)	109.5
N(2)-C(3)-C(2)	111.5(2)	C(5)-C(15)-H(15B)	109.5
N(2)-C(3)-H(3)	124.2	C(5)-C(15)-H(15C)	109.5
C(2)-C(3)-H(3)	124.2	H(15A)-C(15)-H(15B)	109.5
O(1)-C(4)-C(2)	106.31(19)	H(15A)-C(15)-H(15C)	109.5
O(1)-C(4)-C(5)	112.24(19)	H(15B)-C(15)-H(15C)	109.5
O(1)-C(4)-C(14)	112.96(19)	N(1)-C(16)-C(17)	111.4(2)
C(2)-C(4)-C(5)	111.8(2)	N(1)-C(16)-C(18)	107.8(2)
C(2)-C(4)-C(14)	112.7(2)	N(1)-C(16)-C(19)	107.8(2)
C(14)-C(4)-C(5)	100.90(19)	C(17)-C(16)-C(19)	108.2(3)
C(6)-C(5)-C(4)	112.0(2)	C(18)-C(16)-C(17)	111.1(3)
C(6)-C(5)-C(15)	127.3(2)	C(18)-C(16)-C(19)	110.5(3)
C(15)-C(5)-C(4)	120.6(2)	C(16)-C(17)-H(17A)	109.5
C(5)-C(6)-H(6)	125.2	C(16)-C(17)-H(17B)	109.5
C(5)-C(6)-C(7)	109.6(2)	C(16)-C(17)-H(17C)	109.5
C(7)-C(6)-H(6)	125.2	H(17A)-C(17)-H(17B)	109.5
N(3)-C(7)-C(6)	128.7(2)	H(17A)-C(17)-H(17C)	109.5
N(3)-C(7)-C(14)	123.0(2)	H(17B)-C(17)-H(17C)	109.5
C(14)-C(7)-C(6)	108.3(2)	C(16)-C(18)-H(18A)	109.5
N(3)-C(8)-C(9)	118.9(2)	C(16)-C(18)-H(18B)	109.5
N(3)-C(8)-C(13)	121.8(2)	C(16)-C(18)-H(18C)	109.5
C(9)-C(8)-C(13)	119.4(2)	H(18A)-C(18)-H(18B)	109.5
C(8)-C(9)-H(9)	119.9	H(18A)-C(18)-H(18C)	109.5
C(10)-C(9)-C(8)	120.3(2)	H(18B)-C(18)-H(18C)	109.5
C(10)-C(9)-H(9)	119.9	C(16)-C(19)-H(19A)	109.5
C(9)-C(10)-H(10)	119.9	C(16)-C(19)-H(19B)	109.5
C(9)-C(10)-C(11)	120.2(2)	C(16)-C(19)-H(19C)	109.5
C(11)-C(10)-H(10)	119.9	H(19A)-C(19)-H(19B)	109.5
C(10)-C(11)-H(11)	119.6	H(19A)-C(19)-H(19C)	109.5
C(12)-C(11)-C(10)	120.8(2)	H(19B)-C(19)-H(19C)	109.5
C(12)-C(11)-H(11)	119.6	C(21)-C(20)-C(1)	119.9(3)
C(11)-C(12)-H(12)	120.0	C(25)-C(20)-C(1)	121.5(3)
C(11)-C(12)-C(13)	119.9(2)	C(25)-C(20)-C(21)	118.5(3)
C(13)-C(12)-H(12)	120.0	C(20)-C(21)-H(21)	119.8
N(4)-C(13)-C(8)	121.9(2)	C(22)-C(21)-C(20)	120.5(3)
N(4)-C(13)-C(12)	118.6(2)	C(22)-C(21)-H(21)	119.8
C(12)-C(13)-C(8)	119.5(2)	C(21)-C(22)-H(22)	119.8
N(4)-C(14)-C(4)	126.1(2)	C(21)-C(22)-C(23)	120.5(3)
N(4)-C(14)-C(7)	124.7(2)	C(23)-C(22)-H(22)	119.8
C(7)-C(14)-C(4)	109.2(2)	O(2)-C(23)-C(22)	114.9(3)

O(2)-C(23)-C(24)	125.0(3)	C(31)-C(32)-H(32)	125.1
C(24)-C(23)-C(22)	120.1(3)	C(31)-C(32)-C(33)	109.8(2)
C(23)-C(24)-H(24)	120.4	C(33)-C(32)-H(32)	125.1
C(23)-C(24)-C(25)	119.3(3)	N(7)-C(33)-C(32)	128.4(2)
C(25)-C(24)-H(24)	120.4	N(7)-C(33)-C(40)	123.2(2)
C(20)-C(25)-C(24)	121.2(3)	C(40)-C(33)-C(32)	108.4(2)
C(20)-C(25)-H(25)	119.4	N(7)-C(34)-C(35)	119.0(2)
C(24)-C(25)-H(25)	119.4	N(7)-C(34)-C(39)	121.3(2)
O(2)-C(26)-H(26A)	109.5	C(35)-C(34)-C(39)	119.7(2)
O(2)-C(26)-H(26B)	109.5	C(34)-C(35)-H(35)	119.7
O(2)-C(26)-H(26C)	109.5	C(36)-C(35)-C(34)	120.6(2)
H(26A)-C(26)-H(26B)	109.5	C(36)-C(35)-H(35)	119.7
H(26A)-C(26)-H(26C)	109.5	C(35)-C(36)-H(36)	120.3
H(26B)-C(26)-H(26C)	109.5	C(35)-C(36)-C(37)	119.5(2)
C(30)-O(3)-H(3O)	112(2)	C(37)-C(36)-H(36)	120.3
C(49)-O(4)-C(52)	117.3(3)	C(36)-C(37)-H(37)	119.4
N(6)-N(5)-C(27)	111.5(2)	C(38)-C(37)-C(36)	121.2(3)
N(6)-N(5)-C(42)	118.7(2)	C(38)-C(37)-H(37)	119.4
C(27)-N(5)-C(42)	129.8(2)	C(37)-C(38)-H(38)	119.9
C(29)-N(6)-N(5)	105.5(2)	C(37)-C(38)-C(39)	120.1(3)
C(33)-N(7)-C(34)	114.8(2)	C(39)-C(38)-H(38)	119.9
C(40)-N(8)-C(39)	114.1(2)	N(8)-C(39)-C(34)	122.2(2)
N(5)-C(27)-C(28)	106.1(2)	N(8)-C(39)-C(38)	118.9(2)
N(5)-C(27)-C(46)	127.2(2)	C(38)-C(39)-C(34)	118.9(2)
C(28)-C(27)-C(46)	126.6(2)	N(8)-C(40)-C(30)	126.7(2)
C(27)-C(28)-C(29)	105.2(2)	N(8)-C(40)-C(33)	124.2(2)
C(27)-C(28)-C(30)	128.7(2)	C(33)-C(40)-C(30)	109.1(2)
C(29)-C(28)-C(30)	125.9(2)	C(31)-C(41)-H(41A)	109.5
N(6)-C(29)-C(28)	111.7(2)	C(31)-C(41)-H(41B)	109.5
N(6)-C(29)-H(29)	124.2	C(31)-C(41)-H(41C)	109.5
C(28)-C(29)-H(29)	124.2	H(41A)-C(41)-H(41B)	109.5
O(3)-C(30)-C(28)	106.32(19)	H(41A)-C(41)-H(41C)	109.5
O(3)-C(30)-C(31)	112.54(19)	H(41B)-C(41)-H(41C)	109.5
O(3)-C(30)-C(40)	112.5(2)	N(5)-C(42)-C(43)	107.9(3)
C(28)-C(30)-C(31)	110.3(2)	N(5)-C(42)-C(44)	110.0(3)
C(28)-C(30)-C(40)	114.6(2)	N(5)-C(42)-C(45)	108.8(3)
C(40)-C(30)-C(31)	100.75(19)	N(5)-C(42)-C(43A)	116.1(15)
C(32)-C(31)-C(30)	111.9(2)	N(5)-C(42)-C(44A)	102.3(17)
C(32)-C(31)-C(41)	127.1(2)	N(5)-C(42)-C(45A)	104.3(17)
C(41)-C(31)-C(30)	120.9(2)	C(44)-C(42)-C(43)	110.8(4)

C(45)-C(42)-C(43)	108.3(4)
C(45)-C(42)-C(44)	111.0(4)
C(44A)-C(42)-C(43A)	111.3(15)
C(45A)-C(42)-C(43A)	111.4(15)
C(45A)-C(42)-C(44A)	110.8(15)
C(42)-C(43)-H(43A)	109.5
C(42)-C(43)-H(43B)	109.5
C(42)-C(43)-H(43C)	109.5
H(43A)-C(43)-H(43B)	109.5
H(43A)-C(43)-H(43C)	109.5
H(43B)-C(43)-H(43C)	109.5
C(42)-C(44)-H(44A)	109.5
C(42)-C(44)-H(44B)	109.5
C(42)-C(44)-H(44C)	109.5
H(44A)-C(44)-H(44B)	109.5
H(44A)-C(44)-H(44C)	109.5
H(44B)-C(44)-H(44C)	109.5
C(42)-C(45)-H(45A)	109.5
C(42)-C(45)-H(45B)	109.5
C(42)-C(45)-H(45C)	109.5
H(45A)-C(45)-H(45B)	109.5
H(45A)-C(45)-H(45C)	109.5
H(45B)-C(45)-H(45C)	109.5
C(42)-C(43A)-H(43D)	109.5
C(42)-C(43A)-H(43E)	109.5
C(42)-C(43A)-H(43F)	109.5
H(43D)-C(43A)-H(43E)	109.5
H(43D)-C(43A)-H(43F)	109.5
H(43E)-C(43A)-H(43F)	109.5
C(42)-C(44A)-H(44D)	109.5
C(42)-C(44A)-H(44E)	109.5
C(42)-C(44A)-H(44F)	109.5
H(44D)-C(44A)-H(44E)	109.5
H(44D)-C(44A)-H(44F)	109.5
H(44E)-C(44A)-H(44F)	109.5
C(42)-C(45A)-H(45D)	109.5
C(42)-C(45A)-H(45E)	109.5
C(42)-C(45A)-H(45F)	109.5
H(45D)-C(45A)-H(45E)	109.5
H(45D)-C(45A)-H(45F)	109.5
H(45E)-C(45A)-H(45F)	109.5
C(47)-C(46)-C(27)	122.8(3)
C(51)-C(46)-C(27)	118.9(2)
C(51)-C(46)-C(47)	117.8(3)
C(46)-C(47)-H(47)	119.7
C(48)-C(47)-C(46)	120.6(3)
C(48)-C(47)-H(47)	119.7
C(47)-C(48)-H(48)	119.8
C(47)-C(48)-C(49)	120.4(3)
C(49)-C(48)-H(48)	119.8
O(4)-C(49)-C(48)	116.1(3)
O(4)-C(49)-C(50)	124.0(3)
C(50)-C(49)-C(48)	119.9(3)
C(49)-C(50)-H(50)	120.6
C(49)-C(50)-C(51)	118.8(3)
C(51)-C(50)-H(50)	120.6
C(46)-C(51)-H(51)	118.8
C(50)-C(51)-C(46)	122.5(3)
C(50)-C(51)-H(51)	118.8
O(4)-C(52)-H(52A)	109.5
O(4)-C(52)-H(52B)	109.5
O(4)-C(52)-H(52C)	109.5
H(52A)-C(52)-H(52B)	109.5
H(52A)-C(52)-H(52C)	109.5
H(52B)-C(52)-H(52C)	109.5
O(6)-O(5)-H(5O)	98(4)
O(5)-O(6)-H(6O)	102(3)
O(8)-O(7)-H(7O)	109.5
O(7)-O(8)-H(8O)	109.5

Table S5. Anisotropic displacement parameters ($Å^2$x 10^3) for **15a**. The anisotropic displacement factor exponent takes the form: $-2\pi^2$[h^2 $a^{*2}U^{11}$ + ... + 2 h k a* b* U^{12}]

	U^{11}	U^{22}	U^{33}	U^{23}	U^{13}	U^{12}
O(1)	24(1)	21(1)	21(1)	-9(1)	-6(1)	4(1)
O(2)	67(2)	59(2)	61(2)	-32(1)	-39(1)	33(1)
N(1)	32(1)	24(1)	27(1)	-13(1)	-4(1)	4(1)
N(2)	32(1)	24(1)	29(1)	-12(1)	-6(1)	7(1)
N(3)	22(1)	23(1)	22(1)	-9(1)	-3(1)	3(1)
N(4)	20(1)	22(1)	23(1)	-9(1)	-3(1)	1(1)
C(1)	25(1)	23(1)	25(1)	-11(1)	0(1)	2(1)
C(2)	22(1)	21(1)	21(1)	-7(1)	-1(1)	0(1)
C(3)	27(1)	23(1)	24(1)	-10(1)	-4(1)	3(1)
C(4)	21(1)	22(1)	18(1)	-7(1)	-4(1)	2(1)
C(5)	26(1)	24(1)	21(1)	-11(1)	-1(1)	-3(1)
C(6)	21(1)	24(1)	22(1)	-10(1)	-1(1)	1(1)
C(7)	19(1)	22(1)	22(1)	-11(1)	-4(1)	1(1)
C(8)	23(1)	22(1)	22(1)	-10(1)	-2(1)	0(1)
C(9)	27(1)	24(1)	26(1)	-9(1)	-5(1)	4(1)
C(10)	36(1)	22(1)	20(1)	-5(1)	-2(1)	0(1)
C(11)	30(1)	26(1)	27(1)	-9(1)	2(1)	-4(1)
C(12)	24(1)	29(1)	25(1)	-11(1)	-3(1)	0(1)
C(13)	24(1)	22(1)	21(1)	-10(1)	-5(1)	2(1)
C(14)	21(1)	21(1)	22(1)	-11(1)	-5(1)	2(1)
C(15)	26(1)	26(1)	27(1)	-9(1)	1(1)	-2(1)
C(16)	42(2)	30(1)	33(1)	-20(1)	-4(1)	5(1)
C(17)	71(2)	46(2)	35(2)	-27(2)	-12(2)	14(2)
C(18)	76(3)	52(2)	48(2)	-34(2)	9(2)	-23(2)
C(19)	58(2)	50(2)	51(2)	-34(2)	-6(2)	17(2)
C(20)	30(1)	31(1)	26(1)	-17(1)	-7(1)	7(1)
C(21)	31(1)	46(2)	37(2)	-25(1)	-6(1)	5(1)
C(22)	31(2)	61(2)	51(2)	-32(2)	-15(1)	14(2)
C(23)	51(2)	46(2)	45(2)	-31(2)	-27(2)	26(2)
C(24)	65(2)	31(1)	27(1)	-15(1)	-13(1)	12(1)
C(25)	39(2)	32(1)	29(1)	-15(1)	-8(1)	7(1)
C(26)	85(3)	53(2)	49(2)	-27(2)	-30(2)	35(2)
O(3)	28(1)	21(1)	22(1)	-10(1)	-7(1)	5(1)
O(4)	57(1)	32(1)	31(1)	-10(1)	-13(1)	8(1)
N(5)	45(1)	26(1)	31(1)	-15(1)	-9(1)	9(1)

N(6)	58(2)	27(1)	32(1)	-13(1)	-14(1)	13(1)
N(7)	23(1)	21(1)	23(1)	-9(1)	-3(1)	2(1)
N(8)	21(1)	23(1)	25(1)	-9(1)	-3(1)	1(1)
C(27)	29(1)	23(1)	26(1)	-9(1)	-2(1)	3(1)
C(28)	26(1)	21(1)	22(1)	-8(1)	-3(1)	3(1)
C(29)	47(2)	25(1)	26(1)	-11(1)	-10(1)	7(1)
C(30)	24(1)	21(1)	19(1)	-7(1)	-3(1)	1(1)
C(31)	27(1)	23(1)	20(1)	-9(1)	-1(1)	-2(1)
C(32)	22(1)	24(1)	23(1)	-9(1)	-2(1)	0(1)
C(33)	20(1)	22(1)	21(1)	-10(1)	-3(1)	2(1)
C(34)	24(1)	23(1)	21(1)	-10(1)	-1(1)	0(1)
C(35)	30(1)	22(1)	26(1)	-9(1)	-4(1)	4(1)
C(36)	39(2)	21(1)	23(1)	-6(1)	-1(1)	-3(1)
C(37)	29(1)	30(1)	27(1)	-10(1)	3(1)	-6(1)
C(38)	24(1)	29(1)	30(1)	-12(1)	-3(1)	0(1)
C(39)	23(1)	22(1)	22(1)	-8(1)	-2(1)	0(1)
C(40)	21(1)	20(1)	21(1)	-8(1)	-2(1)	2(1)
C(41)	29(1)	26(1)	27(1)	-8(1)	2(1)	-4(1)
C(42)	67(2)	32(2)	39(2)	-22(1)	-9(2)	7(2)
C(43)	77(3)	41(2)	63(3)	-32(2)	-22(2)	1(2)
C(44)	102(4)	69(3)	62(3)	-50(3)	17(3)	-7(3)
C(45)	122(5)	43(2)	71(3)	-42(2)	-41(3)	35(3)
C(43A)	77(3)	41(2)	63(3)	-32(2)	-22(2)	1(2)
C(44A)	102(4)	69(3)	62(3)	-50(3)	17(3)	-7(3)
C(45A)	122(5)	43(2)	71(3)	-42(2)	-41(3)	35(3)
C(46)	34(1)	26(1)	24(1)	-12(1)	-5(1)	3(1)
C(47)	34(1)	29(1)	30(1)	-16(1)	-4(1)	1(1)
C(48)	35(2)	33(1)	37(2)	-20(1)	-9(1)	6(1)
C(49)	47(2)	25(1)	25(1)	-14(1)	-11(1)	5(1)
C(50)	42(2)	32(1)	25(1)	-10(1)	-1(1)	-3(1)
C(51)	33(1)	36(2)	26(1)	-11(1)	-4(1)	1(1)
C(52)	74(3)	34(2)	31(2)	-6(1)	-10(2)	0(2)
O(5)	88(3)	47(2)	55(2)	-25(2)	-30(2)	37(2)
O(6)	47(2)	62(2)	37(2)	-12(1)	-9(1)	12(1)
O(7)	47(11)	57(12)	60(12)	-21(9)	-18(8)	6(8)
O(8)	47(11)	57(12)	60(12)	-21(9)	-18(8)	6(8)

Table S6. Hydrogen coordinates (x 10^4) and isotropic displacement parameters ($Å^2$x 10^3) for **15a**.

	x	y	z	U(eq)
H(1O)	7760(50)	1190(20)	4169(18)	33
H(3)	5974	2770	4412	29
H(6)	13232	748	5106	27
H(9)	10370	-1799	7246	31
H(10)	7497	-2474	7955	33
H(11)	4264	-1926	7699	34
H(12)	3909	-680	6781	31
H(15A)	13195	2126	4016	41
H(15B)	11894	2610	4379	41
H(15C)	11000	2441	3743	41
H(17A)	8090	2920	7380	70
H(17B)	9610	2491	7018	70
H(17C)	7319	2181	7280	70
H(18A)	9104	4157	6186	82
H(18B)	8596	4175	5413	82
H(18C)	10385	3638	5856	82
H(19A)	5447	3731	6744	73
H(19B)	4428	3059	6596	73
H(19C)	5027	3879	5922	73
H(21)	12348	2227	5904	42
H(22)	14524	1404	6750	53
H(24)	9816	-43	7885	47
H(25)	7624	781	7027	39
H(26A)	14246	-786	8764	89
H(26B)	12707	-898	8238	89
H(26C)	11980	-464	8743	89
H(3O)	2110(50)	3840(20)	10916(19)	35
H(29)	2996	2057	11021	39
H(32)	-3223	4344	9877	28
H(35)	8	6660	7591	31
H(36)	2953	7209	6846	34
H(37)	6081	6587	7164	35
H(38)	6282	5435	8211	33
H(41A)	-3500	3033	11024	43
H(41B)	-1413	2747	11407	43

H(41C)	-2084	2445	10824	43
H(43A)	-162	1125	8715	83
H(43B)	-584	1968	8690	83
H(43C)	-927	1208	9453	83
H(44A)	3604	1549	8186	107
H(44B)	4789	2012	8545	107
H(44C)	2815	2380	8111	107
H(45A)	2865	384	9309	106
H(45B)	1936	436	10045	106
H(45C)	4229	714	9739	106
H(43D)	884	1480	8236	83
H(43E)	-613	1849	8664	83
H(43F)	1288	2345	8145	83
H(44D)	4505	1205	8593	107
H(44E)	4833	2032	8603	107
H(44F)	5138	1248	9327	107
H(45D)	1344	356	9447	106
H(45E)	2369	485	10083	106
H(45F)	61	731	9921	106
H(47)	-2218	3172	8981	35
H(48)	-3163	4206	7915	39
H(50)	2745	4638	7134	41
H(51)	3670	3610	8210	39
H(52A)	-212	6018	5855	73
H(52B)	1286	5294	6069	73
H(52C)	1380	5866	6463	73
H(5O)	4920(100)	4050(40)	4730(30)	92
H(6O)	3510(90)	4590(30)	3500(30)	78
H(7O)	5905	9754	7947	83
H(8O)	6778	9561	9157	83

Table S7. Torsion angles [°] for **15a**.

O(1)-C(4)-C(5)-C(6)	-120.7(2)
O(1)-C(4)-C(5)-C(15)	61.6(3)
O(1)-C(4)-C(14)-N(4)	-58.6(3)
O(1)-C(4)-C(14)-C(7)	121.0(2)
O(2)-C(23)-C(24)-C(25)	179.0(3)
N(1)-N(2)-C(3)-C(2)	-0.1(3)
N(1)-C(1)-C(2)-C(3)	1.1(3)
N(1)-C(1)-C(2)-C(4)	179.1(2)
N(1)-C(1)-C(20)-C(21)	-88.8(3)
N(1)-C(1)-C(20)-C(25)	94.6(3)
N(2)-N(1)-C(1)-C(2)	-1.3(3)
N(2)-N(1)-C(1)-C(20)	179.2(2)
N(2)-N(1)-C(16)-C(17)	162.1(3)
N(2)-N(1)-C(16)-C(18)	-75.8(3)
N(2)-N(1)-C(16)-C(19)	43.5(3)
N(3)-C(7)-C(14)-N(4)	-0.7(4)
N(3)-C(7)-C(14)-C(4)	179.8(2)
N(3)-C(8)-C(9)-C(10)	-178.6(2)
N(3)-C(8)-C(13)-N(4)	-2.5(4)
N(3)-C(8)-C(13)-C(12)	178.1(2)
C(1)-N(1)-N(2)-C(3)	0.9(3)
C(1)-N(1)-C(16)-C(17)	-28.4(4)
C(1)-N(1)-C(16)-C(18)	93.7(4)
C(1)-N(1)-C(16)-C(19)	-147.0(3)
C(1)-C(2)-C(3)-N(2)	-0.6(3)
C(1)-C(2)-C(4)-O(1)	173.8(2)
C(1)-C(2)-C(4)-C(5)	-63.4(3)
C(1)-C(2)-C(4)-C(14)	49.5(3)
C(1)-C(20)-C(21)-C(22)	-177.7(3)
C(1)-C(20)-C(25)-C(24)	178.0(2)
C(2)-C(1)-C(20)-C(21)	91.8(3)
C(2)-C(1)-C(20)-C(25)	-84.8(4)
C(2)-C(4)-C(5)-C(6)	119.9(2)
C(2)-C(4)-C(5)-C(15)	-57.8(3)
C(2)-C(4)-C(14)-N(4)	62.0(3)
C(2)-C(4)-C(14)-C(7)	-118.5(2)
C(3)-C(2)-C(4)-O(1)	-8.6(3)
C(3)-C(2)-C(4)-C(5)	114.2(3)
C(3)-C(2)-C(4)-C(14)	-132.9(3)
C(4)-C(2)-C(3)-N(2)	-178.6(2)
C(4)-C(5)-C(6)-C(7)	-0.6(3)
C(5)-C(4)-C(14)-N(4)	-178.6(2)
C(5)-C(4)-C(14)-C(7)	1.0(2)
C(5)-C(6)-C(7)-N(3)	-180.0(2)
C(5)-C(6)-C(7)-C(14)	1.3(3)
C(6)-C(7)-C(14)-N(4)	178.2(2)
C(6)-C(7)-C(14)-C(4)	-1.4(3)
C(7)-N(3)-C(8)-C(9)	-176.4(2)
C(7)-N(3)-C(8)-C(13)	4.5(3)
C(8)-N(3)-C(7)-C(6)	178.4(2)
C(8)-N(3)-C(7)-C(14)	-3.0(3)
C(8)-C(9)-C(10)-C(11)	0.8(4)
C(9)-C(8)-C(13)-N(4)	178.4(2)
C(9)-C(8)-C(13)-C(12)	-1.0(4)
C(9)-C(10)-C(11)-C(12)	-1.8(4)
C(10)-C(11)-C(12)-C(13)	1.4(4)
C(11)-C(12)-C(13)-N(4)	-179.3(2)
C(11)-C(12)-C(13)-C(8)	0.0(4)
C(13)-N(4)-C(14)-C(4)	-177.8(2)
C(13)-N(4)-C(14)-C(7)	2.8(3)
C(13)-C(8)-C(9)-C(10)	0.5(4)
C(14)-N(4)-C(13)-C(8)	-1.2(3)
C(14)-N(4)-C(13)-C(12)	178.2(2)
C(14)-C(4)-C(5)-C(6)	-0.2(3)
C(14)-C(4)-C(5)-C(15)	-177.9(2)
C(15)-C(5)-C(6)-C(7)	176.9(2)
C(16)-N(1)-N(2)-C(3)	172.5(2)
C(16)-N(1)-C(1)-C(2)	-171.2(3)
C(16)-N(1)-C(1)-C(20)	9.3(5)
C(20)-C(1)-C(2)-C(3)	-179.4(3)
C(20)-C(1)-C(2)-C(4)	-1.4(4)
C(20)-C(21)-C(22)-C(23)	0.1(5)
C(21)-C(20)-C(25)-C(24)	1.4(4)
C(21)-C(22)-C(23)-O(2)	-178.7(3)
C(21)-C(22)-C(23)-C(24)	0.6(5)
C(22)-C(23)-C(24)-C(25)	-0.3(5)

C(23)-C(24)-C(25)-C(20)	-0.7(4)
C(25)-C(20)-C(21)-C(22)	-1.1(4)
C(26)-O(2)-C(23)-C(22)	-175.3(3)
C(26)-O(2)-C(23)-C(24)	5.4(5)
O(3)-C(30)-C(31)-C(32)	119.7(2)
O(3)-C(30)-C(31)-C(41)	-62.7(3)
O(3)-C(30)-C(40)-N(8)	58.0(3)
O(3)-C(30)-C(40)-C(33)	-120.0(2)
O(4)-C(49)-C(50)-C(51)	178.1(3)
N(5)-N(6)-C(29)-C(28)	0.3(4)
N(5)-C(27)-C(28)-C(29)	-0.6(3)
N(5)-C(27)-C(28)-C(30)	-174.9(3)
N(5)-C(27)-C(46)-C(47)	103.8(3)
N(5)-C(27)-C(46)-C(51)	-84.6(4)
N(6)-N(5)-C(27)-C(28)	0.8(3)
N(6)-N(5)-C(27)-C(46)	177.0(3)
N(6)-N(5)-C(42)-C(43)	117.9(3)
N(6)-N(5)-C(42)-C(44)	-121.2(4)
N(6)-N(5)-C(42)-C(45)	0.6(5)
N(6)-N(5)-C(42)-C(43A)	161.9(17)
N(6)-N(5)-C(42)-C(44A)	-76.7(18)
N(6)-N(5)-C(42)-C(45A)	38.9(18)
N(7)-C(33)-C(40)-N(8)	1.8(4)
N(7)-C(33)-C(40)-C(30)	179.9(2)
N(7)-C(34)-C(35)-C(36)	-179.9(2)
N(7)-C(34)-C(39)-N(8)	2.1(4)
N(7)-C(34)-C(39)-C(38)	-179.3(2)
C(27)-N(5)-N(6)-C(29)	-0.7(4)
C(27)-N(5)-C(42)-C(43)	-59.5(4)
C(27)-N(5)-C(42)-C(44)	61.4(5)
C(27)-N(5)-C(42)-C(45)	-176.8(4)
C(27)-N(5)-C(42)-C(43A)	-15.5(18)
C(27)-N(5)-C(42)-C(44A)	105.9(18)
C(27)-N(5)-C(42)-C(45A)	-138.5(18)
C(27)-C(28)-C(29)-N(6)	0.2(3)
C(27)-C(28)-C(30)-O(3)	-164.4(3)
C(27)-C(28)-C(30)-C(31)	73.3(3)
C(27)-C(28)-C(30)-C(40)	-39.5(4)
C(27)-C(46)-C(47)-C(48)	170.8(2)
C(27)-C(46)-C(51)-C(50)	-171.5(3)

C(28)-C(27)-C(46)-C(47)	-80.7(4)
C(28)-C(27)-C(46)-C(51)	90.8(4)
C(28)-C(30)-C(31)-C(32)	-121.7(2)
C(28)-C(30)-C(31)-C(41)	55.9(3)
C(28)-C(30)-C(40)-N(8)	-63.6(3)
C(28)-C(30)-C(40)-C(33)	118.4(2)
C(29)-C(28)-C(30)-O(3)	22.4(3)
C(29)-C(28)-C(30)-C(31)	-99.9(3)
C(29)-C(28)-C(30)-C(40)	147.3(3)
C(30)-C(28)-C(29)-N(6)	174.6(3)
C(30)-C(31)-C(32)-C(33)	0.5(3)
C(31)-C(30)-C(40)-N(8)	178.0(2)
C(31)-C(30)-C(40)-C(33)	0.0(2)
C(31)-C(32)-C(33)-N(7)	179.9(2)
C(31)-C(32)-C(33)-C(40)	-0.4(3)
C(32)-C(33)-C(40)-N(8)	-177.8(2)
C(32)-C(33)-C(40)-C(30)	0.2(3)
C(33)-N(7)-C(34)-C(35)	175.1(2)
C(33)-N(7)-C(34)-C(39)	-4.3(3)
C(34)-N(7)-C(33)-C(32)	-177.9(2)
C(34)-N(7)-C(33)-C(40)	2.6(3)
C(34)-C(35)-C(36)-C(37)	-0.3(4)
C(35)-C(34)-C(39)-N(8)	-177.4(2)
C(35)-C(34)-C(39)-C(38)	1.2(4)
C(35)-C(36)-C(37)-C(38)	0.1(4)
C(36)-C(37)-C(38)-C(39)	0.8(4)
C(37)-C(38)-C(39)-N(8)	177.2(2)
C(37)-C(38)-C(39)-C(34)	-1.4(4)
C(39)-N(8)-C(40)-C(30)	178.2(2)
C(39)-N(8)-C(40)-C(33)	-4.1(3)
C(39)-C(34)-C(35)-C(36)	-0.4(4)
C(40)-N(8)-C(39)-C(34)	2.1(3)
C(40)-N(8)-C(39)-C(38)	-176.4(2)
C(40)-C(30)-C(31)-C(32)	-0.3(3)
C(40)-C(30)-C(31)-C(41)	177.3(2)
C(41)-C(31)-C(32)-C(33)	-177.0(2)
C(42)-N(5)-N(6)-C(29)	-178.6(3)
C(42)-N(5)-C(27)-C(28)	178.4(3)
C(42)-N(5)-C(27)-C(46)	-5.4(5)
C(46)-C(27)-C(28)-C(29)	-176.8(3)

C(46)-C(27)-C(28)-C(30)	8.9(5)		C(48)-C(49)-C(50)-C(51)	-1.5(4)
C(46)-C(47)-C(48)-C(49)	0.1(4)		C(49)-C(50)-C(51)-C(46)	0.7(4)
C(47)-C(46)-C(51)-C(50)	0.4(4)		C(51)-C(46)-C(47)-C(48)	-0.8(4)
C(47)-C(48)-C(49)-O(4)	-178.5(2)		C(52)-O(4)-C(49)-C(48)	178.7(3)
C(47)-C(48)-C(49)-C(50)	1.1(4)		C(52)-O(4)-C(49)-C(50)	-0.9(4)

Table S8. Hydrogen bonds for **15a** [Å and °].

D-H...A	d(D-H)	d(H...A)	d(D...A)	<(DHA)
O(1)-H(1O)...N(3)#1	0.89(3)	2.05(4)	2.917(3)	163(3)
O(3)-H(3O)...N(7)#2	0.85(4)	2.07(4)	2.894(3)	162(3)
O(5)-H(5O)...N(2)	0.90(7)	1.91(7)	2.805(4)	170(6)
O(6)-H(6O)...O(4)#3	0.95(6)	1.81(6)	2.754(4)	172(5)
O(7)-H(7O)...O(2)#4	0.84	1.57	2.33(2)	148.8
O(8)-H(8O)...N(6)#5	0.84	2.05	2.70(3)	134.5

Symmetry transformations used to generate equivalent atoms: #1 -x+2,-y,-z+1 #2 -x,-y+1,-z+2
#3 -x,-y+1,-z+1 #4 x-1,y+1,z #5 -x+1,-y+1,-z+2

X-ray crystallographic data for compound 15m:

X-ray diffraction data were collected at 100K on a Rigaku Synergy S diffractometer equipped with a HyPix600HE area-detector (kappa geometry, shutterless ω-scan technique), using monochromatized Cu K_a-radiation. The intensity data were integrated and corrected for absorption and decay by the CrysAlisPro program.[3] The structure was solved by direct methods using SHELXT[4] and refined on F^4 using SHELXL-2018[5] in the OLEX2 program.[6] Positions of all atoms were found from the electron density-difference map. Atoms were refined with individual anisotropic (non-hydrogen atoms) or isotropic (hydrogen atoms) displacement parameters.

Table S9. Crystal data and structure refinement for **15m**.

Identification code	**15m**
Empirical formula	C29 H24 N4 O2
Formula weight	460.52
Temperature	100.0(1) K
Wavelength	1.54184 Å
Crystal system	Monoclinic
Space group	$P2_1/n$
Unit cell dimensions	a = 15.06336(14) Å □= 90°.<br>b = 7.17340(5) Å □=109.6165(10)°.<br>c = 22.2144(2) Å □ = 90°.
Volume	2261.07(4) Å^3
Z	4
Density (calculated)	1.353 g/cm^3
Absorption coefficient	0.694 mm^{-1}
F(000)	968
Crystal size	0.05 x 0.04 x 0.01 mm^3
Theta range for data collection	3.122 to 79.505°.
Index ranges	-19<=h<=19, -8<=k<=9, -28<=l<=28
Reflections collected	32034
Independent reflections	4916 [R(int) = 0.0239]
Observed reflections	4630
Completeness to theta = 67.684°	100.0 %
Absorption correction	Semi-empirical from equivalents
Max. and min. transmission	1.00000 and 0.93109
Refinement method	Full-matrix least-squares on F^2
Data / restraints / parameters	4916 / 0 / 412
Goodness-of-fit on F^2	1.055
Final R indices [I>2sigma(I)]	R1 = 0.0382, wR2 = 0.0990
R indices (all data)	R1 = 0.0400, wR2 = 0.1004
Largest diff. peak and hole	0.303 and -0.238 e.Å^{-3}
CCDC	2159682

Table S10. Atomic coordinates (x 10^4) and equivalent isotropic displacement parameters ($Å^2$x 10^3) for **15m**. U(eq) is defined as one third of the trace of the orthogonalized U^{ij} tensor.

	x	y	z	U(eq)
O(1)	9252(1)	10460(1)	3488(1)	29(1)
O(2)	5538(1)	3309(1)	4358(1)	24(1)
N(1)	4706(1)	8736(1)	4195(1)	19(1)
N(2)	5395(1)	9588(1)	4030(1)	20(1)
N(3)	8743(1)	3984(1)	4910(1)	22(1)
N(4)	7254(1)	5050(1)	5390(1)	20(1)
C(1)	5996(1)	8224(2)	4016(1)	19(1)
C(2)	5687(1)	6495(2)	4171(1)	19(1)
C(3)	4853(1)	6882(2)	4279(1)	19(1)
C(4)	3942(1)	9842(2)	4288(1)	21(1)
C(5)	3115(1)	10145(2)	3682(1)	19(1)
C(6)	3225(1)	11078(2)	3161(1)	22(1)
C(7)	2460(1)	11360(2)	2609(1)	24(1)
C(8)	1572(1)	10717(2)	2570(1)	25(1)
C(9)	1451(1)	9807(2)	3088(1)	24(1)
C(10)	2223(1)	9529(2)	3643(1)	21(1)
C(11)	6869(1)	8692(2)	3879(1)	20(1)
C(12)	6863(1)	8946(2)	3255(1)	25(1)
C(13)	7671(1)	9498(2)	3135(1)	27(1)
C(14)	8504(1)	9804(2)	3644(1)	23(1)
C(15)	8530(1)	9486(2)	4266(1)	24(1)
C(16)	7712(1)	8942(2)	4378(1)	23(1)
C(17)	10077(1)	10946(2)	4014(1)	28(1)
C(18)	6171(1)	4626(2)	4250(1)	19(1)
C(19)	6493(1)	4090(2)	3685(1)	21(1)
C(20)	7429(1)	3833(2)	3868(1)	22(1)
C(21)	7856(1)	4137(2)	4553(1)	20(1)
C(22)	7117(1)	4642(2)	4797(1)	19(1)
C(23)	5799(1)	3896(2)	3029(1)	26(1)
C(24)	8917(1)	4368(2)	5550(1)	22(1)
C(25)	9848(1)	4197(2)	5977(1)	25(1)
C(26)	10046(1)	4545(2)	6618(1)	28(1)
C(27)	9330(1)	5116(2)	6851(1)	27(1)
C(28)	8415(1)	5315(2)	6443(1)	25(1)
C(29)	8190(1)	4922(2)	5787(1)	21(1)

Table S11. Bond lengths [Å] and angles [°] for **15m**.

O(1)-C(14)	1.3682(14)
O(1)-C(17)	1.4329(16)
O(2)-H(2)	0.93(2)
O(2)-C(18)	1.4195(13)
N(1)-N(2)	1.3574(13)
N(1)-C(3)	1.3511(14)
N(1)-C(4)	1.4682(14)
N(2)-C(1)	1.3404(14)
N(3)-C(21)	1.3106(15)
N(3)-C(24)	1.3845(15)
N(4)-C(22)	1.2972(15)
N(4)-C(29)	1.3924(15)
C(1)-C(2)	1.4075(15)
C(1)-C(11)	1.4833(15)
C(2)-C(3)	1.3833(15)
C(2)-C(18)	1.5088(15)
C(3)-H(3)	0.933(15)
C(4)-H(4A)	0.978(16)
C(4)-H(4B)	1.006(15)
C(4)-C(5)	1.5114(15)
C(5)-C(6)	1.3941(16)
C(5)-C(10)	1.3898(16)
C(6)-H(6)	0.972(16)
C(6)-C(7)	1.3876(17)
C(7)-H(7)	0.982(15)
C(7)-C(8)	1.3895(17)
C(8)-H(8)	0.987(15)
C(8)-C(9)	1.3864(18)
C(9)-H(9)	1.017(16)
C(9)-C(10)	1.3960(17)
C(10)-H(10)	0.978(16)
C(11)-C(12)	1.3964(17)
C(11)-C(16)	1.3890(16)
C(12)-H(12)	1.009(16)
C(12)-C(13)	1.3877(17)
C(13)-H(13)	0.983(18)
C(13)-C(14)	1.3957(17)
C(14)-C(15)	1.3891(17)

C(15)-H(15)	0.999(17)
C(15)-C(16)	1.3922(16)
C(16)-H(16)	0.970(17)
C(17)-H(17A)	0.990(17)
C(17)-H(17B)	0.990(17)
C(17)-H(17C)	1.020(16)
C(18)-C(19)	1.5380(16)
C(18)-C(22)	1.5322(15)
C(19)-C(20)	1.3416(16)
C(19)-C(23)	1.4877(16)
C(20)-H(20)	1.013(15)
C(20)-C(21)	1.4547(16)
C(21)-C(22)	1.4382(15)
C(23)-H(23A)	1.018(18)
C(23)-H(23B)	0.993(18)
C(23)-H(23C)	0.992(18)
C(24)-C(25)	1.4108(16)
C(24)-C(29)	1.4227(16)
C(25)-H(25)	0.953(16)
C(25)-C(26)	1.3762(18)
C(26)-H(26)	0.999(17)
C(26)-C(27)	1.4051(19)
C(27)-H(27)	0.990(18)
C(27)-C(28)	1.3788(18)
C(28)-H(28)	0.988(16)
C(28)-C(29)	1.4081(17)
C(14)-O(1)-C(17)	116.11(10)
C(18)-O(2)-H(2)	111.1(11)
N(2)-N(1)-C(4)	120.10(9)
C(3)-N(1)-N(2)	112.05(9)
C(3)-N(1)-C(4)	127.79(10)
C(1)-N(2)-N(1)	105.15(9)
C(21)-N(3)-C(24)	114.05(10)
C(22)-N(4)-C(29)	114.21(10)
N(2)-C(1)-C(2)	110.86(10)
N(2)-C(1)-C(11)	119.43(10)
C(2)-C(1)-C(11)	129.65(10)

C(1)-C(2)-C(18)	128.65(10)
C(3)-C(2)-C(1)	105.08(10)
C(3)-C(2)-C(18)	126.15(10)
N(1)-C(3)-C(2)	106.87(10)
N(1)-C(3)-H(3)	122.2(9)
C(2)-C(3)-H(3)	131.0(9)
N(1)-C(4)-H(4A)	107.8(9)
N(1)-C(4)-H(4B)	106.7(8)
N(1)-C(4)-C(5)	113.75(9)
H(4A)-C(4)-H(4B)	109.8(12)
C(5)-C(4)-H(4A)	109.3(9)
C(5)-C(4)-H(4B)	109.4(8)
C(6)-C(5)-C(4)	121.05(10)
C(10)-C(5)-C(4)	119.99(10)
C(10)-C(5)-C(6)	118.95(11)
C(5)-C(6)-H(6)	119.5(9)
C(7)-C(6)-C(5)	120.55(11)
C(7)-C(6)-H(6)	119.9(9)
C(6)-C(7)-H(7)	121.0(9)
C(6)-C(7)-C(8)	120.17(11)
C(8)-C(7)-H(7)	118.8(9)
C(7)-C(8)-H(8)	120.3(9)
C(9)-C(8)-C(7)	119.82(11)
C(9)-C(8)-H(8)	119.9(9)
C(8)-C(9)-H(9)	121.6(9)
C(8)-C(9)-C(10)	119.85(11)
C(10)-C(9)-H(9)	118.5(9)
C(5)-C(10)-C(9)	120.65(11)
C(5)-C(10)-H(10)	120.2(9)
C(9)-C(10)-H(10)	119.1(9)
C(12)-C(11)-C(1)	121.56(10)
C(16)-C(11)-C(1)	120.05(10)
C(16)-C(11)-C(12)	118.36(11)
C(11)-C(12)-H(12)	119.7(9)
C(13)-C(12)-C(11)	120.98(11)
C(13)-C(12)-H(12)	119.3(9)
C(12)-C(13)-H(13)	120.8(10)
C(12)-C(13)-C(14)	119.84(11)
C(14)-C(13)-H(13)	119.3(10)
O(1)-C(14)-C(13)	116.23(11)

O(1)-C(14)-C(15)	123.94(11)
C(15)-C(14)-C(13)	119.81(11)
C(14)-C(15)-H(15)	121.9(9)
C(14)-C(15)-C(16)	119.59(11)
C(16)-C(15)-H(15)	118.5(9)
C(11)-C(16)-C(15)	121.34(11)
C(11)-C(16)-H(16)	119.5(10)
C(15)-C(16)-H(16)	119.1(10)
O(1)-C(17)-H(17A)	106.1(10)
O(1)-C(17)-H(17B)	111.5(10)
O(1)-C(17)-H(17C)	111.4(9)
H(17A)-C(17)-H(17B)	109.5(13)
H(17A)-C(17)-H(17C)	110.4(13)
H(17B)-C(17)-H(17C)	107.9(13)
O(2)-C(18)-C(2)	106.52(9)
O(2)-C(18)-C(19)	112.77(9)
O(2)-C(18)-C(22)	111.66(9)
C(2)-C(18)-C(19)	113.59(9)
C(2)-C(18)-C(22)	111.73(9)
C(22)-C(18)-C(19)	100.67(9)
C(20)-C(19)-C(18)	111.95(10)
C(20)-C(19)-C(23)	127.29(11)
C(23)-C(19)-C(18)	120.76(10)
C(19)-C(20)-H(20)	125.3(9)
C(19)-C(20)-C(21)	110.31(10)
C(21)-C(20)-H(20)	124.4(9)
N(3)-C(21)-C(20)	128.55(11)
N(3)-C(21)-C(22)	123.61(11)
C(22)-C(21)-C(20)	107.83(10)
N(4)-C(22)-C(18)	126.61(10)
N(4)-C(22)-C(21)	124.17(11)
C(21)-C(22)-C(18)	109.22(10)
C(19)-C(23)-H(23A)	111.2(10)
C(19)-C(23)-H(23B)	110.1(10)
C(19)-C(23)-H(23C)	111.3(10)
H(23A)-C(23)-H(23B)	107.5(14)
H(23A)-C(23)-H(23C)	109.2(14)
H(23B)-C(23)-H(23C)	107.4(14)
N(3)-C(24)-C(25)	118.44(11)
N(3)-C(24)-C(29)	122.21(10)

C(25)-C(24)-C(29)	119.35(11)
C(24)-C(25)-H(25)	117.3(9)
C(26)-C(25)-C(24)	120.06(12)
C(26)-C(25)-H(25)	122.6(9)
C(25)-C(26)-H(26)	119.1(10)
C(25)-C(26)-C(27)	120.56(12)
C(27)-C(26)-H(26)	120.3(10)
C(26)-C(27)-H(27)	119.4(10)
C(28)-C(27)-C(26)	120.54(12)
C(28)-C(27)-H(27)	120.1(10)
C(27)-C(28)-H(28)	122.1(9)
C(27)-C(28)-C(29)	120.05(12)
C(29)-C(28)-H(28)	117.8(9)
N(4)-C(29)-C(24)	121.69(10)
N(4)-C(29)-C(28)	118.90(11)
C(28)-C(29)-C(24)	119.40(11)

Table S12. Anisotropic displacement parameters ($Å^2$x 10^3) for **15m**. The anisotropic displacement factor exponent takes the form: $-2□^2[h^2 a^{*2}U^{11} + ... + 2 h k a^* b^* U^{12}]$

	U^{11}	U^{22}	U^{33}	U^{23}	U^{13}	U^{12}
O(1)	23(1)	35(1)	32(1)	1(1)	13(1)	-4(1)
O(2)	25(1)	15(1)	34(1)	-2(1)	14(1)	-3(1)
N(1)	17(1)	17(1)	22(1)	0(1)	6(1)	0(1)
N(2)	19(1)	17(1)	24(1)	1(1)	7(1)	-1(1)
N(3)	22(1)	20(1)	25(1)	-2(1)	7(1)	1(1)
N(4)	23(1)	16(1)	22(1)	1(1)	8(1)	0(1)
C(1)	18(1)	16(1)	20(1)	0(1)	4(1)	1(1)
C(2)	18(1)	16(1)	20(1)	-1(1)	5(1)	-1(1)
C(3)	19(1)	16(1)	20(1)	-1(1)	6(1)	-1(1)
C(4)	20(1)	20(1)	22(1)	-1(1)	7(1)	2(1)
C(5)	20(1)	15(1)	21(1)	-1(1)	6(1)	2(1)
C(6)	21(1)	21(1)	27(1)	0(1)	10(1)	-1(1)
C(7)	29(1)	20(1)	23(1)	3(1)	10(1)	3(1)
C(8)	24(1)	24(1)	24(1)	-1(1)	4(1)	3(1)
C(9)	21(1)	24(1)	27(1)	-2(1)	7(1)	-2(1)
C(10)	23(1)	18(1)	23(1)	0(1)	9(1)	-1(1)
C(11)	21(1)	14(1)	27(1)	1(1)	8(1)	1(1)
C(12)	22(1)	27(1)	25(1)	0(1)	6(1)	-1(1)
C(13)	27(1)	32(1)	24(1)	1(1)	11(1)	-1(1)
C(14)	22(1)	20(1)	31(1)	0(1)	13(1)	0(1)
C(15)	21(1)	22(1)	28(1)	1(1)	6(1)	0(1)
C(16)	22(1)	21(1)	24(1)	2(1)	8(1)	0(1)
C(17)	23(1)	28(1)	36(1)	0(1)	11(1)	-3(1)
C(18)	20(1)	15(1)	24(1)	0(1)	8(1)	-1(1)
C(19)	23(1)	15(1)	24(1)	-1(1)	8(1)	-1(1)

C(20)	23(1)	18(1)	24(1)	-2(1)	9(1)	0(1)
C(21)	22(1)	15(1)	24(1)	0(1)	8(1)	1(1)
C(22)	22(1)	13(1)	23(1)	2(1)	8(1)	1(1)
C(23)	24(1)	28(1)	25(1)	-2(1)	6(1)	0(1)
C(24)	24(1)	16(1)	24(1)	0(1)	7(1)	0(1)
C(25)	23(1)	21(1)	29(1)	0(1)	6(1)	2(1)
C(26)	27(1)	24(1)	27(1)	2(1)	2(1)	1(1)
C(27)	33(1)	25(1)	21(1)	1(1)	5(1)	-2(1)
C(28)	30(1)	21(1)	24(1)	0(1)	10(1)	-1(1)
C(29)	23(1)	16(1)	23(1)	2(1)	7(1)	-1(1)

Table S13. Hydrogen coordinates (x 10^4) and isotropic displacement parameters (Å^{2}x 10 ^{3}) for **15m**.

	x	y	z	U(eq)
H(2)	5628(12)	2130(30)	4213(9)	43(5)
H(3)	4436(10)	6100(20)	4389(7)	22(3)
H(4A)	4204(11)	11050(20)	4464(7)	27(4)
H(4B)	3724(10)	9150(20)	4607(7)	22(3)
H(6)	3842(11)	11550(20)	3189(7)	29(4)
H(7)	2532(10)	12020(20)	2241(7)	25(4)
H(8)	1028(11)	10910(20)	2177(7)	27(4)
H(9)	809(11)	9340(20)	3077(7)	28(4)
H(10)	2129(11)	8890(20)	4005(8)	29(4)
H(12)	6263(11)	8740(20)	2884(8)	29(4)
H(13)	7662(12)	9700(30)	2696(9)	40(5)
H(15)	9116(11)	9680(20)	4641(8)	32(4)
H(16)	7729(11)	8760(20)	4815(8)	31(4)
H(17A)	10516(12)	11530(20)	3824(8)	35(4)
H(17B)	10378(11)	9830(20)	4261(8)	31(4)
H(17C)	9920(11)	11850(20)	4319(7)	29(4)
H(20)	7793(10)	3500(20)	3574(7)	26(4)
H(23A)	6101(12)	3310(30)	2727(9)	42(5)
H(23B)	5554(12)	5140(20)	2855(8)	35(4)
H(23C)	5252(12)	3130(20)	3029(8)	38(4)
H(25)	10323(11)	3860(20)	5801(7)	28(4)
H(26)	10706(12)	4390(20)	6916(8)	36(4)
H(27)	9489(12)	5400(20)	7312(8)	36(4)

H(28)	7899(11)	5710(20)	6595(7)	27(4)

Table S14. Torsion angles [°] for **15m**.

O(1)-C(14)-C(15)-C(16)	175.45(11)
O(2)-C(18)-C(19)-C(20)	-118.63(11)
O(2)-C(18)-C(19)-C(23)	60.90(14)
O(2)-C(18)-C(22)-N(4)	-61.42(14)
O(2)-C(18)-C(22)-C(21)	118.91(10)
N(1)-N(2)-C(1)-C(2)	0.18(12)
N(1)-N(2)-C(1)-C(11)	-177.21(9)
N(1)-C(4)-C(5)-C(6)	-60.79(14)
N(1)-C(4)-C(5)-C(10)	120.78(11)
N(2)-N(1)-C(3)-C(2)	0.55(13)
N(2)-N(1)-C(4)-C(5)	87.81(12)
N(2)-C(1)-C(2)-C(3)	0.14(13)
N(2)-C(1)-C(2)-C(18)	-176.04(10)
N(2)-C(1)-C(11)-C(12)	-81.58(14)
N(2)-C(1)-C(11)-C(16)	96.42(13)
N(3)-C(21)-C(22)-N(4)	2.02(18)
N(3)-C(21)-C(22)-C(18)	-178.31(10)
N(3)-C(24)-C(25)-C(26)	-179.25(11)
N(3)-C(24)-C(29)-N(4)	2.19(17)
N(3)-C(24)-C(29)-C(28)	-179.17(11)
C(1)-C(2)-C(3)-N(1)	-0.40(12)
C(1)-C(2)-C(18)-O(2)	-175.81(11)
C(1)-C(2)-C(18)-C(19)	-51.05(16)
C(1)-C(2)-C(18)-C(22)	62.00(15)
C(1)-C(11)-C(12)-C(13)	175.90(11)
C(1)-C(11)-C(16)-C(15)	-176.29(11)
C(2)-C(1)-C(11)-C(12)	101.59(15)
C(2)-C(1)-C(11)-C(16)	-80.41(16)
C(2)-C(18)-C(19)-C(20)	120.05(11)
C(2)-C(18)-C(19)-C(23)	-60.42(14)
C(2)-C(18)-C(22)-N(4)	57.77(14)
C(2)-C(18)-C(22)-C(21)	-121.89(10)
C(3)-N(1)-N(2)-C(1)	-0.46(12)
C(3)-N(1)-C(4)-C(5)	-95.30(13)
C(3)-C(2)-C(18)-O(2)	8.76(15)
C(3)-C(2)-C(18)-C(19)	133.52(11)
C(3)-C(2)-C(18)-C(22)	-113.42(12)
C(4)-N(1)-N(2)-C(1)	176.90(9)
C(4)-N(1)-C(3)-C(2)	-176.55(10)
C(4)-C(5)-C(6)-C(7)	-179.52(11)
C(4)-C(5)-C(10)-C(9)	179.68(10)
C(5)-C(6)-C(7)-C(8)	0.17(18)
C(6)-C(5)-C(10)-C(9)	1.21(17)
C(6)-C(7)-C(8)-C(9)	0.61(18)
C(7)-C(8)-C(9)-C(10)	-0.47(18)
C(8)-C(9)-C(10)-C(5)	-0.45(18)
C(10)-C(5)-C(6)-C(7)	-1.07(17)
C(11)-C(1)-C(2)-C(3)	177.19(11)
C(11)-C(1)-C(2)-C(18)	1.0(2)
C(11)-C(12)-C(13)-C(14)	0.05(19)
C(12)-C(11)-C(16)-C(15)	1.78(17)
C(12)-C(13)-C(14)-O(1)	-175.94(11)
C(12)-C(13)-C(14)-C(15)	2.45(19)
C(13)-C(14)-C(15)-C(16)	-2.81(18)
C(14)-C(15)-C(16)-C(11)	0.68(18)
C(16)-C(11)-C(12)-C(13)	-2.14(18)
C(17)-O(1)-C(14)-C(13)	174.08(11)
C(17)-O(1)-C(14)-C(15)	-4.24(17)
C(18)-C(2)-C(3)-N(1)	175.90(10)
C(18)-C(19)-C(20)-C(21)	0.21(14)
C(19)-C(18)-C(22)-N(4)	178.67(11)
C(19)-C(18)-C(22)-C(21)	-1.00(11)
C(19)-C(20)-C(21)-N(3)	178.58(11)
C(19)-C(20)-C(21)-C(22)	-0.87(13)
C(20)-C(21)-C(22)-N(4)	-178.50(10)
C(20)-C(21)-C(22)-C(18)	1.18(12)

C(21)-N(3)-C(24)-C(25)	178.42(10)
C(21)-N(3)-C(24)-C(29)	-1.50(16)
C(22)-N(4)-C(29)-C(24)	-0.66(16)
C(22)-N(4)-C(29)-C(28)	-179.31(10)
C(22)-C(18)-C(19)-C(20)	0.48(12)
C(22)-C(18)-C(19)-C(23)	-179.99(10)
C(23)-C(19)-C(20)-C(21)	-179.28(11)
C(24)-N(3)-C(21)-C(20)	-179.82(11)
C(24)-N(3)-C(21)-C(22)	-0.45(16)
C(24)-C(25)-C(26)-C(27)	-1.50(19)
C(25)-C(24)-C(29)-N(4)	-177.73(11)
C(25)-C(24)-C(29)-C(28)	0.91(17)
C(25)-C(26)-C(27)-C(28)	0.74(19)
C(26)-C(27)-C(28)-C(29)	0.87(19)
C(27)-C(28)-C(29)-N(4)	177.00(11)
C(27)-C(28)-C(29)-C(24)	-1.67(17)
C(29)-N(4)-C(22)-C(18)	179.08(10)
C(29)-N(4)-C(22)-C(21)	-1.31(16)
C(29)-C(24)-C(25)-C(26)	0.67(18)

Table S15. Hydrogen bonds for **15m** [Å and °].

D-H...A	d(D-H)	d(H...A)	d(D...A)	<(DHA)
O(2)-H(2)...N(2)#1	0.93(2)	1.87(2)	2.7564(13)	156.8(16)

Symmetry transformations used to generate equivalent atoms:
#1 x,y-1,z

X-ray crystallographic data for compound 18:

X-ray diffraction data were collected at 100K on a Rigaku Synergy S diffractometer equipped with a HyPix600HE area-detector (kappa geometry, shutterless ω-scan technique), using monochromatized Cu K_a-radiation. The intensity data were integrated and corrected for absorption and decay by the CrysAlisPro program[1]. The structure was solved by direct methods using SHELXT[2] and refined on F^2 using SHELXL-2018[3] in the OLEX2 program.[4] Positions of all atoms were found from the electron density-difference map. Atoms were refined with individual anisotropic (non-hydrogen atoms) or isotropic (hydrogen atoms) displacement parameters.

Table S16. Crystal data and structure refinement for **18**.

Identification code	**18**
Empirical formula	C20 H24 N2 O4
Formula weight	356.41
Temperature	100.0(1) K
Wavelength	1.54184 Å
Crystal system	Triclinic
Space group	$P\bar{1}$
Unit cell dimensions	a = 7.0064(2) Å □=101.528(3)°.<br>b = 10.6340(4) Å □=101.535(3)°.<br>c = 13.5148(5) Å □=108.514(3)°.
Volume	897.17(6) $Å^3$
Z	2
Density (calculated)	1.319 g/cm^3
Absorption coefficient	0.753 mm^{-1}
F(000)	380
Crystal size	0.167 x 0.042 x 0.024 mm^3
Theta range for data collection	3.480 to 79.797°.
Index ranges	-8<=h<=6, -13<=k<=13, -16<=l<=17
Reflections collected	15589
Independent reflections	3705 [R(int) = 0.0454]
Observed reflections	3149
Completeness to theta = 67.684°	98.9 %
Absorption correction	Semi-empirical from equivalents
Max. and min. transmission	1.00000 and 0.70049
Refinement method	Full-matrix least-squares on F^2
Data / restraints / parameters	3705 / 0 / 332
Goodness-of-fit on F^2	1.032
Final R indices [I>2sigma(I)]	R1 = 0.0484, wR2 = 0.1218
R indices (all data)	R1 = 0.0566, wR2 = 0.1266
Extinction coefficient	0.0026(7)
Largest diff. peak and hole	0.370 and -0.330 e.$Å^{-3}$
CCDC	2159684

Table S17. Atomic coordinates (x 10^4) and equivalent isotropic displacement parameters (Å^{2}x 10^3) for **18**. U(eq) is defined as one third of the trace of the orthogonalized U^{ij} tensor.

	x	y	z	U(eq)
O(1)	10633(2)	8091(1)	4950(1)	24(1)
O(2)	-774(2)	2873(1)	1740(1)	19(1)
O(3)	3453(2)	3802(1)	815(1)	20(1)
O(4)	6112(2)	3356(2)	2507(1)	24(1)
N(1)	-205(2)	6544(2)	966(1)	18(1)
N(2)	1790(2)	7320(2)	1576(1)	16(1)
C(1)	2636(3)	6558(2)	2101(1)	16(1)
C(2)	1119(3)	5226(2)	1790(1)	15(1)
C(3)	-596(3)	5289(2)	1093(1)	17(1)
C(4)	2730(3)	8813(2)	1606(2)	20(1)
C(5)	4875(3)	9109(2)	1393(2)	27(1)
C(6)	2903(3)	9715(2)	2679(2)	25(1)
C(7)	1292(3)	9100(2)	745(2)	27(1)
C(8)	4751(3)	7067(2)	2862(1)	16(1)
C(9)	6388(3)	6783(2)	2537(2)	18(1)
C(10)	8318(3)	7141(2)	3254(2)	20(1)
C(11)	8671(3)	7792(2)	4311(2)	19(1)
C(12)	7066(3)	8092(2)	4652(2)	19(1)
C(13)	5124(3)	7722(2)	3921(1)	18(1)
C(14)	11065(3)	8740(2)	6049(2)	24(1)
C(15)	1248(3)	3961(2)	2093(1)	16(1)
C(16)	2706(3)	3329(2)	1611(1)	17(1)
C(17)	4509(3)	3554(2)	2553(2)	18(1)
C(18)	3918(3)	3967(2)	3503(2)	19(1)
C(19)	2161(3)	4228(2)	3277(1)	17(1)
C(20)	1087(3)	4696(2)	4039(2)	20(1)

_

Table S18. Bond lengths [Å] and angles [°] for **18**.

O(1)-C(11)	1.370(2)
O(1)-C(14)	1.431(2)
O(2)-H(2)	0.86(3)
O(2)-C(15)	1.429(2)
O(3)-H(3)	0.97(3)
O(3)-C(16)	1.399(2)
O(4)-C(17)	1.218(2)
N(1)-N(2)	1.360(2)
N(1)-C(3)	1.329(2)
N(2)-C(1)	1.370(2)
N(2)-C(4)	1.502(2)
C(1)-C(2)	1.393(2)
C(1)-C(8)	1.485(2)
C(2)-C(3)	1.399(2)
C(2)-C(15)	1.507(2)
C(3)-H(3A)	0.99(3)
C(4)-C(5)	1.533(3)
C(4)-C(6)	1.529(3)
C(4)-C(7)	1.527(3)
C(5)-H(5A)	0.97(3)
C(5)-H(5B)	0.96(3)
C(5)-H(5C)	1.04(3)
C(6)-H(6A)	1.05(3)
C(6)-H(6B)	0.95(3)
C(6)-H(6C)	1.01(3)
C(7)-H(7A)	0.98(2)
C(7)-H(7B)	1.02(3)
C(7)-H(7C)	0.99(3)
C(8)-C(9)	1.403(2)
C(8)-C(13)	1.392(3)
C(9)-H(9)	0.96(2)
C(9)-C(10)	1.383(3)
C(10)-H(10)	0.95(2)
C(10)-C(11)	1.391(3)
C(11)-C(12)	1.399(3)
C(12)-H(12)	0.96(3)
C(12)-C(13)	1.396(2)
C(13)-H(13)	0.99(2)
C(14)-H(14A)	1.00(2)
C(14)-H(14B)	0.99(2)
C(14)-H(14C)	1.00(2)
C(15)-C(16)	1.564(2)
C(15)-C(19)	1.531(2)
C(16)-H(16)	0.98(2)
C(16)-C(17)	1.523(2)
C(17)-C(18)	1.454(3)
C(18)-H(18)	0.97(3)
C(18)-C(19)	1.337(3)
C(19)-C(20)	1.489(2)
C(20)-H(20A)	0.98(3)
C(20)-H(20B)	0.97(3)
C(20)-H(20C)	1.01(2)
C(11)-O(1)-C(14)	117.78(15)
C(15)-O(2)-H(2)	114(2)
C(16)-O(3)-H(3)	106.9(18)
C(3)-N(1)-N(2)	105.68(14)
N(1)-N(2)-C(1)	111.12(14)
N(1)-N(2)-C(4)	119.22(14)
C(1)-N(2)-C(4)	129.65(15)
N(2)-C(1)-C(2)	106.59(15)
N(2)-C(1)-C(8)	126.37(16)
C(2)-C(1)-C(8)	127.04(16)
C(1)-C(2)-C(3)	104.87(15)
C(1)-C(2)-C(15)	129.02(15)
C(3)-C(2)-C(15)	126.10(15)
N(1)-C(3)-C(2)	111.72(15)
N(1)-C(3)-H(3A)	119.7(14)
C(2)-C(3)-H(3A)	128.6(14)
N(2)-C(4)-C(5)	109.52(15)
N(2)-C(4)-C(6)	108.82(15)
N(2)-C(4)-C(7)	109.01(15)
C(6)-C(4)-C(5)	111.73(16)
C(7)-C(4)-C(5)	108.56(17)
C(7)-C(4)-C(6)	109.17(16)
C(4)-C(5)-H(5A)	109.3(15)

C(4)-C(5)-H(5B)	113.2(14)
C(4)-C(5)-H(5C)	107.8(13)
H(5A)-C(5)-H(5B)	108(2)
H(5A)-C(5)-H(5C)	110(2)
H(5B)-C(5)-H(5C)	109(2)
C(4)-C(6)-H(6A)	108.8(15)
C(4)-C(6)-H(6B)	111.5(16)
C(4)-C(6)-H(6C)	108.0(14)
H(6A)-C(6)-H(6B)	110(2)
H(6A)-C(6)-H(6C)	107(2)
H(6B)-C(6)-H(6C)	111(2)
C(4)-C(7)-H(7A)	110.3(14)
C(4)-C(7)-H(7B)	108.5(14)
C(4)-C(7)-H(7C)	112.5(15)
H(7A)-C(7)-H(7B)	110(2)
H(7A)-C(7)-H(7C)	110(2)
H(7B)-C(7)-H(7C)	106(2)
C(9)-C(8)-C(1)	119.73(16)
C(13)-C(8)-C(1)	121.75(15)
C(13)-C(8)-C(9)	118.28(16)
C(8)-C(9)-H(9)	120.4(13)
C(10)-C(9)-C(8)	120.66(17)
C(10)-C(9)-H(9)	118.9(13)
C(9)-C(10)-H(10)	120.4(14)
C(9)-C(10)-C(11)	120.57(16)
C(11)-C(10)-H(10)	119.1(14)
O(1)-C(11)-C(10)	115.62(16)
O(1)-C(11)-C(12)	124.61(17)
C(10)-C(11)-C(12)	119.77(16)
C(11)-C(12)-H(12)	120.6(14)
C(13)-C(12)-C(11)	119.11(17)
C(13)-C(12)-H(12)	120.3(14)
C(8)-C(13)-C(12)	121.60(16)
C(8)-C(13)-H(13)	119.6(14)

C(12)-C(13)-H(13)	118.8(14)
O(1)-C(14)-H(14A)	110.8(14)
O(1)-C(14)-H(14B)	103.7(14)
O(1)-C(14)-H(14C)	111.9(14)
H(14A)-C(14)-H(14B)	107.8(19)
H(14A)-C(14)-H(14C)	110.7(19)
H(14B)-C(14)-H(14C)	111.6(19)
O(2)-C(15)-C(2)	110.43(13)
O(2)-C(15)-C(16)	105.24(13)
O(2)-C(15)-C(19)	109.37(14)
C(2)-C(15)-C(16)	114.87(14)
C(2)-C(15)-C(19)	113.59(14)
C(19)-C(15)-C(16)	102.76(13)
O(3)-C(16)-C(15)	118.87(14)
O(3)-C(16)-H(16)	111.7(13)
O(3)-C(16)-C(17)	111.14(14)
C(15)-C(16)-H(16)	105.8(13)
C(17)-C(16)-C(15)	104.52(14)
C(17)-C(16)-H(16)	103.4(13)
O(4)-C(17)-C(16)	125.42(17)
O(4)-C(17)-C(18)	126.58(17)
C(18)-C(17)-C(16)	107.91(14)
C(17)-C(18)-H(18)	124.3(15)
C(19)-C(18)-C(17)	110.93(17)
C(19)-C(18)-H(18)	124.7(15)
C(18)-C(19)-C(15)	112.41(15)
C(18)-C(19)-C(20)	126.81(17)
C(20)-C(19)-C(15)	120.77(15)
C(19)-C(20)-H(20A)	109.8(15)
C(19)-C(20)-H(20B)	112.1(15)
C(19)-C(20)-H(20C)	109.8(14)
H(20A)-C(20)-H(20B)	110(2)
H(20A)-C(20)-H(20C)	105(2)
H(20B)-C(20)-H(20C)	110(2)

Table S19. Anisotropic displacement parameters ($Å^2 x 10^3$) for **18**. The anisotropic displacement factor exponent takes the form: $-2\pi^2[h^2 a^{*2}U^{11} + ... + 2 h k a^* b^* U^{12}]$

	U^{11}	U^{22}	U^{33}	U^{23}	U^{13}	U^{12}
O(1)	15(1)	28(1)	24(1)	4(1)	0(1)	8(1)
O(2)	12(1)	19(1)	24(1)	3(1)	4(1)	6(1)
O(3)	18(1)	26(1)	18(1)	7(1)	6(1)	10(1)
O(4)	16(1)	34(1)	28(1)	10(1)	7(1)	15(1)
N(1)	13(1)	21(1)	17(1)	3(1)	1(1)	8(1)
N(2)	16(1)	17(1)	17(1)	3(1)	2(1)	8(1)
C(1)	16(1)	18(1)	17(1)	4(1)	6(1)	9(1)
C(2)	12(1)	19(1)	15(1)	2(1)	4(1)	8(1)
C(3)	13(1)	20(1)	17(1)	3(1)	3(1)	8(1)
C(4)	22(1)	16(1)	21(1)	4(1)	4(1)	8(1)
C(5)	28(1)	25(1)	31(1)	12(1)	11(1)	10(1)
C(6)	29(1)	22(1)	22(1)	3(1)	3(1)	11(1)
C(7)	31(1)	21(1)	26(1)	7(1)	-1(1)	12(1)
C(8)	14(1)	15(1)	21(1)	6(1)	4(1)	6(1)
C(9)	17(1)	19(1)	20(1)	5(1)	6(1)	8(1)
C(10)	15(1)	20(1)	26(1)	6(1)	8(1)	9(1)
C(11)	14(1)	18(1)	24(1)	7(1)	2(1)	6(1)
C(12)	17(1)	19(1)	17(1)	3(1)	3(1)	6(1)
C(13)	14(1)	18(1)	21(1)	6(1)	6(1)	7(1)
C(14)	22(1)	24(1)	22(1)	6(1)	0(1)	6(1)
C(15)	11(1)	17(1)	18(1)	2(1)	3(1)	5(1)
C(16)	16(1)	18(1)	19(1)	4(1)	5(1)	8(1)
C(17)	16(1)	16(1)	22(1)	6(1)	5(1)	6(1)
C(18)	17(1)	20(1)	20(1)	6(1)	3(1)	6(1)
C(19)	15(1)	16(1)	18(1)	5(1)	5(1)	6(1)
C(20)	20(1)	24(1)	19(1)	6(1)	6(1)	12(1)

Table S20. Hydrogen coordinates (x 10^4) and isotropic displacement parameters ($Å^2$x 10^3) for **18**.

	x	y	z	U(eq)
H(2)	-1740(50)	3120(30)	1920(20)	43(8)
H(3)	2240(50)	3510(30)	200(30)	49(8)
H(3A)	-1980(40)	4540(30)	710(20)	29(6)
H(5A)	4710(40)	8500(30)	720(20)	33(6)
H(5B)	5880(40)	8970(20)	1920(20)	26(6)
H(5C)	5440(40)	10130(30)	1370(20)	29(6)
H(6A)	3460(40)	10750(30)	2680(20)	38(7)
H(6B)	3800(40)	9570(30)	3230(20)	34(7)
H(6C)	1440(40)	9490(30)	2770(20)	30(6)
H(7A)	1120(40)	8500(20)	50(20)	24(6)
H(7B)	1950(40)	10120(30)	770(20)	32(6)
H(7C)	-100(40)	8970(30)	860(20)	32(6)
H(9)	6170(30)	6310(20)	1815(19)	19(5)
H(10)	9410(40)	6940(20)	3032(18)	21(5)
H(12)	7300(40)	8560(20)	5380(20)	25(6)
H(13)	3990(40)	7920(20)	4168(19)	24(6)
H(14A)	11140(40)	9720(30)	6171(19)	25(6)
H(14B)	12490(40)	8770(20)	6359(19)	25(6)
H(14C)	10010(40)	8200(20)	6364(19)	24(6)
H(16)	1950(30)	2320(20)	1386(17)	18(5)
H(18)	4710(40)	4060(30)	4200(20)	32(6)
H(20A)	1280(40)	5660(30)	4110(20)	29(6)
H(20B)	1590(40)	4580(30)	4730(20)	30(6)
H(20C)	-480(40)	4180(20)	3752(19)	26(6)

Table S21. Torsion angles [°] for **18**.

O(1)-C(11)-C(12)-C(13)	-179.45(17)
O(2)-C(15)-C(16)-O(3)	110.52(17)
O(2)-C(15)-C(16)-C(17)	-124.85(14)
O(2)-C(15)-C(19)-C(18)	116.85(16)
O(2)-C(15)-C(19)-C(20)	-61.9(2)
O(3)-C(16)-C(17)-O(4)	-41.5(2)
O(3)-C(16)-C(17)-C(18)	141.63(15)
O(4)-C(17)-C(18)-C(19)	173.76(18)
N(1)-N(2)-C(1)-C(2)	1.39(19)
N(1)-N(2)-C(1)-C(8)	-177.96(16)
N(1)-N(2)-C(4)-C(5)	-128.98(17)
N(1)-N(2)-C(4)-C(6)	108.63(17)
N(1)-N(2)-C(4)-C(7)	-10.3(2)
N(2)-N(1)-C(3)-C(2)	0.46(19)
N(2)-C(1)-C(2)-C(3)	-1.03(18)
N(2)-C(1)-C(2)-C(15)	177.56(16)
N(2)-C(1)-C(8)-C(9)	-101.2(2)
N(2)-C(1)-C(8)-C(13)	84.5(2)
C(1)-N(2)-C(4)-C(5)	52.1(2)
C(1)-N(2)-C(4)-C(6)	-70.3(2)
C(1)-N(2)-C(4)-C(7)	170.72(17)
C(1)-C(2)-C(3)-N(1)	0.36(19)
C(1)-C(2)-C(15)-O(2)	170.12(16)
C(1)-C(2)-C(15)-C(16)	-71.1(2)
C(1)-C(2)-C(15)-C(19)	46.8(2)
C(1)-C(8)-C(9)-C(10)	-174.29(17)
C(1)-C(8)-C(13)-C(12)	174.37(16)
C(2)-C(1)-C(8)-C(9)	79.6(2)
C(2)-C(1)-C(8)-C(13)	-94.8(2)
C(2)-C(15)-C(19)-C(18)	-119.29(17)
C(2)-C(15)-C(19)-C(20)	61.9(2)
C(3)-N(1)-N(2)-C(1)	-1.15(19)
C(3)-N(1)-N(2)-C(4)	179.73(15)
C(3)-C(2)-C(15)-O(2)	-11.6(2)
C(3)-C(2)-C(15)-C(16)	107.23(19)
C(3)-C(2)-C(15)-C(19)	-134.86(17)
C(4)-N(2)-C(1)-C(2)	-179.61(16)
C(4)-N(2)-C(1)-C(8)	1.0(3)
C(8)-C(1)-C(2)-C(3)	178.31(16)
C(8)-C(1)-C(2)-C(15)	-3.1(3)
C(8)-C(9)-C(10)-C(11)	-0.1(3)
C(9)-C(8)-C(13)-C(12)	-0.1(3)
C(9)-C(10)-C(11)-O(1)	179.65(16)
C(9)-C(10)-C(11)-C(12)	-0.3(3)
C(10)-C(11)-C(12)-C(13)	0.4(3)
C(11)-C(12)-C(13)-C(8)	-0.3(3)
C(13)-C(8)-C(9)-C(10)	0.3(3)
C(14)-O(1)-C(11)-C(10)	-178.95(16)
C(14)-O(1)-C(11)-C(12)	0.9(3)
C(15)-C(2)-C(3)-N(1)	-178.28(16)
C(15)-C(16)-C(17)-O(4)	-170.92(17)
C(15)-C(16)-C(17)-C(18)	12.22(18)
C(16)-C(15)-C(19)-C(18)	5.43(19)
C(16)-C(15)-C(19)-C(20)	-173.34(15)
C(16)-C(17)-C(18)-C(19)	-9.4(2)
C(17)-C(18)-C(19)-C(15)	2.3(2)
C(17)-C(18)-C(19)-C(20)	-179.01(17)
C(19)-C(15)-C(16)-O(3)	-135.02(15)
C(19)-C(15)-C(16)-C(17)	-10.39(17)

Table S22. Hydrogen bonds for **18** [Å and °].

D-H...A	d(D-H)	d(H...A)	d(D...A)	<(DHA)
O(2)-H(2)...O(4)#1	0.86(3)	1.90(3)	2.7390(18)	166(3)
O(3)-H(3)...N(1)#2	0.97(3)	1.88(3)	2.8333(19)	165(3)

Symmetry transformations used to generate equivalent atoms: #1 x-1,y,z #2 -x,-y+1,-z

10. References

1. Komogortsev, A.N.; Lichitsky, B.V.; Tretyakov, A.D.; Fakhrutdinov, A.N.; Dudinov, A.A.; Krayushkin, M.M. *J. Heterocyclic. Chem*. **2019**, *56*, 3081-3087.
2. Komogortsev, A.N.; Melekhina, V.G.; Lichitsky, B.V.; Dudinov, A.A.; Fakhrutdinov, A.N.; Krayushkin, M.M. *Russ. Chem. Bull*. **2020**, *69*, 4, 758-762.
3. CrysAlisPro. Version 1.171.41. *Rigaku Oxford Diffraction*, **2021**.
4. Sheldrick, G. M. SHELXT - Integrated space-group and crystal-structure determination. *Acta Cryst.* **2015**, A71(1), 3-8. http://doi.org/10.1107/S2053273314026370
5. Sheldrick, G. M. Crystal structure refinement with SHELXL. *Acta Cryst.* **2015**, C71(1), 3-8. http://doi.org/10.1107/S2053229614024218
6. Dolomanov O.V.; Bourhis L.J.; Gildea R.J.; Howard J.A.K.; Puschmann H. OLEX2: a complete structure solution, refinement and analysis program. *J. Appl. Cryst.* **2009**, 42(2), 229-341. http://doi.org/10.1107/S0021889808042726
